# On the Role of Noncovalent Ligand-Substrate Interactions in Au(I) Catalysis: An Experimental and Computational Study of Protodeauration

**DOI:** 10.1021/acscatal.2c03384

**Published:** 2022-10-13

**Authors:** Taegeun Jo, Svenja Taschinski, Isaac F. Leach, Christina Bauer, A. Stephen K. Hashmi, Johannes E. M. N. Klein

**Affiliations:** † Molecular Inorganic Chemistry, Stratingh Institute for Chemistry, Faculty of Science and Engineering, University of Groningen, Nijenborgh 4, 9747 AG Groningen, The Netherlands; ‡ Organisch-Chemisches Institut, Heidelberg University, Im Neuenheimer Feld 270, 69120 Heidelberg, Germany

**Keywords:** protodeauration, gold catalysis, vinyl gold(I) complexes, noncovalent interactions (NCIs), DFT calculations

## Abstract

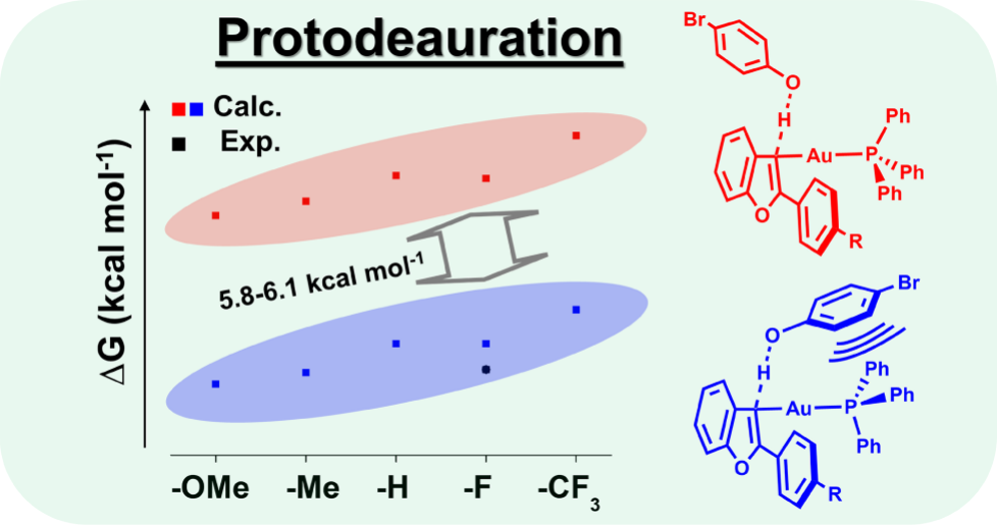

A systematic study of protodeauration, a crucial step often found in gold catalysis, was performed using isolated vinyl gold(I) complexes. By varying substituents on gold complexes, we explore how their properties influence protodeauration. Phenols were employed as the proton source, and their substituents were also varied, providing insight through variation of their acidity. A linear Hammett correlation is identified for the series of substituted vinyl gold(I) complexes, while a nonlinear trend is found for the series of substituted phenols. Computationally, we reproduce our experimental observations and identify significant noncovalent interactions (NCIs) between the proton donor and vinyl gold(I) complexes. This finding is of particular importance for gold-catalyzed reactions as they often employ linear two-coordinate complexes where the site of the reaction is spatially remote from the ligand bound to gold. The NCIs between substrates and intermediates lead to a significant acceleration of the protodeauration step in this work, opening the door to alternative strategies in the field of gold catalysis.

**Introduction**

Transformations catalyzed by gold and involving substrates containing unsaturated C-C bonds (e.g., alkynes, allenes, and alkenes) have been extensively studied over the last few decades.^1^ These types of reactions often follow a similar pathway (Scheme 1), where two key steps may be highlighted.^1e,1i,2^ Frequently, a gold catalyst will interact with a substrate, leading to a vinyl gold complex as an intermediate which results from the activation of π-bonds present in the substrate.^3^ Notably, only a limited number of vinyl gold(I) complexes have been reported to date, which could be characterized or even isolated.^4^ The second crucial step is the subsequent reaction of the said vinyl gold(I) intermediate, which, in the presence of a proton source (or, more generally, an electrophile), undergoes protodeauration to release the final product—thereby completing the catalytic cycle.^5^ Due to the fundamental relevance of these steps to the field of gold catalysis, several efforts have been made to gain detailed mechanistic insight. However, gaining insight into the whole catalytic cycle might be challenging because the two key steps highlighted above generally have opposing requirements. For example, electron-withdrawing groups (EWGs) on the spectator ligands promote reaction rates, likely resulting from the acceleration of the first step (formation of the vinyl gold complex).^6^ On the other hand, protodeauration tends to be accelerated by electron-donating groups (EDGs) on the spectator ligands.^7^ For example, Wang et al.^4i^ reported systematic studies on ligand effects in the gold-catalyzed activation of alkynes. In their systems, EDGs increase the reaction rates by promoting the (rate-limiting) protodeauration step. Even though these reports propose catalytic cycles that fall into the same general scheme, they have opposing electronic requirements since the rate-determining step is either the formation of the vinyl complex or protodeauration. Therefore, obtaining a detailed understanding of these systems during catalysis becomes generally difficult.

**Scheme 1:**
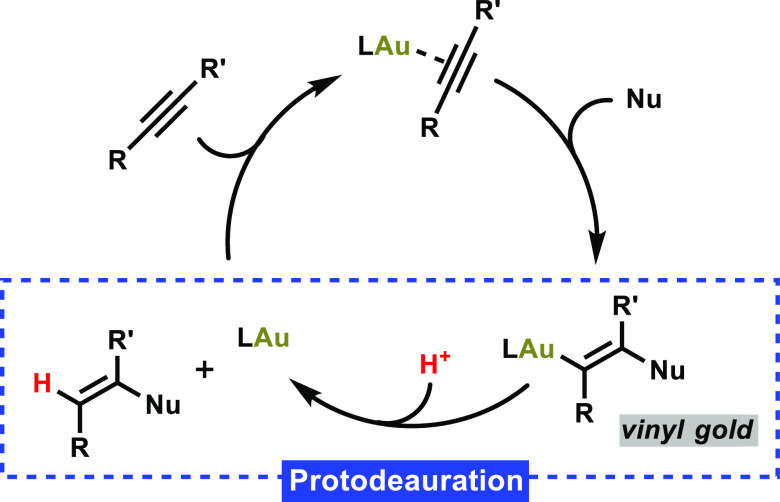
Schematic Catalytic Cycle between a Gold Catalyst and a Substrate Featuring a C–C Triple Bond

In a previous study, we reported the preparation of vinyl gold(I) complexes^8^ based on earlier reports^3a,4e^ (Scheme 2) and found that these complexes are indeed rather easily handled. In this communication, we decided to design a study with the intent of probing the protodeauration step in an isolated fashion, where our result would not be complicated by the surrounding steps taking place in gold-catalyzed reactions.

**Scheme 2:**
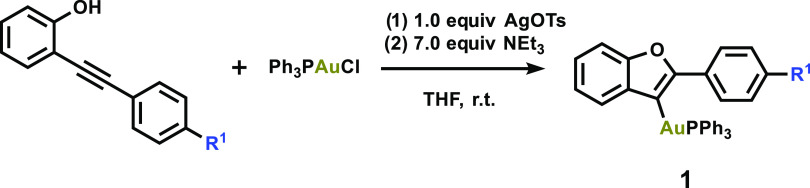
Synthesis of Vinyl Gold(I) Complexes with Systematic Variation

We prepared vinyl Au(I) complexes (**1**_**R**_) with different substituents placed on the benzofuran unit following our previously reported procedure (Scheme 2).^4e^ The resulting complexes **1** were isolated in good yields (72–81%). With these well-characterized and isolated vinyl gold(I) complexes in hand, we conducted systematic kinetic studies focusing on the protodeauration step. We first focused on the effect of placing substituents on the benzofuran units in the vinyl gold(I) complexes and then exposing these complexes to 4-bromophenol as an acid (Figure 1).

**Figure 1:**
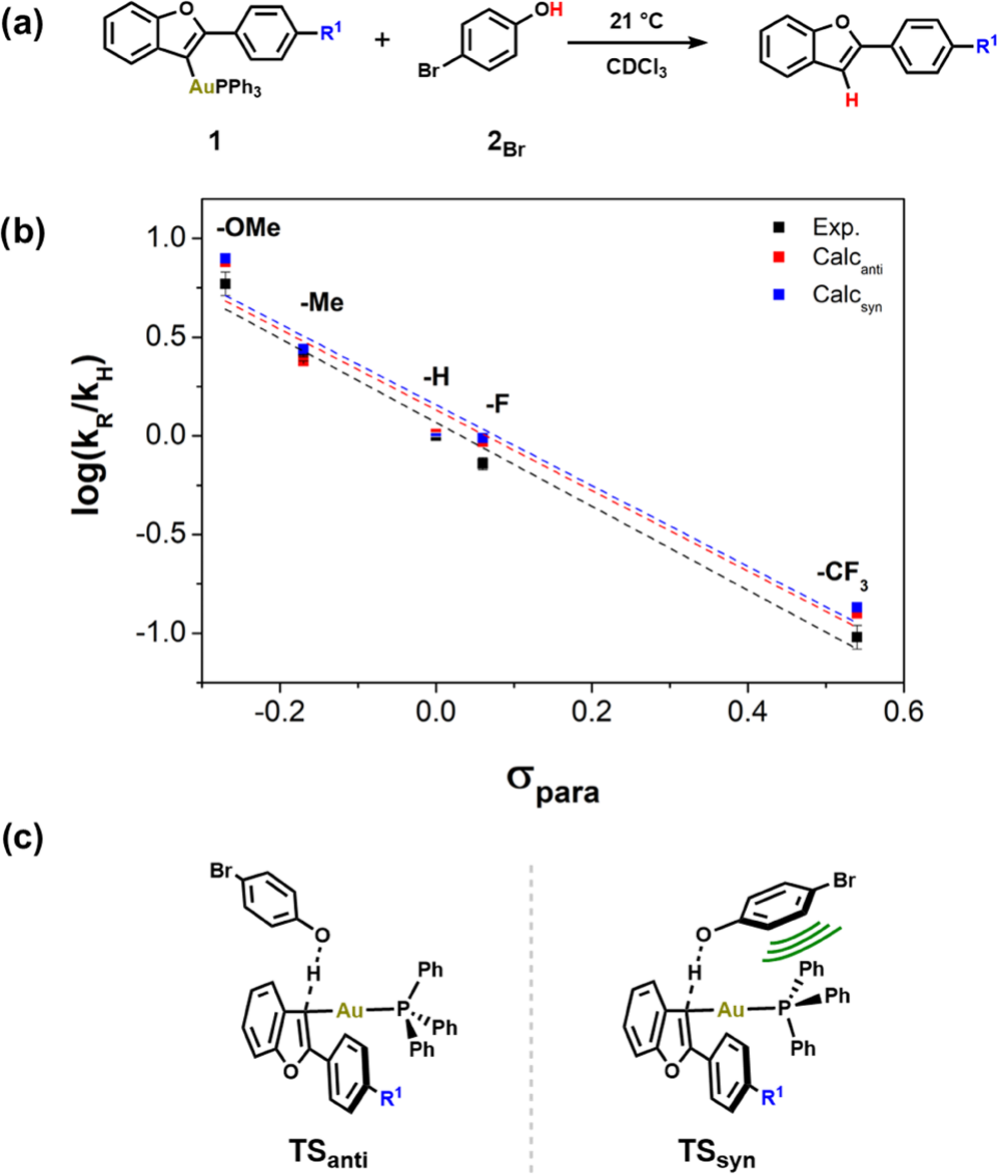
(a) Reaction scheme of the protodeauration of various substituted vinyl Au(I) complexes (**1**_**R**_) with a 4-bromophenol. (b) Experimentally obtained Hammett correlations of σ_para_, the *para*-Hammett substituent parameter, vs. log(*k*_R_/*k*_H_) (black dashed line and squares). Theoretically obtained Hammett correlations with PW6B95-D3(BJ)/def2-TZVPP/cPCM//PBEh-3c/cPCM (*anti*-conformation: red dashed line and squares and *syn*-conformation: blue dashed line and squares). (c) Simplified TS structures based on the conformation.

The rates of reaction were recorded by ^1^H NMR spectroscopy (Tables S1–S6). A clear trend was observed, as expected; EDGs accelerate the rates of reaction (up to ca. 100×), and vice versa, EWGs result in reduced rates (Figure 1b). For these complexes, a linear Hammett correlation between σ_para_ and log(*k*_R_/*k*_H_) was found (ρ_exp._ = −2.19), which indicates the built-up of positive charge in the vinyl gold(I) complexes during protonation. This observation is, in principle, not surprising. This did, however, change once we explored density functional theory (DFT) calculations. Reaction paths for the protodeauration were optimized at the PBEh-3c/cPCM(CHCl_3_) level of theory^9^ (which includes the D3 model for dispersion effects and gCP corrections for the basis set superposition error),^10^ and electronic energies were refined using the PW6B95-D3(BJ) functional^11^ in combination with the larger def2-TZVPP^12^ basis set (for full computational details see the Supporting Information). Two conformers (TS_syn_ and TS_anti_) were identified, which differ in the proximity of the phenol and gold-phosphine moieties (Figure 1c). For both sets of structures, we computed Hammett plots corresponding to the *syn* and *anti* conformers.^13^ Although the slopes for both computed series match the slope obtained from our experimental data (*ρ*_calc-anti_ = −2.11 and *ρ*_calc-syn_ = −2.12), the free energies of activation for TS_syn_ are consistently lower than those of TS_anti_, by 5.8–6.6 kcal mol^–1^ independent of the nature of the substituent (Figure 2).

**Figure 2:**
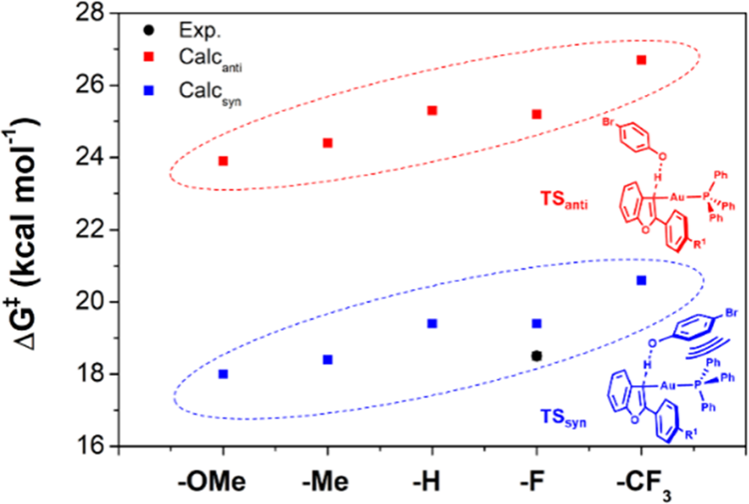
Computed activation energies of **1**_**R**_ (R = OMe, Me, H, F, CF_3_) in the *anti* (red) and *syn* (blue) conformations regarding the orientation of 4-bromophenol. The experimentally obtained free energy of activation for **1**_**F**_ is plotted (black) for comparison.

For the F-substituted vinyl complex (**1**_**F**_), we determined the free energy of activation experimentally for the protodeauration. Upon addition of different amounts of 4-bromophenol (50, 75, 100, and 125 equiv) to **1**_**F**_, a second-order rate constant for this reaction was obtained (*k*_2_ = 1.48 × 10^–1^ M^–1^ s^–1^) (Figure S16), which corresponds to a barrier of Δ*G*_294.6_^‡^ = 18.5 kcal mol^–1^. Therefore, we conclude that the most relevant conformers for the TS are the computed *syn*-conformers because: (i) The *syn*-conformers are consistently calculated to be energetically favorable by ca. 6 kcal mol^–1^ and (ii) the experimentally obtained free energy of activation of protodeauration for the F-substituted complex **1**_**F**_ is in good agreement with our findings for the *syn*-conformer (Δ*G*_298.2_^‡^(*syn*, *anti*) = (19.4, 25.2) kcal mol^–1^). The energetic preference for the *syn*-conformation is remarkable, as one may expect no significant interactions between the phenol and gold-phosphine moieties since these are oriented *trans* to the vinyl gold moiety. If any interaction is to be expected, it might be an unfavorable steric clash, resulting in a preference for the *anti*-conformation. We thus began to explore the origin of this difference in barrier heights, ΔΔ*G*_anti-syn_^‡^. Since the key structural difference between the conformations is the proximity of the phenol′s aromatic system to the gold-phosphine moiety (Figure 1c), we suspected favorable noncovalent interactions (NCIs) between these sites might be playing a crucial role. Such interactions have been well studied in other fields and have been found to significantly affect reaction barriers.^14^ Although these studies clearly show the variation of NCIs upon the change of substituents, the observed ΔΔ*G*_anti-syn_^‡^ remains almost constant regardless of the substituents on the vinyl substituent. *R*^1^ is rather far removed from the interacting sites, resulting in a negligible change of NCIs due to substitution. The electronic effects from substitution are, therefore, solely responsible for the observed linear Hammett correlation. How might we experimentally probe the role of NCIs leading to a preferential *syn*-conformation in the protodeauration step? If NCIs between the phenol and gold-phosphine moieties are playing a significant role in the reaction, we should expect them to depend on the nature of the substituent present in the phenol. As the Hammett correlation identified for the substitution at the vinyl moiety indicated a linear correlation resulting from substituent effects, we may anticipate that substitution at the phenol would also result in a linear correlation in the absence of relevant NCIs. However, should the NCIs become relevant, as in the *syn*-conformation, a perturbation of this linear correlation may be observed, as has been indicated before for linear free energy relationships.^15^ We accordingly introduced substituents at the *para* position of the phenol, which will probe the significance of the aforementioned NCIs.

The protodeauration of **1**_**OMe**_ with *para*-substituted phenols was monitored by ^1^H NMR spectroscopy to observe the reaction rate (*k*_obs_) when varying the phenol substituents. In contrast to the previous series (**1** and **2**_**Br**_), EWGs accelerated the reaction in this series (**1**_**OMe**_) and *para*-substituted phenols (**2**) (Figure 3, see black squares). The result indicates a build-up of negative charge at the phenols during the reaction, forming a phenolate anion stabilized by the EWGs, i.e., the phenol acts as an acid. Although log(*k*_R_/*k*_H_) generally increases with *σ*_para_, we did not obtain a reasonably linear correlation, unlike in the previous case (Figure 1). This nonlinearity indicates that the expected acidity effects (accounted for in the Hammett σ_para_ values) do not adequately explain the trend. To further probe these experimental results, we optimized all *syn* and *anti* pathways and calculated log(*k*_R_/*k*_H_)_calc_ (Figure 3, see red and blue squares). The computed data also exhibit a trend similar to the experimentally observed one. It appears that the interaction between the phenol unit and the complex (TS_syn_) perturbs the slope in the Hammett plot, similar to the experimental data. These results are noticeably different from the previous ones (Figure 1), where a linear correlation was found regardless of conformation. Furthermore, ΔΔ*G*_syn-anti_^‡^ shows a more significant variability (5.2–8.2 kcal mol^–1^), leading us to conclude that the magnitude of the NCIs varies considerably in this series *R*^2^ = (NMe_2_, OMe, H, F, Cl, Br).

**Figure 3:**
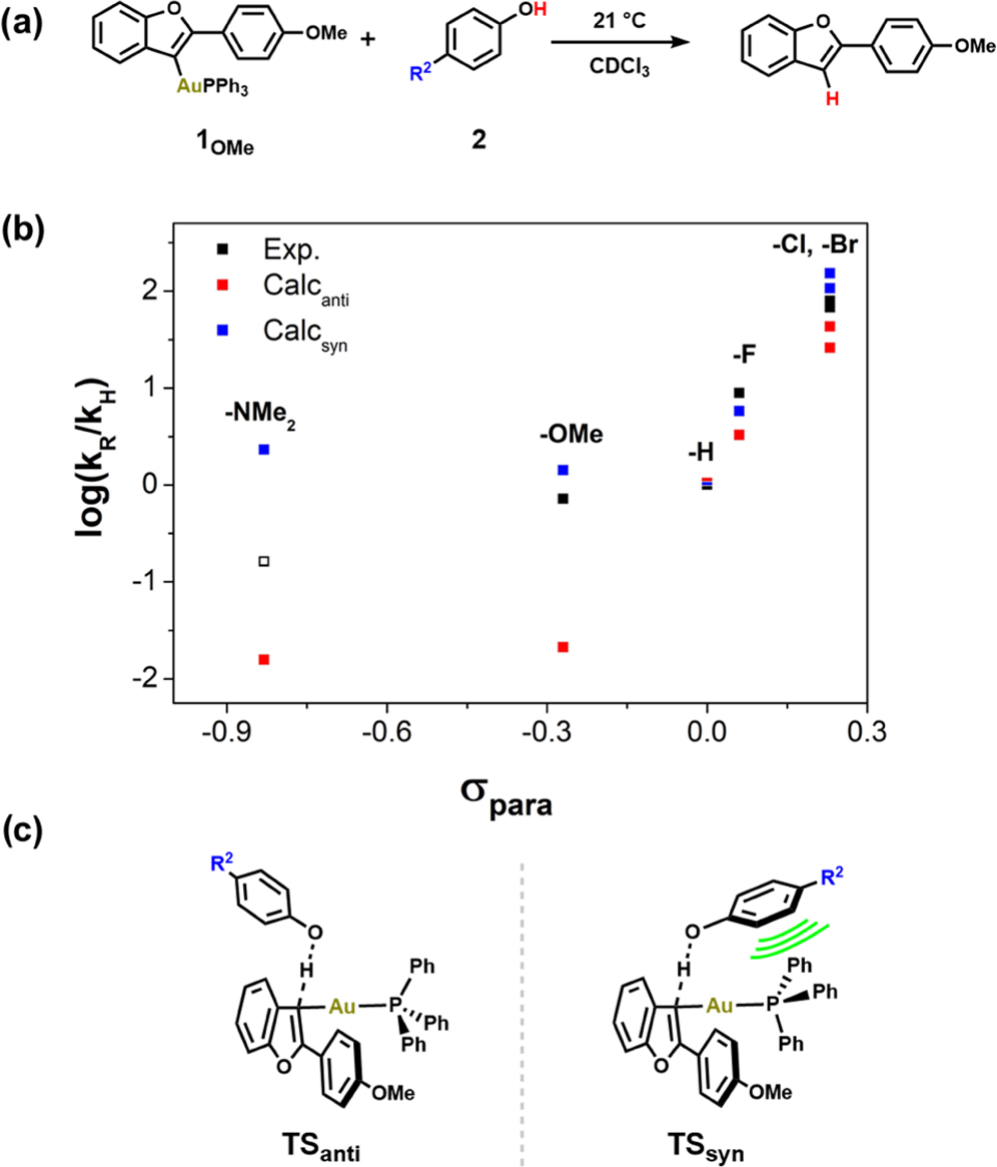
(a) Reaction scheme of the protodeauration of **1**_**OMe**_ with *para*-substituted phenols. (b) Experimentally obtained Hammett correlations of σ_para_, the *para*-Hammett substituent parameter, vs. log(*k*_R_/*k*_H_) (black squares). The data for 4-dimethylaminophenol (**2**_**NMe2**_) are represented as an empty box as the reaction was not followed completely due to the slow rate. Computationally obtained Hammett correlations with PW6B95-D3(BJ)/def2-TZVPP/cPCM//PBEh-3c/cPCM (*anti*-conformation: red squares and *syn*-conformation: blue squares). (c) Qualitative depictions of TS structures for the *anti-* and *syn*-conformation.

To quantify the effect of the dispersion interaction on the observed kinetic trends, we performed a series of calculations with the B3LYP^16^-D3(BJ)^10a^ functional at the (PBEh-3c) optimized geometries. The intrinsic parameterization of the B3LYP functional is well known to poorly describe dispersion effects,^17,18^ allowing us to mostly separate the dispersion energy (Table S23) between the phenol and the vinyl gold(I) complex, *E*(disp), from the electronic barrier height, Δ*E*^‡^. The *anti*-*syn* difference in barrier height (ΔΔ*E*_anti-syn_^‡^) generally increases with the *anti*-*syn* difference in the dispersion energies of the transition states (Δ*E*(disp)_anti-syn_) (Figure 4). The reduction in barrier height (when going from *anti* to *syn*) is more than accounted for by the dispersion interaction between the phenol and the vinyl gold(I) complex. We note that the difference in dispersion energy does not directly translate into a reduction in barrier height due to some minor destabilizing effects, e.g., steric clashes.

**Figure 4:**
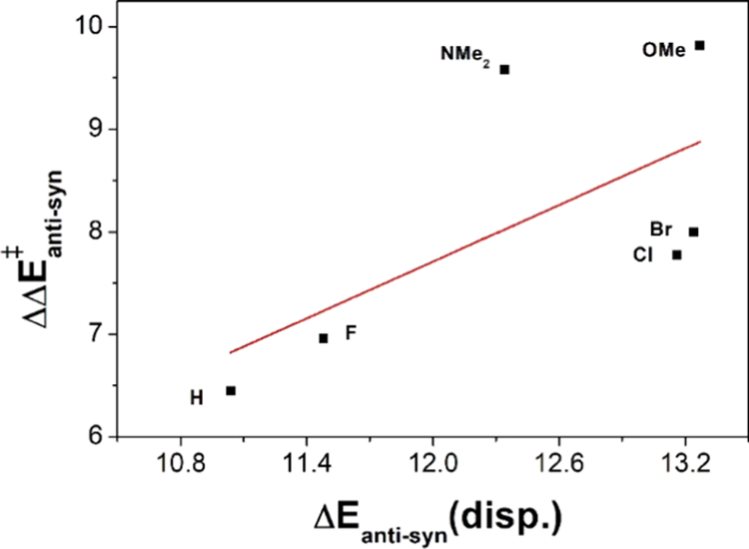
Correlation between the difference in barrier heights (ΔΔ*E*_anti-syn_^‡^) and dispersion energy (Δ*E*(disp)_anti-syn_) calculated with B3LYP-D3(BJ)/def2-TZVPP//PBEh-3c.

To further probe the role and spatial distribution of the NCIs, we employed the reduced density gradient (RDG) method from Johnson et al.,^19^ using the freely available MultiWFN code^20^ with VMD^21^ for visualization via all-electron single point calculations at the B3LYP-ZORA^22^-def2-TZVPP//PBEh-3c level in ORCA 4.2.1.^23^ Examination of the RDG isosurfaces reveals a pronounced increase in favorable NCIs in the region between the phenol and gold-phosphine moieties of the TS_syn-H_ structure, as compared to TS_anti-H_ (Figure 5). This analysis, therefore, further substantiates the origin of the energetic preference for the *syn*-conformation, seen experimentally and computationally across this series of substituents.

**Figure 5:**
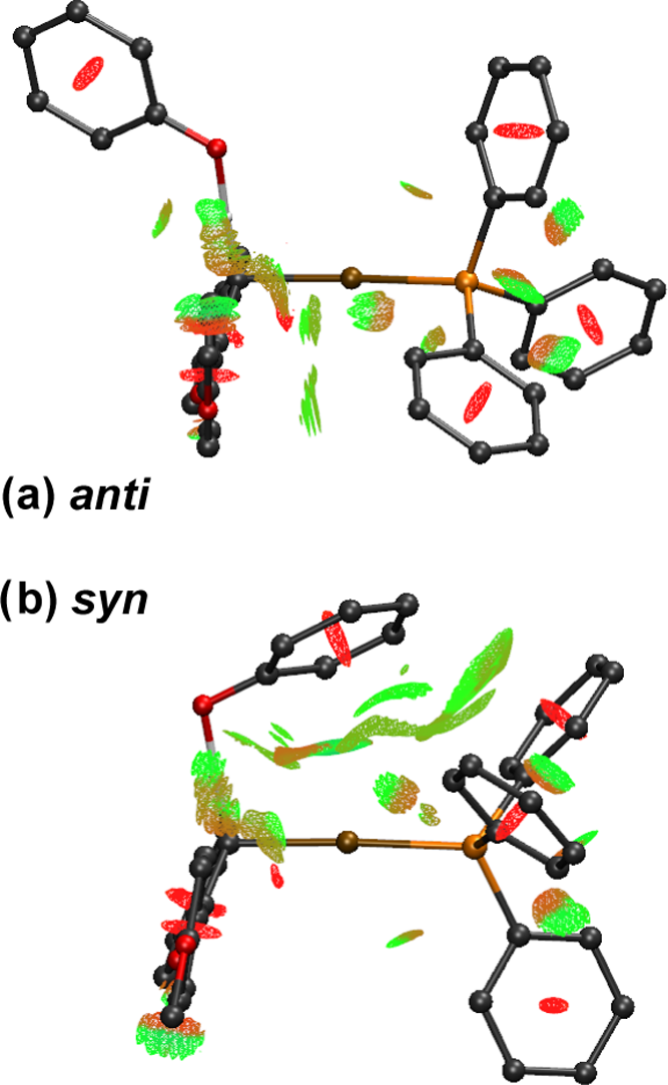
Side view of RDG isosurfaces (*s* = 0.45 au) of (a) TS_anti-H_ and (b) TS_syn-H_, colored with sign (λ_2_)ρ such that green indicates favorable vdW interactions and red steric clashes. Calculated with B3LYP-ZORA-D3(BJ)/def2-TZVPP//PBEh-3c.

The kinetics of the protodeauration step were investigated in detail for a series of isolated vinyl gold(I) complexes employing phenols as proton sources. Our findings shed light on broader questions in gold catalysis, as controlling the reactivity in linear gold(I) complexes is sometimes considered to be challenging due to the 180° bond angle between the metal center and the (often) several Ångstroms of separation between the spectator ligand and the substrate.^24^ Indeed, this argument is often used to motivate work in enantioselective gold(III) catalysis.^25^ In this work, however, we highlight a strategy to fine-tune the reactivity in gold(I) complexes, namely, the modulation of noncovalent interactions (NCIs) between the spectator ligand and the substrate. This was achieved via substitution, which, in our case, resulted in more than three orders of magnitude rate enhancement based on our computational analysis. The substitution on the proton source, or more broadly speaking electrophile, seven bonds away from the gold center, challenges the perception that ligand effects in two-coordinate linear gold(I) complexes are mostly confined to electronic effects and that no special control may be realized. As indicated in the introduction, the key steps in many catalytic cycles for gold-catalyzed transformations of unsaturated compounds involve an initial formation of a vinyl gold intermediate followed by protodeauration. These steps have inherently different requirements with respect to the choice of the (spectator) ligand, where EWGs promote the initial formation of a vinyl gold intermediate, and EDGs promote protodeauration, or, more generally, the reaction with an electrophile. This leads to an inevitable tradeoff when matching requirements in designing efficient catalysts. Our results presented within this work, however, indicate that this tradeoff may, in fact, not be as significant as chemical intuition might make us believe. If the formation of the initial vinyl gold moiety required a more electron-withdrawing ligand, this may, in fact, be compensated through the NCIs identified here. This stems from the fact that the addition of a nucleophile to a gold-activated alkyne occurs opposite to the gold and its (spectator) ligands. Here, NCIs may, in fact, not play such a dominant role. Hence, the significant rate enhancement based on NCIs identified here may, in fact, compensate sufficiently and lead to more efficient catalytic systems.

## Supporting Information Available

Experimental details, characterization data, full computational details, and Cartesian coordinates (PDF)

## References

[ref1] aDyker G. An eldorado for homogeneous catalysis?. Angew. Chem., Int. Ed. 2000, 39, 4237–4239. 10.1002/1521-3773(20001201)39:233.0.co;2-a. 29711922

[ref2] Lu Z. C.; Hammond G. B.; Xu B. Improving Homogeneous Cationic Gold Catalysis through a Mechanism-Based Approach. Acc. Chem. Res. 2019, 52, 1275–1288. 10.1021/acs.accounts.8b00544. 31002231

[ref3] aHashmi A. S. K. Homogeneous gold catalysis beyond assumptions and proposals-characterized intermediates. Angew. Chem., Int. Ed. 2010, 49, 5232–5241. 10.1002/anie.200907078. 20572216

[ref4] aAkana J. A.; Bhattacharyya K. X.; Muller P.; Sadighi J. P. Reversible C-F bond formation and the Au-catalyzed hydrofluorination of alkynes. J. Am. Chem. Soc. 2007, 129, 7736–7737. 10.1021/ja0723784. 17547409

[ref5] aBabaAhmadi R.; Ghanbari P.; Rajabi N. A.; Hashmi A. S. K.; Yates B. F.; Ariafard A. A Theoretical Study on the Protodeauration Step of the Gold(I)-Catalyzed Organic Reactions. Organometallics 2015, 34, 3186–3195. 10.1021/acs.organomet.5b00219.

[ref6] aMarkham J. P.; Staben S. T.; Toste F. D. Gold(I)-catalyzed ring expansion of cyclopropanols and cyclobutanols. J. Am. Chem. Soc. 2005, 127, 9708–9709. 10.1021/ja052831g. 15998074

[ref7] Leyva A.; Corma A. Isolable Gold(I) Complexes Having One Low-Coordinating Ligand as Catalysts for the Selective Hydration of Substituted Alkynes at Room Temperature without Acidic Promoters. J. Org. Chem. 2009, 74, 2067–2074. 10.1021/jo802558e. 19170603

[ref8] Taschinski S.; Dopp R.; Ackermann M.; Rominger F.; de Vries F.; Menger M. F. S. J.; Rudolph M.; Hashmi A. S. K.; Klein J. E. M. N. Light-Induced Mechanistic Divergence in Gold(I) Catalysis: Revisiting the Reactivity of Diazonium Salts. Angew. Chem., Int. Ed. 2019, 58, 16988–16993. 10.1002/anie.201908268. PMC6899485 31552696

[ref9] Grimme S.; Brandenburg J. G.; Bannwarth C.; Hansen A. Consistent structures and interactions by density functional theory with small atomic orbital basis sets. J. Chem. Phys. 2015, 143, 054107 10.1063/1.4927476. 26254642

[ref10] aGrimme S.; Antony J.; Ehrlich S.; Krieg H. A consistent and accurate ab initio parametrization of density functional dispersion correction (DFT-D) for the 94 elements H-Pu. J. Chem. Phys. 2010, 132, 154104 10.1063/1.3382344. 20423165

[ref11] Zhao Y.; Truhlar D. G. Design of density functionals that are broadly accurate for thermochemistry, thermochemical kinetics, and nonbonded interactions. J. Phys. Chem. A 2005, 109, 5656–5667. 10.1021/jp050536c. 16833898

[ref12] Weigend F.; Ahlrichs R. Balanced basis sets of split valence, triple zeta valence and quadruple zeta valence quality for H to Rn: Design and assessment of accuracy. Phys. Chem. Chem. Phys. 2005, 7, 3297–3305. 10.1039/b508541a. 16240044

[ref13] Yang T. H.; Quesne M. G.; Neu H. M.; Reinhard F. G. C.; Goldberg D. P.; de Visser S. P. Singlet versus Triplet Reactivity in an Mn(V)-Oxo Species: Testing Theoretical Predictions Against Experimental Evidence. J. Am. Chem. Soc. 2016, 138, 12375–12386. 10.1021/jacs.6b05027. 27545752 PMC5228574

[ref14] aUyeda C.; Jacobsen E. N. Transition-State Charge Stabilization through Multiple Non-covalent Interactions in the Guanidinium-Catalyzed Enantioselective Claisen Rearrangement. J. Am. Chem. Soc. 2011, 133, 5062–5075. 10.1021/ja110842s. 21391614 PMC3070243

[ref15] aNeel A. J.; Hilton M. J.; Sigman M. S.; Toste F. D. Exploiting non-covalent π interactions for catalyst design. Nature 2017, 543, 637–646. 10.1038/nature21701. 28358089 PMC5907483

[ref16] aBecke A. D. Density-functional exchange-energy approximation with correct asymptotic behavior. Phys. Rev. A 1988, 38, 3098–3100. 10.1103/PhysRevA.38.3098. 9900728

[ref17] Kruse H.; Goerigk L.; Grimme S. Why the Standard B3LYP/6-31G* Model Chemistry Should Not Be Used in DFT Calculations of Molecular Thermochemistry: Understanding and Correcting the Problem. J. Org. Chem. 2012, 77, 10824–10834. 10.1021/jo302156p. 23153035

[ref18] a This can be seen further in the parametrization of the D3(BJ) model by Gimme and co-workers:;

[ref19] Johnson E. R.; Keinan S.; Mori-Sánchez P.; Contreras-García J.; Cohen A. J.; Yang W. Revealing Noncovalent Interactions. J. Am. Chem. Soc. 2010, 132, 6498–6506. 10.1021/ja100936w. 20394428 PMC2864795

[ref20] Lu T.; Chen F. Multiwfn: A multifunctional wavefunction analyzer. J. Comput. Chem. 2012, 33, 580–592. 10.1002/jcc.22885. 22162017

[ref21] Humphrey W.; Dalke A.; Schulten K. VMD: Visual molecular dynamics. J. Mol. Graphics 1996, 14, 33–38. 10.1016/0263-7855(96)00018-5. 8744570

[ref22] avan Lenthe E.; Ehlers A.; Baerends E.-J. Geometry optimizations in the zero order regular approximation for relativistic effects. J. Chem. Phys. 1999, 110, 8943–8953. 10.1063/1.478813.

[ref23] aNeese F. The ORCA program system. WIREs Comput. Mol. Sci. 2012, 2, 73–78. 10.1002/wcms.81.

[ref24] aCera G.; Bandini M. Enantioselective Gold(I) Catalysis with Chiral Monodentate Ligands. Isr. J. Chem. 2013, 53, 848–855. 10.1002/ijch.201300029.

[ref25] aBohan P. T.; Toste F. D. Well-Defined Chiral Gold(III) Complex Catalyzed Direct Enantioconvergent Kinetic Resolution of 1,5-Enynes. J. Am. Chem. Soc. 2017, 139, 11016–11019. 10.1021/jacs.7b06025. 28771334 PMC5911161

