# Supplementary material for: On the Role of Noncovalent Ligand-Substrate Interactions in Au(I) Catalysis: An Experimental and Computational Study of Protodeauration
Source: ACS Catal. 2022 Oct 13;12(21):13158–63. doi: 10.1021/acscatal.2c03384 (PMC9638986; doi:10.1021/acscatal.2c03384)
Supplement: Supplementary file 1 — cs2c03384_si_001.pdf [file cs2c03384_si_001.pdf]

On the Role of Noncovalent Ligand-Substrate Interactions in Au(I) Catalysis: An Experimental and Computational Study of Protodeauration

Taegeun Jo,[a] Svenja Taschinski,[a,b] Isaac F. Leach,[a] Christina Bauer,[a,b] A. Stephen K. Hashmi,[b,*] and Johannes E. M. N. Klein[a,*]

[a]*Molecular Inorganic Chemistry, Stratingh Institute for Chemistry, Faculty of Science and Engineering, University of Groningen, Nijenborgh 4, 9747 AG Groningen (The Netherlands). Correspondence to: j.e.m.n.klein@rug.nl*

[b]*Organisch-Chemisches Institut, Heidelberg University, Im Neuenheimer Feld 270, 69120 Heidelberg (Germany). Correspondence to: hashmi@hashmi.de*

Table of Contents:

**1. General Remarks**	**S2**
**2. Synthesis and characterization of 3_R and 1_R**	**S3-13**
**3. Kinetic studies**	**S14-26**
**4. Computational details**	**S27-33**
**5. Cartesian Coordinates**	**S33-106**
**6. References**	**S107**

1. General Remarks

Note

The experimental data provided below originates in part from Chapter 3 of the Ph.D. thesis of S. Taschinski (2020).[1]

Chemicals and Solvents

Chemicals and solvents were purchased from commercial suppliers (ABCR, Acros, Alfa Aesar, Chempur, Fluka, Fluorochem, Merck, Euriso-Top, BOOM and Sigma Aldrich) or obtained from the chemical store at the University of Heidelberg and the University of Groningen and used as delivered. Dry solvents were dispensed from a solvent purification system MB SPS-800 or obtained by using drying columns.[2]

Reactions

Reactions requiring inert conditions were carried out in heat-gun dried glassware under an atmosphere of nitrogen using standard Schlenk-techniques. For some reactions degassed solvents were used by sparging them with nitrogen for at least one hour.

NMR Spectroscopy (NMR)

NMR spectra were, if not mentioned otherwise, recorded at room temperature at the chemistry department of the University of Heidelberg on the following spectrometers: Bruker Avance-III-300, Bruker Avance DRX-300, Bruker Avance-III-500 and Bruker Avance-III-600 and at the University of Groningen on the following spectrometers: Varian Oxford 300, AgilentTech 400/54 Premium Shielded, Varian Oxford 500, Bruker Avance-Neo 600. Chemical shifts are given in ppm and coupling constants in Hz. ^{1}H and ^{13}C spectra were calibrated in relation to deuterated solvents, namely $CDCl_3$ (7.26 ppm; 77.16 ppm). The following abbreviations were used for ^{1}H NMR spectra to indicate the signal multiplicity: s (singlet), bs (broad singlet), d (doublet), t (triplet), q (quartet) and m (multiplet) as well as combinations of them. When combinations of multiplicities are given the first character noted refers to the biggest coupling constant. All ^{13}C NMR spectra were measured with ^{1}H decoupling. The nature of the observed C atoms in these spectra is indicated as follows: s (quaternary carbon), d (CH group), t (CH_2 group), q (CH_3 group) and were determined by DEPT135 spectra.

Mass Spectrometry (MS and HR MS)

Mass spectra were determined at the MS department of the University of Heidelberg and the Microanalytical Department of the University of Groningen.

Infrared Spectroscopy (IR)

Infrared spectra were recorded on an FT IR spectrometer named Bruker LUMOS, Germanium ATR-Kristall (Heidelberg) and JASCO FT/IR-4700 (Groningen). The method is denoted in brackets. For the most significant bands the wave number $\tilde{\nu}$ (cm^{-1}) is given.

Melting Points

Melting points were measured in open glass capillaries in a Büchi melting point apparatus and were not corrected.

Flash Column Chromatography

Flash column chromatography was accomplished using Silica gel 60 (0.04 - 0.063 mm/ 230 - 400 mesh ASTM) purchased from Macherey-Nagel, SiliaFlash® P60 (0.04 - 0.063 mm/ 230 - 400 mesh) purchased from Silicycle UltraPure Silica gels and aluminium oxide (activated, neutral, Brockmann Activity I) from Fluka. As eluents mixtures of petroleum ether (PE) and ethyl acetate (EA) or diethyl ether (Et_2O) were used.

2. Synthesis and characterization of 3_R and 1_R

The following compounds were prepared using literature known procedures.

	Compound	Ref. Procedure
**3**	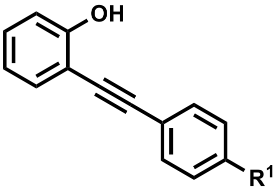 **R^1= H, Me, OMe, F, CF_3**	3
**1**	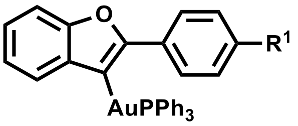 **R^1= H, Me, OMe, F, CF_3**	4

2.1 Synthesis of compound 3

A series of **3** (R= H, Me, OMe, F, CF_3) was synthesized according to a modified procedure previously reported by Wegner and co-workers.[3]

2-Iodophenol (2.00 g, 9.09 mmol, 1.00 eq) was dissolved in THF, bis(triphenylphosphine)palladium(II) dichloride (160 mg, 227 µmol, 2.50 mol %), copper iodide (86.6 mg, 455 µmol, 5.00 mol-%), diisopropylamine (1.28 mL, 920 mg, 9.09 mmol, 1.00 eq) and 1-ethynyl-4-(trifluoromethyl)benzene (1.63 mL, 1.70 g, 10.0 mmol, 1.10 eq) were added and the mixture was stirred at room temperature for 24 hours, worked up and purified by flash column chromatography. Due to not full conversion of the starting material, the procedure was repeated two times and purification by flash column chromatography (SiO2, PE/EA, 20:1) yielded the product $\mathbf{3_{CF3}}$ (120 mg, 459 µmol, 5%) as a light brown, crystalline solid. R_f (SiO_2, PE/EA, 10:1) = 0.50; ^{1}H NMR (400 MHz, $CDCl_3$): δ = 5.74 (s, 1 H), 6.94 (td, J = 7.53 Hz, J = 1.12 Hz, 1 H), 7.00 (dd, J = 8.27 Hz, J = 1.09 Hz, 1 H), 7.31 (ddd, J = 8.78 Hz, J = 7.30 Hz, J = 1.66 Hz, 1 H), 7.44 (dd, J = 7.70 Hz, J = 1.66 Hz, 1 H), 7.64 (s, 4 H) ppm; ^{19}F NMR (376 MHz, CDCl3): δ = -62.90 (s, 3 F) ppm. The spectroscopic data matches previously reported data.[5]

The detail of synthesis and characterization of $\mathbf{3_H}$, $\mathbf{3_{Me}}$, $\mathbf{3_{OMe}}$ and $\mathbf{3_F}$ was described in our recently published study.[6]

2.2 Synthesis of 1_R

A series of **1** (R= H, Me, OMe, F, CF_3) were synthesized according to a modified procedure previously reported by Hashmi and co-workers.[4] The detail of synthesis and characterization of $\mathbf{1_{Me}}$ was described in our recently published study.[6]

In a heat-gun dried Schlenk tube, Ph_3PAuCl (1.00 eq) and AgOTs (1.00 eq) were dissolved in 50.0 mL THF and stirred for one hour at room temperature under exclusion of light. Triethylamine (7.00 eq) and substituted *p*-(phenylethynyl)phenol (1.02/1.05 eq) were added and the mixture was stirred for the mentioned time at room temperature. The mixture was filtered through a pad of neutral Al_2O_3 and evaporated at room temperature. The crude solids were recrystallized from dichloromethane/pentane, filtered and washed with pentane to give the products **1**.

2.2.1 (2-Phenylbenzofuran-3-yl)(triphenyl-λ5-phosphanyl) gold(I), 1_H

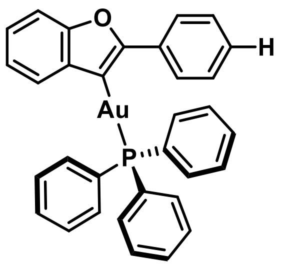

$\mathbf{1_H}$

Ph_3PAuCl (146 mg, 294 µmol, 1.00 eq) and AgOTs (82.1 mg, 294 µmol, 1.00 eq) were dissolved in 50.0 mL THF and stirred for one hour at room temperature. Triethylamine (297 µL, 208 mg, 2.08 mmol, 7.00 eq) and 2-(phenylethynyl)phenol (58.3 mg, 300 µmol, 1.02 eq) were added and the mixture was stirred for 46 hours. Recrystallization yielded the product $\mathbf{1_H}$, (155 mg, 284 µmol, 81%) as a light yellow, crystalline solid. M.P.: decomp > 179 °C; IR (ATR): $\tilde{\nu}$ = 634, 666, 688, 710, 736, 749, 765, 812, 847, 886, 915, 970, 998, 1008, 1017, 1028, 1039, 1069, 1100, 1142, 1157, 1181, 1199, 1251, 1308, 1330, 1388, 1434, 1448, 1465, 1479, 3014, 3032, 3047, 3066 cm^{-1}; ^{1}H NMR (600 MHz, CD_2Cl_2): δ = 7.14 (dd, *J* = 7.38 Hz, *J* = 1.09 Hz, 1 H), 7.19 (dd, *J* = 7.59 Hz, *J* = 1.41 Hz, 1 H), 7.25-7.30 (m, 1 H), 7.31-7.35 (m, 2 H), 7.49 (d, *J* = 8.27 Hz, 1 H), 7.50-7.61 (m, 9 H), 7.65-7.70 (m, 6 H), 7.78-7.80 (m, 1H), 8.40 (dt, *J* = 8.40 Hz, *J* = 1.32 Hz, 2 H) ppm; ^{13}C NMR (151 MHz, CD_2Cl_2): δ = 110.65 (d), 121.83 (d), 123.74 (d), 124.95 (d), 125.71 (d, 2 C), 127.57 (d), 128.54 (d, 2 C), 129.56 (d, 3 C), 129.64 (d, 3 C), 130.88 (s), 131.23 (s), 131.83 (d, d, *J* = 2.43 Hz, 3 C), 134.48 (s), 134.70 (d, 3 C), 134.79 (d, 3 C), 139.63 (s, 3 C), 155.49 (s), 162.21 (s) ppm; ^{31}P NMR (243 MHz, CD_2Cl_2): δ = 45.18 (s) ppm; HR MS (DART(+)) $C_{32}H_{25}AuOP$ $[M+H]^+$: calcd. 653.1303, found 653.1302.

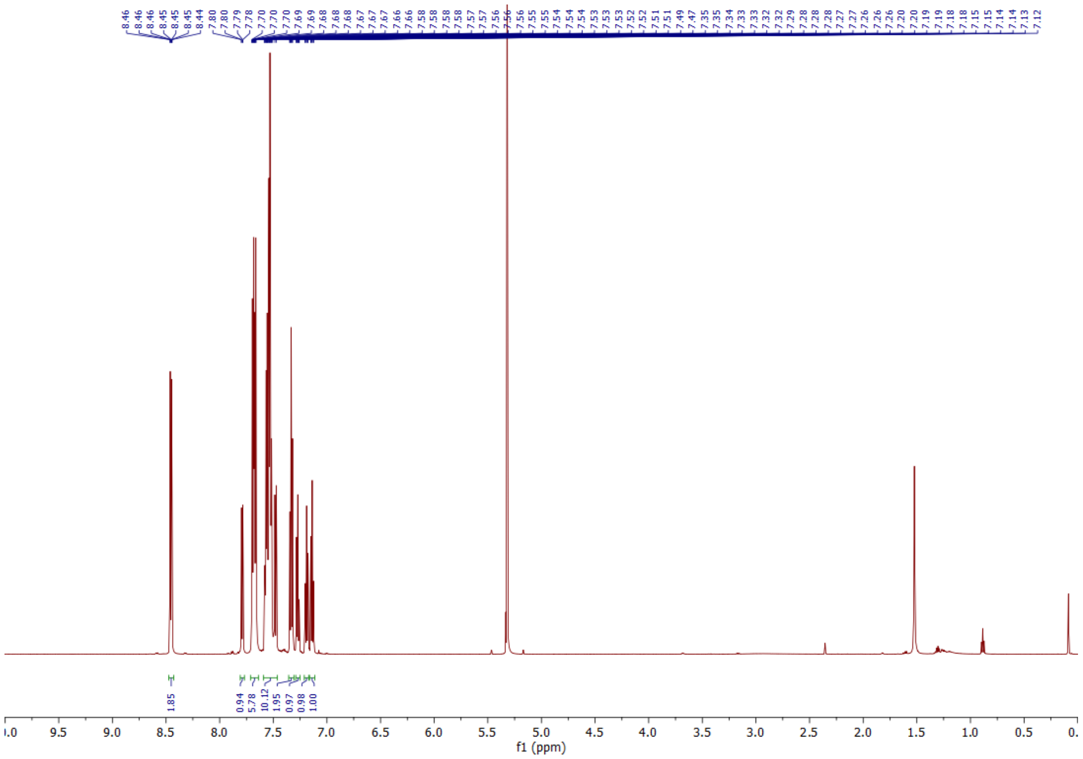

Figure S1. ^{1}H NMR of $\mathbf{1_H}$ ((2-Phenylbenzofuran-3-yl)(triphenyl-λ^5-phosphanyl) gold(I)).

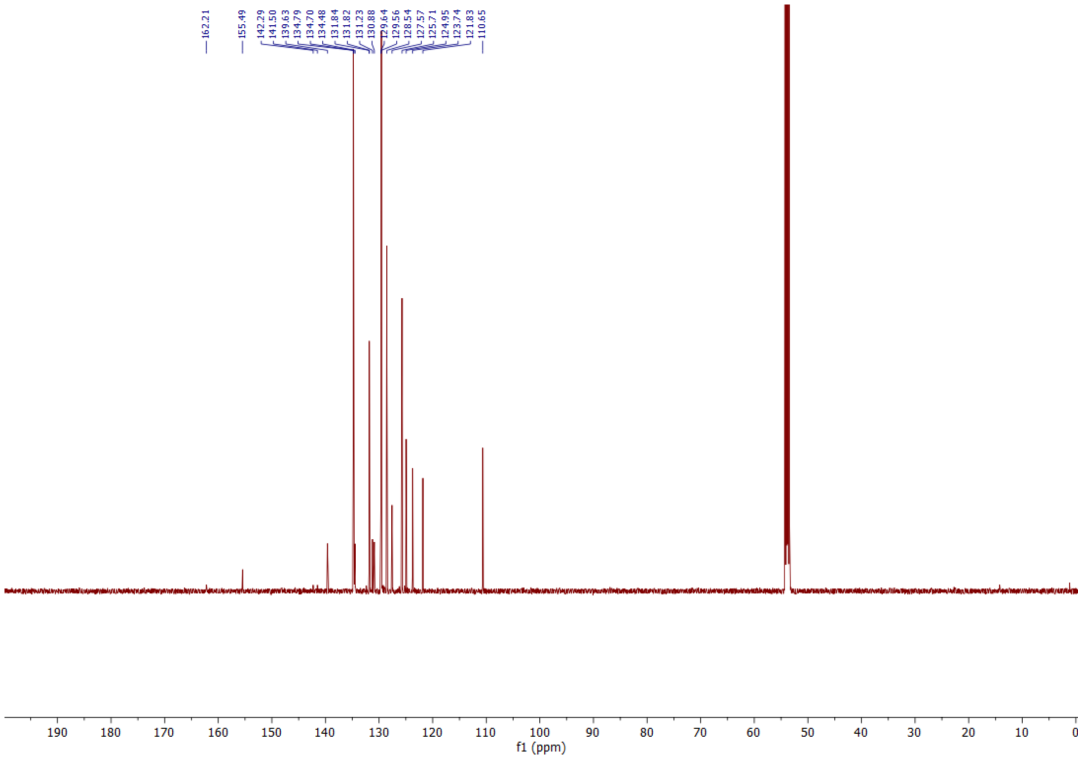

Figure S2. ^{13}C NMR of $\mathbf{1_H}$ ((2-Phenylbenzofuran-3-yl)(triphenyl-λ^5-phosphanyl) gold(I)).

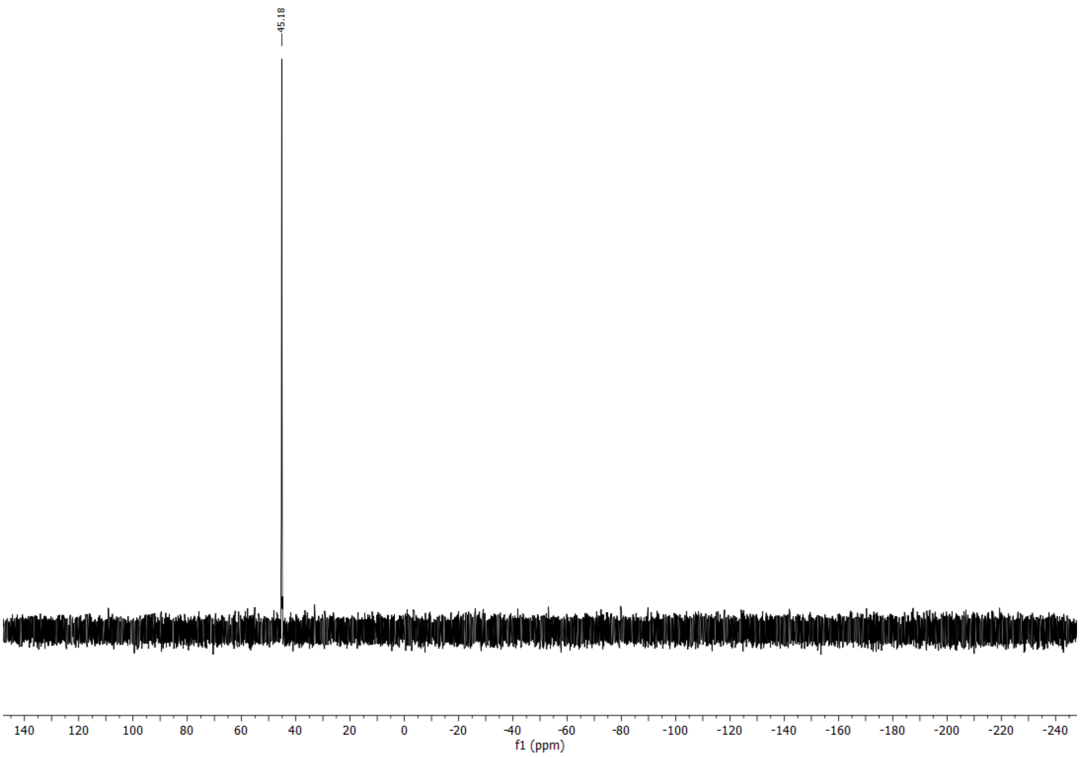

Figure S3. ^{31}P NMR of $\mathbf{1_H}$ ((2-Phenylbenzofuran-3-yl)(triphenyl-λ^5-phosphanyl) gold(I)).

2.2.2 (2-(4-Methoxyphenyl)benzofuran-3-yl) (triphenyl- λ^5-phosphanyl) gold(I), $\mathbf{1_{OMe}}$

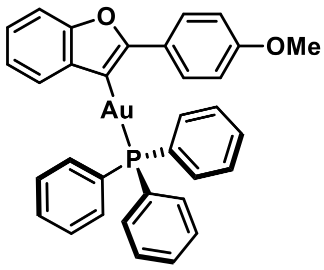

1_{OMe}

Ph_3APuCl (146 mg, 294 µmol, 1.00 eq) and AgOTs (82.1 mg, 294 µmol, 1.00 eq) were dissolved in 50.0 mL THF and stirred for one hour at room temperature. Triethylamine (297 µL, 208 mg, 2.08 mmol, 7.00 eq) and 2-((4-methoxyphenyl)-ethynyl)phenol (67.3 mg, 300 µmol, 1.02 eq) were added and the mixture was stirred for 18 hours. Recrystallization yielded the product $\mathbf{1_{OMe}}$ (155 mg, 277 µmol, 77%) as a colorless, crystalline solid. M.P.: decomp > 189 °C; IR (ATR): $\tilde{\nu}$ = 633, 643, 694, 709, 741, 779, 808, 832, 886, 925, 969, 998, 1009, 1026, 1046, 1070, 1101, 1172, 1242, 1284, 1302, 1331, 1414, 1434, 1448, 1464, 1490, 1538, 1581, 1609, 2837, 2939, 2991, 3048 cm^{-1}; ^1H NMR (600 MHz, CD_2Cl_2): δ = 3.83 (s, 3 H), 6.83-6.90 (m, 2 H), 7.12 (t, *J* = 7.21 Hz, 1 H), 7.13-7.18 (m, 1 H), 7.45 (d, *J* = 7.85 Hz, 1 H), 7.47-7.60 (m, 9 H), 7.63-7.71 (m, 6 H), 7.76 (d, *J* = 7.39 Hz, 1 H), 8.42-8.27 (m, 2 H) ppm; ^{13}C NMR (151 MHz, CD_2Cl_2): δ = 55.65 (q), 110.46 (d), 113.92 (d, 2 C), 121.75 (d), 123.23 (d), 124.75 (d), 127.12 (d, 2 C), 127.37 (s), 129.55 (d, 3 C), 129.63 (d, 3 C), 131.12 (s, d, *J* = 51.8 Hz), 131.81 (d, d, *J* = 2.33 Hz, 3 C), 134.70 (d, 3 C), 134.79 (d, 3 C), 139.81 (s, d, *J* = 2.51 Hz, 3 C), 139.98 (s, d, *J*= 118 Hz), 155.31 (s, d, *J* = 4.96 Hz), 159.61 (s), 162.25 (s, d, *J* = 10.32 Hz) ppm; ^{31}P NMR (243 MHz, CD_2Cl_2): δ = 45.30 (s) ppm; HR MS (DART(+)) $C_{33}H_{27}AuO_2P$ $[M+H]^+$: calcd. 683.1409, found 683.1394.

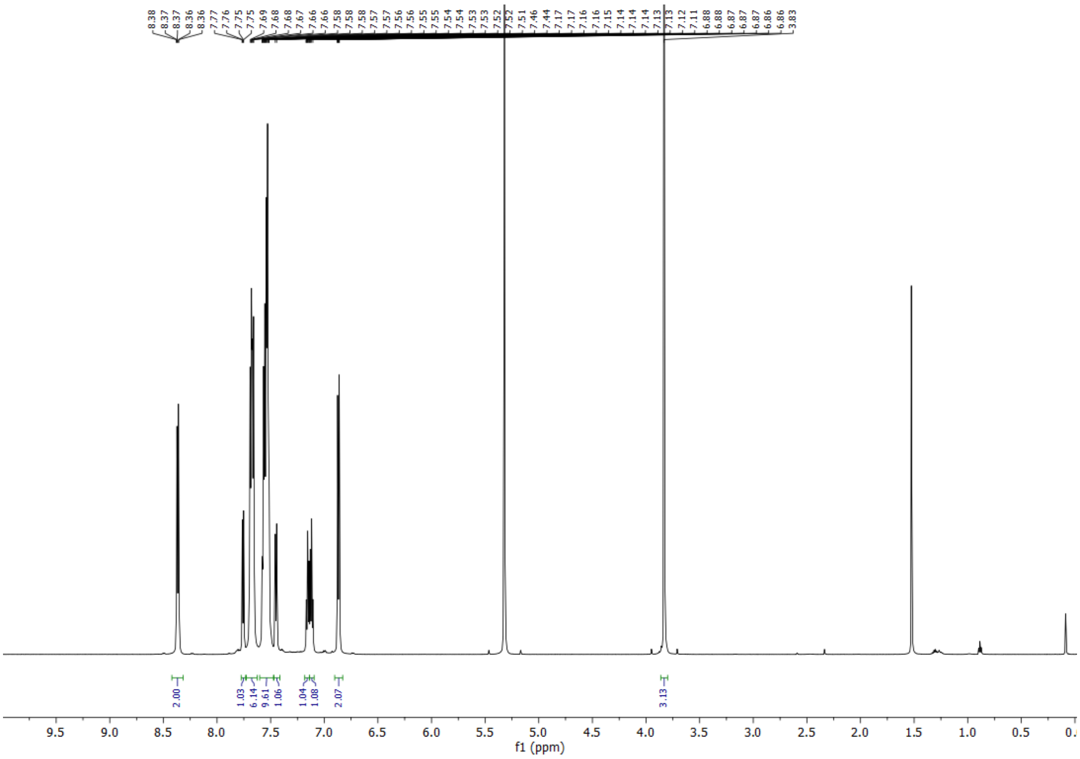

Figure S4. ^{1}H NMR of **1$_{\mathbf{OMe}}$** ((2-(4-Methoxyphenyl)benzofuran-3-yl) (triphenyl- λ^{5}-phosphanyl) gold(I)).

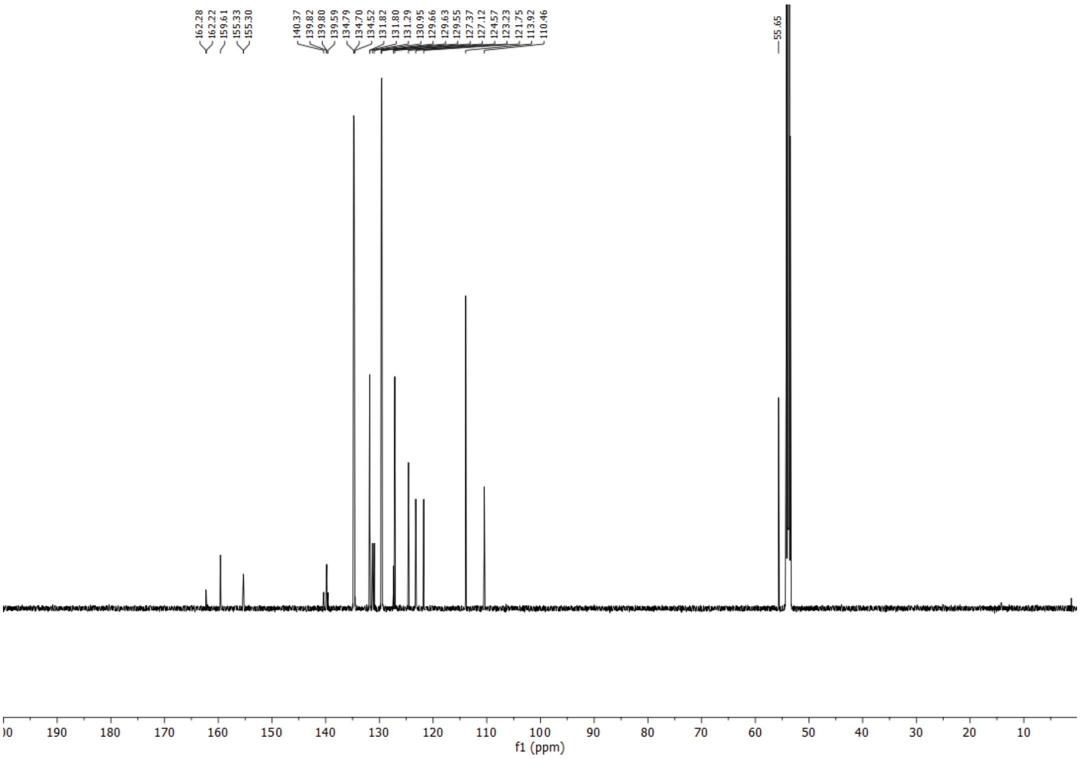

Figure S5. ^{13}C NMR of **1$_{\mathbf{OMe}}$** ((2-(4-Methoxyphenyl)benzofuran-3-yl) (triphenyl- λ^{5}-phosphanyl) gold(I)).

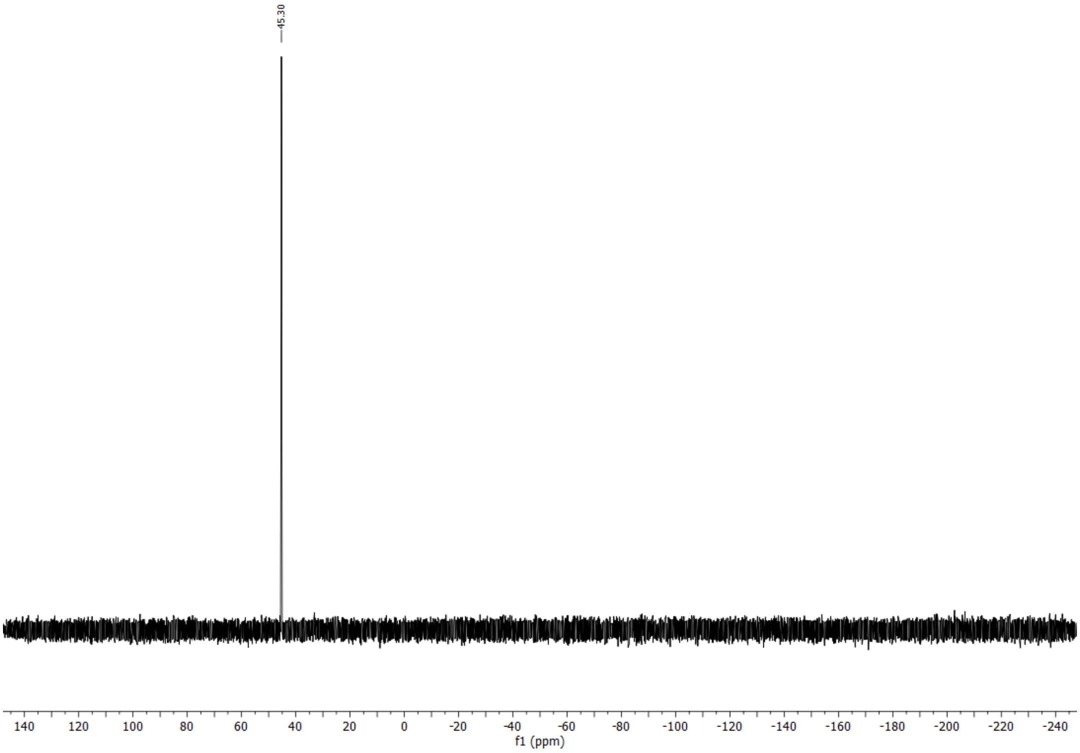

Figure S6. ^{31}P NMR of **1$_{OMe}$** ((2-(4-Methoxyphenyl)benzofuran-3-yl) (triphenyl- λ^5-phosphanyl) gold(I)).

2.2.3 (2-(4-Fluorophenyl)benzofuran-3-yl)(triphenyl- λ^5-phosphanyl) gold(I), 1$_F$

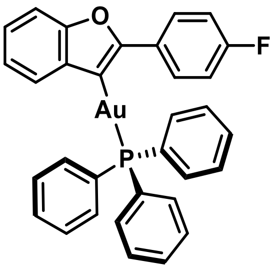

1$_F$

Ph_3PAuCl (146 mg, 294 µmol, 1.00 eq) and AgOTs (82.1 mg, 294 µmol, 1.00 eq) were dissolved in 50.0 mL THF and stirred for 50 minutes at room temperature. Triethylamine (297 µL, 208 mg, 2.08 mmol, 7.00 eq) and 2-((4-fluorophenyl)ethynyl)phenol (63.7 mg, 300 µmol, 1.02 eq) were added and the mixture was stirred for 32 hours. Recrystallization yielded the product **1$_F$** (161 mg, 240 µmol, 82%) as a colorless, crystalline solid. M.P.: decomp > 185 °C; IR (ATR): $\tilde{\nu}$ = 630, 665, 693, 710, 743, 791, 819, 839, 887, 922, 969, 997, 1011, 1026, 1070, 1100, 1154, 1189, 1217, 1251, 1282, 1310, 1332, 1434, 1447, 1488, 1541, 1588, 1773, 1895, 3048 cm^{-1}; ^{1}H NMR (600 MHz, CD_2Cl_2): δ = 6.88-7.04 (m, 2 H), 7.13 (t, *J* = 7.34 Hz, 1 H), 7.16-7.20 (m, 1 H), 7.47 (d, *J* = 7.96 Hz, 1 H), 7.50-7.59 (m, 9 H), 7.63-7.70 (m, 6 H), 7.78 (d, *J* = 7.57 Hz, 1 H), 8.40-8.44 (m, 2 H) ppm; ^{13}C NMR (151 MHz, CD_2Cl_2): δ = 110.64 (d), 115.32 (d, d, *J* = 21.5 Hz, 2 C), 121.91 (d), 123.74 (d), 124.91 (d), 127.44 (d, d, *J* = 7.83 Hz, 2 C), 129.59 (d, 3 C), 129.67 (d, 3 C), 130.81 (s, 2 C), 130.92 (s, d, *J* = 3.26 Hz), 131.16 (s, 2 C), 131.88 (d, d, *J* = 2.37 Hz, 3 C), 134.69 (d, 3 C), 134.78 (d, 3 C), 139.58 (s, 2 C), 155.48 (s), 162.62 (s, d, *J* = 245.9 Hz) ppm; ^{19}F NMR (377 MHz, CD_2Cl_2): δ = -115.82 (s) ppm; ^{31}P NMR (242.92 MHz, CD_2Cl_2): δ = 45.20 (s) ppm; HR MS (DART(+)) $C_{32}H_{23}AuFOP$ $[M]^+$: calcd. 670.1131, found 670.1148.

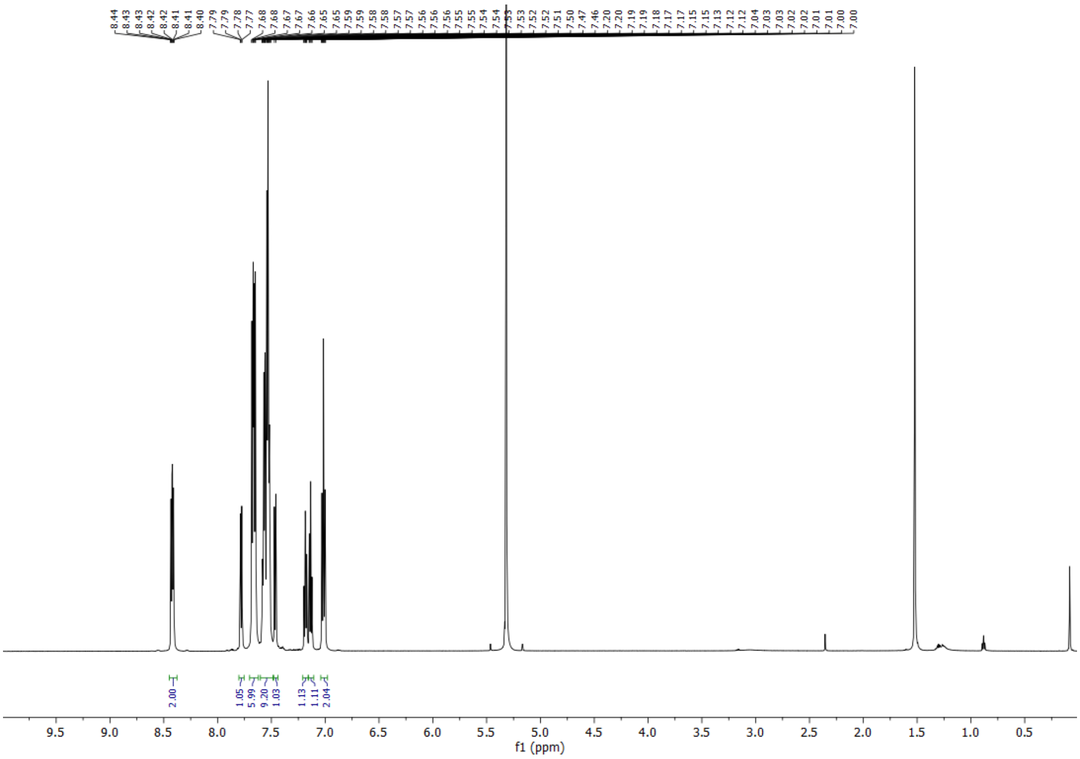

Figure S7. ^{1}H NMR of $\mathbf{1_F}$ ((2-(4-Fluorophenyl)benzofuran-3-yl)(triphenyl- λ5-phosphanyl) gold(I)).

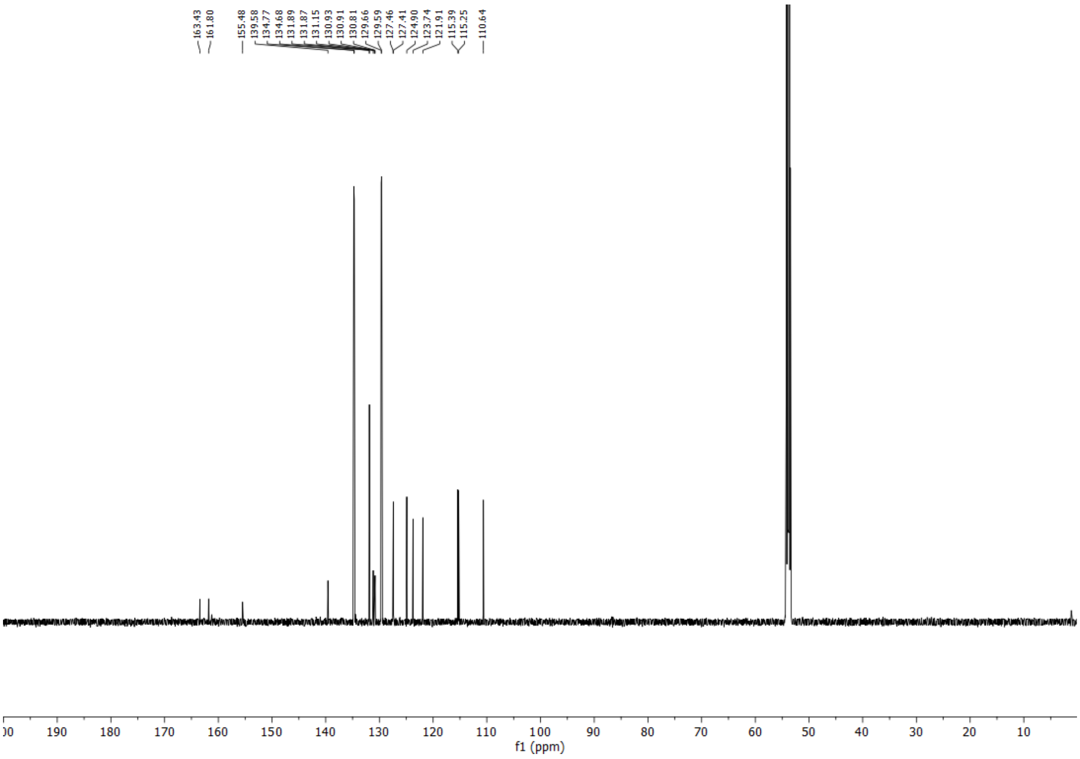

Figure S8. ^{13}C NMR of $\mathbf{1_F}$ ((2-(4-Fluorophenyl)benzofuran-3-yl)(triphenyl- λ5-phosphanyl) gold(I)).

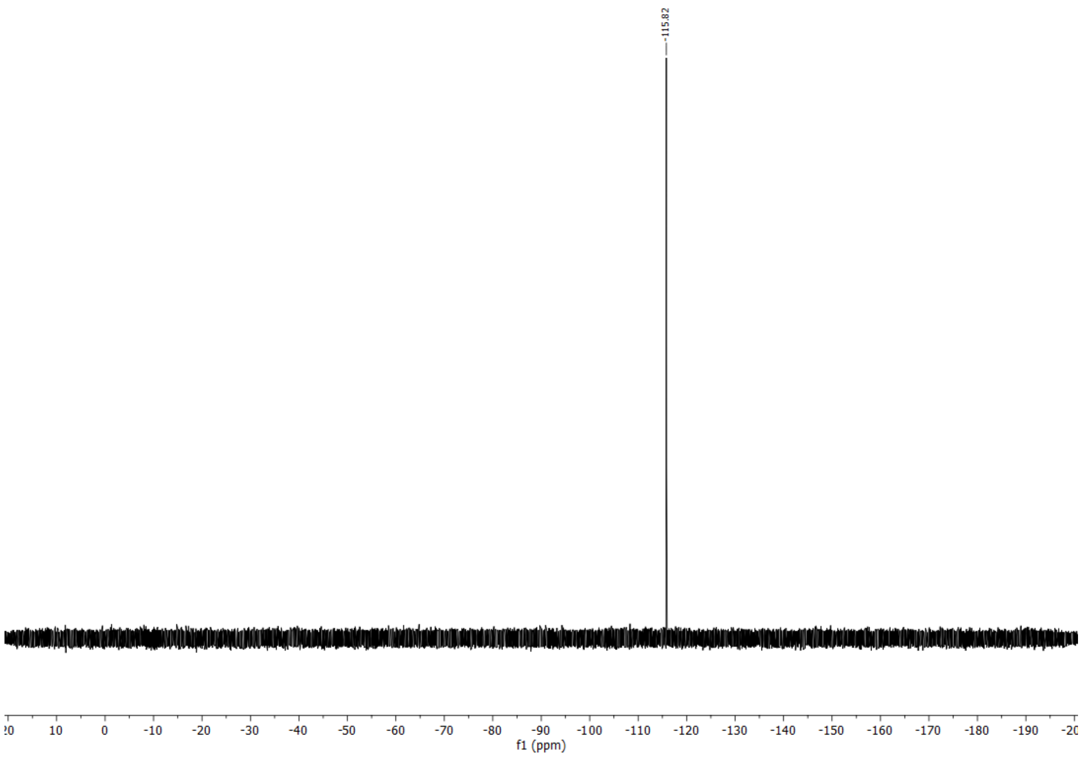

Figure S9. ^{19}F NMR of $\mathbf{1_F}$ ((2-(4-Fluorophenyl)benzofuran-3-yl)(triphenyl- λ5-phosphanyl) gold(I)).

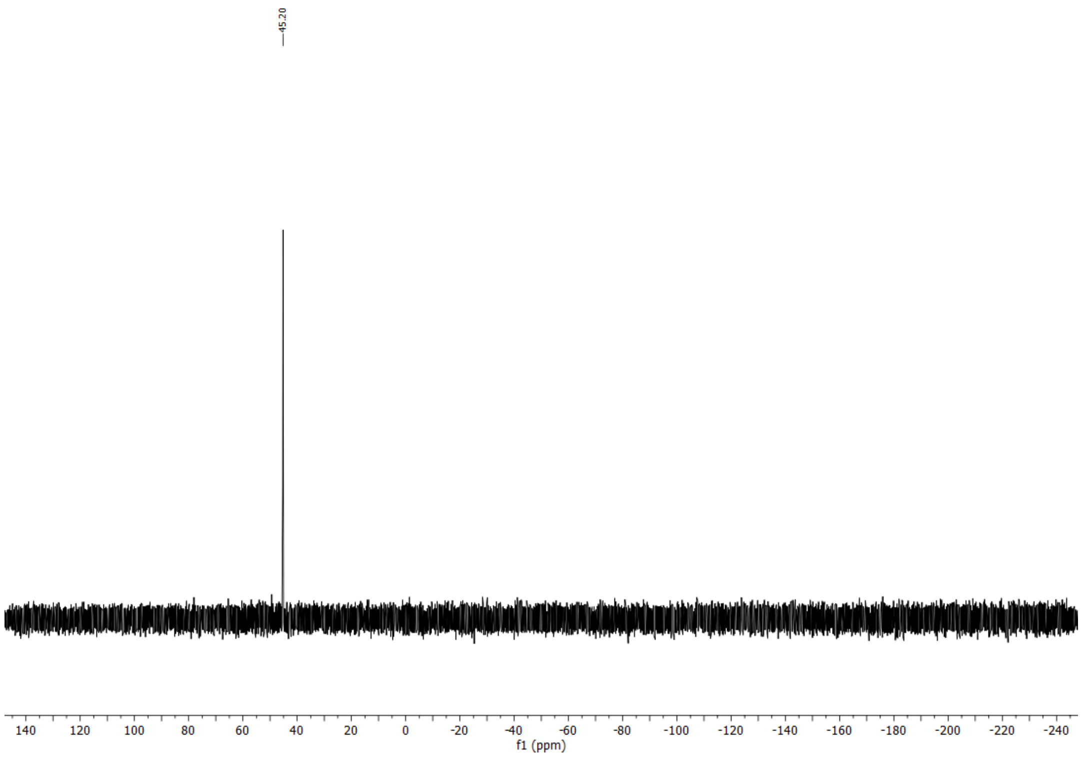

Figure S10. ^{31}P NMR of $\mathbf{1_F}$ ((2-(4-Fluorophenyl)benzofuran-3-yl)(triphenyl- λ5-phosphanyl) gold(I)).

2.2.4 (2-(4-(Trifluoromethyl)phenyl)benzofuran-3-yl)(triphenyl- λ^5-phosphanyl) gold(I), 1_{CF3}

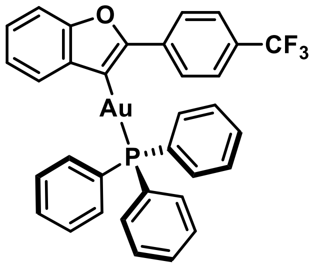

1_{CF3}

Ph_3PAuCl (146 mg, 294 µmol, 1.00 eq) and AgOTs (82.1 mg, 294 µmol, 1.00 eq) were dissolved in 50.0 mL THF and stirred for 50 minutes at room temperature. Triethylamine (297 µL, 208 mg, 2.08 mmol, 7.00 eq) and 2-((4-(trifluoromethyl)-phenyl)ethynyl)phenol **1_{CF3}** (78.7 mg, 300 µmol, 1.02 eq) were added and the mixture was stirred for 69 hours. Recrystallization yielded the product **1_{CF3}** (152 mg, 211 µmol, 72%) as a colorless, crystalline solid. M.P.: decomp > 214 °C; IR (ATR): $\tilde{\nu}$ = 661, 691, 710, 742, 824, 839, 888, 973, 998, 1013, 1027, 1038, 1068, 1101, 1123, 1153, 1182, 1193, 1257, 1321, 1409, 1435, 1447, 1480, 1612, 3048 cm^{-1}; ^1H NMR (600 MHz, CD_2Cl_2): δ = 7.17 (td, *J* = 7.38 Hz, *J* = 1.03 Hz, 1 H), 7.22-7.26 (m, 1 H), 7.48-7.52 (m, 1 H), 7.52-7.60 (m, 11 H), 7.64-7.71 (m, 6 H), 8.82 (dd, *J* = 7.64 Hz, *J* = 1.33 Hz, 1 H), 8.57-8.60 (m, 2 H) ppm; ^{13}C NMR (151 MHz, CD_2Cl_2): δ = 110.88 (d), 122.09 (d), 124.57 (d), 125.18 (s, d, *J* = 272 Hz), 125.45 (d, 2 C), 125.48 (d), 125.51 (s, d, *J* = 3.88 Hz), 125.62 (d, 2 C), 128.53 (s), 128.75 (s), 129.64 (d, 3 C), 129.71 (d, 3 C), 130.91 (s, d, *J* = 52.6 Hz), 131.95 (d, d, *J* = 2.49 Hz, 3 C), 134.69 (d, 3 C), 134.78 (d, 3 C), 138.00 (s), 139.36 (s, d, *J* = 3.02 Hz), 144.67 (s, d, *J* = 116.2 Hz), 155.72 (s, d, *J* = 4.53 Hz), 161 (s, d, *J* = 10.6 Hz) ppm; ^{19}F NMR (565 MHz, CD_2Cl_2): δ = -62.63 (s, 3 F) ppm; ^{31}P NMR (243 MHz, CD_2Cl_2): δ = 45.02 (s) ppm; HR MS (DART(+)) $C_{33}H_{24}AuF_3OP$ $[M+H]^+$: calcd. 721.1476, found 721.1177.

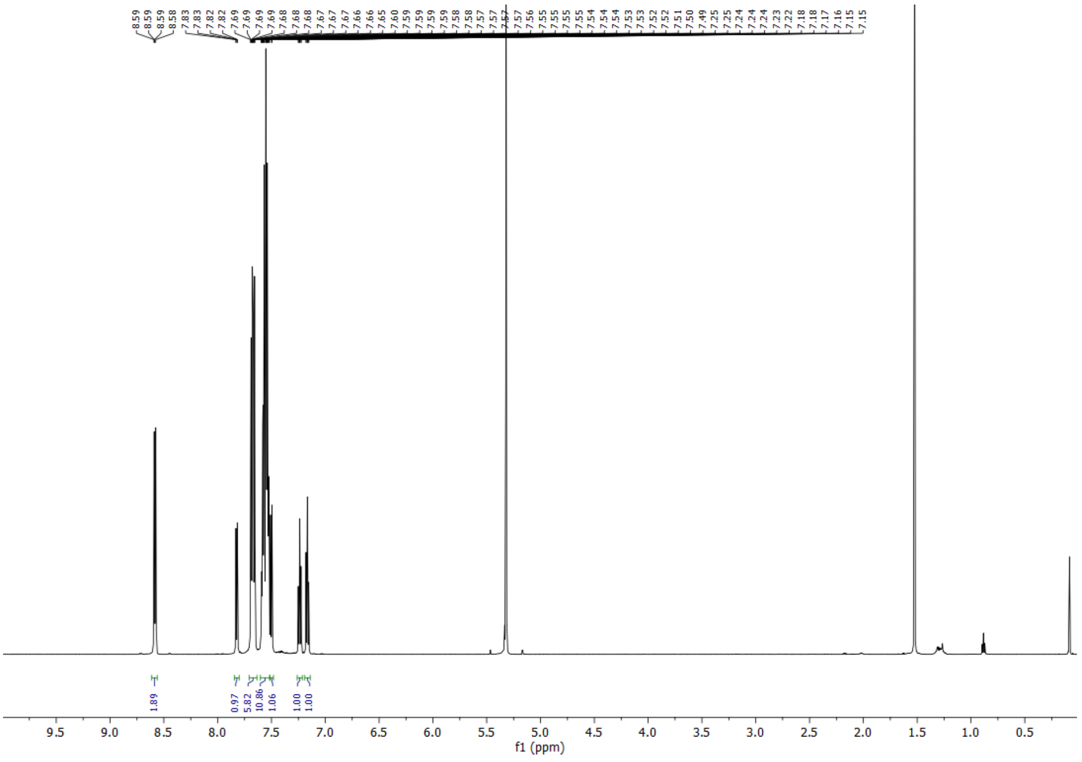

Figure S11. ^1H NMR of **1_{CF3}** ((2-(4-(Trifluoromethyl)phenyl)benzofuran-3-yl)(triphenyl- λ5-phosphanyl) gold(I)).

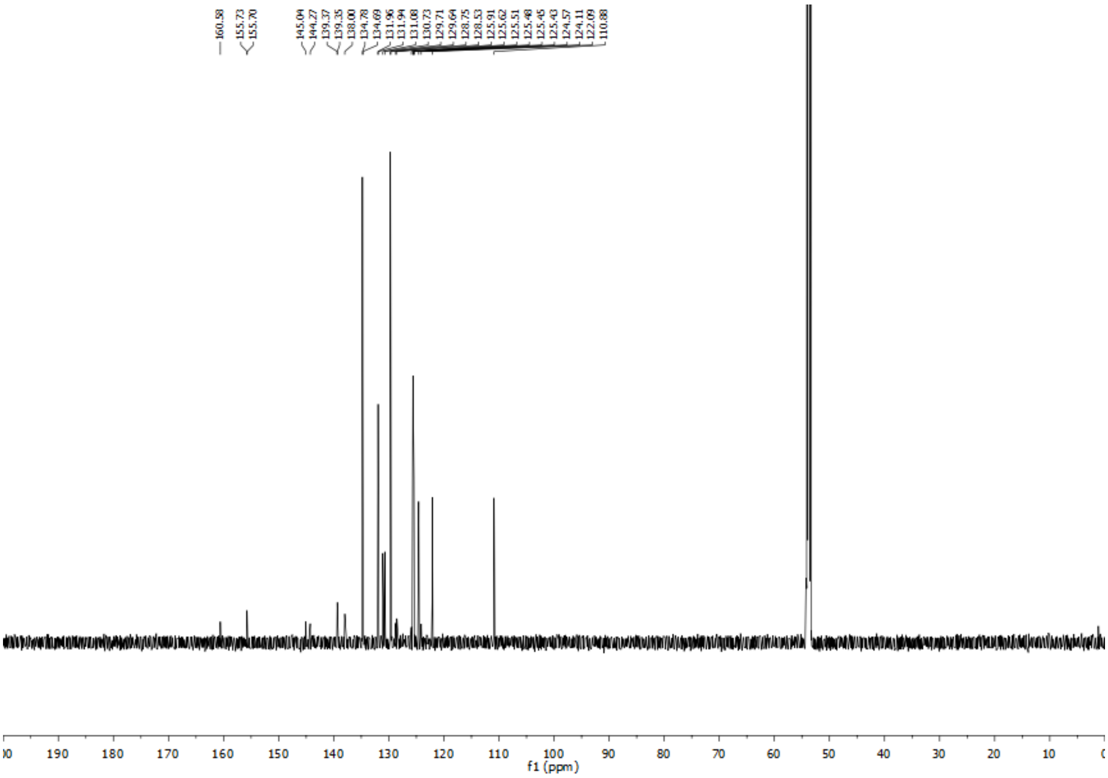

Figure S12. ^{13}C NMR of $\mathbf{1}_{\mathbf{CF3}}$ ((2-(4-(Trifluoromethyl)phenyl)benzofuran-3-yl)(triphenyl- λ5-phosphanyl) gold(I)).

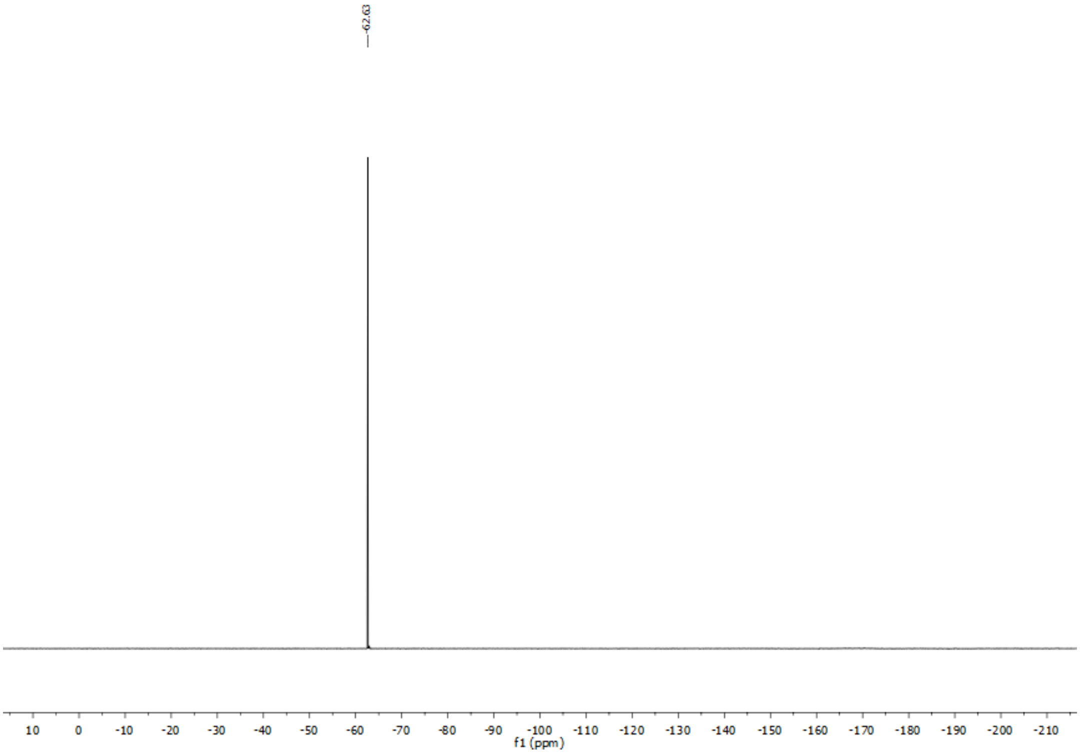

Figure S13. ^{19}F NMR of $\mathbf{1}_{\mathbf{CF3}}$ ((2-(4-(Trifluoromethyl)phenyl)benzofuran-3-yl)(triphenyl- λ5-phosphanyl) gold(I)).

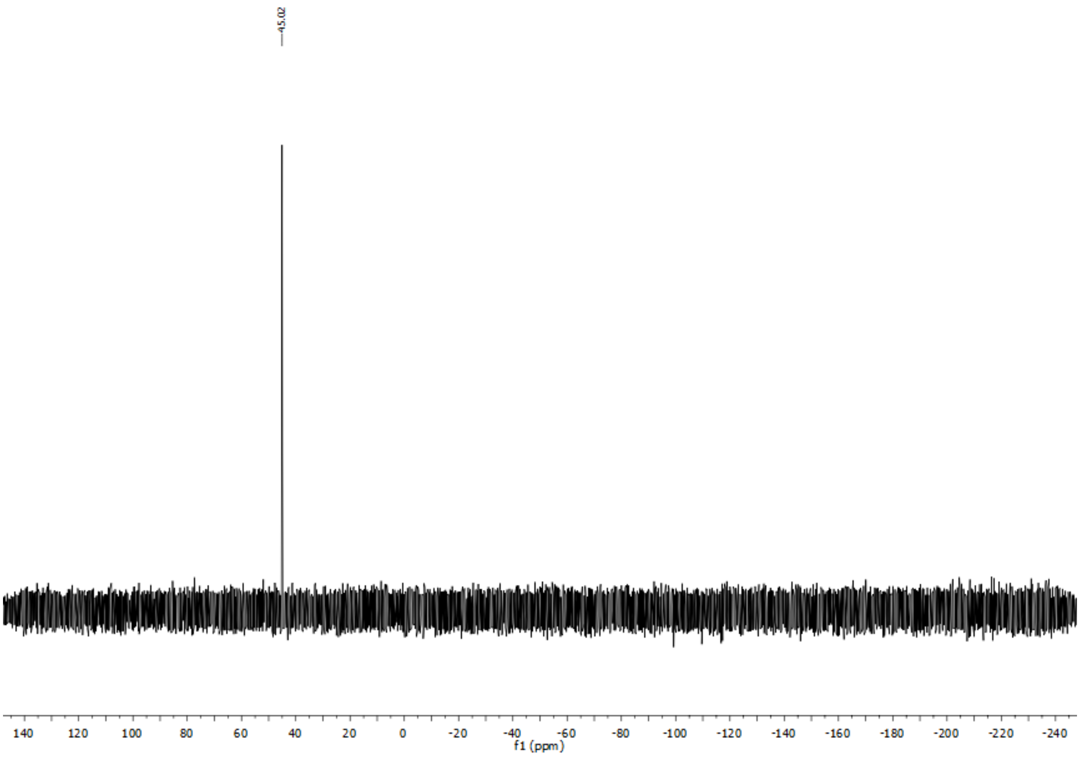

Figure S14. ^{31}P NMR of $\mathbf{1_{CF3}}$ ((2-(4-(Trifluoromethyl)phenyl)benzofuran-3-yl)(triphenyl- λ5-phosphanyl) gold(I)).

3. Kinetic studies

3.1 General procedures

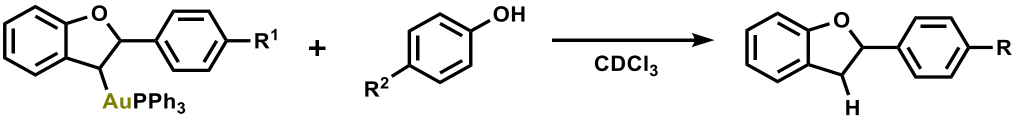

1 **2**

Scheme 1. Protodearuation of **1** with *p*-substituted phenols (**2**).

400 µL of a stock solution of gold(I) complex (**1**) in $CDCl_3$ (Stock solution: 6.25 mM including 10 µL tetrachloroethane in 3 mL $CDCl_3$ as internal standard, $CDCl_3$ freshly filtered over K_2CO_3) and 100 µL of a stock solution of para-substituted phenols (**2**) in $CDCl_3$ (250 mM of Stock solution in $CDCl_3$ freshly filtered over K_2CO_3) were added to a NMR tube and the mixture was shaken vigorously. The reaction was monitored with ^{1}H NMR spectroscopy (500 MHz) at 21.4 °C until complete conversion was reached. The reaction rate constants (k_R) were obtained by recording the decay of $\mathbf{1_R}$ in the presence of phenols sources. The time traces were fitted reasonably using an exponential decay profile (Eq. 1).

$$\mathbf{y = y_0 + A \cdot exp(-x \cdot \mathit{k_R})} \qquad \textbf{(Eq. 1)}$$

3.2 Kinetic experiments of the various substituted vinyl gold complexes (1) and 4-bromophenols for a Hammett Plot.

Table S1. Determination of the Hammett parameter for the protodeauration of a 5.0 mM solution of $\mathbf{1_H}$ using 10.0 eq 4-bromophenols.

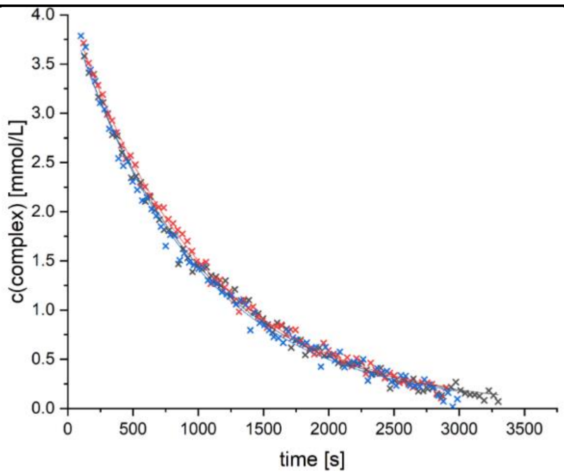

Entry	k_H [s^{-1}]	Average of k_H [s^{-1}]	Δ k_H [s^{-1}]	R^2	log (k_H/k_H)	Average of log (k_H/k_H)	Δ log (k_H/k_H)
**1**	1.02E-03	1.03E-03	± 0.02E-03	0.9958	0.0	0.00	-
**2**	1.01E-03			0.9973	0.0		
**3**	1.05E-03			0.9941	0.0		

Table S2. Determination of the Hammett parameter for the protodeauration of a 5.0 mM solution of $\mathbf{1}_{OMe}$ using 10.0 eq 4-bromophenol.

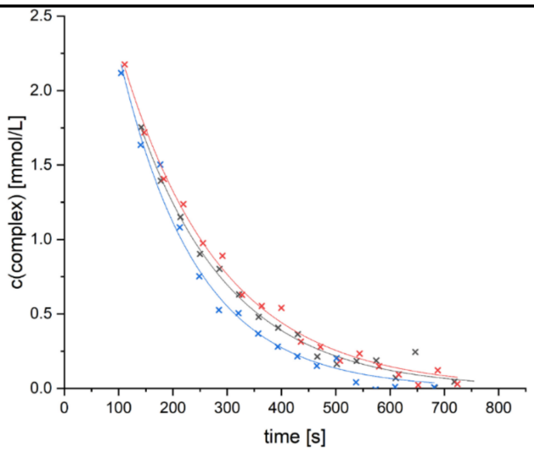

Entry	k_{OMe} [s^{-1}]	Average of k_{OMe} [s^{-1}]	Δ k_{OMe} [s^{-1}]	R^2	log (k_{OMe}/k_H)	Average of log (k_{OMe}/k_H)	Δ log (k_{OMe}/k_H)
**1**	5.81E-03	6.10E-03	± 0.81E-03	0.9888	7.53E-01	0.77	± 0.06
**2**	5.48E-03			0.9933	7.27E-01		
**3**	7.01E-03			0.9871	8.34E-01		

Table S3. Determination of the Hammett parameter for the protodeauration of a 5.00 mM solution of $\mathbf{1}_{Me}$ using 10.0 eq 4-bromophenol.

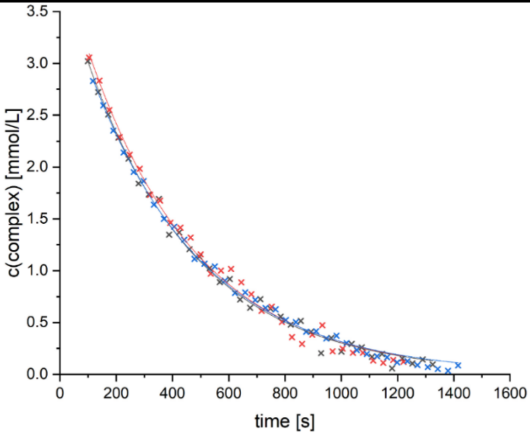

Entry	k_{Me} [s^{-1}]	Average of k_{Me} [s^{-1}]	Δ k_{Me} [s^{-1}]	R^2	log (k_{Me}/k_H)	Average of log (k_{Me}/k_H)	Δ log (k_{Me}/k_H)
**1**	2.55E-03	2.53E-03	± 0.04E-03	0.9949	3.95E-01	0.39	± 0.01
**2**	2.56E-03			0.9919	3.97E-01		
**3**	2.49E-03			0.9967	3.85E-01		

Table S4. Determination of the Hammett parameter for the protodeauration of a 5.0 mM solution of $\mathbf{1_F}$ using 10.0 eq 4-bromophenol.

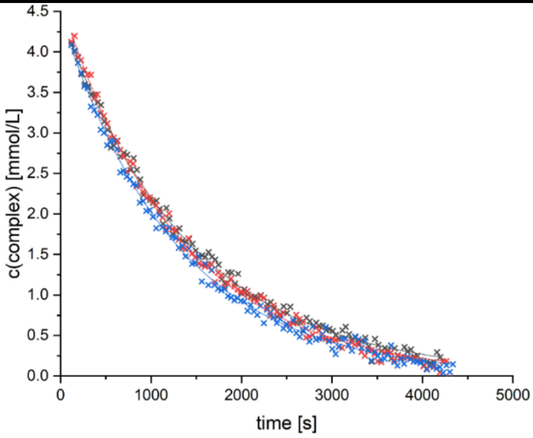

**Entry**	***k*$_F$** [s^{-1}]	**Average of *k*$_F$** [s^{-1}]	**Δ *k*$_F$** [s^{-1}]	**R^2**	**log (*k*$_F$/*k*$_H$)**	**Average of log (*k*$_F$/*k*$_H$)**	**Δ log (*k*$_F$/*k*$_H$)**
**1**	7.03E-04	7.51E-04	± 0.44E-04	0.9958	-1.65E-01	-0.14	± 0.03
**2**	7.64E-04			0.9962	-1.28E-01		
**3**	7.88E-04			0.9957	-1.15E-01		

Table S5. Determination of the Hammett parameter for the protodeauration of a 5.0 mM solution of $\mathbf{1_{CF3}}$ using 10.0 eq 4-bromophenol.

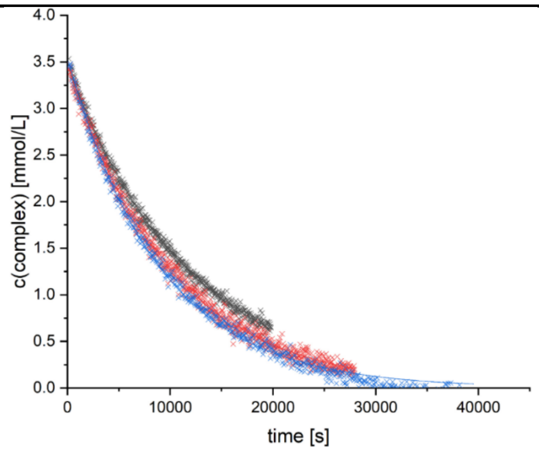

**Entry**	***k*$_{CF3}$** [s^{-1}]	**Average of *k*$_{CF3}$** [s^{-1}]	**Δ *k*$_{CF3}$** [s^{-1}]	**R^2**	**log (*k*$_{CF3}$/*k*$_H$)**	**Average of log (*k*$_{CF3}$/*k*$_H$)**	**Δ log (*k*$_{CF3}$/*k*$_H$)**
**1**	8.58E-05	9.95E-05	± 1.32E-05	0.9969	-1.08E+00	-1.02	± 0.06
**2**	1.01E-04			0.9956	-1.01E+00		
**3**	1.12E-04			0.9921	-9.62E-01		

Table S6. Rate constants for the protodeauration of different substituted **1** and the determination of log(k_R/k_H).

Entry	R	k_R [s^{-1}]	Δk_R [s^{-1}]	log (k_R/k_H)	Δlog (k_R/k_H)	σ_{theo}
**1**	**OMe**	6.10E-03	± 0.81E-03	0.77	± 0.06	- 0.27
**2**	**Me**	2.53E-03	± 0.04E-03	0.39	± 0.01	- 0.17
**3**	**H**	1.03E-03	± 0.02E-03	0.00	-	0.00
**4**	**F**	7.51E-04	± 0.44E-04	- 0.14	± 0.03	0.06
**5**	**CF_3**	9.95E-05	± 1.32E-05	- 1.02	± 0.06	0.54

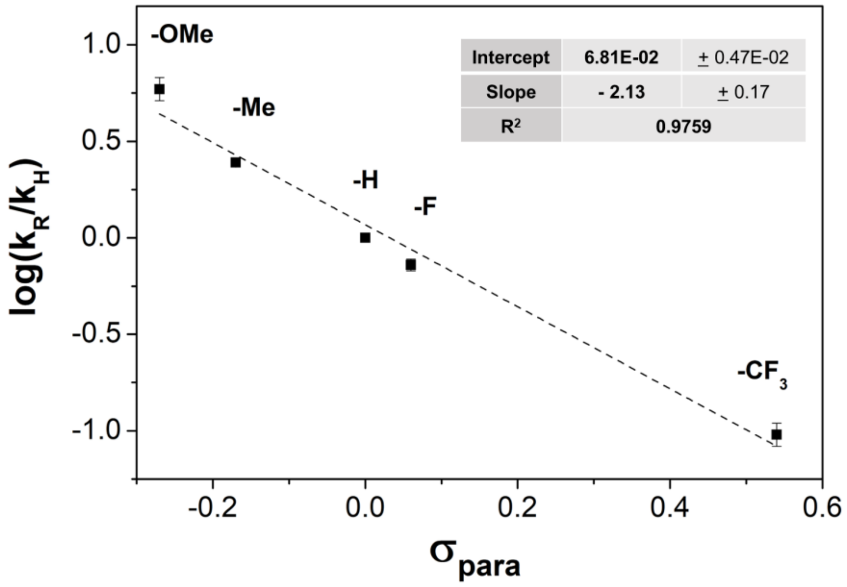

Figure S15. Experimentally obtained Hammett correlations of σ_{para}[7], the *para*-Hammett substituent parameter, *versus* log (k_R/k_H).

3.3 Kinetic Experiments of the 1_F and various amount of 4-bromophenols.

Table S7. Determination of $k_{F,10eq.}$ for the protodeauration of a 5.0 mM solution of F-substituted complex $\mathbf{1_F}$ by using 10.0 eq 4-bromophenol.

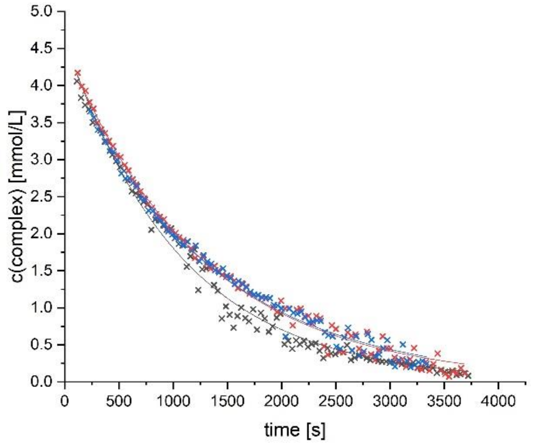

Entry	$k_{F,10eq.}$ [s^{-1}]	average of $k_{F,10eq.}$ [s^{-1}]	$\Delta k_{F,10eq.}$ [s^{-1}]	R^2
1	9.40E-04	8.32E-04	$\pm$ 0.95E-04	0.9898
2	7.95E-04			0.9918
3	7.63E-04			0.9905

Table S8. Determination of $k_{F,15eq.}$ for the protodeauration of a 5.0 mM solution of F-substituted complex $\mathbf{1_F}$ by using 15.0 eq 4-bromophenol.

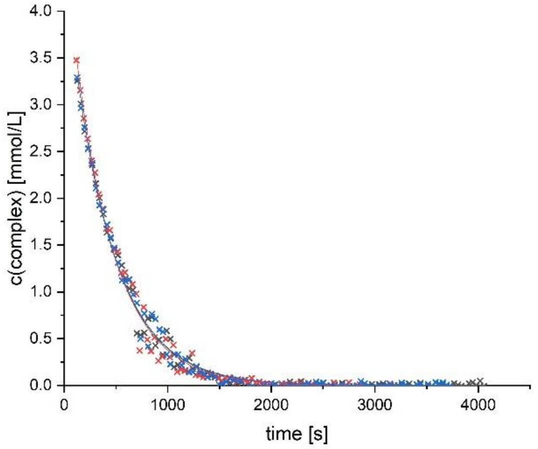

Entry	$k_{F,15eq.}$ [s^{-1}]	average of $k_{F,15eq.}$ [s^{-1}]	$k_{F,15eq.}$ [s^{-1}]	R^2
1	2.44E-03	2.46E-03	$\pm$ 0.06E-03	0.9922
2	2.53E-03			0.9900
3	2.41E-03			0.9921

Table S9. Determination of $k_{F,20eq.}$ for the protodeauration of a 5.0 mM solution of F-substituted complex $\mathbf{1_F}$ by using 20.0 eq 4-bromophenol.

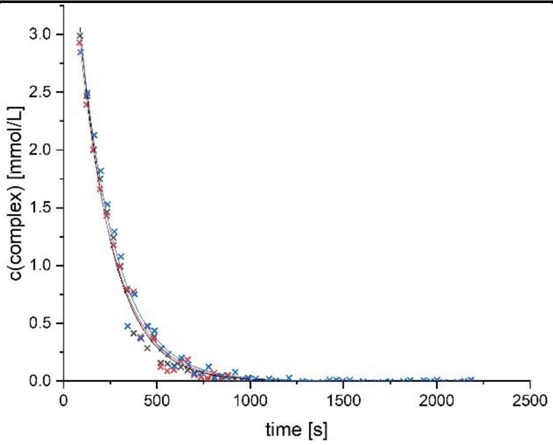

Entry	$k_{F,20eq.}$ [s^{-1}]	average of $k_{F,20eq.}$ [s^{-1}]	Δk $k_{F,20eq.}$ [s^{-1}]	R^2
**1**	5.68E-03	5.41E-03	$\pm$ 0.25E-03	0.9923
**2**	5.37E-03			0.9920
**3**	5.18E-03			0.9890

Table S10. Determination of $k_{F,25eq.}$ for the protodeauration of a 5.0 mM solution of F-substituted complex $\mathbf{1_F}$ by using 25.0 eq 4-bromophenol.

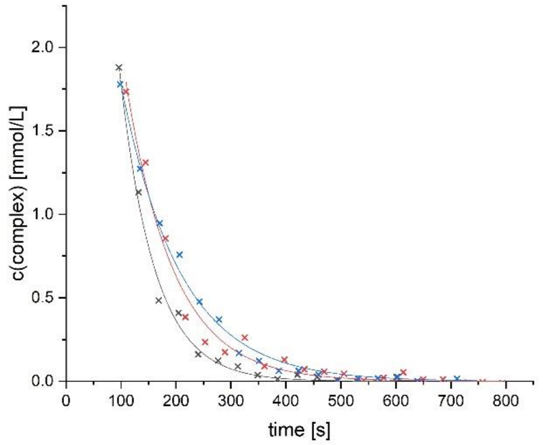

Entry	$k_{F,25eq.}$ [s^{-1}]	average of $k_{F,25eq.}$ [s^{-1}]	$\Delta k_{F,25eq.}$ [s^{-1}]	R^2
**1**	1.59E-02	1.22E-02	$\pm$ 0.34E-02	0.9931
**2**	1.15E-02			0.9820
**3**	9.31E-03			0.9939

Table S11. Determination of $k_{F,30eq.}$ for the protodeauration of a 5.0 mM solution of F-substituted complex $\mathbf{1_F}$ by using 30.0 eq 4-bromophenol.

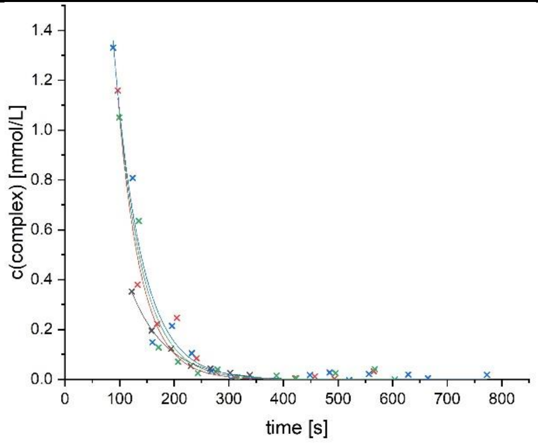

**Entry**	$k_{F,30eq.}$ [s^{-1}]	**average of** $k_{F,30eq.}$ [s^{-1}]	$\Delta k_{F,30eq.}$ [s^{-1}]	R^2
**1**	1.55E-02	1.99E-02	± 0.33E-02	0.9965
**2**	2.32E-02			0.9680
**3**	1.96E-02			0.9706
**4**	2.12E-02			0.9719

3.4 Determination of Second Order Rate Constant and Gibbs Free Energy

The second order rate constant k_2 was determined, using the values of k_F for 10.0 eq, 15.0 eq, 20.0 eq and 25.0 eq of *p*-bromophenol. The data for 30.0 eq *p*-bromophenol are excluded due to the fast reaction time, which does not allow for a proper monitoring of the reaction (see **Table S12**).

Table S12. Rate constants for the protodeauration of F-substituted complex $\mathbf{1_F}$ towards different concentrations of 4-bromophenol ($\mathbf{2_{Br}}$).

Entry	4-bromophemol [mM]	k_F [s^{-1}]	average of k_F [s^{-1}]	Δk_F [s^{-1}]
**1**	50.1E-03	9.40E-04	8.32E-04	± 0.95E-04
**2**		7.95E-04		
**3**		7.63E-04		
**1**	75.0 E-03	2.44E-03	2.46E-03	± 0.06E-03
**2**		2.53E-03		
**3**		2.41E-03		
**1**	100.1 E-03	5.68E-03	5.41E-03	± 0.25E-03
**2**		5.37E-03		
**3**		5.18E-03		
**1**	125.1 E-03	1.59E-02	1.22E-02	± 0.34E-02
**2**		1.15E-02		
**3**		9.31E-03		
**1**	250.0 E-03	1.55E-02	1.99E-02	± 0.33E-02
**2**		2.32E-02		
**3**		1.96E-02		
**4**		2.12E-02		

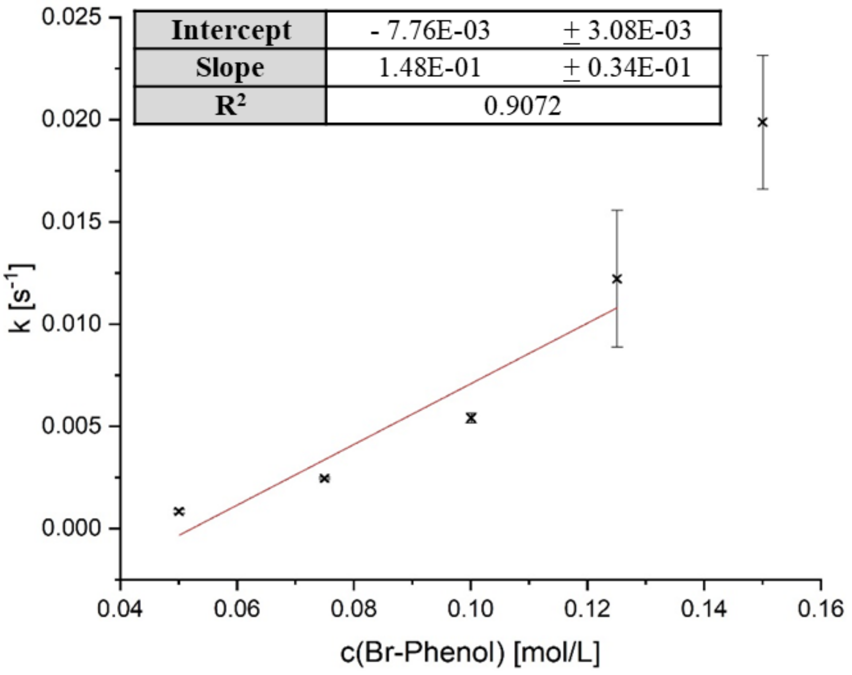

Figure S16. Determination of the second-order rate constant k_2 of $\mathbf{1_F}$ for protodeauration by $\mathbf{2_{Br}}$.

3.5. Kinetic Experiments of 1_F and various *p*-substituted phenols (2).

Table S13. Determination of the Hammett parameter for the protodeauration of a 5.02 mmol/L solution of complex $\mathbf{1_{OMe}}$ using 10.0 eq phenol (49.9 mmol/L in $CDCl_3$)

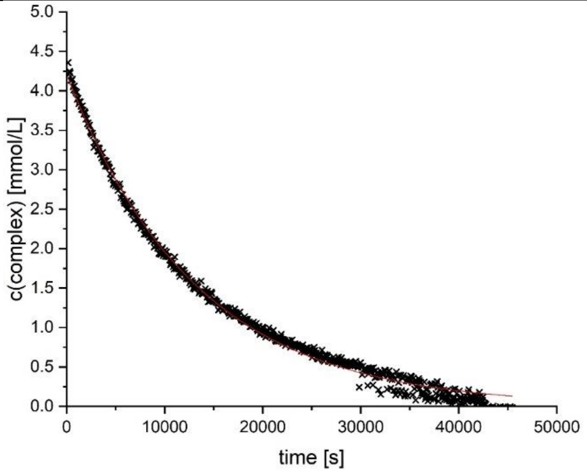

Entry	k_H [s^{-1}]	R^2	log (k_H/k_H)	σ_{theo}
**1**	7.64E-05	0.9942	0.00	0.00

Table S14. Determination of the Hammett parameter for the protodeauration of a 5.02 mmol/L solution of complex **1**$_{OMe}$ using 10.0 eq 4-methoxyphenol (50.3 mmol/L in $CDCl_3$).

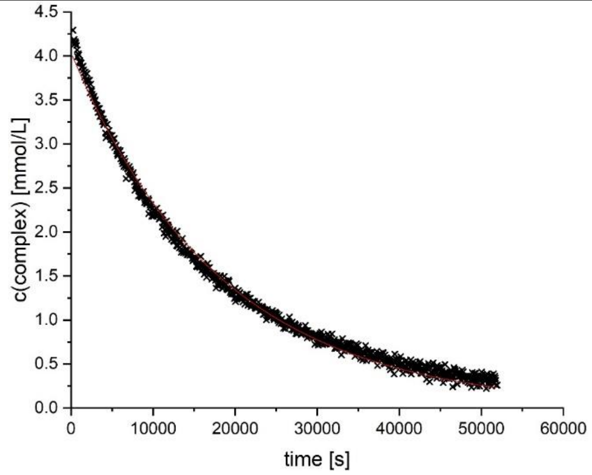

Entry	k_{OMe} [s^{-1}]	R^2	log (k_{OMe}/k_H)	σ_{theo}
**1**	5.50E-05	0.9942	-0.14	-0.27

Table S15. Determination of the Hammett parameter for the protodeauration of a 5.02 mmol/L solution of complex **1**$_{OMe}$ using 10.0 eq 4-dimethylaminophenol (55.3 mmol/L in $CDCl_3$).

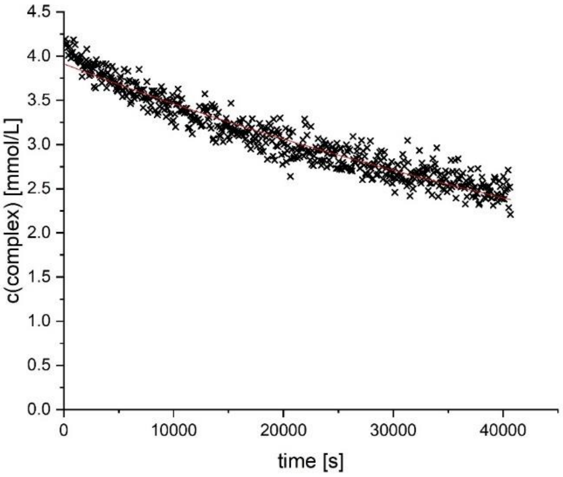

Entry	$k_{N(CH3)2}$ [$^{-}s^{-1}$]	R^2	log ($k_{N(CH3)2}/k_H$)	σ_{theo}
**1**	1.23E-05	0.9274	-0.79	-0.83

Table S16. Determination of the Hammett parameter for the protodeauration of a 5.02 mmol/L solution of complex $\mathbf{1}_{\mathbf{OMe}}$ using 10.0 eq 4-fluorophenol (50.0 mmol/L in $CDCl_3$).

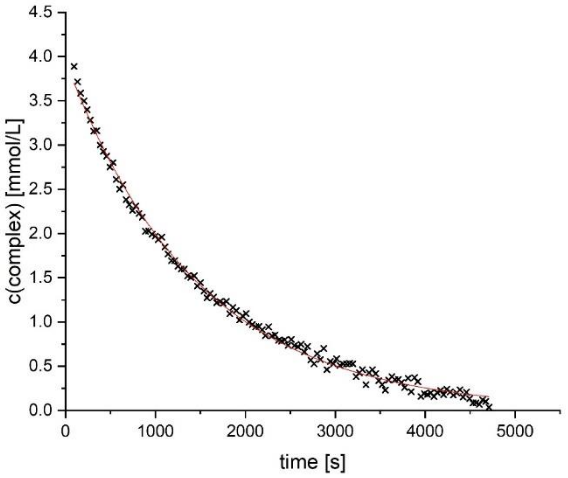

Entry	k_F [s^{-1}]	R^2	log (k_F/k_H)	σ_{theo}
**1**	6.85E-04	0.9960	0.95	0.06

Table S17. Determination of the Hammett parameter for the protodeauration of a 5.02 mmol/L solution of complex $\mathbf{1}_{\mathbf{OMe}}$ using 10.0 eq 4-chlorophenol (43.6 mmol/L in $CDCl_3$).

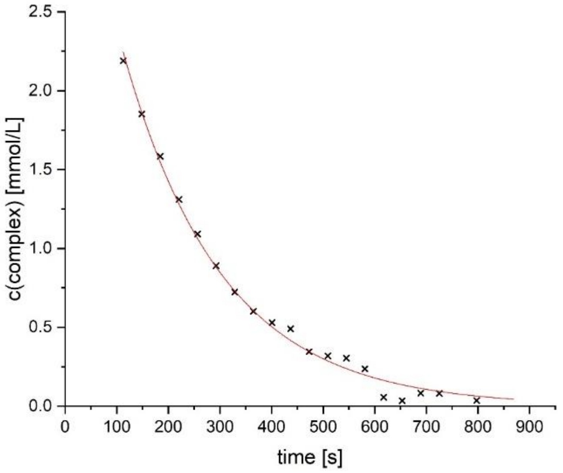

Entry	k_{Cl} [s^{-1}]	R^2	log (k_{Cl}/k_H)	σ_{theo}
**1**	5.19E-03	0.9853	1.83	0.23

Table S18. Rate constants for the protodeauration of complex $\mathbf{1_{OMe}}$ and the determination of log(k_R/k_H).

**Entry**	**R**	k_R [s^{-1}]	**log (k_R/k_H)**	σ_{theo}
**1**	**OMe**	5.50E-05	- 0.14	- 0.83
**2**	**$N(CH_3)_2$**	1.23E-05	- 0.79	- 0.27
**3**	**H**	7.64E-05	0	0
**4**	**F**	6.85E-04	0.95	0.06
**5**	**Cl**	5.19E-03	1.83	0.23
**6**	**Br**	6.10E-03	1.90	0.23

4. Computational details

4.1 Geometry optimizations and relative energies of all species.

All calculations were performed in ORCA 4.2.1. Geometries were optimized at the PBEh-3c/cPCM(chloroform) level of theory.[8] Increased accuracy of the DFT integration grid was requested via the *Grid6 NoFinalGrid* keywords. The SCF energetic convergence criterion was set to 10^{-8} au (*TightSCF*). Solvent effects were modelled implicitly (*cPCM*)[9], with chloroform specified as the solvent, using a modified (van der Waals Gaussian) cavity (*vdw_gaussian*)[10].

Electronic energies were refined using the PW6B95-D3(BJ) functional[11] and the larger def2-TZVPP[12] basis set in combination with Weigend's universal fitting basis set (*def2/J*).[12][ref] These calculations utilized the *RIJCOSX* approximation to speed up the two electron integrals.

Table S19. TS structures of **1** and $\mathbf{2_{Br}}$.

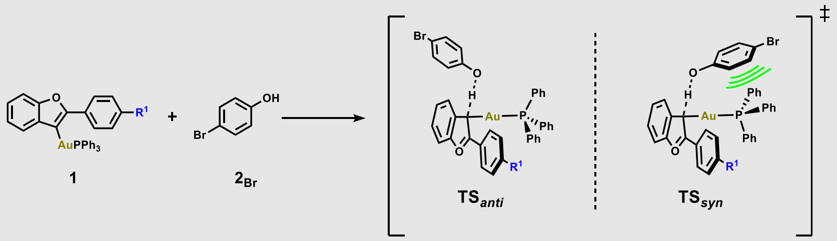

R_1 =	OMe	Me	H	F	CF_3
TS*anti*	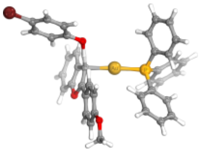	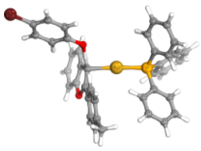	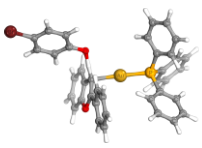	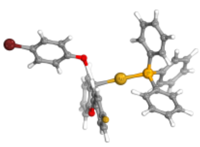	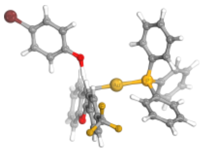
TS*syn*	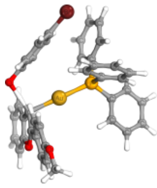	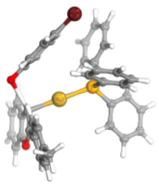	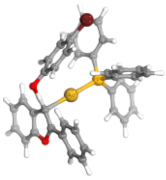	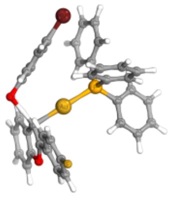	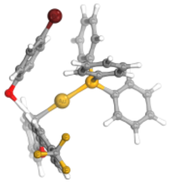

Table S20. Energy profile of **1** and $\mathbf{2_{Br}}$

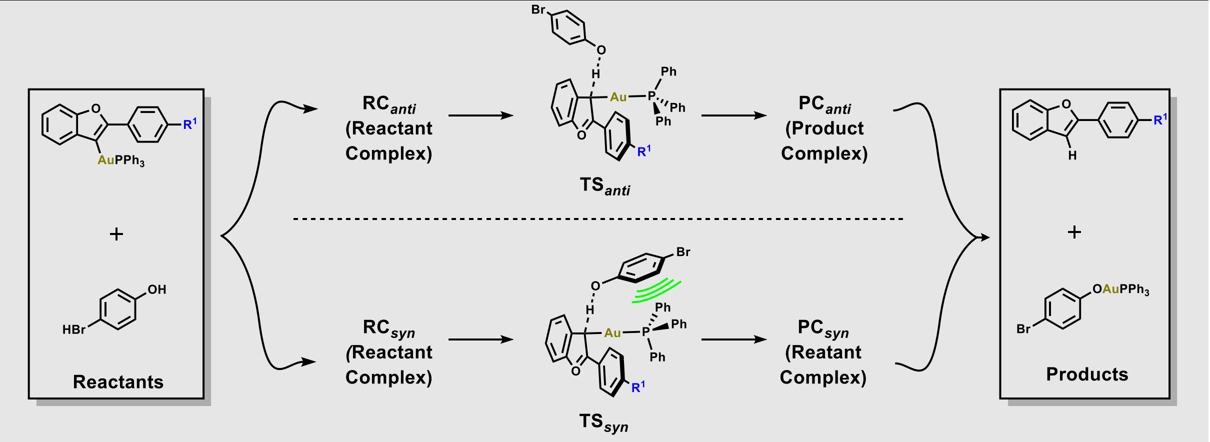

PW6B95-D3(BJ)/def2-TZVPP/cPCM//PBEh-3c/cPCM
$\Delta G_{298.15}$ (kcal mol^{-1})

R_1	Reactants		RC	TS	PC	Products
OMe	0.0	*anti*	0.8	23.9	-3.8	-4.0
		syn	0.0	18.0	14.3	
Me	0.0	*anti*	0.6	24.4	-4.5	-4.2
		syn	-0.2	18.4	6.1	
H	0.0	*anti*	1.1	25.3	-3.8	-3.8
		syn	0.3	19.4	6.8	
F	0.0	*anti*	1.1	25.2	-4.0	-3.3
		syn	-0.1	19.4	7.4	
CF_3	0.0	*anti*	3.4	26.7	-3.6	-3.2
		syn	-0.1	20.6	7.8	

Table S21. TS structures of $\mathbf{1_{OMe}}$ and **2**.

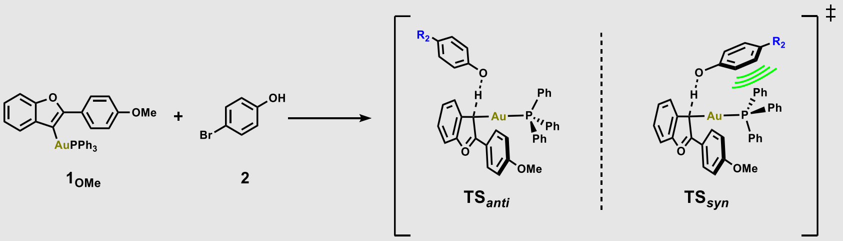

R_1 =	Cl	F	H	OMe	NMe_2
**TS*anti***	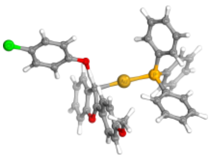	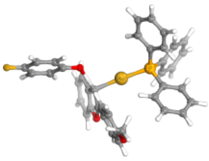	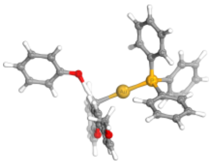	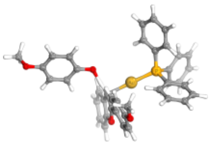	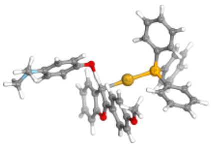
**TS*syn***	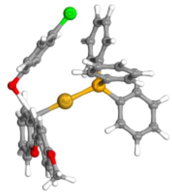	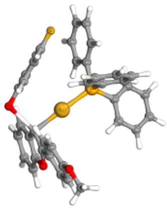	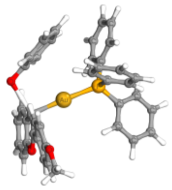	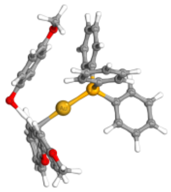	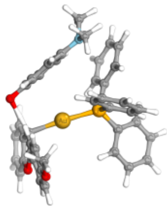

Table S22. Energy profile of $\mathbf{1_{OMe}}$ and **2**

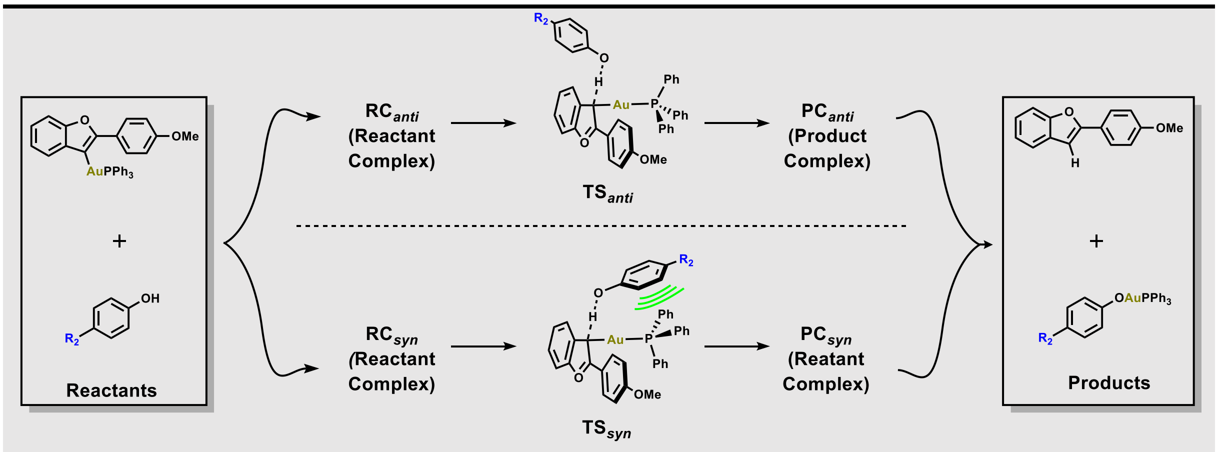

PW6B95-D3(BJ)/def2-TZVPP/cPCM//PBEh-3c/cPCM $\Delta G_{298.15}$ (kcal mol^{-1})						
R_1	Reactants		RC	TS	PC	Products
Cl	0.0	*anti*	0.8	24.3	-5.0	-5.7
		syn	0.2	18.5	14.4	
F	0.0	*anti*	2.8	25.6	-4.1	-5.1
		syn	0.7	19.8	7.9	
H	0.0	*anti*	3.0	25.7	-3.2	-4.9
		syn	1.0	20.5	7.6	
OMe	0.0	*anti*	2.9	28.6	-3.6	-4.1
		syn	0.2	20.4	8.7	
NMe_2	0.0	*anti*	1.3	28.7	-1.6	-2.9
		syn	0.2	20.9	8.7	

4.2 Characterization of NCIs

4.2.1 Quantification of NCIs

We probed the role of NCIs via calculations with B3LYP-D3(BJ)/def2-TZVPP//PBEh-3c in ORCA 4.2.1. We chose this approach as the parameterization of B3LYP is known to lack almost all dispersion effects.[13] This enables a relatively clean separation between the dispersion energy (given by the D3 model from Grimme *et al.*) and all other electronic effects (given by E_{SCF}). Increased accuracy of the DFT integration grid was requested via the *Grid6 NoFinalGrid* keywords. The SCF energetic convergence criterion was set to 10^{-8} au (*TightSCF*). Solvent effects were modelled implicitly (*cPCM*) [9], with chloroform specified as the solvent, using a modified (van der Waals Gaussian) cavity (*vdw_gaussian*)[10]. While the theoretical Hammett plots with and without dispersion corrections are very similar for TS_{syn} (Figure S17), they show divergence for TS_{anti} (Figure S18). Without the dispersion correction, the Hammett correlation is ~linear, but is V-shaped when dispersion is included in the calculations. This further validates our hypothesis that Hammett non-linearity is indicative of NCIs playing a non-negligible role, as discussed in the main text.

To obtain the dispersion energy between the phenol and the vinyl gold complex, *E(disp)*, the standalone DFTD3 program (v3.1) was used. These calculations defined two fragments: the phenol with its substituent (2_R) and the vinyl gold complex (1_{OMe}). The *E(disp)* term we use is simply the sum over the atomic pairwise dispersion interactions between the atoms in each fragment. D3 parameterization for triple zeta basis sets was requested via the *-tz* flag.

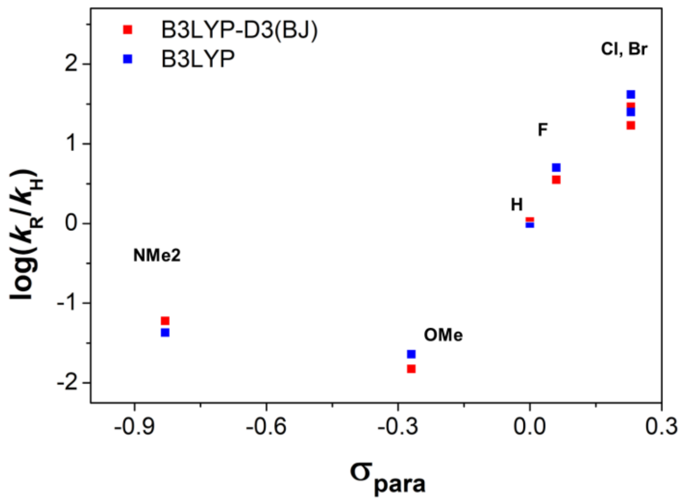

Figure S17. Theoretically obtained Hammett correlations of *anti*- conformers with PW6B95/def2-TZVPP/cPCM//PBEh-3c/cPCM with D3(BJ) (red squares) and without D3(BJ) (blue squares).

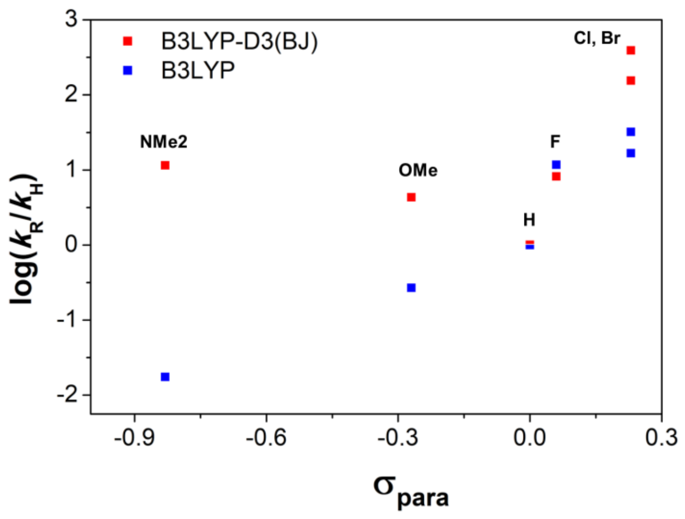

Figure S18. Theoretically obtained Hammett correlations of *syn*- conformers with PW6B95/def2-TZVPP/cPCM//PBEh-3c/cPCM with D3(BJ) (red squares) and without D3(BJ) (blue squares).

Table S23: Relative electronic energies of the transition states (**TS**$_{anti}$ and **TS**$_{syn}$), referenced to the separated reactants (**1**$_{OMe}$ and **2**), calculated with B3LYP-D3(BJ)/def2-TZVPP//PBEh-3c.

R^2	**TS**$_{anti}$	**TS**$_{syn}$	$\Delta\Delta E^{\ddagger}_{anti\text{-}syn}$
**Br**	11.26	3.26	8.00
**Cl**	11.58	3.81	7.77
**F**	12.51	5.55	6.96
**H**	13.26	6.80	6.45
**OMe**	15.75	5.93	9.82
**CF_3**	9.89	3.44	6.44
**NMe_2**	14.93	5.35	9.58
**Me**	14.04	5.78	8.27

Table S24: Dispersion energies between the phenol and vinyl gold fragments in the optimized transition states (**TS**$_{anti}$ and **TS**$_{syn}$) calculated with D3(BJ)//PBEh-3c.

R^2	**TS**$_{anti}$	**TS**$_{syn}$	$\Delta E(disp)_{anti\text{-}syn}$
**Br**	-7.38	-20.6	13.24
**Cl**	-7.27	-15.7	13.16
**F**	-20.4	-17.5	11.48
**H**	-7.13	-7.27	11.04
**OMe**	-18.2	-20.4	13.27
**CF_3**	-12.3	-11.6	11.40
**NMe_2**	-10.2	-22.6	12.34
**Me**	-22.6	-11.6	13.25

4.2.2 NCI plots

The RDG isosurfaces shown in Figure 5 are based on calculations performed in ORCA 4.2.1, using the B3LYP-ZORA-D3(BJ)//PBEh-3c level of theory. The *ZORA/def2-TZVPP* basis set was used for all atoms, apart from gold, which used *SARC-ZORA-TZVP*. For density fitting, a contracted version of Weigend's *def2/J* universal fitting basis set (SARC/J) was used. The *orca_2aim* utility was used to create .*wfn* files, which were used to perform the topological NCI analysis of the calculated density in MultiWFN v3.8.[14] Finally, the isosurfaces were plotted in VMD,[15] using s=0.45 au. The Reduced Density Gradient (RDG) isosurfaces were colored with a blue-green-red scale of sign(λ_2)ρ, from 0.02 to -0.35 au, such that blue, green and red indicate H-bonds, favorable vdW interactions and steric clashes, respectively (Figures 5 and S19-S20).

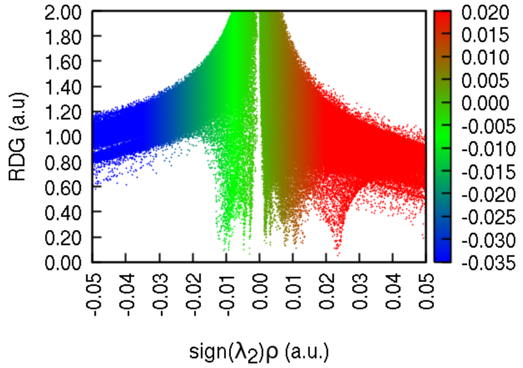

Figure S19: NCI plot for H-TS$_{anti}$, calculated with B3LYP-ZORA-D3(BJ)/def2-TZVPP//PBEh-3c.

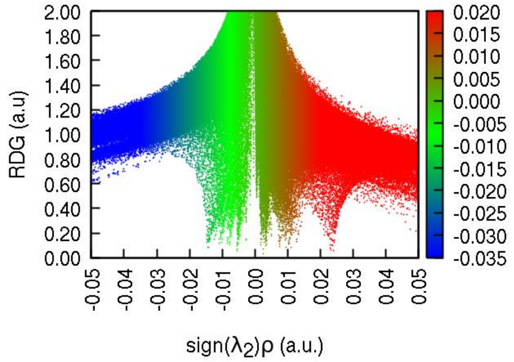

Figure S20: NCI plot for H-TS$_{syn}$, calculated with B3LYP-ZORA-D3(BJ)/def2-TZVPP//PBEh-3c.

5. Cartesian Coordinates

1	C -0.31242683162069 -4.32955055844659 -1.55291194018240<br>C -0.33264465383352 -5.47639776071623 -0.77349713605412<br>C 0.82370509757140 -5.89224816839934 -0.13376473523330<br>C 1.99976471570040 -5.16724702613980 -0.26787857927162<br>C 2.01774156241079 -4.02298791626666 -1.04677663006389<br>C 0.86180994416599 -3.59532049195391 -1.69678060252993<br>H -1.22278230637626 -4.01331118810656 -2.04949563084188<br>H -1.25492104787381 -6.03326524143462 -0.67560487457659<br>Br 0.80035394702063 -7.45541010689407 0.92666359221330<br>H 2.90729575393538 -5.48440352413490 0.22823697607047<br>H 2.93474851548345 -3.45759775695069 -1.15268882754597<br>O 0.94083461875249 -2.47773070881156 -2.44182808123086<br>H 0.09289258166376 -2.28164466574505 -2.85498635775323	**4-Bromophenol (2_{Br})**<br>$E_{PBEh-3c/CPCM(Chloroform)}$=<br>-2879.21779248<br>$ZPE_{PBEh-3c/CPCM(Chloroform)}$=<br>0.09768465<br>$FEC_{(298.15)PBEh-3c/CPCM(Chloroform)}$=<br>0.06559861<br>$E_{PW6B95-D3(BJ)/def2-TZVPP/CPCM(Chloroform)}$=<br>-2882.68081048013<br>$E_{B3LYP-D3(BJ)/def2-TZVPP/CPCM(Chloroform)}$=<br>-2880.888180334329
2	Au -1.06825245998776 -4.03722861328497 -6.35890375139459<br>P -0.73602527873000 -5.41851370540138 -4.50926187767147<br>O -1.41579240716467 -0.95092238577679 -9.28512455193410<br>C -0.96395049857407 -1.49469349323359 -8.10468971929070<br>C -1.34462347779652 -2.79450095132192 -7.94748198273829<br>C -2.09125558866588 -3.08615330360369 -9.14972751922622<br>C -2.75559614062973 -4.21262013332350 -9.63839870704093<br>H -2.77178447120770 -5.13307981588481 -9.06702969703050<br>C -3.39231470336081 -4.13146901001744 -10.86552455513924<br>H -3.91082807048525 -4.99656237593136 -11.25840397390478<br>C -3.37938496744748 -2.94677046448706 -11.61150155775210<br>H -3.88585070638301 -2.91374947025397 -12.56725346015437<br>C -2.72912937228136 -1.81236126951684 -11.14781534748569<br>H -2.71585538332339 -0.89306344744424 -11.71882547443774<br>C -2.10103956677095 -1.91976459486821 -9.91963640093922<br>C -0.20868575486952 -0.57238592265108 -7.26403661666141<br>C 0.79437516755064 -1.02753031398203 -6.40105416287201<br>H 1.06056348329939 -2.07594982898721 -6.38808309046020<br>C 1.48500075498981 -0.16150710494103 -5.57914065598315<br>H 2.25807603898379 -0.53774367581145 -4.92057032189673<br>C 1.20878889108021 1.20717053495230 -5.60309830178283<br>C 0.23165499067004 1.68246345613362 -6.47338572257414<br>H -0.01102509068070 2.73487601992268 -6.52879929362390<br>C -0.46132620312450 0.79833502227746 -7.28890218532798<br>H -1.22467175450093 1.19872451080197 -7.94298860046338<br>C 0.37536134114595 -4.66078136922391 -3.28431201933279<br>C 1.52858259448169 -4.02672426511463 -3.74677592124786<br>H 1.74265001228202 -3.98642726741942 -4.80852110769894<br>C 2.41078449656139 -3.44353704295648 -2.85144073880330<br>H 3.30298512194489 -2.95379470720435 -3.21887620470013<br>C 2.14425502361700 -3.48110726080783 -1.48842106124369<br>H 2.82931890487413 -3.01878640556698 -0.78990018125519<br>C 0.99671732699317 -4.10735233015143 -1.02455968196168<br>H 0.78465070753244 -4.13726556193342 0.03605924169669<br>C 0.11325268884664 -4.69836086763214 -1.91819081883804<br>H -0.77856465198622 -5.18326917381095 -1.54283741152221<br>C 0.01929385126562 -7.02480884566384 -4.90029682712719<br>C 0.97698303326276 -7.61074197129325 -4.07684535751084<br>H 1.31555244569660 -7.10426170908559 -3.18205664027376<br>C 1.50685654204846 -8.85174933573745 -4.40188914532095<br>H 2.25314876307382 -9.30042372162777 -3.75960403004501<br>C 1.08187473809846 -9.51383510660642 -5.54503646646404<br>H 1.49775074569174 -10.48056047702950 -5.79712781639307<br>C 0.12692623560108 -8.93245515833110 -6.36909956104506<br>H -0.20321061208061 -9.44274625281516 -7.26419886798008<br>C -0.39956211044931 -7.68994355966850 -6.05180515826991<br>H -1.13698156345262 -7.23916338013124 -6.70576908700693<br>C -2.25206468989904 -5.81980539163906 -3.58970092752973<br>C -3.19947634179175 -4.81282716158652 -3.40763336905355<br>H -3.03754490429984 -3.82730122545523 -3.82859792704676<br>C -4.35549163155717 -5.06616046329060 -2.68608209236598<br>H -5.08509925439626 -4.27932745022508 -2.54767105980898<br>C -4.57933989096169 -6.32875431646313 -2.15213413663365<br>H -5.48553033107269 -6.52801941858742 -1.59518780976761<br>C -3.64325627039267 -7.33576561616784 -2.33851874423855	**1_{OMe}**<br>$E_{PBEh-3c/CPCM(Chloroform)}$=<br>-1897.24046558<br>$ZPE_{PBEh-3c/CPCM(Chloroform)}$=<br>0.51081401<br>$FEC_{(298.15)PBEh-3c/CPCM(Chloroform)}$=<br>0.45046691<br>$E_{PW6B95-D3(BJ)/def2-TZVPP/CPCM(Chloroform)}$=<br>-1903.57755743961<br>$E_{B3LYP-D3(BJ)/def2-TZVPP/CPCM(Chloroform)}$=<br>-1900.536294588074

	H -3.81592613115663 -8.32162338694044 -1.92733946273927<br>C -2.48023971540812 -7.08398443447695 -3.05340836724961<br>H -1.75741081019019 -7.87780056680231 -3.19045169768472<br>O 1.93122610736729 1.97847327219270 -4.77022858288128<br>C 1.70056002380554 3.37032760955616 -4.77064973853050<br>H 1.90517632239443 3.81994411840983 -5.74630510335172<br>H 0.67588174304814 3.61684566854153 -4.47877497677266<br>H 2.38315578387199 3.79766423738040 -4.04000879621566	
3	Au -1.04964108309288 -4.03805232129716 -6.33117446739945<br>P -0.72332542918547 -5.43808369525103 -4.50053903968878<br>O -1.25392060359590 -0.98144872594166 -9.31567513564869<br>C -0.91327599224478 -1.47929829257862 -8.08007269084870<br>C -1.23362699813581 -2.80054220686871 -7.94342005592188<br>C -1.81906009370864 -3.14936950266926 -9.21869932594362<br>C -2.35387763398536 -4.32075742691434 -9.75895803864814<br>H -2.38581179735046 -5.23250695386048 -9.17412579817529<br>C -2.83913408709523 -4.29482142957233 -11.05577264669475<br>H -3.25550955569656 -5.19451179113802 -11.49007435687738<br>C -2.80351394214730 -3.12178753545587 -11.81925302644636<br>H -3.19056048891143 -3.13258453223450 -12.82970585931055<br>C -2.28178259221142 -1.94381285201952 -11.30468348459220<br>H -2.25207076025425 -1.03360140897846 -11.88936174400267<br>C -1.80304204591182 -1.99683000718842 -10.00769964049057<br>C -0.32552626864654 -0.49429596055474 -7.17760521889966<br>C 0.53582987640506 -0.86864422388253 -6.13961565012394<br>H 0.82185233683846 -1.90396853020599 -6.01397539693818<br>C 1.06506341592521 0.06274892948269 -5.27147370058000<br>H 1.73170208096716 -0.25323327337594 -4.47874754521790<br>C 0.76265799830002 1.41841210107036 -5.41705090722170<br>C -0.07616400058582 1.81309788764721 -6.45551074677702<br>H -0.33495854211961 2.85191362309888 -6.60842122476303<br>C -0.60644268947516 0.86428791543012 -7.31866554425189<br>H -1.26464872352212 1.20537575034762 -8.10655814408516<br>C 0.56529601610447 -4.79963954411371 -3.38624720614695<br>C 1.71847815869703 -4.25619183092624 -3.95320177410244<br>H 1.83071325166392 -4.20594262425622 -5.03004066290949<br>C 2.73281733644463 -3.77375205787696 -3.14129061507695<br>H 3.62404251282185 -3.35426351421925 -3.58910658681012<br>C 2.60056981783558 -3.82142368708429 -1.75910564228838<br>H 3.39000863135112 -3.43799306784747 -1.12601053667098<br>C 1.45314999659689 -4.35601638727604 -1.19175538998366<br>H 1.34462588030150 -4.39253414417035 -0.11580340854138<br>C 0.43689581299316 -4.84609187680754 -2.00122712210964<br>H -0.45325785185381 -5.26013558547869 -1.54547047050981<br>C -0.17458959423899 -7.10839527373934 -4.95608710819944<br>C 0.82647439640645 -7.77651936297638 -4.25723660539244<br>H 1.32879501096572 -7.29907374780827 -3.42578154991292<br>C 1.18862366651366 -9.06480097499524 -4.62699974427302<br>H 1.96990141193805 -9.57834370869612 -4.08216522420613<br>C 0.55296554921770 -9.69025142440293 -5.68952797200960<br>H 0.84032857759254 -10.69226208329638 -5.97934750919451<br>C -0.44458310649612 -9.02505002672669 -6.39023108106765<br>H -0.93308602101047 -9.50509218063111 -7.22742619072898<br>C -0.80423610615565 -7.73701947861738 -6.02989821786092<br>H -1.57504088518807 -7.22029419095966 -6.59001230252748<br>C -2.18287582488237 -5.68718730407302 -3.44664730804576<br>C -3.02094890351103 -4.59823134259490 -3.21042830659716<br>H -2.81254757766705 -3.63885977152106 -3.66976150058944<br>C -4.12729820149322 -4.73642946440250 -2.38686389476595<br>H -4.77255822241395 -3.88671564632420 -2.20730131741192<br>C -4.41050745520372 -5.96462602113243 -1.80330446397412<br>H -5.27853979554780 -6.07386556378292 -1.16639312044650<br>C -3.58333574791929 -7.05283901871518 -2.04226818641899<br>H -3.80315637557157 -8.01200748454291 -1.59229299119435<br>C -2.47018927497630 -6.91674210232261 -2.86016723116666<br>H -1.83198340353181 -7.77262376459429 -3.03883175122563<br>C 1.02794056413294 -5.63554424447627 -9.13150530992984<br>C 0.97965745252365 -6.98138484114765 -9.46116462267498<br>C 1.83002159875986 -7.87398043884097 -8.82921928751298<br>C 2.71853570463629 -7.43269959065964 -7.85973292217098<br>C 2.76262711164889 -6.08831274600046 -7.52962116439167<br>C 1.92555481590364 -5.17797696262684 -8.17047022395972<br>H 0.36596503695541 -4.94343657076172 -9.63843726213801<br>H 0.27905558825032 -7.31507556510262 -10.21508988287475<br>Br 1.78806801957127 -9.70311282262592 -9.30109047804728	**RC*anti* (1OMe and 2Br)**<br>$E_{PBEh-3c/CPCM(Chloroform)}$=<br>-4776.47485835<br>$ZPE_{PBEh-3c/CPCM(Chloroform)}$=<br>0.60937738<br>$FEC_{(298.15)PBEh-3c/CPCM(Chloroform)}$=<br>0.54024367<br>$E_{PW6B95-D3(BJ)/def2-TZVPP/CPCM(Chloroform)}$=<br>-4786.27832846391<br>$E_{B3LYP-D3(BJ)/def2-TZVPP/CPCM(Chloroform)}$=<br>-4781.446053483069

	H 3.37664220365973 -8.12504293866706 -7.35230239678135<br>H 3.45487082978357 -5.74251261670461 -6.77233988866212<br>O 2.03125199192251 -3.87931769813088 -7.83161555680341<br>H 1.29161288334684 -3.37151485195530 -8.19341205817698<br>O 1.32372247687256 2.25578622997463 -4.52698100624326<br>C 1.05929751608343 3.63716833169062 -4.64099139288891<br>H 1.40501101482739 4.04341103832488 -5.59557635725944<br>H -0.00499676540678 3.86171279647140 -4.52866883403297<br>H 1.60511477158880 4.12423969376923 -3.83654521009550	
4	Au -0.94842974296794 -4.64173771658856 -6.12611596427685<br>P -0.83919847604178 -5.27350945722640 -3.92582223240345<br>O -1.91693657872001 -1.85918019104308 -8.32139397044991<br>C -0.83435312322706 -2.54075135445042 -7.92064402093664<br>C -0.93893756354964 -3.91063776140379 -8.16762054747664<br>C -2.23446357106629 -4.02884558124575 -8.82732134629038<br>C -2.95336589922780 -5.08939742356287 -9.37189420074662<br>H -2.55610570679808 -6.09611530016525 -9.35038305087342<br>C -4.18957772070322 -4.82242843193508 -9.93964086306197<br>H -4.76455048824371 -5.63161114621194 -10.37029927245775<br>C -4.71213326028655 -3.52587188556863 -9.96931940380815<br>H -5.68030455359941 -3.35264442214434 -10.41977090689966<br>C -4.01015879734115 -2.45330388642671 -9.43626137439630<br>H -4.40324567528874 -1.44572945412558 -9.46010372275060<br>C -2.78108335478983 -2.75115685205575 -8.88403216024127<br>C 0.23302930662846 -1.77036647438644 -7.30934890962522<br>C 1.51208300741837 -2.32910790574087 -7.18067151718533<br>H 1.71938700634981 -3.31054123880959 -7.58924347152924<br>C 2.51825983003207 -1.64179338134677 -6.54240575186750<br>H 3.50382194798196 -2.07991574835733 -6.45038291469252<br>C 2.27829170549778 -0.37050065244618 -6.00705570501753<br>C 1.01629267457671 0.20375125983922 -6.14917372050883<br>H 0.79500783215070 1.18605949170171 -5.75567471587402<br>C 0.01038661146367 -0.49360128739312 -6.79903421645561<br>H -0.96403026940359 -0.03097140483679 -6.88149762164476<br>C -0.29134377826384 -3.88267904459024 -2.89240026555553<br>C 0.71168630589192 -3.04390939624840 -3.37853745225879<br>H 1.14044545283232 -3.20504698353044 -4.36085776688787<br>C 1.17011586299758 -1.98825028722708 -2.60680549727219<br>H 1.94967174479302 -1.34388974596087 -2.99200377509259<br>C 0.62565312477508 -1.75802044357810 -1.34975284176762<br>H 0.97950343387919 -0.92969880464163 -0.75002647768514<br>C -0.37623036852294 -2.58690499910801 -0.86552407376379<br>H -0.80462747881566 -2.40847614905593 0.11185139911844<br>C -0.83540517364598 -3.64912316517659 -1.63243172776561<br>H -1.61808930634751 -4.28792268225974 -1.24381114802721<br>C 0.34084604293349 -6.61548711194243 -3.61646214873094<br>C 1.17384316167351 -6.61291950082020 -2.50093572155986<br>H 1.15373093166135 -5.78762278614069 -1.80093204163339<br>C 2.04180514135372 -7.67363099959625 -2.28306857108622<br>H 2.68983175631491 -7.66560905100237 -1.41671857093455<br>C 2.07949650496188 -8.73779461903314 -3.17276989959914<br>H 2.75926285799261 -9.56214511856246 -3.00159918001363<br>C 1.25113515701303 -8.74144979191828 -4.28768973633494<br>H 1.28380940394713 -9.56551101745650 -4.98780267587130<br>C 0.38689595065763 -7.68189907029107 -4.51328484798393<br>H -0.24763867243511 -7.68743249453370 -5.39182839803752<br>C -2.41596771382632 -5.82409051558282 -3.21783816875790<br>C -3.57556019760531 -5.12654950716361 -3.55434535755241<br>H -3.53213639967600 -4.29274530100652 -4.24545347193479<br>C -4.79293450390383 -5.49807397548389 -3.00681894766301<br>H -5.68920006831166 -4.95303335150602 -3.27153605243154<br>C -4.86167326592252 -6.57272478998790 -2.12910612419690<br>H -5.81400064281213 -6.86656532176143 -1.70758551606590<br>C -3.71076773984415 -7.27314352697921 -1.79822242717637<br>H -3.76172693892793 -8.11303229675338 -1.11812097612767<br>C -2.48752537596772 -6.90075245813371 -2.33861494080693<br>H -1.59665150330297 -7.45497210630709 -2.07298276194461<br>C 0.75118668855897 -4.93490055652615 -11.15553388613756<br>C 1.24276732949260 -4.88721000593562 -12.44888978019670<br>C 2.55938760153429 -5.24268249581879 -12.70434860789071<br>C 3.38368789329681 -5.64612465088683 -11.66212880222526<br>C 2.89209894276255 -5.68627747653009 -10.36977760283623<br>C 1.55648654715946 -5.33676400787165 -10.06392725727268<br>H -0.28388548002051 -4.66360584198346 -10.98172055868917<br>H 0.58893204828902 -4.57389310800320 -13.25324200870343	**TS_{anti} (1_{OMe} and 2_{Br})**<br>$E_{PBEh\text{-}3c/CPCM(Chloroform)}$=<br>-4776.43243908<br>$ZPE_{PBEh\text{-}3c/CPCM(Chloroform)}$=<br>0.60379917<br>$FEC_{(298.15)PBEh\text{-}3c/CPCM(Chloroform)}$=<br>0.53406465<br>$E_{PW6B95\text{-}D3(BJ)/def2\text{-}TZVPP/CPCM(Chloroform)}$=<br>-4786.23523092985<br>$E_{B3LYP\text{-}D3(BJ)/def2\text{-}TZVPP/CPCM(Chloroform)}$=<br>-4781.406523008058

	Br 3.23187682119975 -5.18320409470432 -14.47652685507079<br>H 4.41285445807374 -5.92676084231605 -11.84856038166929<br>H 3.54531538766692 -6.00095353013083 -9.56435447198976<br>O 1.11425620263385 -5.38602507102304 -8.84499530182535<br>H 0.03621252070782 -4.61603124992755 -8.51299373007333<br>O 3.30825546509867 0.20956940407798 -5.37807873978478<br>C 3.12771660891014 1.49313317867384 -4.81395597983879<br>H 2.87264948878512 2.23890251572941 -5.57106808670201<br>H 2.35403385999239 1.49262123343451 -4.04169217224799<br>H 4.07678050046686 1.76562934101228 -4.35964662097392	
5	Au 0.34331852407695 -5.74120282030253 -6.91520216320578<br>P -0.19633359750618 -5.68853595926671 -4.73203447907771<br>O -2.80338901650867 -1.82047253498792 -6.90711568925946<br>C -1.72893503393351 -2.34947213032328 -7.55634109551644<br>C -2.11918612951895 -3.30699678106154 -8.43849799641802<br>C -3.54568210050570 -3.39068238176057 -8.33695049628377<br>C -4.54329304917187 -4.16595956244733 -8.93017733384565<br>H -4.29803883730900 -4.91313988698781 -9.67368885589841<br>C -5.85555638815367 -3.95474712924793 -8.54255944621357<br>H -6.64522270406747 -4.54393360134051 -8.99001298283661<br>C -6.18551023550766 -2.99132057020468 -7.58127686557250<br>H -7.22171047077829 -2.85124556744714 -7.30348742964618<br>C -5.20943093037310 -2.21440697536990 -6.97443771412492<br>H -5.45578407437383 -1.47205301183715 -6.22686631435029<br>C -3.90636612132504 -2.44421983620750 -7.37407950588549<br>C -0.42509921192098 -1.82666174026273 -7.19437403148609<br>C 0.71770211403392 -2.23403069400933 -7.89273128024981<br>H 0.63575592788474 -2.92186541601098 -8.72386010217624<br>C 1.96549932623407 -1.75817934805926 -7.55584402925359<br>H 2.83676946712511 -2.08984843191118 -8.10620627207731<br>C 2.11351031536189 -0.84618975527569 -6.50755757672997<br>C 0.98518057869739 -0.42358639646927 -5.81117887194008<br>H 1.05817623608436 0.27549677273169 -4.98998362287854<br>C -0.26521242317705 -0.91536403088631 -6.15285335904005<br>H -1.11994094435657 -0.57705409925558 -5.58301176893321<br>C 0.35608806541155 -4.17048675445875 -3.90378737536331<br>C 1.67778368414547 -3.76518619640667 -4.09052462969160<br>H 2.33372058300827 -4.32181170874139 -4.74992108995037<br>C 2.16152086780592 -2.64936164426550 -3.42931881470040<br>H 3.18911781459150 -2.34354878616759 -3.57504266474726<br>C 1.32671685246249 -1.92127424004881 -2.59094841632630<br>H 1.70391295779032 -1.04484508784532 -2.07987113138894<br>C 0.00718303046063 -2.31023637307654 -2.41832160608711<br>H -0.64826759362469 -1.74047591986618 -1.77296391071893<br>C -0.48043518119218 -3.43537543276512 -3.07059154572067<br>H -1.51065081716567 -3.73214823433256 -2.92346745075945<br>C 0.55629431771166 -7.04226287176238 -3.77992188136362<br>C 1.09572993207094 -6.83833823481526 -2.51297029751048<br>H 1.10610777384667 -5.85031445397445 -2.07130593769950<br>C 1.62875992916294 -7.90747237507182 -1.80564514060028<br>H 2.04954480454984 -7.74197055861516 -0.82268214288561<br>C 1.62251057196821 -9.18068744541011 -2.35622042889560<br>H 2.04067534482006 -10.01180154159783 -1.80365224171155<br>C 1.08343972318859 -9.38763825295690 -3.61975176650019<br>H 1.08007821453249 -10.37830429972381 -4.05454409414915<br>C 0.55543235369330 -8.32271640790106 -4.33190278163230<br>H 0.14393165467720 -8.49307762358312 -5.31994353641003<br>C -1.97769985412581 -5.82334002929253 -4.41203030756014<br>C -2.87355289469709 -5.30049272214203 -5.34131461188013<br>H -2.51430260938000 -4.85268539487033 -6.25907746310063<br>C -4.23885124230642 -5.36136362118705 -5.10630330987562<br>H -4.92446910739404 -4.95431475206945 -5.83838619504751<br>C -4.71606815447965 -5.94794064113248 -3.94235911256053<br>H -5.78151228624937 -6.00106405745901 -3.76039863382904<br>C -3.82678779896679 -6.47411585808080 -3.01447069974808<br>H -4.19625764955182 -6.93657582717375 -2.10876421540425<br>C -2.46047887946312 -6.41395252888603 -3.24577440708389<br>H -1.77758182767450 -6.83247334537949 -2.51739911062509<br>C 1.77569285845125 -5.31295238382210 -10.92620185160057<br>C 2.86627569455411 -4.85890115397918 -11.64608764354921<br>C 4.02213608567529 -4.47487445218544 -10.98010247896769<br>C 4.08247365798052 -4.55038820025849 -9.59738372661111<br>C 2.98781500637473 -5.00665408494496 -8.88011985748757<br>C 1.80095714522015 -5.40254941058531 -9.52154312219769<br>H 0.87715835384650 -5.60744545044279 -11.45468002177514	**PC*anti* (1OMe and 2Br)**<br>$E_{PBEh\text{-}3c/CPCM(Chloroform)}$=<br>-4776.48353981<br>$ZPE_{PBEh\text{-}3c/CPCM(Chloroform)}$=<br>0.60937243<br>$FEC_{(298.15)PBEh\text{-}3c/CPCM(Chloroform)}$=<br>0.54041020<br>$E_{PW6B95\text{-}D3(BJ)/def2\text{-}TZVPP/CPCM(Chloroform)}$=<br>-4786.28580827149

	H 2.80418047336715 -4.80474410237199 -12.72540031702093<br>Br 5.51544605987563 -3.84789573992887 -11.96010649130172<br>H 4.97734573086992 -4.25552320883937 -9.06437473022356<br>H 3.06344381197844 -5.05615700188480 -7.79929797285199<br>O 0.73312149738263 -5.84517421589458 -8.89853943507787<br>H -1.47636931804857 -3.89479351708544 -9.07418291390956<br>O 3.36733078540630 -0.43975552530688 -6.24443678490596<br>C 3.56738329940834 0.52902734445566 -5.23802346073792<br>H 3.02604762329399 1.45569365363244 -5.44703793766180<br>H 3.27047323613363 0.16275622952055 -4.25133752121276<br>H 4.63322077481725 0.74359508593013 -5.22253846691195	
6	Au -0.39360435896672 -2.72169655506155 0.52078731690873<br>P 0.16239845515772 -0.96298259301961 1.81010698343847<br>C 0.55021300420056 0.52704310881534 0.84730039096338<br>C 1.31323679424611 0.39326707331356 -0.31211913359774<br>H 1.63925018930260 -0.58516956273734 -0.64512307901345<br>C 1.65970547287088 1.51423883545959 -1.04946393538455<br>H 2.25147686536527 1.40329765791029 -1.94831336645816<br>C 1.24056180106187 2.77347121021679 -0.63929615246828<br>H 1.50602496186314 3.64741053963203 -1.21956319856530<br>C 0.47719905442732 2.90959377970011 0.51104689504752<br>H 0.14656692006837 3.88855707184419 0.83184020287882<br>C 0.13281274859297 1.79041979011626 1.25657953930816<br>H -0.46237434059821 1.91025244628299 2.15255561050895<br>C 1.62095044213692 -1.26069789855324 2.84962431562696<br>C 2.58125761300397 -0.27479040355673 3.05947770595847<br>H 2.49381557770731 0.69049556154580 2.57786421521533<br>C 3.66433559485895 -0.52775072543254 3.88982541903585<br>H 4.40975124087367 0.24068316422733 4.04618762513092<br>C 3.79075758482680 -1.75966509309499 4.51536237280105<br>H 4.63693853080845 -1.95469712377174 5.16105253539183<br>C 2.83430451353555 -2.74529937175100 4.30781340337776<br>H 2.93187254556887 -3.70896127192361 4.78974137726526<br>C 1.75468484901027 -2.50007980212324 3.47427596920900<br>H 1.01779664710387 -3.27807550907573 3.31277271043418<br>C -1.15758459161220 -0.46657892179306 2.95407128753567<br>C -2.46528389581630 -0.40477406587090 2.47328032495227<br>H -2.68551958952105 -0.67457616011328 1.44699326528737<br>C -3.49360512846408 0.00361971151289 3.30725931390566<br>H -4.50573577721630 0.04999048733552 2.92794082180325<br>C -3.22534508494397 0.34365604448396 4.62736118739353<br>H -4.03027920614771 0.65585771009457 5.27971079394769<br>C -1.92661875653613 0.27769714079686 5.11007357709738<br>H -1.71443740238220 0.53873937580489 6.13838144030302<br>C -0.89161367579231 -0.12422373836240 4.27650468138731<br>H 0.11734367661481 -0.17035207818181 4.66517134078385<br>C -0.97668494875383 -5.90586382668962 -2.29107381679556<br>C -0.41111284954970 -6.47835385963008 -3.41590059207208<br>C 0.76938704030867 -5.96319024102280 -3.93400144791786<br>C 1.37781209712589 -4.87799104127259 -3.32349249783318<br>C 0.80781267624148 -4.30732587421381 -2.19629938756493<br>C -0.38676183940967 -4.80259103870491 -1.64460221828197<br>H -1.89799996579299 -6.31465381120656 -1.89442169045925<br>H -0.89792235004305 -7.32554073510018 -3.88184141844072<br>Br 1.54758738607923 -6.74337933624577 -5.47385329762642<br>H 2.29861089282887 -4.46571995058433 -3.71582523644354<br>H 1.30758081668470 -3.45956553428505 -1.73999504546229<br>O -0.97648824992867 -4.30976485771450 -0.58062772451238	**4-BrPhO-Au-PPh_3**<br>$E_{PBEh\text{-}3c/CPCM(Chloroform)}$=<br>-4048.78438054<br>$ZPE_{PBEh\text{-}3c/CPCM(Chloroform)}$=<br>0.37021440<br>$FEC_{(298.15)PBEh\text{-}3c/CPCM(Chloroform)}$=<br>0.31515138<br>$E_{PW6B95\text{-}D3(BJ)/def2\text{-}TZVPP/CPCM(Chloroform)}$=<br>-4055.76585464599
7	O -1.86291555600898 7.57315079171328 -1.25473476983311<br>C -2.19807314878336 6.29731717802490 -0.91729694251150<br>C -3.06481190726662 5.76988862480015 -1.81973266484319<br>C -3.29644964439232 6.78686451748300 -2.80033397942902<br>C -4.06114376213483 6.89593588003266 -3.96288531676919<br>H -4.67085425820399 6.07066760030285 -4.30710437866310<br>C -4.02029402519846 8.08794787885816 -4.66498055959192<br>H -4.60543581659992 8.19503909904005 -5.56890854140904<br>C -3.23557433648852 9.16291674303017 -4.22993622390326<br>H -3.22717712275702 10.08054727137634 -4.80292318824937<br>C -2.46784180042616 9.07594961460857 -3.07818367102601<br>H -1.85921656522537 9.90258645069843 -2.73645066377385<br>C -2.52349711574877 7.87678971467341 -2.39293132016899<br>C -1.59663536312190 5.75928867256554 0.28945856300273<br>C -1.93779331258571 4.47706373128824 0.73699842906497<br>H -2.65324314469099 3.88037802766355 0.18574229763417<br>C -1.38149240758928 3.94851339948655 1.88007655015711	**2-(4-methoxyphenyl)benzofuran**<br>$E_{PBEh\text{-}3c/CPCM(Chloroform)}$=<br>-727.68133135<br>$ZPE_{PBEh\text{-}3c/CPCM(Chloroform)}$=<br>0.23827959<br>$FEC_{(298.15)PBEh\text{-}3c/CPCM(Chloroform)}$=<br>0.19944599<br>$E_{PW6B95\text{-}D3(BJ)/def2\text{-}TZVPP/CPCM(Chloroform)}$=<br>-730.497410692499

	H -1.65841832640799 2.95605401219960 2.21218832357208<br>C -0.45707820477319 4.68856078557355 2.62417664557164<br>C -0.10545927477538 5.96373936324582 2.19049320880329<br>H 0.60814549904251 6.56771174836436 2.73382671507676<br>C -0.67312345644717 6.48610500529966 1.03693145908249<br>H -0.37785641283284 7.47969306839103 0.72801593403894<br>H -3.48920673825636 4.77922755484651 -1.79954242411422<br>O 0.03139531239503 4.09500534358796 3.72465615973627<br>C 0.95031636659714 4.80651120535308 4.52660922347625<br>H 0.52177160762701 5.73437135629173 4.91486603930098<br>H 1.19611476883419 4.15811114736166 5.36390230032738<br>H 1.86948429521927 5.04288508583908 3.98376706644071	
8	Au -0.78879147292754 -2.22471190014321 -0.74893758884790<br>P -0.09876715354401 -1.13816252169377 1.19283282582839<br>O -1.61527893760290 -4.12159433276971 -4.48990263012045<br>C -0.78765749584390 -3.53550813098528 -3.56265271256714<br>C -1.44022436744662 -3.21983696532928 -2.40543014048151<br>C -2.79540246455366 -3.66972009337953 -2.63670542831943<br>C -3.96786503457114 -3.66619645614482 -1.87845345333398<br>H -3.97370164110593 -3.25748700250617 -0.87476010168036<br>C -5.12116305069071 -4.19387042875745 -2.43408845680229<br>H -6.03861969882718 -4.19969688376945 -1.85991104245191<br>C -5.12464068326869 -4.72238423972523 -3.73060225828674<br>H -6.04170071725715 -5.12790138606799 -4.13736590262567<br>C -3.97374750027393 -4.73721473607764 -4.50511328909664<br>H -3.96997675362010 -5.14481157278845 -5.50762915476968<br>C -2.83536459143536 -4.20452570934096 -3.92703274529648<br>C 0.59006154583077 -3.35862848464298 -4.00608088310514<br>C 1.65166116789872 -3.32442016380690 -3.09431576949785<br>H 1.46779623772043 -3.47102301866611 -2.03852034131207<br>C 2.95144458033046 -3.13309431383861 -3.51297134028660<br>H 3.75705202470485 -3.11427654743624 -2.78972256193550<br>C 3.24355365279446 -2.98380671858574 -4.87067132765758<br>C 2.20302740951488 -3.03611100885793 -5.79403880485643<br>H 2.38329717751122 -2.92928129114485 -6.85496424624574<br>C 0.89847816110507 -3.22314090823701 -5.35882943279912<br>H 0.11209769941640 -3.24717243612414 -6.10151968553641<br>C -0.30782998601815 0.66665540381064 1.17822172861402<br>C -0.00613898513114 1.35666201566563 0.00481781568670<br>H 0.31660847035636 0.81759852656582 -0.87841379984731<br>C -0.11852502267561 2.73755646454334 -0.03955509650632<br>H 0.11843183557472 3.26650911788441 -0.95313967953218<br>C -0.54332092144494 3.43693851962353 1.08281617341080<br>H -0.63777460549216 4.51430713847688 1.04537364261024<br>C -0.85256587944143 2.75304599037998 2.24997527196050<br>H -1.18743368912959 3.29447368036776 3.12489766414280<br>C -0.73372679410715 1.37102631721576 2.30105878363625<br>H -0.97624338127665 0.84851871815254 3.21751577364118<br>C 1.65458251660201 -1.41365905339556 1.58447156732354<br>C 2.47503695030867 -0.40243740035543 2.07618015656880<br>H 2.08864983102341 0.59837682435942 2.21991371105333<br>C 3.80126074634337 -0.67372597895919 2.38452685997503<br>H 4.43529635941155 0.11719953747526 2.76297637124736<br>C 4.31108115475623 -1.95239977514737 2.20934153896694<br>H 5.34512179287176 -2.16069614434958 2.45111876790667<br>C 3.49441508577245 -2.96369238891205 1.71956261157192<br>H 3.88610466054148 -3.96244932748130 1.57839905704858<br>C 2.17271584586122 -2.69531463355237 1.40156593307615<br>H 1.54632100328177 -3.48987810190325 1.01404733352566<br>C -1.00653748799315 -1.71408382452039 2.65894013318618<br>C -2.37848196069786 -1.93562242995556 2.54100369084893<br>H -2.88106021541621 -1.76683214288214 1.59556671033864<br>C -3.10884120432929 -2.37901638117219 3.63217215099783<br>H -4.17246772372415 -2.55100254029274 3.53276655955022<br>C -2.47381387168373 -2.61013860258873 4.84651230227813<br>H -3.04277908641147 -2.96440149014072 5.69611559782289<br>C -1.10904084862560 -2.39300068573842 4.96651828302132<br>H -0.60940588517677 -2.57894440755699 5.90774234705827<br>C -0.37446126968784 -1.94636551792736 3.87634897444211<br>H 0.69165397386561 -1.79223592507320 3.98072037667433<br>C -0.80077682304435 -5.28109967772261 1.01735533051942<br>C -0.53006236740381 -5.19565791828993 2.37534598204140<br>C 0.67316792318309 -5.67689104482505 2.86618230305656<br>C 1.60428829302415 -6.24926085146628 2.01086373373192<br>C 1.33625147181671 -6.32345897087134 0.65434013560803	**RC*syn* (1$_{\mathbf{OMe}}$ and 2$_{\mathbf{Br}}$)**<br>$E_{PBEh-3c/CPCM(Chloroform)}$=<br>-4776.47586269<br>$ZPE_{PBEh-3c/CPCM(Chloroform)}$=<br>0.60931136<br>$FEC_{(298.15)PBEh-3c/CPCM(Chloroform)}$=<br>0.54010256<br>$E_{PW6B95-D3(BJ)/def2-TZVPP/CPCM(Chloroform)}$=<br>-4786.27939925749<br>$E_{B3LYP-D3(BJ)/def2-TZVPP/CPCM(Chloroform)}$=<br>-4781.448083830748

	C 0.13384796897550 -5.83595595828547 0.14414377959084<br>H -1.75314169867280 -4.92145798593114 0.64547120582645<br>H -1.26521364852748 -4.75308163819200 3.03451318926893<br>Br 1.05865373200637 -5.55224366619574 4.71159834132016<br>H 2.54453333390557 -6.62988199839521 2.38724356723615<br>H 2.06549078047783 -6.75877229607787 -0.01703590619108<br>O -0.06925472514014 -5.93742440483281 -1.17951052199627<br>H -0.78362228613762 -5.35173239751331 -1.46766423911258<br>O 4.53800873130353 -2.80420476050408 -5.18749957871321<br>C 4.88591357849243 -2.65967604471031 -6.54729310784191<br>H 5.96517616006369 -2.53130144396564 -6.57879989039787<br>H 4.62056389913322 -3.54305018409353 -7.13468551626377<br>H 4.41527632639583 -1.78261634317540 -7.00012204706644	
9	Au -0.69537715671745 -2.83462272881987 -0.60197555475852<br>P -0.11669147006919 -1.38081241181716 1.07996740354984<br>O -1.64410803850869 -3.19766020165768 -4.22111831860779<br>C -0.74205174482608 -3.65002621181154 -3.33389640947904<br>C -1.33184956479992 -4.15911477143485 -2.17768879394103<br>C -2.75912979039667 -4.00746986530731 -2.44473396613773<br>C -3.91653931647849 -4.32434596381861 -1.73834064370580<br>H -3.85888435502530 -4.79204114510273 -0.76343662781617<br>C -5.14169435887957 -4.02369994580947 -2.31286310960355<br>H -6.05353161945633 -4.26309451475056 -1.78157805632564<br>C -5.22615417041006 -3.41632708502624 -3.56962183022824<br>H -6.19827564621562 -3.19499252545195 -3.98954904429279<br>C -4.08536810024271 -3.09464450159034 -4.29248249066330<br>H -4.14057073019596 -2.62819213376366 -5.26704418506389<br>C -2.88188426177827 -3.41095747471202 -3.69514210541141<br>C 0.64962049124190 -3.56739366274309 -3.73218252577406<br>C 1.62503454780361 -4.27749738162371 -3.01884577224747<br>H 1.33578635221601 -4.91698286679425 -2.19555829990175<br>C 2.95272296784579 -4.19902274252843 -3.36807083130386<br>H 3.69569145997899 -4.75681006365857 -2.81255418069133<br>C 3.35445860860794 -3.40664854341142 -4.44993161077144<br>C 2.39286056294214 -2.70523644580240 -5.17541173670132<br>H 2.66312862805033 -2.08609098091769 -6.01949163911518<br>C 1.05722048239555 -2.79173248161289 -4.81593698631397<br>H 0.33443049010498 -2.23056887740545 -5.39285887456319<br>C -0.55470163832318 0.35220331533952 0.76407591443165<br>C -0.36496650203727 0.86410255605066 -0.51859783753554<br>H 0.00401761042488 0.22560170913342 -1.31297752697998<br>C -0.65107830648143 2.19361309043276 -0.78585671185782<br>H -0.50182520405183 2.58489444843157 -1.78342748159897<br>C -1.13653235851381 3.01678802182327 0.22228440541925<br>H -1.36615658125450 4.05292874650557 0.01093019617821<br>C -1.33251719330351 2.50891210555986 1.49859052044428<br>H -1.71324557781893 3.14674622493930 2.28527051640992<br>C -1.04123354212094 1.17973351302300 1.77259663096705<br>H -1.19683415943547 0.79410046268108 2.77205324155593<br>C 1.66041777586091 -1.37878791882664 1.44173307511636<br>C 2.32199391204098 -0.22698571956421 1.86111273027765<br>H 1.79206193428052 0.71328086886138 1.94439289607368<br>C 3.67262674881861 -0.28038662873698 2.17388250696248<br>H 4.18360054502901 0.61713378019889 2.49645949201564<br>C 4.36479544736068 -1.47991123360417 2.07489625334231<br>H 5.41786477736418 -1.51819809595403 2.32126021516414<br>C 3.70753337988029 -2.62853339778950 1.65503988722194<br>H 4.24239412825164 -3.56554745934189 1.57298560889817<br>C 2.36047116971491 -2.57873496811359 1.33181800579337<br>H 1.86053882638918 -3.48038301850622 0.99907499279715<br>C -0.94004075591690 -1.81729204011084 2.63749165647734<br>C -2.30699945672952 -2.09396654350598 2.60361657157345<br>H -2.85563115929171 -2.03355868443465 1.67050507789837<br>C -2.97105675645079 -2.45706507025780 3.76327186896306<br>H -4.03097619253704 -2.67196240832843 3.73078427268161<br>C -2.27365962078146 -2.55607340704416 4.96201763689614<br>H -2.79086106875508 -2.85044463665684 5.86585215213826<br>C -0.91437428156339 -2.28549733033413 4.99695578816082<br>H -0.36631010000148 -2.36975797357810 5.92583996268623<br>C -0.24546541357381 -1.91448527752427 3.83762254835738<br>H 0.81773112262287 -1.71807335938292 3.87686124757633<br>C -0.81798004790344 -5.52404939855526 1.12974144347169<br>C -0.31106149799022 -5.25375128132407 2.39400609965078<br>C 1.01095781396491 -5.55069494938894 2.68129976791321<br>C 1.83006098826294 -6.11662077977439 1.70985495605571	**TS$_{syn}$ (1_{OMe} and 2_{Br})**<br>$E_{PBEh-3c/CPCM(Chloroform)}$=<br>-4776.44111482<br>$ZPE_{PBEh-3c/CPCM(Chloroform)}$=<br>0.60397849<br>$FEC_{(298.15)PBEh-3c/CPCM(Chloroform)}$=<br>0.53601938<br>$E_{PW6B95-D3(BJ)/def2-TZVPP/CPCM(Chloroform)}$=<br>-4786.2466722327<br>$E_{B3LYP-D3(BJ)/def2-TZVPP/CPCM(Chloroform)}$=<br>-4781.419274468979

	C 1.32538434946458 -6.37102026533239 0.44848671295825<br>C -0.02272670701412 -6.10412338107560 0.11368598111135<br>H -1.86885948819395 -5.33840730989342 0.93667275898375<br>H -0.95796254299527 -4.81657454859473 3.14438815026463<br>Br 1.71802051147779 -5.18212172799321 4.39980682083185<br>H 2.86484059967564 -6.34982650676854 1.92797393077320<br>H 1.97059960140721 -6.81183515494463 -0.30264045825153<br>O -0.48858591857259 -6.35697997263781 -1.07300673227194<br>H -0.91037498670726 -5.23687892379030 -1.62568487383962<br>O 4.66699654828742 -3.38705875828459 -4.71319532000361<br>C 5.13410730035784 -2.61097308316848 -5.79795538996673<br>H 6.21270135772357 -2.74208166286087 -5.82802574945712<br>H 4.71224107770934 -2.94468120039653 -6.74945238279161<br>H 4.91200033976212 -1.54905713722889 -5.66489908806944	
10	Au -0.78103094365061 -3.06852705779034 -0.58058533601100<br>P -0.19749613629560 -1.52938229767540 1.01914155570555<br>O -1.53197226175997 -2.68238936684696 -3.76648584911624<br>C -0.76120085479291 -3.58141418794315 -3.13207190649853<br>C -1.51411545631857 -4.37133510158722 -2.25799310878919<br>C -2.88427242791984 -3.92386797705384 -2.45629140426553<br>C -4.12052664410418 -4.30022486857159 -1.93995784256315<br>H -4.20110340815581 -5.11165202883739 -1.22864027997639<br>C -5.24130292954646 -3.60346043110934 -2.36178035156950<br>H -6.21500566119113 -3.87871429017735 -1.97888865942574<br>C -5.14209506215691 -2.54760981814821 -3.27344154990445<br>H -6.03784843881267 -2.02364187888099 -3.57866275815347<br>C -3.91811279787336 -2.15897785536175 -3.79942069952310<br>H -3.83315894805163 -1.34641866492847 -4.50827886781582<br>C -2.82067231327739 -2.87828774803973 -3.37252723039701<br>C 0.64017243127645 -3.63531830451754 -3.49801245055382<br>C 1.42512665993846 -4.72001720526743 -3.08428300367025<br>H 0.98054302039204 -5.53286135583876 -2.52127802144012<br>C 2.76187803521301 -4.77734072777682 -3.40233241811355<br>H 3.36090291641986 -5.62008404720868 -3.08186372299473<br>C 3.35848594041531 -3.75291294585708 -4.14658919107038<br>C 2.58244465480555 -2.67394829668371 -4.57023965243083<br>H 3.00724221193858 -1.86562791562691 -5.14888009214650<br>C 1.23695670662700 -2.62284049770628 -4.24649445946933<br>H 0.65967488681622 -1.77129693848596 -4.58112901612826<br>C -0.77490881969971 0.14356444510686 0.60702107045270<br>C -0.60347424417003 0.60159787321276 -0.69896058068417<br>H -0.16174139256905 -0.04192409986623 -1.45132346976184<br>C -0.99930431161460 1.88362876771398 -1.04347261263817<br>H -0.86311030399301 2.23300017301040 -2.05833411178505<br>C -1.57754890729345 2.71322615842881 -0.09036772556656<br>H -1.89289421805560 3.71200043587655 -0.36234151109164<br>C -1.75454397609778 2.25872865126759 1.20798538347734<br>H -2.20603826799750 2.90104270978665 1.95243539683279<br>C -1.35309965648626 0.97683507937922 1.56002999552420<br>H -1.49465422846415 0.63415976776643 2.57688172036379<br>C 1.59310816557539 -1.37616460594057 1.24518746182117<br>C 2.15667208161263 -0.19071370305003 1.71620857932256<br>H 1.53305111811006 0.66824043826473 1.93045944849318<br>C 3.52646378081352 -0.10556585478880 1.91047717088278<br>H 3.96000723668769 0.81594470364125 2.27578552210067<br>C 4.33837811995904 -1.19878436592072 1.63499810300571<br>H 5.40764671195932 -1.12903294983143 1.78635769103634<br>C 3.78011002254054 -2.37735377981637 1.16164030715977<br>H 4.40839112935060 -3.23049682069284 0.94261584991444<br>C 2.41041737659489 -2.46750915884575 0.96177880007720<br>H 1.98961276858729 -3.39292382532620 0.58816312017765<br>C -0.91420122854060 -1.90784363470843 2.63943397443752<br>C -2.26100732227483 -2.26915152577089 2.69297683757581<br>H -2.85418208702903 -2.31411916813948 1.78647272213272<br>C -2.84737057641521 -2.58114744060498 3.90763469424771<br>H -3.89157982840703 -2.86197336871837 3.94393605972181<br>C -2.09142569269309 -2.54476059239135 5.07410051459977<br>H -2.54739165755065 -2.79932347361333 6.02187529396019<br>C -0.75222749781900 -2.19098825683813 5.02152504747119<br>H -0.15874706039903 -2.17077342838208 5.92576365160824<br>C -0.16097843293844 -1.86966829495077 3.80650615975826<br>H 0.88787886182271 -1.60689683495184 3.77787945913232<br>C -0.62787821640023 -5.68861058121414 1.05645017668021<br>C -0.05729248107648 -5.24891762943192 2.24487692445216<br>C 1.30578022248083 -5.38316107011364 2.45173710151193	**PC*syn* (1_{OMe} and 2_{Br})**<br>$E_{PBEh\text{-}3c/CPCM(Chloroform)}$=<br>-4776.44738891<br>$ZPE_{PBEh\text{-}3c/CPCM(Chloroform)}$=<br>0.60837309<br>$FEC_{(298.15)PBEh\text{-}3c/CPCM(Chloroform)}$=<br>0.53966167<br>$E_{PW6B95\text{-}D3(BJ)/def2\text{-}TZVPP/CPCM(Chloroform)}$=<br>-4786.25610387226

	C 2.10574230215484 -5.95221753799483 1.46241073533828<br>C 1.54270783976086 -6.39281525736702 0.28350128273097<br>C 0.13530909024285 -6.33127308878390 0.02964167768699<br>H -1.70735580063861 -5.64262153992212 0.94864767598486<br>H -0.69105845015492 -4.81086657033845 3.00648753328516<br>Br 2.09591440858695 -4.80883158101878 4.07841961282674<br>H 3.17459853940301 -6.05075513238098 1.61125758387437<br>H 2.18013342899323 -6.84881324828309 -0.46626763843285<br>O -0.38565987845780 -6.80067309573216 -1.02888230431990<br>H -1.20352966039509 -5.36506781571327 -1.88504857234302<br>O 4.66377395467819 -3.89302686995006 -4.40368951828389<br>C 5.32521336423642 -2.89362184592837 -5.15323700606712<br>H 6.36145831310771 -3.20993660723093 -5.23974016955833<br>H 4.90354563270721 -2.79066823354791 -6.15630878275006<br>H 5.29384654235502 -1.92210696669465 -4.65348612195744	
11	Au 0.22271314945735 -4.05436728081541 -2.23669777680458<br>P 0.93573384393028 -3.76361790501079 -0.03482558546050<br>O -0.97587464667563 -3.77563101000594 -6.31963980243486<br>C -0.33806398129586 -3.34430143695909 -5.17980415024261<br>C -0.41442824104476 -4.24620390721100 -4.16101162832046<br>C -1.15872838222375 -5.34723208679940 -4.72779794452393<br>C -1.59332047201666 -6.58327015562111 -4.24571077312118<br>H -1.37117734719482 -6.88661676519379 -3.22956610806771<br>C -2.31132087949878 -7.41291345971212 -5.09020415475902<br>H -2.65422065127710 -8.37512600879023 -4.73227932102543<br>C -2.60445800510357 -7.02995742415287 -6.40486704189502<br>H -3.16741009427912 -7.70003939085128 -7.04135192865516<br>C -2.18753453748398 -5.80632800145219 -6.90775446229698<br>H -2.41201011114905 -5.50391491601302 -7.92237834040372<br>C -1.47272919908943 -4.99543523886472 -6.04364339602336<br>C 0.24971931608093 -2.01197969447299 -5.27460423777478<br>C 1.41577069606972 -1.68744997301748 -4.57838356413474<br>H 1.93182852522162 -2.44920719562912 -4.00854234240584<br>C 1.94760887352409 -0.41037823085300 -4.63552335745609<br>H 2.85707109939294 -0.19267818135736 -4.08697589051718<br>C 1.34962519560195 0.58890924374041 -5.40035812296250<br>C 0.20468830353178 0.25493443802366 -6.11899225784447<br>H -0.27766703825248 1.00578550802280 -6.73386496841020<br>C -0.33794623446248 -1.01901220494966 -6.06189851857635<br>H -1.23711970303088 -1.23241944250170 -6.62515996231595<br>C 1.29832589940008 -2.02122288448113 0.34241439046202<br>C 1.98611983409471 -1.27075934884074 -0.61101525168505<br>H 2.28033871195310 -1.71931293764272 -1.55242501129542<br>C 2.29724627945452 0.05651695442664 -0.36261699707653<br>H 2.82831735438494 0.63157351079259 -1.10976535971634<br>C 1.91754130871559 0.64682408004825 0.83616329532775<br>H 2.15401006459792 1.68537579876406 1.02726195169525<br>C 1.22928223302320 -0.09466125417168 1.78558552410503<br>H 0.92913273145990 0.36230194659916 2.71933479243093<br>C 0.91989911565878 -1.42620093643629 1.54240036010064<br>H 0.38131689247995 -1.99282443776148 2.29096257636659<br>C 2.45894316048675 -4.66401252560395 0.38243486910519<br>C 3.45583926207272 -4.10419008280661 1.17686006764776<br>H 3.35620251727039 -3.09388461468289 1.55246828597822<br>C 4.58933575336419 -4.84123053284171 1.49119284699794<br>H 5.36188240346209 -4.39913082638217 2.10643711497828<br>C 4.73127882462068 -6.13817258694301 1.01868331439874<br>H 5.61654572937694 -6.71014379793456 1.26404929781093<br>C 3.73915120657943 -6.69995859931020 0.22536658126887<br>H 3.84813093642636 -7.70896240384720 -0.14965966920449<br>C 2.60910733028960 -5.96471091652724 -0.09691177773160<br>H 1.84417066741335 -6.40741140058686 -0.72427921948940<br>C -0.27507155339563 -4.27853096145166 1.21977394114816<br>C -1.62148089425454 -3.99510247182634 0.99273931363556<br>H -1.92988539009052 -3.50275124345682 0.07776534334536<br>C -2.57531239296018 -4.34203704964036 1.93674145021788<br>H -3.61786029772024 -4.11800551480791 1.75380504189946<br>C -2.19341134444318 -4.98350680086588 3.10806379938444<br>H -2.93941155069859 -5.26046570074653 3.84142558111659<br>C -0.85571087687537 -5.27447920444562 3.33417577419170<br>H -0.55476159553926 -5.77751669711021 4.24366387796250<br>C 0.10359680596359 -4.92221492646549 2.39472261361732<br>H 1.14377347019295 -5.15433807647748 2.58350675291935<br>C 1.91213679049896 1.97917962276941 -5.43324352685498<br>H 2.99952748420596 1.97331088131058 -5.35893298334391	**1_{Me}**<br>$E_{PBEh-3c/CPCM(Chloroform)}$=<br>-1822.19032040<br>$ZPE_{PBEh-3c/CPCM(Chloroform)}$=<br>0.50537825<br>$FEC_{(298.15)PBEh-3c/CPCM(Chloroform)}$=<br>0.44582386<br>$E_{PW6B95-D3(BJ)/def2-TZVPP/CPCM(Chloroform)}$=<br>-1828.25317881523

	H 1.63955396818712 2.50104423648848 -6.34994626190665<br>H 1.53523842561133 2.57309787634324 -4.59767306837540	
12	Au 0.34894029595342 -4.06044925340434 -2.24374672012007<br>P 1.04157796226469 -3.81004476203079 -0.03557865538450<br>O -0.74606568343378 -3.79894221870579 -6.36923194632515<br>C -0.32821113897238 -3.27830168770225 -5.16699508721009<br>C -0.16633269614161 -4.23891096176286 -4.20965657950605<br>C -0.50963873737971 -5.46736520252229 -4.88989730126795<br>C -0.55908364962221 -6.81065158323145 -4.51095446101837<br>H -0.30251461705941 -7.10772828390576 -3.50092305027113<br>C -0.93876217785898 -7.75349358985783 -5.45105840430589<br>H -0.98117218569792 -8.79908665711157 -5.17471572890770<br>C -1.27261597372285 -7.37921908695910 -6.75855374706605<br>H -1.56611661703009 -8.13917146780874 -7.47077116369259<br>C -1.23603790757125 -6.05180568764580 -7.15913782210714<br>H -1.49435312055984 -5.75633178951162 -8.16767557654267<br>C -0.85353472396823 -5.12955904421691 -6.20085742605272<br>C -0.19206360396236 -1.82427572427167 -5.15567030652368<br>C 0.69330627877703 -1.17879039068559 -4.28737074124535<br>H 1.33656782580649 -1.75523735510565 -3.63645084251498<br>C 0.78917630226555 0.20061299267175 -4.26245479304324<br>H 1.48673802720421 0.66600016344948 -3.57536642192008<br>C 0.02041243823935 0.99644805302416 -5.11083391580525<br>C -0.84661023845348 0.35292745746459 -5.98765622710427<br>H -1.45817517575229 0.94121033422734 -6.66147790535909<br>C -0.95343043423780 -1.02950196518977 -6.01534157636726<br>H -1.65196451888313 -1.48502546261195 -6.70458394060794<br>C 1.91360110502654 -2.23255193676516 0.21131708450073<br>C 2.83448237220441 -1.83052120696026 -0.75684809675533<br>H 3.01407583141154 -2.44309724919204 -1.63291594272565<br>C 3.52866942581259 -0.64061326396340 -0.60594713817478<br>H 4.24194030748485 -0.33670810983411 -1.36072163042855<br>C 3.30263494909752 0.16115298454381 0.50607984020521<br>H 3.83988204668999 1.09366016587058 0.61949625510932<br>C 2.38421828830851 -0.23275090514745 1.46801900596981<br>H 2.20334277052436 0.38984612523107 2.33429588716334<br>C 1.69110497599188 -1.42751433378064 1.32427342299416<br>H 0.97682748362880 -1.72250374701204 2.08203153510321<br>C 2.21105187701391 -5.09093943448743 0.50314672154963<br>C 3.34407483753295 -4.79407645972977 1.25474593599741<br>H 3.57144567457593 -3.77100291938335 1.52548574086737<br>C 4.19407981240048 -5.81445218549933 1.65994250767771<br>H 5.07575878019479 -5.57750299850681 2.24086742284406<br>C 3.91530564981010 -7.13043880727821 1.32130027007253<br>H 4.58210389491667 -7.92346928178556 1.63313515179230<br>C 2.78530240946545 -7.42947946844726 0.57138738988735<br>H 2.57068717830328 -8.45333080729822 0.29643802733970<br>C 1.93867850836670 -6.41433132766964 0.15814801910754<br>H 1.06664340253409 -6.65516117480859 -0.43881220557147<br>C -0.28425091236845 -3.82367586089401 1.20673808177102<br>C -1.49761864757240 -3.21547275000681 0.88846337612139<br>H -1.64693871122337 -2.77287960104177 -0.08952038043557<br>C -2.52207991494520 -3.17425620199692 1.82159228702815<br>H -3.46078882884777 -2.70000591103466 1.56759453765741<br>C -2.34542184035064 -3.74851694811592 3.07365694349061<br>H -3.14786472882750 -3.72206007966573 3.79921615090465<br>C -1.14178127304501 -4.36187465614650 3.39170163047571<br>H -1.00241240551336 -4.81388690925749 4.36485847986592<br>C -0.11133095830427 -4.39940244064712 2.46278899326907<br>H 0.82276141449236 -4.88116943392545 2.72198665535432<br>C 3.03641709525545 -6.30390603311217 -3.87550135753027<br>C 3.48680296610993 -7.50179165747253 -3.34170705596149<br>C 4.55732944265750 -7.50209143819852 -2.46260215425942<br>C 5.17443211039128 -6.31239441549854 -2.10478408083118<br>C 4.72140962615495 -5.11691516519369 -2.63706655579193<br>C 3.65513675993143 -5.10465501838835 -3.53308545882857<br>H 2.20274320352845 -6.31491591449404 -4.56770176752831<br>H 2.99510351765705 -8.42322156912563 -3.62423738611560<br>Br 5.18593248022640 -9.13542456384429 -1.75006211593859<br>H 6.00318107554896 -6.30326461821915 -1.40985772886465<br>H 5.20069034391653 -4.18722981911261 -2.35746695510580<br>O 3.27178462797405 -3.91724780651995 -4.03835403554711<br>H 2.44009516308510 -4.00680222544273 -4.52346639469140<br>C 0.14503014152527 2.49101850953307 -5.08538157073358<br>H 1.12241233005032 2.81141406881482 -5.45115376144967	**PC*anti* (1$_{Me}$ and 2$_{Br}$)**<br>$E_{PBEh-3c/CPCM(Chloroform)}$=<br>-4701.42443531<br>$ZPE_{PBEh-3c/CPCM(Chloroform)}$=<br>0.60378976<br>$FEC_{(298.15)PBEh-3c/CPCM(Chloroform)}$=<br>0.53505124<br>$E_{PW6B95-D3(BJ)/def2-TZVPP/CPCM(Chloroform)}$=<br>-4710.95366091563

	H -0.61145396980567 2.96642741837628 -5.70821728605428<br>H 0.03957953939812 2.88185576592367 -4.07253500673049	
13	Au 0.67618989189581 -4.28169583958248 -1.76020096746377<br>P 0.84272158562300 -3.37991539843151 0.33901078567667<br>O -1.05694299127755 -3.87094222787232 -4.95300846631071<br>C 0.17139164190152 -3.79303741799238 -4.42128823630444<br>C 0.57173929672038 -4.98249820519104 -3.81483263687334<br>C -0.55785477522447 -5.87247418488463 -4.05451481840895<br>C -0.82222048025007 -7.20700755588687 -3.75774652866669<br>H -0.09608932110168 -7.81064808097987 -3.22860080446775<br>C -2.03757694099943 -7.73958727100681 -4.15732631230640<br>H -2.26380537612073 -8.77499395995131 -3.93934361665339<br>C -2.98184443420523 -6.96644565692875 -4.84075678336148<br>H -3.92076492240820 -7.41413835152078 -5.13788100595343<br>C -2.73692119353289 -5.63611683728483 -5.15109648825268<br>H -3.45846061803580 -5.03222737323896 -5.68484078201182<br>C -1.51596774339850 -5.13683449947994 -4.74429681152591<br>C 0.86531424249526 -2.52177479167912 -4.56315390798465<br>C 2.24900840811453 -2.46252955891194 -4.36711879165044<br>H 2.80683544598454 -3.36667931780950 -4.15605917085349<br>C 2.91258964261197 -1.25459453126501 -4.45131461375898<br>H 3.98537390268468 -1.23038476847894 -4.30154727721855<br>C 2.22795327464563 -0.06964551010381 -4.73018095580682<br>C 0.85408220443657 -0.14094609403551 -4.93898339905083<br>H 0.29979379345040 0.76223240641485 -5.16299078061617<br>C 0.17626293489336 -1.34738992761258 -4.86226821869027<br>H -0.89396741921075 -1.36067959111625 -5.01992157183751<br>C 0.73267428152214 -1.56796892138233 0.25644647682364<br>C 1.36956704635206 -0.90631394356068 -0.79373917191961<br>H 1.90306759878934 -1.46192994723206 -1.55667318404233<br>C 1.32250701636485 0.47621581672458 -0.87354011390977<br>H 1.81735093300760 0.98156538080179 -1.69262546582888<br>C 0.63508428737203 1.20618942644847 0.08780059828704<br>H 0.59310101453009 2.28539672199780 0.02033904267857<br>C -0.00324092495015 0.55077922604893 1.13086642124360<br>H -0.54228080684611 1.11583880731502 1.87968558960147<br>C 0.04430502568332 -0.83385394588895 1.21837646761945<br>H -0.45944946097651 -1.33350378709251 2.03585836036577<br>C 2.41717078922725 -3.74447531089517 1.16174809305514<br>C 3.08107336960236 -2.78481302728561 1.92151806967678<br>H 2.68076362459423 -1.78355384340178 2.01570136864493<br>C 4.26886876706593 -3.10940750243333 2.56144496454996<br>H 4.78328003124287 -2.35965311914772 3.14780178550319<br>C 4.79514628741296 -4.38842915530029 2.44787448314357<br>H 5.72308642830995 -4.63776633093668 2.94557098117358<br>C 4.13555476392113 -5.34686301119336 1.68935952804849<br>H 4.54685811384288 -6.34271439463122 1.59243716417550<br>C 2.95196151255355 -5.02647489707838 1.04336037244023<br>H 2.45018192053241 -5.77739158945692 0.44432066943058<br>C -0.45670576023286 -3.90199944069705 1.49175939292698<br>C -1.76673964322798 -3.98635056830886 1.02134417561713<br>H -1.99113951642588 -3.77097147068576 -0.01692295133787<br>C -2.79109484232927 -4.34814333371536 1.88124935468873<br>H -3.80565594809424 -4.41128524674552 1.51097543908884<br>C -2.51296244716554 -4.63776913927674 3.21132217737292<br>H -3.31275272930892 -4.92696915734394 3.88036351681458<br>C -1.20945752885339 -4.56254669756610 3.67993496015561<br>H -0.98900366868131 -4.79154050409819 4.71409359358911<br>C -0.18032145355297 -4.19361187807992 2.82431756780849<br>H 0.83247327696713 -4.13801982117275 3.20178884651088<br>C 2.74393832906013 -7.00250627907482 -5.82290984676025<br>C 3.28862177984094 -7.73174984989687 -6.86588263028267<br>C 4.64605491473868 -8.01902542243947 -6.87808531608837<br>C 5.45737280356206 -7.57515966561653 -5.84233438282004<br>C 4.91164393263525 -6.84358868306155 -4.80284809512565<br>C 3.53375395963493 -6.52896685936115 -4.74853024839282<br>H 1.67994073891813 -6.79601733490320 -5.83388451704585<br>H 2.64426441442175 -8.07364761449303 -7.66624593358896<br>Br 5.39200952411597 -9.02039782080598 -8.30537798520103<br>H 6.51815822155108 -7.79301576109371 -5.83896601275815<br>H 5.55534889410105 -6.50181286207171 -4.00082764548212<br>O 3.04455495623558 -5.84413067103279 -3.76170631690118<br>H 1.75154053892437 -5.40014091047246 -3.81927232883550<br>C 2.95917486170904 1.23725820438599 -4.78846787617611<br>H 3.84151934389043 1.17014696844341 -5.42562626825050	**TS$_{anti}$ (1$_{Me}$ and 2$_{Br}$)**<br>E$_{PBEh-3c/CPCM(Chloroform)}$=<br>-4701.38101595<br>ZPE$_{PBEh-3c/CPCM(Chloroform)}$=<br>0.59828364<br>FEC$_{(298.15)PBEh-3c/CPCM(Chloroform)}$=<br>0.52911163<br>E$_{PW6B95-D3(BJ)/def2-TZVPP/CPCM(Chloroform)}$=<br>-4710.90977739208

	H 2.32583271425729 2.03587291711988 -5.17139467807175<br>H 3.30435138946183 1.53476504647526 -3.79608124186347	
14	Au 1.80939144782773 -5.02364990634380 -1.86810474694485<br>P 1.03765058339516 -4.22984827111015 0.09175540629746<br>O -0.61298939965554 -2.56654460104986 -3.53361304097504<br>C 0.00893412408910 -3.30761290654135 -4.49176362417579<br>C -0.63310072240805 -4.48910758489770 -4.68121269043953<br>C -1.74417277977685 -4.49440310619479 -3.77892178246274<br>C -2.78390469355756 -5.37761416256644 -3.47902145224223<br>H -2.87060424637335 -6.32814413080983 -3.98923991624339<br>C -3.70480200601121 -5.00267522784204 -2.51586932316789<br>H -4.52090084234573 -5.66978860690700 -2.27026183264277<br>C -3.60177300208164 -3.77663785329393 -1.84653317027289<br>H -4.33521721729426 -3.51932578424709 -1.09400911449007<br>C -2.57620781026229 -2.88649086405803 -2.12607781562852<br>H -2.48654140427030 -1.93914076217540 -1.61125773497039<br>C -1.67199317951325 -3.27726124307173 -3.09557542513106<br>C 1.19259299901122 -2.72708637797437 -5.10102332767816<br>C 1.83987225913818 -3.38756561684599 -6.14771773105647<br>H 1.46124116472005 -4.33441023707771 -6.50988061843223<br>C 2.96819992582482 -2.84838318596402 -6.73352689806461<br>H 3.45371424367711 -3.38634642342161 -7.53809890254879<br>C 3.49811385129233 -1.63480992569479 -6.29923823292709<br>C 2.84832255367388 -0.97656758448454 -5.25962817897946<br>H 3.23326934539257 -0.02767919159549 -4.90487557481881<br>C 1.71383989779348 -1.50738231154627 -4.66651469341390<br>H 1.23507389980668 -0.95857083281528 -3.86643751367533<br>C 0.30303418400022 -2.57183358091828 0.04733478910385<br>C 0.85243054068916 -1.61418910405661 -0.80118416374294<br>H 1.67628734315569 -1.87162507235600 -1.45603236581062<br>C 0.34273928910036 -0.32514414343923 -0.81688611721241<br>H 0.77118249060234 0.41338721091428 -1.48169776258977<br>C -0.71951313824163 0.01260707865624 0.01102616427528<br>H -1.12032052973803 1.01771437571373 -0.00608057943245<br>C -1.27095868623868 -0.94028490185718 0.85685552892630<br>H -2.10107322409797 -0.68116917596563 1.50067096587762<br>C -0.76278453903751 -2.23094065009158 0.87661253363821<br>H -1.20481447406668 -2.96725884034360 1.53541187941868<br>C 2.35819468821895 -4.13259059177623 1.33846759504887<br>C 2.49958022661805 -3.03044704806103 2.17612402174017<br>H 1.83044129480476 -2.18435381209348 2.09114894228589<br>C 3.50865930512343 -3.00760502377151 3.12982889818487<br>H 3.61512823935955 -2.14538631804125 3.77473772551343<br>C 4.37480066244731 -4.08337225763707 3.25507075472381<br>H 5.16072984589119 -4.06272958917350 3.99861704652963<br>C 4.23610773136660 -5.18590396866089 2.42103043292531<br>H 4.91177572815584 -6.02595431295573 2.51206344409600<br>C 3.23609591313509 -5.20964688721612 1.46253664544514<br>H 3.14203895221663 -6.07192252556048 0.81267511383247<br>C -0.23485920383504 -5.29702264959409 0.82549091842498<br>C -1.25833544929753 -5.76326647231610 0.00220315554100<br>H -1.27330116265728 -5.51541127319448 -1.05272977395852<br>C -2.27050036971131 -6.54793866698704 0.53118785457375<br>H -3.06265300211982 -6.90340928262964 -0.11424259242319<br>C -2.26250616494707 -6.87893279661881 1.88008549948601<br>H -3.04997317496030 -7.49762623783731 2.29025202695518<br>C -1.24263862501625 -6.41909911420507 2.70110341583752<br>H -1.23249725836001 -6.67608700380123 3.75208207321670<br>C -0.23016531305946 -5.62650338282710 2.17816741340095<br>H 0.55860330819311 -5.27188224884931 2.82892620642913<br>C 3.82703364832911 -6.24421064060811 -5.44941599141973<br>C 4.89768505312442 -5.91118356603160 -6.25882872926082<br>C 5.73192513965696 -4.86212205237808 -5.90003062021057<br>C 5.49695496529080 -4.15751005373107 -4.73013333822315<br>C 4.42546884369118 -4.49627754995714 -3.92149189756647<br>C 3.55145313780284 -5.54330142880100 -4.25984410946943<br>H 3.17842291715718 -7.06267823243371 -5.73770035606762<br>H 5.07200881406881 -6.47314671279291 -7.16761549136794<br>Br 7.18980035584696 -4.39095542749618 -7.01536571407130<br>H 6.14031257366924 -3.33707722570564 -4.43866924915206<br>H 4.26072405639167 -3.92165342845627 -3.01639402458322<br>O 2.51275590490929 -5.90603438355363 -3.54485078460211<br>H -0.34907775204071 -5.26956551460040 -5.36802952988378<br>C 4.74928791366055 -1.07789652262426 -6.90778818013139<br>H 4.82988284540157 -1.33196850968470 -7.96432339086525	**PC*anti* (1$_{Me}$ and 2$_{Br}$)**<br>$E_{PBEh\text{-}3c/CPCM(Chloroform)}$=<br>-4701.43457565<br>$ZPE_{PBEh\text{-}3c/CPCM(Chloroform)}$=<br>0.60387410<br>$FEC_{(298.15)PBEh\text{-}3c/CPCM(Chloroform)}$=<br>0.53626018<br>$E_{PW6B95\text{-}D3(BJ)/def2\text{-}TZVPP/CPCM(Chloroform)}$=<br>-4710.96298875757

	H 4.79464958810146 0.00673473793468 -6.81381439782617<br>H 5.63330586239516 -1.48626525193648 -6.41280472410746	
15	O -1.19208326524128 3.96027849242006 -5.60748069217590<br>C -1.36378605175046 2.62453876360438 -5.40757919227363<br>C -2.64097261445545 2.35072696922430 -5.03759230113802<br>C -3.32927278780270 3.60524684507843 -5.00135688537083<br>C -4.62866539519607 4.02155960399987 -4.70643244315135<br>H -5.39073373846990 3.30750030084180 -4.42250863082338<br>C -4.91748042774436 5.37231712461566 -4.78664177050948<br>H -5.91793115799928 5.71845213740062 -4.56267236621548<br>C -3.93963165510545 6.30571297396600 -5.15302760124904<br>H -4.20025754967124 7.35440603672126 -5.20581514151676<br>C -2.64314397465100 5.91425471666561 -5.45018082097159<br>H -1.88330594497669 6.63023109258182 -5.73392150647228<br>C -2.37651215697205 4.56030276173179 -5.36372143466112<br>C -0.20365922366156 1.77486838784910 -5.61655551535660<br>C -0.30862796562739 0.39000526275186 -5.45208978659333<br>H -1.25475407667264 -0.05433518678182 -5.17042847129243<br>C 0.78556743687861 -0.42947243816003 -5.64672004167828<br>H 0.67501850079059 -1.49908845824712 -5.51229841289812<br>C 2.02677340936520 0.09391796282648 -6.01307152216635<br>C 2.12594817566861 1.47103848046303 -6.17610978552206<br>H 3.07598289454787 1.90759668461834 -6.46002410360353<br>C 1.03373140690619 2.30373809411897 -5.98275831698805<br>H 1.15753657096353 3.36941582052055 -6.12012898976064<br>H -3.05126232880313 1.37910704123228 -4.81498500698090<br>C 3.20592876143569 -0.80882233606386 -6.22115228116279<br>H 4.09663316547738 -0.24706269866328 -6.49824510471542<br>H 3.01178624323250 -1.53665846001790 -7.01051730558407<br>H 3.43586571453447 -1.37328243629822 -5.31614753716854	**2-(*p*-tolyl)benzofuran**<br>$E_{PBEh\text{-}3c/CPCM(Chloroform)}$=<br>-652.63079945<br>$ZPE_{PBEh\text{-}3c/CPCM(Chloroform)}$=<br>0.23273742<br>$FEC_{(298.15)PBEh\text{-}3c/CPCM(Chloroform)}$=<br>0.19444162<br>$E_{PW6B95\text{-}D3(BJ)/def2\text{-}TZVPP/CPCM(Chloroform)}$=<br>-655.172951464994
16	Au -2.19449699069069 -3.99094229328052 -1.93093347002590<br>P -2.23295672021096 -3.17475894997642 0.25063228559338<br>O -1.90712751530614 -5.28888705824913 -5.99887374407165<br>C -1.42317230140693 -4.67261501123851 -4.87001339544631<br>C -2.27060841402224 -4.78935692417040 -3.80606159408820<br>C -3.37558229064650 -5.56496322309870 -4.32650511260533<br>C -4.56738056442444 -6.05049507693331 -3.78426751389908<br>H -4.82001730465132 -5.85900501526462 -2.74785764547080<br>C -5.41846519317612 -6.78034881171680 -4.59647211374122<br>H -6.34601639056897 -7.16505280700681 -4.19268614925574<br>C -5.10180607234187 -7.03206092156060 -5.93704651857720<br>H -5.78737904206880 -7.60598543091775 -6.54654337961867<br>C -3.92533047494534 -6.56026890382353 -6.50038761401995<br>H -3.67514255141702 -6.75183864400100 -7.53565903871287<br>C -3.09240273975949 -5.83350387018229 -5.66809072256034<br>C -0.13445971136147 -4.01173515593696 -5.04549597259647<br>C 0.74171265866164 -3.83453183753647 -3.97073524451117<br>H 0.48922167669853 -4.22496108705332 -2.99393017034455<br>C 1.95172820521873 -3.18671819321791 -4.13995274426013<br>H 2.60920555870587 -3.06869668210175 -3.28633588537152<br>C 2.34727928626407 -2.70223942113721 -5.38642071735112<br>C 1.48263524642677 -2.89634130854073 -6.45837156214581<br>H 1.76079508525616 -2.53490858840571 -7.44128168824404<br>C 0.26500156683149 -3.54039886614588 -6.29745005601056<br>H -0.38093647684623 -3.65941864058910 -7.15726663157398<br>C -2.90012291148222 -1.49394591142195 0.42314886578603<br>C -2.53709601280661 -0.53947141958918 -0.52608823477227<br>H -1.89593270139284 -0.81156975380540 -1.35661114217378<br>C -2.99551775130369 0.76382348365234 -0.41361729515322<br>H -2.70918948434396 1.49957602664858 -1.15338636103576<br>C -3.82758954368361 1.11946747629130 0.64016358484963<br>H -4.19110408057100 2.13533423507203 0.72342255864109<br>C -4.19745991012830 0.17039303277367 1.58255820133730<br>H -4.84839765572752 0.44345548152410 2.40261871703447<br>C -3.73459780740875 -1.13400632184678 1.47779977065073<br>H -4.02874404136663 -1.86568742888839 2.21937402737692<br>C -0.60946269670155 -3.13155611807652 1.06488342387763<br>C -0.22892435684291 -2.09309940178786 1.91037627229463<br>H -0.89180137752550 -1.25566148722983 2.08613936499772<br>C 1.01076503679269 -2.12609403221386 2.53429601854318<br>H 1.30276016333717 -1.31477582091779 3.18797116812991<br>C 1.87095318234966 -3.19433878659422 2.32191468465088<br>H 2.83627463880176 -3.21726269597463 2.81056885261370<br>C 1.49363659289027 -4.23182865591164 1.47913289476962<br>H 2.16025540109080 -5.06669634764029 1.30736795488381	**PC*syn* (1_{Me} and 2_{Br})**<br>$E_{PBEh\text{-}3c/CPCM(Chloroform)}$=<br>-4701.42541367<br>$ZPE_{PBEh\text{-}3c/CPCM(Chloroform)}$=<br>0.60371370<br>$FEC_{(298.15)PBEh\text{-}3c/CPCM(Chloroform)}$=<br>0.53492601<br>$E_{PW6B95\text{-}D3(BJ)/def2\text{-}TZVPP/CPCM(Chloroform)}$=<br>-4710.95475878199

	C 0.26083505715214 -4.19866839564831 0.84712594891141<br>H -0.02143192189008 -5.00989522059525 0.18694431357978<br>C -3.27080785301976 -4.21700510926944 1.32032578608214<br>C -4.48745819075384 -4.67450912565658 0.81375709456390<br>H -4.79867448242523 -4.40643317401947 -0.18943436855707<br>C -5.30695477333991 -5.47881494616539 1.58940637719240<br>H -6.24844535545235 -5.83110297591065 1.18903686829762<br>C -4.91536774335893 -5.83738008097647 2.87375926711469<br>H -5.55176664859006 -6.47200065674317 3.47649445416703<br>C -3.70543525314988 -5.38602499110147 3.37964790697830<br>H -3.39362765588533 -5.66789784694185 4.37633405650834<br>C -2.88262353712461 -4.57791312508227 2.60640446170315<br>H -1.93638490401532 -4.24201951645970 3.01010262511761<br>C -1.71372476267419 -7.25404897549059 -0.72293843506979<br>C -1.82538964530803 -7.41209014011894 0.65092446342459<br>C -0.69569184473199 -7.69286265481591 1.40291406090547<br>C 0.54315451539060 -7.82307511001471 0.79090461098665<br>C 0.65430883063642 -7.65138615710572 -0.57856869212855<br>C -0.47144938640738 -7.35897030403458 -1.34733246890405<br>H -2.60632798467840 -7.05988429805233 -1.30634071560335<br>H -2.79681312571028 -7.31389955198147 1.11756150622784<br>Br -0.83390477627000 -7.90137240915279 3.27557238701487<br>H 1.42868896031314 -8.04670345691911 1.37087185597225<br>H 1.62143496795073 -7.74002821233669 -1.05692777512677<br>O -0.29854909951025 -7.19708315428868 -2.66918317013285<br>H -1.07567265060194 -6.78668507745410 -3.07316439815236<br>C 3.66511546311554 -2.00620078062087 -5.55647469331980<br>H 4.49600022901684 -2.66073887220156 -5.28799206263271<br>H 3.81705273388840 -1.68235911580496 -6.58516472722938<br>H 3.73354499484009 -1.12516712116761 -4.91634195357275	
17	Au -2.04916629048502 -4.56925761865222 -1.86238822983492<br>P -2.23121344313690 -3.38943550326484 0.09973855343188<br>O -2.23659308126305 -4.38978218494825 -5.58368914959921<br>C -1.39960254017018 -4.77362486674001 -4.60492697319783<br>C -1.98310436845280 -5.67303024303041 -3.71697827697105<br>C -3.32217437691609 -5.85402507029199 -4.26759856945528<br>C -4.42386492083878 -6.62462192632433 -3.90394655539837<br>H -4.38543008960762 -7.26907202497047 -3.03470756825051<br>C -5.56840121181147 -6.54373280527571 -4.68092096838603<br>H -6.43542268169169 -7.13525442059029 -4.41759154217205<br>C -5.62786018238833 -5.71194306495798 -5.80392905753766<br>H -6.53703082574149 -5.67257150015248 -6.38889972322750<br>C -4.54143702058792 -4.93738347568636 -6.18596243003525<br>H -4.57726348591376 -4.29417690260018 -7.05506413440638<br>C -3.41519593137486 -5.04344449708528 -5.39435688086003<br>C -0.05375700113781 -4.22566153558697 -4.65451257092954<br>C 0.97129524992462 -4.83862896448747 -3.92649235580436<br>H 0.76949288548486 -5.74088329282688 -3.36379626567906<br>C 2.24969638890420 -4.31835109446660 -3.94695211736018<br>H 3.02959365705223 -4.81226838715507 -3.37962596504561<br>C 2.55519031198317 -3.17678479552119 -4.69093697854260<br>C 1.53291611596958 -2.58033757824015 -5.42256748494198<br>H 1.74349121199207 -1.69611213834257 -6.01168435050348<br>C 0.24547131856951 -3.09360860439442 -5.41185071843894<br>H -0.52385676927470 -2.59615525398710 -5.98732849741996<br>C -3.07787364205876 -1.79132637930976 -0.05548726841838<br>C -2.78231916979543 -0.98732448014435 -1.15543071259143<br>H -2.08679566412415 -1.33117291295509 -1.91235383066776<br>C -3.37933031196517 0.25626491389721 -1.28768419533306<br>H -3.14581915629891 0.87588786492843 -2.14329455940988<br>C -4.28146610078475 0.70014549589794 -0.32896734768171<br>H -4.75256225118341 1.66836634583205 -0.43667323666281<br>C -4.58268285848946 -0.10027787634669 0.76353313181362<br>H -5.28693051099297 0.24135621733833 1.51051137278574<br>C -3.98174073521099 -1.34373007869374 0.90409374863412<br>H -4.22255339826288 -1.95932407264811 1.76128708612543<br>C -0.62821478417874 -3.01981676504216 0.86249183088194<br>C -0.42710814692936 -1.86575209507840 1.61653048642222<br>H -1.22081486487118 -1.13874153106769 1.73280769673069<br>C 0.79974788038024 -1.64145405038879 2.22379389300693<br>H 0.95283338551829 -0.74259455268659 2.80629618511722<br>C 1.82606691045139 -2.56647722445701 2.08523683758818<br>H 2.78158957916809 -2.38883907933997 2.56120421823584<br>C 1.62798510399598 -3.71622826905067 1.33298708282593<br>H 2.42470227462763 -4.43955539044818 1.21960568806434	**TS$_{syn}$ (1$_{Me}$ and 2$_{Br}$)**<br>E$_{PBEh-3c/CPCM(Chloroform)}$=<br>-4701.38988158<br>ZPE$_{PBEh-3c/CPCM(Chloroform)}$=<br>0.59841615<br>FEC$_{(298.15)PBEh-3c/CPCM(Chloroform)}$=<br>0.53112574<br>E$_{PW6B95-D3(BJ)/def2-TZVPP/CPCM(Chloroform)}$=<br>-4710.92128597106

	C 0.40672031244477 -3.94116650365803 0.71660555456767<br>H 0.26713872758984 -4.83919965062587 0.12718812092662<br>C -3.17104943619592 -4.32487942362196 1.33895840683036<br>C -4.37102393421545 -4.91783720860360 0.94516248463876<br>H -4.73084201776240 -4.80615970462806 -0.07147433557617<br>C -5.10802996060343 -5.66193807427749 1.85107730335839<br>H -6.03724301102954 -6.12046849061585 1.53968508756913<br>C -4.64774956160711 -5.82769735445511 3.15254957504498<br>H -5.21877855905946 -6.41820434892351 3.85698632738198<br>C -3.45333407522664 -5.24312990912663 3.54447434279938<br>H -3.08713667948566 -5.37704306776028 4.55356404679829<br>C -2.71408081777217 -4.49029453940735 2.64127217667725<br>H -1.77786320655178 -4.04985631476719 2.95743407734247<br>C -1.68288033066213 -7.48662184552863 -0.61833204883935<br>C -1.52544428742292 -7.35478974037903 0.75516804933733<br>C -0.25314155068531 -7.34532854720959 1.30215852312867<br>C 0.86389314028794 -7.46736352355096 0.48218741382648<br>C 0.70329829370735 -7.58304728491250 -0.88580139123261<br>C -0.57552078723387 -7.61765644696525 -1.48866223251784<br>H -2.68578400029563 -7.55037489675349 -1.02596688346095<br>H -2.40199468296005 -7.26545743144429 1.38466051241920<br>Br -0.02185469563580 -7.15926018456866 3.17308334845085<br>H 1.86130681499197 -7.45960176161259 0.90386165060015<br>H 1.57930474287505 -7.67646765500930 -1.51714033502682<br>O -0.71520235278752 -7.73771216165316 -2.77591461700340<br>H -1.35178901569408 -6.68701004498147 -3.24314100758207<br>C 3.94739385737823 -2.62178774056681 -4.69670697713724<br>H 4.66537851986782 -3.36412638812141 -5.04849080322243<br>H 4.02873199296550 -1.74681510302642 -5.33963325389107<br>H 4.25742473769534 -2.32912156490281 -3.69227140410927	
18	Au -1.32997461814445 -6.12260429986742 -0.54126465182012<br>P -1.81229884171043 -4.10706732981385 0.45416317680768<br>O -3.14723037355755 -3.55046369261115 -4.01062509005953<br>C -2.00966269558774 -4.28518228356139 -4.16789734956610<br>C -2.19599307286901 -5.56835205094750 -3.76459986904422<br>C -3.55705887804078 -5.65457043570271 -3.32643488785541<br>C -4.38002598052236 -6.66598098104484 -2.82718634994641<br>H -4.00037847927062 -7.66916654962427 -2.68120456633764<br>C -5.69695591135856 -6.35550914411164 -2.53213982234423<br>H -6.35381675587029 -7.12519896584369 -2.14851288775434<br>C -6.19890411967582 -5.06156731080223 -2.71962542306432<br>H -7.23271626495969 -4.85277538257866 -2.47805587683961<br>C -5.39715598858210 -4.03989350000651 -3.20718381784939<br>H -5.77905702075383 -3.03830901251727 -3.35403711443195<br>C -4.08844070041731 -4.37356038344326 -3.50367375628335<br>C -0.85985998113785 -3.60501830765935 -4.73669606394471<br>C 0.41100898841316 -4.17725376586369 -4.63261933819058<br>H 0.54104561374965 -5.12861918859772 -4.13066420433723<br>C 1.51564335656619 -3.53224808905406 -5.15559085481104<br>H 2.49121886357698 -3.99384787802686 -5.05681604146466<br>C 1.39858857152572 -2.30002419224660 -5.80122675862626<br>C 0.12967689504921 -1.74010598026511 -5.90614500210479<br>H 0.00657088353904 -0.78619739838526 -6.40515675975543<br>C -0.98536463373007 -2.37681891248779 -5.38291722147949<br>H -1.95372334667752 -1.90494115635988 -5.48140636972661<br>C -1.65497913706871 -2.68102695018025 -0.65363602971107<br>C -0.49694526271110 -2.57749846526131 -1.42346318346974<br>H 0.26089820828888 -3.35147606304641 -1.38163686698587<br>C -0.31036808580470 -1.48537455534835 -2.25287989795597<br>H 0.58633237894503 -1.41524176433504 -2.85409828256083<br>C -1.28503915091409 -0.49886779190246 -2.33122641237266<br>H -1.14358167869402 0.34796549193425 -2.98996802668218<br>C -2.44436622744594 -0.60613357113912 -1.57755579503834<br>H -3.20795946568124 0.15778834748606 -1.64217449352938<br>C -2.63167948691056 -1.69367717392141 -0.73439914455954<br>H -3.53768201015666 -1.76461486854294 -0.14680942132602<br>C -0.68395951193340 -3.76889108039589 1.83783811100101<br>C -0.22941457943136 -2.48049639410413 2.10752874929468<br>H -0.52028321079525 -1.65160210237317 1.47504365952971<br>C 0.60603023581142 -2.25254504626849 3.19206669243732<br>H 0.95853957203520 -1.24985567173574 3.39460484529969<br>C 0.98863563062341 -3.30525659634304 4.01128069014096<br>H 1.64293895453510 -3.12499676425154 4.85413550594854<br>C 0.53738197493797 -4.59146055131177 3.74484808637770<br>H 0.83793577754650 -5.41700877313864 4.37626290167458	**PC*syn* (1_{Me} and 2_{Br})**<br>$E_{PBEh-3c/CPCM(Chloroform)}$=<br>-4701.41381122<br>$ZPE_{PBEh-3c/CPCM(Chloroform)}$=<br>0.60320954<br>$FEC_{(298.15)PBEh-3c/CPCM(Chloroform)}$=<br>0.53505046<br>$E_{PW6B95-D3(BJ)/def2-TZVPP/CPCM(Chloroform)}$=<br>-4710.9448703409

	C -0.29336698955346 -4.82503285203038 2.66030189820328<br>H -0.63167680256026 -5.83481050188712 2.45867188393376<br>C -3.48253673365300 -4.01441316032061 1.15504207578306<br>C -4.53850433302622 -4.55750663953706 0.42609746857734<br>H -4.35674849746904 -5.04788277738925 -0.52266055090268<br>C -5.83359233951369 -4.47316065228496 0.91242840766158<br>H -6.64769321999915 -4.89575633369421 0.33863025104086<br>C -6.07988941575704 -3.85684543829418 2.13219262926057<br>H -7.09042071033724 -3.79788060581340 2.51486710288710<br>C -5.02891678132500 -3.32074173669873 2.86343134955031<br>H -5.21689761583237 -2.84297301087748 3.81583047288417<br>C -3.73126241803748 -3.39576431470317 2.37761492230263<br>H -2.92028852518473 -2.97471712574234 2.95759143907151<br>C -0.78773190528897 -8.12379355326156 -1.30253503995251<br>C -0.72354814984882 -8.71141756588765 0.00846530469709<br>C 0.44376718767719 -8.70924057380510 0.71223836465738<br>C 1.61597355821590 -8.15760788813715 0.13951009180854<br>C 1.61594140833339 -7.67188844258902 -1.13266035798208<br>C 0.44894174795522 -7.75815602175564 -1.99633651738552<br>H -1.61567777564769 -8.44579345250880 -1.93447253884801<br>H -1.62597800378171 -9.14600788466777 0.42228136313242<br>Br 0.50928059841464 -9.37803389928541 2.48120647599974<br>H 2.53158933241014 -8.12726755692306 0.71759415590596<br>H 2.53758885078362 -7.29839028256401 -1.56275749663913<br>O 0.50760398381294 -7.52807138100190 -3.21146117827545<br>H -1.46653211545484 -6.36377625872869 -3.80991303353821<br>C 2.60766297373472 -1.61359647918935 -6.36375154006756<br>H 3.07639156881565 -2.21627652006739 -7.14349590637750<br>H 2.35451922252569 -0.64765565121292 -6.79843952355128<br>H 3.36246336351489 -1.44611433640993 -5.59388624944060	
19	Au -1.13961805247511 -0.81398627112309 -0.40742119440589<br>P 0.14339880122872 -0.42520709778792 1.50048809824158<br>O -3.12434902142462 -0.91147180521183 -4.18526957396408<br>C -2.15363364546691 -0.49165292629863 -3.30539841439140<br>C -2.23184807220852 -1.12056113855177 -2.09847116417271<br>C -3.34907090577533 -2.02397919920043 -2.25011769368915<br>C -3.97254397590404 -2.95391645830075 -1.41596025181402<br>H -3.61348212771129 -3.11567250095692 -0.40658864386402<br>C -5.05581736945273 -3.66488645252831 -1.90337062339790<br>H -5.54981079282109 -4.39052181072994 -1.27018208828400<br>C -5.52802627250341 -3.46262283053104 -3.20625384693894<br>H -6.37702810958486 -4.03347134638369 -3.55895465341445<br>C -4.92909720029002 -2.54311213343798 -4.05428729657970<br>H -5.29002669844343 -2.38136796754629 -5.06167080522806<br>C -3.84910402077579 -1.84488052461223 -3.54270404345280<br>C -1.27531837613644 0.55294554940556 -3.82419789487425<br>C 0.04727709575249 0.66453783201622 -3.38744774536304<br>H 0.45056574497661 -0.06428556666241 -2.69729792533818<br>C 0.86600448111856 1.68136009982516 -3.85187129317020<br>H 1.88631548876019 1.74792684197218 -3.49606786963320<br>C 0.38873815129221 2.59774724833860 -4.77955927924938<br>C -0.91564191863249 2.48210123158230 -5.24056007297223<br>H -1.29826394353372 3.18688638838499 -5.96749235740293<br>C -1.74024796332216 1.47131141678785 -4.77097482715808<br>H -2.75788108208223 1.41037167872356 -5.13338352168889<br>H 1.02937759890924 3.38943320606488 -5.14545823635897<br>C 1.57064067690029 0.64613395392435 1.14601028434153<br>C 2.31746119689295 0.38617375167266 -0.00325709823235<br>H 2.04264489613384 -0.42984431369413 -0.66157791177868<br>C 3.41831672382850 1.16906645716329 -0.31162529042114<br>H 3.99296219184456 0.95950220822710 -1.20423873414830<br>C 3.77645282170067 2.22350241118393 0.51934291200163<br>H 4.63176033362489 2.83953667202662 0.27445112541042<br>C 3.03346154579722 2.48865392638938 1.66018154939220<br>H 3.30741172000549 3.31025385099250 2.30879101906763<br>C 1.93344568501994 1.70207578798733 1.97632610633152<br>H 1.36234177697738 1.92023812087513 2.86937394392657<br>C 0.85143821443917 -1.92706818643067 2.23953388056744<br>C 2.14334000138403 -1.95812859200456 2.75768684323693<br>H 2.77458098830976 -1.07992794629747 2.71146833924388<br>C 2.63148502080492 -3.12142014380822 3.33648749065728<br>H 3.63733674016270 -3.14011245968603 3.73485184673167<br>C 1.83381015252951 -4.25497662138122 3.40348926948851<br>H 2.21748519286579 -5.16116099523193 3.85366830550962<br>C 0.54458326665225 -4.22787516704761 2.88745030885334	**1_H**<br>$E_{PBEh\text{-}3c/CPCM(Chloroform)}$=<br>-1782.95628322<br>$ZPE_{PBEh\text{-}3c/CPCM(Chloroform)}$=<br>0.47720858<br>$FEC_{(298.15)PBEh\text{-}3c/CPCM(Chloroform)}$=<br>0.41005389<br>$E_{PW6B95\text{-}D3(BJ)/def2\text{-}TZVPP/CPCM(Chloroform)}$=<br>-1788.86997336558

	H -0.07876306931112 -5.11100806820080 2.93316204643352<br>C 0.05559373319295 -3.07012005780061 2.30264636480282<br>H -0.94816386437715 -3.06013533397546 1.89374677771516<br>C -0.74366626850114 0.39756087265444 2.85697815465127<br>C -1.66631225316660 1.39250264566464 2.53599165743850<br>H -1.86229676008651 1.64239946523732 1.49980933373259<br>C -2.33976164611600 2.06865246653635 3.54143076460775<br>H -3.05350381154099 2.84013561530985 3.28459645037109<br>C -2.10488347678930 1.74886336596567 4.87240286384003<br>H -2.63611838860106 2.27216782392118 5.65662076404615<br>C -1.19342635460789 0.75369728861060 5.19579712484797<br>H -1.01101300980946 0.49921022624732 6.23152278294948<br>C -0.51164964360743 0.07923980245824 4.19244785655299<br>H 0.19690832695403 -0.69478777172726 4.45776722839590	
20	Au -1.15847383387522 -0.77049594093025 -0.38511309104471<br>P 0.13662981142223 -0.41389970145430 1.51590363994564<br>O -3.06586314870399 -0.95043057048146 -4.20930659634804<br>C -2.17125880106062 -0.43927202091267 -3.29838522892249<br>C -2.19908130636305 -1.10652313886044 -2.10726541916775<br>C -3.19982198157394 -2.13018435456527 -2.30737413137706<br>C -3.72233271061302 -3.15127561347054 -1.51033684543415<br>H -3.36938550192349 -3.29283740883171 -0.49567783819965<br>C -4.69607312621813 -3.97836208364542 -2.04315777576811<br>H -5.11123851872520 -4.77552863734357 -1.44022546700738<br>C -5.15862511515920 -3.80195083522462 -3.35340646798984<br>H -5.92106813811829 -4.46435274323392 -3.74179447591076<br>C -4.66011154090486 -2.79303633194904 -4.16375541711332<br>H -5.01475718951835 -2.65117504821972 -5.17630164490433<br>C -3.68767588147518 -1.98017705711586 -3.60744606372980<br>C -1.42522176880855 0.72322688598478 -3.77483369908665<br>C -0.16021308741942 1.03342824812318 -3.26832283370254<br>H 0.31255589365630 0.38380119143684 -2.54442165123017<br>C 0.52388988408753 2.15709497250942 -3.70294086834155<br>H 1.50231592019345 2.37404947386784 -3.29375224819803<br>C -0.03129964008464 2.98882657421765 -4.66608409580473<br>C -1.27810132029839 2.68035760098459 -5.19238312948867<br>H -1.72152069180972 3.31775335639967 -5.94640688374338<br>C -1.96868965139534 1.56078455942697 -4.75473880350443<br>H -2.94472555178197 1.34986661060859 -5.17076709369161<br>H 0.50506725153820 3.86435958541550 -5.00774124473407<br>C 1.64399880373155 0.52834137670439 1.12787771523306<br>C 2.34734695219295 0.19959825093433 -0.03138387592533<br>H 1.99383213216019 -0.59027748142392 -0.68384029408549<br>C 3.50775201822111 0.88264515717713 -0.35959618473528<br>H 4.04742160762924 0.62044676105635 -1.26004310190142<br>C 3.96984458961201 1.90440121315412 0.46056741704272<br>H 4.87206009137301 2.44215186809193 0.19997181262394<br>C 3.27052459675446 2.23775478708750 1.61123640045820<br>H 3.62529972052731 3.03443186332197 2.25171085212680<br>C 2.11090788593229 1.55175374025075 1.94729540689361<br>H 1.57483771448589 1.82263010600751 2.84765480895083<br>C 0.71821019537360 -1.94933933584939 2.29188275684254<br>C 2.01598595855736 -2.09171944077741 2.77411583444200<br>H 2.73016476886212 -1.28422611637808 2.67612901924153<br>C 2.40280212372400 -3.27764197992007 3.38345689966119<br>H 3.41415685358026 -3.38439620318649 3.75325559359500<br>C 1.49777721163201 -4.32049069784755 3.51739885105105<br>H 1.80262230989424 -5.24591498032849 3.98798025221927<br>C 0.20242778619839 -4.18118451024544 3.03677008243276<br>H -0.50216665424886 -4.99654336765476 3.12978755045660<br>C -0.18578539109965 -3.00311984649702 2.42062709005202<br>H -1.19464461620750 -2.90809989484766 2.03604803373547<br>C -0.67559912561960 0.51531281265813 2.84976354430914<br>C -1.51907960044092 1.56998275435828 2.50321421553675<br>H -1.70135752060864 1.80401876756586 1.46079112278078<br>C -2.13147203917446 2.32554833660937 3.49097911352975<br>H -2.78415774234579 3.14300019329172 3.21462718496577<br>C -1.91439570253873 2.02667692648175 4.82986817198837<br>H -2.39826740572829 2.61247663884367 5.60042297346775<br>C -1.08200670706141 0.97260697717638 5.17884587827350<br>H -0.91405410317072 0.73453932093670 6.22089974380320<br>C -0.46120875234449 0.21810556760734 4.19300367501940<br>H 0.18607225551150 -0.60149061888165 4.47757797288555<br>C -0.24553533638880 -4.35315165755242 -1.54675450176065<br>C -0.24143083351152 -5.46220032826975 -0.71450035690769	**RC*anti* (1_H and 2_{Br})**<br>$E_{PBEh\text{-}3c/CPCM(Chloroform)}$=<br>-4662.19056319<br>$ZPE_{PBEh\text{-}3c/CPCM(Chloroform)}$=<br>0.57588953<br>$FEC_{(298.15)PBEh\text{-}3c/CPCM(Chloroform)}$=<br>0.50941166<br>$E_{PW6B95\text{-}D3(BJ)/def2\text{-}TZVPP/CPCM(Chloroform)}$=<br>-4671.57050887939

	C 0.90226328625438 -5.78032691297625 -0.00064168978213<br>C 2.03792434326561 -4.99042996472383 -0.10282139998499<br>C 2.03073376520017 -3.88186275728505 -0.93259818955106<br>C 0.89255995978347 -3.56065179921215 -1.66853061798700<br>H -1.14230619590340 -4.11673124708608 -2.10745629008670<br>H -1.13466432370629 -6.06794541178172 -0.63810838880260<br>Br 0.91827894571633 -7.30527408439065 1.11517240220578<br>H 2.93046834620234 -5.22427320034764 0.46150642604357<br>H 2.91574315066711 -3.26326205147154 -1.01216222481358<br>O 0.95019066453432 -2.48659914179091 -2.47855910065631<br>H 0.07157605774955 -2.26752396877764 -2.81766416489365	
21	Au -0.79168914290336 -1.07264663381508 0.13141208607381<br>P 0.32092327699296 0.10156875538692 1.75284045505099<br>O -2.73149633871569 -0.63988399488920 -2.92533863513237<br>C -1.56947851485646 -1.15655097625911 -2.50161364237021<br>C -1.73979542988398 -2.12798409021405 -1.51753537318965<br>C -3.18828100682768 -2.20533323413923 -1.37561865201390<br>C -4.04786683849951 -2.98109014329692 -0.60192879655063<br>H -3.65824351373812 -3.72013642927152 0.08624883380020<br>C -5.41218327486940 -2.78019713870599 -0.73662402511694<br>H -6.09938538108061 -3.37265932521238 -0.14718131208367<br>C -5.92385539132307 -1.82597765275688 -1.62250240128835<br>H -6.99474611535339 -1.69525821674184 -1.70223017352076<br>C -5.08572517072430 -1.04581078156778 -2.40624388434726<br>H -5.47245814929311 -0.30980121772641 -3.09826862682968<br>C -3.73271937136650 -1.27323131536558 -2.25293387102253<br>C -0.35873309572279 -0.63899408710444 -3.12671830837212<br>C 0.83882498206482 -1.34894669895839 -2.99844546534198<br>H 0.85594884245177 -2.29420374637683 -2.47015317954423<br>C 2.00390900603466 -0.85002859348071 -3.55329501453811<br>H 2.92486540634437 -1.40872018546430 -3.45265007203029<br>C 1.99141576461584 0.35795375401696 -4.24147943609680<br>C 0.80276189205888 1.06112868474195 -4.37953138961227<br>H 0.78645871524087 2.00043979510642 -4.91611512811245<br>C -0.36979497012005 0.56685028458304 -3.82980652924140<br>H -1.28541462980568 1.13237686384462 -3.93848663901287<br>H 2.90486496992713 0.74628706202721 -4.67258601738690<br>C 1.01828006886157 1.63034258420735 1.06221873425060<br>C 1.59390264497907 1.58664877888311 -0.20804241031958<br>H 1.60749251022723 0.66457888637423 -0.77865954665469<br>C 2.15540774622886 2.72905912880778 -0.75504344075616<br>H 2.59826493148737 2.68710182733520 -1.74136917682882<br>C 2.13950240109357 3.92209515059232 -0.04335666378860<br>H 2.57235817670581 4.81519152285744 -0.47461014079068<br>C 1.56305475188349 3.96978838901450 1.21765169450036<br>H 1.54606342859619 4.89798474442416 1.77328722445204<br>C 1.00323900929530 2.82711571379911 1.77289824012212<br>H 0.55494556609521 2.87745686805012 2.75681488502940<br>C 1.72292252526570 -0.80753302628530 2.45723700594236<br>C 2.92686576082583 -0.17407255005160 2.75396806460978<br>H 3.06188329785373 0.87871653983250 2.54176268200209<br>C 3.96652997508690 -0.89607926217588 3.32277061459475<br>H 4.90140989087992 -0.40067758062880 3.54890038872836<br>C 3.80839021930364 -2.24700075595822 3.59801428821932<br>H 4.62195299774926 -2.80805794649054 4.03876471200850<br>C 2.60916858739578 -2.88146798196696 3.30068344284339<br>H 2.48509056263900 -3.93622387493859 3.50649793321751<br>C 1.56932450211883 -2.16641043401128 2.72782133829957<br>H 0.64097112415148 -2.67208354791579 2.48935534149974<br>C -0.70250562143078 0.61028788433801 3.16076537661192<br>C -2.00128001136808 1.05405749895769 2.91507545504342<br>H -2.39505954382118 1.06520529537580 1.90538231499147<br>C -2.79847369791758 1.48299815212888 3.96418706683909<br>H -3.80541733608984 1.82621883870453 3.76753974558053<br>C -2.30796955587264 1.46265961289916 5.26370888864266<br>H -2.93373404351354 1.79136744244078 6.08303172438280<br>C -1.01860125600683 1.01461799856786 5.51199295235753<br>H -0.63533399470960 0.99341870065908 6.52351705103745<br>C -0.21368564173033 0.58958123005930 4.46406254123742<br>H 0.79010573015258 0.24125189745293 4.67030531398098<br>C -1.47359712202575 -5.43640554840300 -2.84688886600349<br>C -1.58001783684557 -6.50123463596579 -3.72502526572669<br>C -0.52453345210966 -7.39027760950106 -3.86873447553228<br>C 0.63779624867584 -7.21177965273504 -3.13026474168173<br>C 0.74423045475234 -6.14327106215313 -2.25832485375983	**TS*anti* (1H and 2Br)**<br>$E_{PBEh-3c/CPCM(Chloroform)}$=<br>-4662.14607070<br>$ZPE_{PBEh-3c/CPCM(Chloroform)}$=<br>0.5703612<br>$FEC_{(298.15)PBEh-3c/CPCM(Chloroform)}$=<br>0.50308745<br>$E_{PW6B95-D3(BJ)/def2-TZVPP/CPCM(Chloroform)}$=<br>-4671.52574289521

	C -0.30684022540724 -5.21446996018449 -2.07780317265045<br>H -2.31543377161782 -4.76128100380339 -2.74345435037719<br>H -2.49323816379890 -6.63143993223493 -4.29226730133788<br>Br -0.67127093063711 -8.85254380139818 -5.06738053249372<br>H 1.46812862072209 -7.89944567232633 -3.23222170866452<br>H 1.65717646235590 -6.01324734945231 -1.68904572595494<br>O -0.18799697517674 -4.22107759144869 -1.25202732755805<br>H -1.01221853595088 -3.13487464409289 -1.40515412231542	
22	Au -0.46040498902107 -2.86838117513913 0.66032744735455<br>P 0.11221773620223 -1.03580156110083 1.82991982117024<br>O -1.75508304767447 0.47672977299573 -2.14839511148883<br>C -1.33879912596490 -0.71612779548854 -2.65707504183880<br>C -2.12176357755545 -1.73303871880319 -2.21624881734929<br>C -3.11070565711433 -1.14061213479591 -1.36821549087563<br>C -4.20510217327570 -1.60821966131471 -0.63816360168550<br>H -4.45044289455143 -2.66220256154489 -0.61972248228345<br>C -4.97093440091465 -0.68689144784512 0.05535069306940<br>H -5.82726391169971 -1.02484871300638 0.62428878153692<br>C -4.65691155936030 0.67834639355759 0.04156892763270<br>H -5.27326314082257 1.37003764080470 0.60032499144392<br>C -3.57008124570098 1.16265450757217 -0.67048293723214<br>H -3.32230017339440 2.21571422926626 -0.68474341843990<br>C -2.82638319829615 0.22790602864457 -1.36754605351038<br>C -0.20423009053887 -0.69238291925340 -3.56490074264623<br>C 0.43949086351872 -1.88637452213055 -3.89965663676160<br>H 0.11068661920098 -2.82282959202726 -3.46725270590621<br>C 1.51395636485243 -1.88509582238088 -4.77226468752746<br>H 2.00083512473655 -2.81998610107037 -5.01844902373676<br>C 1.96599446069524 -0.69287606475145 -5.32463590771347<br>C 1.33188282859456 0.49758795377163 -4.99648894012433<br>H 1.67517052424537 1.43073097881729 -5.42397304682388<br>C 0.25490747212480 0.50215264961257 -4.12352949640011<br>H -0.22743490673573 1.43976811976979 -3.88257638728926<br>H 2.80667371145631 -0.69269246949152 -6.00598838938069<br>C 0.44994139443712 0.39295044383783 0.76283341386520<br>C 1.29318016739761 0.21047458239911 -0.33253752539399<br>H 1.69408681056666 -0.77056017365298 -0.56038903793990<br>C 1.62720317165731 1.28595945254325 -1.13819967216255<br>H 2.28455588598629 1.13771052962191 -1.98399318414944<br>C 1.10516977062271 2.54466249438485 -0.86869192835346<br>H 1.35993387073976 3.38274045711568 -1.50424233312799<br>C 0.25214919221841 2.72576412520952 0.20998612337328<br>H -0.16035527810183 3.70395504835449 0.41942406990105<br>C -0.07278500288248 1.65404705409340 1.03090158259786<br>H -0.73249327716090 1.80925263573386 1.87452018555331<br>C 1.62589044133367 -1.26160496425949 2.80875024437533<br>C 2.56535309996566 -0.24272990461916 2.94328537434747<br>H 2.42842449876872 0.70227155122498 2.43361756045069<br>C 3.68968192343427 -0.43581598973902 3.73378067134065<br>H 4.41812314097257 0.35822219064519 3.83139962034222<br>C 3.87871059032022 -1.64094764024478 4.39499578379846<br>H 4.75717625290977 -1.78956977948727 5.00917953077639<br>C 2.94303070968852 -2.65892159604073 4.26353371624142<br>H 3.08896288770797 -3.60176509550591 4.77368230500830<br>C 1.82173605494414 -2.47295415882803 3.47033430873152<br>H 1.10095470964103 -3.27566176555483 3.36826757205504<br>C -1.14568719554770 -0.48397865473461 3.01483474660145<br>C -2.48166980573383 -0.50383563803357 2.61976779951397<br>H -2.75910855239031 -0.86662493161604 1.63729804636080<br>C -3.46798721148165 -0.05528895131800 3.48370844612908<br>H -4.50266873558623 -0.07230801795940 3.16772625672668<br>C -3.12780474317635 0.40550353394704 4.74881134778987<br>H -3.89933215379459 0.74827016193044 5.42584149957450<br>C -1.79824405304098 0.42215318713649 5.14663758453502<br>H -1.52978115984133 0.77805879089605 6.13249176031417<br>C -0.80584811989911 -0.01844668149045 4.28236502514154<br>H 0.22711679544079 0.00014325998994 4.60458547483354<br>C -1.10115413348696 -5.41125623956613 -2.55309360696501<br>C -0.49196753800639 -5.80213269499747 -3.73419341532527<br>C 0.88442164736581 -5.69287273264791 -3.86890192514990<br>C 1.64931723431979 -5.20457349612806 -2.81968431421410<br>C 1.03523021547683 -4.81196468047698 -1.64221432641844<br>C -0.35942419901139 -4.89460183471294 -1.47518916585229<br>H -2.17700721342066 -5.49715418439095 -2.45740246562580<br>H -1.10024615840416 -6.18320236512457 -4.54439850140196	**PC*anti* (1_H and 2_{Br})**<br>$E_{PBEh\text{-}3c/CPCM(Chloroform)}$=<br>-4662.20025129<br>$ZPE_{PBEh\text{-}3c/CPCM(Chloroform)}$=<br>0.57572778<br>$FEC_{(298.15)PBEh\text{-}3c/CPCM(Chloroform)}$=<br>0.50944811<br>$E_{PW6B95\text{-}D3(BJ)/def2\text{-}TZVPP/CPCM(Chloroform)}$=<br>-4671.57848217592

	Br 1.72073082407021 -6.20234679396060 -5.48901487232949<br>H 2.72460741184548 -5.11964129926659 -2.91033664350988<br>H 1.65302514678485 -4.43317045995909 -0.83525371079032<br>O -0.98235917599357 -4.53288827602698 -0.37460119802059<br>H -2.03336005337167 -2.77250151802210 -2.48792500991039	
23	O -0.04012726587963 0.01825267942042 -1.56570219935438<br>C -0.11553375349848 -0.43593828250060 -0.28465869740920<br>C -0.39161503167653 -1.76457553901894 -0.25703801722035<br>C -0.49963222042179 -2.18318617339142 -1.62144355938208<br>C -0.76076488841836 -3.38785927542164 -2.27684662657495<br>H -0.94175026089524 -4.29710615127357 -1.71852563080820<br>C -0.78240509086584 -3.38878516210719 -3.66002528710223<br>H -0.98281656280916 -4.31049131183765 -4.19022702958433<br>C -0.54945350686118 -2.21791758687866 -4.39293074386193<br>H -0.57395948192112 -2.25510404130962 -5.47389631343157<br>C -0.28806685963677 -1.01119846360313 -3.76272372285966<br>H -0.10756233682752 -0.10359532465180 -4.32316499612380<br>C -0.27135892385388 -1.03288086689440 -2.38014585260849<br>C 0.10423029417178 0.53791708876319 0.77256433185769<br>C 0.01860286394693 0.14438451994462 2.11108291892603<br>H -0.21411907186063 -0.88250727705969 2.36202417919588<br>C 0.22758512893205 1.05794638205217 3.12889881615432<br>H 0.15713853838077 0.73495586411539 4.15925732773422<br>C 0.52520011652429 2.38251842910326 2.83163749173728<br>C 0.61091252263264 2.78226070400043 1.50597624680204<br>H 0.84173534789542 3.81131008843820 1.26352155664199<br>C 0.40315756494673 1.87096889854914 0.48165734771867<br>H 0.47648443700725 2.20579008408815 -0.54405704292541<br>H -0.50429821683206 -2.37954007084578 0.62107323780155<br>H 0.68813983582035 3.09627934031913 3.62832736067694	**2-phenylbenzofuran**<br>$E_{PBEh-3c/CPCM(Chloroform)}$=<br>-613.39669571<br>$ZPE_{PBEh-3c/CPCM(Chloroform)}$=<br>0.20458554<br>$FEC_{(298.15)PBEh-3c/CPCM(Chloroform)}$=<br>0.16819127<br>$E_{PW6B95-D3(BJ)/def2-TZVPP/CPCM(Chloroform)}$=<br>-615.789453303793
24	Au -1.38406887384279 -6.26139561068044 -3.14271199105032<br>P -2.08152783725426 -5.21232802242170 -1.18290042380678<br>O 0.13424920238426 -7.84028881709825 -6.81470557660693<br>C 0.15981037359059 -6.99220481646659 -5.73384376832358<br>C -0.83777652335511 -7.25044483933677 -4.84015601602892<br>C -1.53530356505912 -8.37860902043551 -5.41668365309977<br>C -2.63291632395854 -9.14921644677291 -5.02707661353574<br>H -3.15403216709487 -8.93712839497324 -4.10079108963753<br>C -3.04053660118781 -10.18742982358559 -5.84687703019845<br>H -3.88866331241297 -10.79568859491392 -5.56041800568101<br>C -2.37415966940748 -10.46767546047692 -7.04645755399932<br>H -2.71684797709046 -11.28578195629967 -7.66629291074057<br>C -1.28226735168356 -9.71746776037876 -7.45684064410930<br>H -0.76341375459944 -9.93024956377969 -8.38236538559471<br>C -0.89433627533115 -8.68582260267775 -6.61993095432705<br>C 1.21679774823999 -5.98634982062363 -5.77156037681763<br>C 1.77470036102196 -5.48752495845061 -4.59158889793570<br>H 1.45891931376581 -5.88125807959254 -3.63466287602659<br>C 2.75719593504813 -4.51122144251414 -4.63068525907986<br>H 3.17645159213669 -4.14018067997082 -3.70441334049152<br>C 3.21580593665032 -4.02522873377104 -5.84795563436342<br>C 2.68419847868581 -4.52940672677905 -7.02691740298083<br>H 3.03660184271470 -4.16145289279426 -7.98183614955134<br>C 1.69626883083663 -5.50135720541148 -6.99210765843789<br>H 1.28668277274352 -5.87079314298298 -7.92291594355010<br>C -3.22488937473084 -3.82396820134051 -1.43997383017464<br>C -2.96313416322831 -2.94071692875382 -2.48662183243853<br>H -2.11249819151468 -3.10314548605558 -3.13850398374661<br>C -3.79066252098948 -1.84956968757796 -2.70039183626468<br>H -3.58104672022822 -1.16761991192442 -3.51378932368355<br>C -4.89039056485761 -1.63953318420622 -1.87824781173374<br>H -5.54027718166207 -0.79146528732730 -2.05001058487363<br>C -5.15890187700496 -2.52092554862701 -0.84068484934892<br>H -6.01677400887038 -2.36217742951512 -0.20064668225806<br>C -4.32815044892388 -3.61058926201067 -0.61825161780404<br>H -4.54641935090291 -4.29068743884660 0.19510382471862<br>C -0.72319107081242 -4.53508499171320 -0.18465175991804<br>C -0.83443566271319 -3.32729046127993 0.49874681977792<br>H -1.73935917058814 -2.73743400060930 0.42895678518932<br>C 0.22138585312753 -2.87037607086014 1.27547468460037<br>H 0.13091501367560 -1.92940178587097 1.80194209986532<br>C 1.38700342138343 -3.61687638048888 1.37721450923271<br>H 2.20800055988247 -3.25822500641274 1.98419650671826<br>C 1.50071576861467 -4.82209931303786 0.69645850933239<br>H 2.40774460807910 -5.40759500566120 0.76938431442010	**RC_{syn} (1_H and 2_{Br})**<br>$E_{PBEh-3c/CPCM(Chloroform)}$=<br>-4662.19156629<br>$ZPE_{PBEh-3c/CPCM(Chloroform)}$=<br>0.57577380<br>$FEC_{(298.15)PBEh-3c/CPCM(Chloroform)}$=<br>0.50912951<br>$E_{PW6B95-D3(BJ)/def2-TZVPP/CPCM(Chloroform)}$=<br>-4671.57157035989

	C 0.45265542577415 -5.27776988827585 -0.08747995705223<br>H 0.55454850159223 -6.21542233035219 -0.62074237047605<br>C -2.95645458604457 -6.35888507908640 -0.07545650939519<br>C -3.84879195885736 -7.27048486027789 -0.63974746194587<br>H -4.01239780389459 -7.28571459501604 -1.71121220680580<br>C -4.53001907051538 -8.16864300102430 0.16627543861948<br>H -5.21896469652341 -8.87435641386762 -0.27881579475493<br>C -4.32081343870131 -8.16757875963911 1.54023322241532<br>H -4.84663503606967 -8.87476047657624 2.16827122312022<br>C -3.43257497565708 -7.26386082777247 2.10431898364475<br>H -3.26090185929476 -7.26427949304736 3.17235746258417<br>C -2.75050684938793 -6.36047374711136 1.30035498340474<br>H -2.05155457354862 -5.66910434487351 1.75279700609675<br>C -0.28479453234129 -9.00812181673334 -1.22406483632312<br>C -0.55892043848474 -8.92956912821898 0.13355080362666<br>C 0.47437890887215 -8.70884554146545 1.03002889685004<br>C 1.78043151789958 -8.57516790385997 0.58001484330816<br>C 2.05022519978512 -8.64394245974175 -0.77642820403092<br>C 1.01985226925195 -8.85580542039200 -1.69128816691650<br>H -1.09641015665079 -9.20177855728991 -1.91579499058925<br>H -1.57964652131315 -9.04221318042605 0.47446273658025<br>Br 0.10945743330949 -8.57923537248374 2.87923297732708<br>H 2.59288389113631 -8.40609113062310 1.27424253756738<br>H 3.06658749539723 -8.52900728827368 -1.13118063060568<br>O 1.34354690417161 -8.91116594105784 -2.99361574277156<br>H 0.55585522986346 -8.83949068598566 -3.54928909988468<br>H 3.98604288310007 -3.26566718483279 -5.87752989626953	
25	Au -1.04277570257703 -6.72566630930491 -2.89876343767726<br>P -1.96833059406092 -5.42251478061494 -1.24978033599679<br>O -0.45952233042752 -7.03109716476413 -6.55758894367689<br>C 0.21868852918703 -7.04620066981197 -5.39754776242574<br>C -0.28170632104350 -7.97472200149060 -4.49066604517362<br>C -1.37533628738064 -8.59643374836954 -5.22907359358490<br>C -2.28652815180955 -9.60965867796237 -4.94055926726940<br>H -2.26588063811161 -10.11601385620929 -3.98375039171828<br>C -3.21839813426489 -9.95019688544388 -5.90758706325498<br>H -3.93466829616760 -10.73585064796526 -5.70604766115581<br>C -3.25340033491327 -9.29896401673928 -7.14535599623808<br>H -3.99421447037252 -9.58975625966101 -7.87813725628736<br>C -2.35394693687477 -8.28868176631597 -7.45493514860785<br>H -2.37164423869389 -7.78245213220269 -8.41080852615230<br>C -1.43554036640118 -7.97528501799700 -6.47247270138367<br>C 1.35234034008921 -6.13735380856056 -5.29973994263145<br>C 2.33052601702859 -6.36214699678705 -4.32703439242400<br>H 2.26125115982153 -7.22839226642397 -3.68197352753695<br>C 3.40481768653558 -5.49851206991147 -4.20759570492497<br>H 4.15828695177101 -5.68490205609434 -3.45381718029341<br>C 3.52098456998801 -4.40390127381693 -5.05599581694146<br>C 2.55862861605081 -4.18251403847456 -6.03155624256092<br>H 2.64578609724723 -3.33386586116094 -6.69701968257136<br>C 1.48007563952382 -5.04357890408289 -6.15851175513143<br>H 0.73507815382836 -4.85115557457399 -6.91890390778623<br>C -3.17617635851931 -4.20371412072729 -1.84210308592009<br>C -2.89941035288736 -3.50923401152897 -3.01875109511832<br>H -1.99698478983412 -3.72232378073547 -3.58002464924934<br>C -3.77975800052578 -2.54284376578768 -3.47893137079962<br>H -3.55933454943522 -2.00720651893241 -4.39273376675915<br>C -4.94493721749821 -2.27075207254372 -2.77324025074053<br>H -5.63515067999192 -1.52093571641147 -3.13707074736600<br>C -5.22650484385287 -2.96440253686794 -1.60501278276937<br>H -6.13453882444334 -2.75688238974872 -1.05450308104269<br>C -4.34468013197449 -3.92859278427448 -1.13659340678033<br>H -4.57397637739498 -4.46299410682453 -0.22355219513470<br>C -0.73867210618164 -4.47762804943375 -0.30974013090926<br>C -1.02794083432634 -3.22091323407216 0.21699943453259<br>H -1.99102636603966 -2.75856089923736 0.04205677172655<br>C -0.07528758024165 -2.55102093268026 0.97088673128196<br>H -0.30295171997033 -1.57373037232387 1.37554941649723<br>C 1.16358151454800 -3.13227653431922 1.20567481877536<br>H 1.90377237406140 -2.60739469857368 1.79533654395964<br>C 1.45423199257725 -4.38400612410053 0.68016380855857<br>H 2.41889021329487 -4.84091158541706 0.85754160163780<br>C 0.50953789349903 -5.05371600111220 -0.08184605393714<br>H 0.75102100783789 -6.02701944148967 -0.49154480975422<br>C -2.85418473119170 -6.43703831059207 -0.03303329882887	**TS*syn* (1_H and 2_{Br})**<br>$E_{PBEh\text{-}3c/CPCM(Chloroform)}$=<br>-4662.15535555<br>$ZPE_{PBEh\text{-}3c/CPCM(Chloroform)}$=<br>0.57047806<br>$FEC_{(298.15)PBEh\text{-}3c/CPCM(Chloroform)}$=<br>0.50518698<br>$E_{PW6B95\text{-}D3(BJ)/def2\text{-}TZVPP/CPCM(Chloroform)}$=<br>-4671.53712883564

	C -3.72371659681420 -7.42078583598524 -0.50485022446543<br>H -3.86787307718908 -7.56029584938630 -1.57023038160605<br>C -4.40323745631145 -8.23293289075013 0.38744819907903<br>H -5.07533534008398 -8.99511450611133 0.01595577246586<br>C -4.21198333213131 -8.07560930874393 1.75562169681421<br>H -4.73513685834992 -8.71762883174532 2.45213232710284<br>C -3.34483319471063 -7.10171293188469 2.22626427451589<br>H -3.18641894872075 -6.98186277070865 3.28965101230338<br>C -2.66629152971068 -6.28018557438572 1.33531291156195<br>H -1.98371620877714 -5.53223482332778 1.71628975407173<br>C -0.19293661413856 -9.22262495903261 -1.10866990355248<br>C -0.37009436980635 -8.87714162243990 0.22489097355162<br>C 0.70851983406014 -8.42228233446126 0.96480889127783<br>C 1.96460490796084 -8.30897443129525 0.37771211486654<br>C 2.13355515811458 -8.64021385762317 -0.95346770920901<br>C 1.06785819756970 -9.13064758088452 -1.74327316873184<br>H -1.03275643854531 -9.63259398522962 -1.65897590233006<br>H -1.35102273658542 -8.97253742357404 0.67347685576741<br>Br 0.48207591011263 -7.94539699122607 2.78399686016322<br>H 2.81126873610251 -7.95193736258360 0.95073357785677<br>H 3.11564688934831 -8.54895730260552 -1.40272699215651<br>O 1.24128721032546 -9.45211942414123 -2.99143035664256<br>H 0.47156814337027 -8.69612207514669 -3.74157230599824<br>H 4.36132539642867 -3.72900891525281 -4.95964884116087	
26	Au -0.15454342712683 -7.69269522035790 -1.26512129324689<br>P -1.48556608303530 -5.87620206385771 -0.80078003019256<br>O -2.02625387254910 -6.55579222885622 -5.40247208902974<br>C -0.69883698955094 -6.82718105889015 -5.25474151992712<br>C -0.50817360111310 -8.00952207653319 -4.61549674129161<br>C -1.81486789340116 -8.53037609189746 -4.34444868477117<br>C -2.32151883015234 -9.68769689039580 -3.74971219017394<br>H -1.65702444225768 -10.44182273724235 -3.34785129885026<br>C -3.69544555422301 -9.85003149966169 -3.69502105873933<br>H -4.11006445112416 -10.74137288596029 -3.24266972923176<br>C -4.56385407527789 -8.88100971364853 -4.21368011323036<br>H -5.63255077855220 -9.03892182326718 -4.15313988685872<br>C -4.08265535034360 -7.72094794997084 -4.80204731503713<br>H -4.74841505115725 -6.96889834793290 -5.20417810219027<br>C -2.70758593610720 -7.58320538415845 -4.85471751139095<br>C 0.23610985109114 -5.86086061838836 -5.80554221748068<br>C 1.57325480918861 -5.88307777492092 -5.40208369165848<br>H 1.91260446190356 -6.61788341487745 -4.68208058388113<br>C 2.47180038982959 -4.95888585378009 -5.90775443084189<br>H 3.50343240068939 -4.98500909138193 -5.58182365093217<br>C 2.05331709545904 -4.00011626387493 -6.82284003351819<br>C 0.72718254570257 -3.97741066390241 -7.23203201384474<br>H 0.39284092891666 -3.23753779904024 -7.94768732996566<br>C -0.17774628271292 -4.89836519092669 -6.72784980486753<br>H -1.20788568722636 -4.86146329167312 -7.05557945864340<br>C -1.62643328656882 -4.69446009965967 -2.16804511231447<br>C -0.45129498003453 -4.26987770765029 -2.78717306704681<br>H 0.50724106862725 -4.67125242486881 -2.47850337016772<br>C -0.50286081273696 -3.33413972891521 -3.80569575204318<br>H 0.41165298535734 -3.01427097359758 -4.28728875876560<br>C -1.72777915382545 -2.82922079976799 -4.22307549995663<br>H -1.76743182806850 -2.10685615996839 -5.02786634649127<br>C -2.89939587711567 -3.26066844810227 -3.61919569927448<br>H -3.85513632883982 -2.87375226049880 -3.94734726326637<br>C -2.85287141503778 -4.19018011925827 -2.58835619681187<br>H -3.77270570368663 -4.51500611880874 -2.11994099019127<br>C -0.83682741113013 -4.91423391788155 0.59757985038159<br>C -0.94111647174523 -3.52586154922362 0.63454667724377<br>H -1.38830458691544 -2.98629816520514 -0.19046428533094<br>C -0.46663429175296 -2.82343477618868 1.73335925251300<br>H -0.54777555470201 -1.74471743125158 1.75467491917467<br>C 0.11046622257843 -3.50095313165592 2.79808826560008<br>H 0.48277738285180 -2.95051076531542 3.65212865113403<br>C 0.21622638253677 -4.88531563331017 2.76435247563023<br>H 0.67101422503466 -5.41785902543263 3.58903076852856<br>C -0.25193084410077 -5.59107469707983 1.66709809988534<br>H -0.15533689885317 -6.67049582759775 1.64981581518580<br>C -3.18454315866317 -6.31574781105062 -0.34243916873526<br>C -3.82463256350175 -7.32400606675432 -1.06023487071703<br>H -3.30510710570948 -7.84848880921481 -1.85314728361369<br>C -5.13565338964917 -7.66201423143586 -0.76388896038075	**PC*syn* (1$_{H}$ and 2$_{Br}$)**<br>E$_{PBEh-3c/CPCM(Chloroform)}$= -4662.17977270<br>ZPE$_{PBEh-3c/CPCM(Chloroform)}$= 0.57530599<br>FEC$_{(298.15)PBEh-3c/CPCM(Chloroform)}$= 0.50930321<br>E$_{PW6B95-D3(BJ)/def2-TZVPP/CPCM(Chloroform)}$= -4671.56138713624

	H -5.62582460721550 -8.44467946633660 -1.32760990116553<br>C -5.81006488978605 -7.00414943979715 0.25631508417553<br>H -6.83123278926451 -7.27376765922132 0.49215021315136<br>C -5.17256638542954 -6.00397579438563 0.97728817846448<br>H -5.69398991426656 -5.49182307230578 1.77507937480376<br>C -3.86248610601773 -5.65643059242001 0.67968788820540<br>H -3.37616364624580 -4.87530747333217 1.24941000075608<br>C 1.16829287297264 -9.44314253895461 -1.51217358533250<br>C 1.16068440786649 -9.77368722936952 -0.11245181207512<br>C 2.09441053044693 -9.24643234645533 0.72867959682012<br>C 3.10490071885155 -8.39318422644837 0.22097504145303<br>C 3.19790456486732 -8.12763628455970 -1.11130528495229<br>C 2.32505839222951 -8.75620624049903 -2.09039987442172<br>H 0.64096618132972 -10.13276039915571 -2.17173143745255<br>H 0.39122238977040 -10.44320274467137 0.25375264328588<br>Br 2.02583100639521 -9.58785083251331 2.58860698113200<br>H 3.82228906434410 -7.94910668425265 0.90038543820560<br>H 4.00845947909730 -7.50940207669408 -1.47833587388117<br>O 2.54641901642519 -8.69734591350554 -3.30717351901062<br>H 0.44382549025709 -8.47219513648014 -4.39990631109528<br>H 2.75725144215389 -3.27768423355124 -7.21489621144053	
27	Au -1.15056402019014 -0.82205168831939 -0.39368419019910<br>P 0.14972126905919 -0.43467370193169 1.50298705189449<br>O -3.15640964185313 -0.90584117736590 -4.16048905880912<br>C -2.17894525517213 -0.49429564522208 -3.28481982470750<br>C -2.25497458207296 -1.12267519599044 -2.07799551640239<br>C -3.37914817491242 -2.01853712863250 -2.22489456356012<br>C -4.00479708080203 -2.94468826611116 -1.38828028234581<br>H -3.64146988061690 -3.11010543563219 -0.38105213106890<br>C -5.09600471699269 -3.64710324242831 -1.87049281131614<br>H -5.59178425007798 -4.36976657069352 -1.23532055834386<br>C -5.57399649853056 -3.43994321040825 -3.17040839918886<br>H -6.42907914119709 -4.00417028119046 -3.51900899539516<br>C -4.97305770343978 -2.52386101273142 -4.02080185563526<br>H -5.33836383294598 -2.35833909764023 -5.02598398797162<br>C -3.88502001412960 -1.83458592972604 -3.51449113137725<br>C -1.29469094176538 0.54238969262103 -3.80810957740248<br>C 0.03239748635848 0.64071233859970 -3.38360419322513<br>H 0.43678814697587 -0.09157915794387 -2.69851711528170<br>C 0.86455180129437 1.64638283164707 -3.84653259637910<br>H 1.89071028305410 1.71294200870662 -3.50913503642343<br>C 0.36601352731650 2.55173595468056 -4.76296940561397<br>C -0.93196121066030 2.47427943654826 -5.22832995490355<br>H -1.29948745780865 3.19197980392299 -5.94991580607600<br>C -1.75566938323588 1.46844904031395 -4.74865910629588<br>H -2.77571428155678 1.42176822000925 -5.10521720097372<br>F 1.16671980587153 3.52620311232034 -5.21973285249919<br>C 1.56872593114552 0.64241733083986 1.13305560221802<br>C 2.31011161571167 0.37869233881404 -0.01896372121525<br>H 2.03735472735052 -0.44498708605168 -0.66860592834935<br>C 3.40314381904426 1.16703524398203 -0.34075071143253<br>H 3.97383285474139 0.95443802876470 -1.23520182361532<br>C 3.75883298035729 2.23078067272520 0.47944260549689<br>H 4.60783440785358 2.85119049336893 0.22394274660352<br>C 3.02150638937855 2.49933600907774 1.62307922881529<br>H 3.29370187388751 3.32795293795750 2.26343836831440<br>C 1.92934367495759 1.70725320922414 1.95274991498805<br>H 1.36268667157998 1.92823857339138 2.84792316429720<br>C 0.87179938115437 -1.93502100971047 2.23106468209363<br>C 2.16794771290772 -1.96010542175965 2.73894192809627<br>H 2.79356858240442 -1.07798675610813 2.69070283356838<br>C 2.66742569777885 -3.12236052548340 3.31002153244300<br>H 3.67646113014256 -3.13645914954196 3.70043861400306<br>C 1.87692228655599 -4.26081247500083 3.37950698642062<br>H 2.26945739622506 -5.16619758859830 3.82360842362493<br>C 0.58350810172541 -4.23962307443948 2.87382026812196<br>H -0.03425729768464 -5.12655851167390 2.92155044349704<br>C 0.08310927430976 -3.08284542760287 2.29675194798264<br>H -0.92392716620972 -3.07740109153272 1.89589200731778<br>C -0.72566046983842 0.38287763830767 2.86997987750326<br>C -1.65708534972600 1.37330467103284 2.56057285125015<br>H -1.86651330468013 1.62297370408292 1.52697275911204<br>C -2.32209715380690 2.04532966087520 3.57436630969005<br>H -3.04269798046193 2.81336998851451 3.32653462050477<br>C -2.06993533795816 1.72581238478321 4.90222570436091	**1_F**<br>$E_{PBEh\text{-}3c/CPCM(Chloroform)}$=<br>-1882.00974792<br>$ZPE_{PBEh\text{-}3c/CPCM(Chloroform)}$=<br>0.46883430<br>$FEC_{(298.15)PBEh\text{-}3c/CPCM(Chloroform)}$=<br>0.41005389<br>$E_{PW6B95\text{-}D3(BJ)/def2\text{-}TZVPP/CPCM(Chloroform)}$=<br>-1888.25732035227

	H -2.59456365624673 2.24585276724023 5.69303101742111<br>C -1.14965301379963 0.73510486464867 5.21415552217319<br>H -0.95378622116241 0.48083964130957 6.24747127786625<br>C -0.47626771830418 0.06485325870766 4.20238785013312<br>H 0.23928199569720 -0.70572031554691 4.45881767219574	
28	Au -1.15315762770016 -0.78150429006083 -0.38107754206980<br>P 0.15401572694138 -0.42471174206076 1.51174646534451<br>O -3.10653600328053 -0.94194025341052 -4.18291553724231<br>C -2.20040919184506 -0.43755616052548 -3.28055437128500<br>C -2.21032782484773 -1.11309140281581 -2.09413010371022<br>C -3.21357536699724 -2.13618366861793 -2.28804937251806<br>C -3.72528331044704 -3.16252280734037 -1.49089060917285<br>H -3.35735328062887 -3.31296672611224 -0.48285992103573<br>C -4.70820000532867 -3.98422734581347 -2.01539767188978<br>H -5.11513170272936 -4.78530425294442 -1.41205371838652<br>C -5.19009244394094 -3.79743264874304 -3.31708078506724<br>H -5.95921479070175 -4.45603202846948 -3.69866312518087<br>C -4.70229590846908 -2.78321764165171 -4.12726540953448<br>H -5.07195803861979 -2.63330612963815 -5.13324972629142<br>C -3.72037877894227 -1.97613872671002 -3.57949863187148<br>C -1.46161759792604 0.72759249214348 -3.76082849689935<br>C -0.19151802085595 1.03969384890791 -3.26924701743093<br>H 0.29039364967903 0.39189790386370 -2.55038573940690<br>C 0.49478568391236 2.16072078629174 -3.70438371054932<br>H 1.47880696401905 2.38901913555406 -3.31620260075633<br>C -0.09180695757741 2.97206964366165 -4.65614895495966<br>C -1.33661643206442 2.68933523011150 -5.18252536295315<br>H -1.77672117010670 3.33691898756493 -5.92933762816975<br>C -2.01438169633200 1.56756624992543 -4.73275249473546<br>H -2.99413118198609 1.36010299897613 -5.14067992641760<br>F 0.56807724132664 4.05753467400002 -5.08507637567555<br>C 1.66101234357690 0.51517526178442 1.11734591057335<br>C 2.35631251426458 0.19014216825761 -0.04782501943337<br>H 1.99577712884626 -0.59345492360019 -0.70398293105786<br>C 3.51814627114157 0.86978531967710 -0.37813075335403<br>H 4.05195012472643 0.61028027526829 -1.28284970552504<br>C 3.98935654161923 1.88475657286511 0.44526800525291<br>H 4.89263289764762 2.41973743981266 0.18269468460492<br>C 3.29758325783940 2.21485140115174 1.60134411282990<br>H 3.65888468599772 3.00653311418949 2.24435401005386<br>C 2.13687084039973 1.53194745163787 1.93988779566724<br>H 1.60694690328228 1.79999305888527 2.84471297310289<br>C 0.73628387902420 -1.96027707417691 2.28702986656779<br>C 2.03911473292346 -2.10940385133806 2.75307576432367<br>H 2.75779556589041 -1.30769019061204 2.64171609720473<br>C 2.42524560723553 -3.29493565876495 3.36378117689741<br>H 3.44057192480261 -3.40706459503668 3.72086179203251<br>C 1.51443906053829 -4.33018795276873 3.51567618655637<br>H 1.81882702272190 -5.25499257373881 3.98777000432307<br>C 0.21384903889652 -4.18398967167471 3.05140578350912<br>H -0.49565036928530 -4.99328453395900 3.15873233934804<br>C -0.17357608852461 -3.00673369669092 2.43334685576134<br>H -1.18679635743028 -2.90641067980850 2.06172444839047<br>C -0.65175302752115 0.50592609953259 2.84848830859519<br>C -1.49580072464382 1.56121724590503 2.50506429339324<br>H -1.68251287465084 1.79469789814347 1.46331085823618<br>C -2.10301185611676 2.31806265176263 3.49501724831693<br>H -2.75619990098431 3.13590467983751 3.22104181927374<br>C -1.87998209339464 2.01996520915792 4.83311101701985<br>H -2.35969702144556 2.60677599940444 5.60548954826569<br>C -1.04692387005440 0.96543787737785 5.17901887022506<br>H -0.87425921309592 0.72800302530074 6.22044413587204<br>C -0.43138608521305 0.20955899202637 4.19094620645098<br>H 0.21645329159980 -0.61044178226268 4.47310214087749<br>C -0.25565494737627 -4.31892586677857 -1.54691574681834<br>C -0.26559116738580 -5.42590137757415 -0.71207347559819<br>C 0.88011391579266 -5.76783209520638 -0.01245072712278<br>C 2.03149888948048 -5.00319509716005 -0.13083974035421<br>C 2.03803151425852 -3.89635701735244 -0.96310270147380<br>C 0.89800849040188 -3.55234890640880 -1.68544025822840<br>H -1.15271274609699 -4.06379487121991 -2.09852709674916<br>H -1.17095967129898 -6.01166170886887 -0.62343572677017<br>Br 0.87774115895653 -7.29276486738039 1.10333344535187<br>H 2.92573983332799 -5.25611697343274 0.42248918668557<br>H 2.93524668978059 -3.29754997980991 -1.05636306501363	**RC*anti* (1$_F$ and 2$_{Br}$)**<br>$E_{PBEh\text{-}3c/CPCM(Chloroform)}$=<br>-4761.24393312<br>$ZPE_{PBEh\text{-}3c/CPCM(Chloroform)}$=<br>0.56751479<br>$FEC_{(298.15)PBEh\text{-}3c/CPCM(Chloroform)}$=<br>0.50017829<br>$E_{PW6B95\text{-}D3(BJ)/def2\text{-}TZVPP/CPCM(Chloroform)}$=<br>-4770.95785484899

	O 0.96906398398645 -2.48277344624497 -2.50101320452658<br>H 0.09025821798444 -2.23923688207867 -2.82247769078705	
29	Au -0.80520680769036 -1.08650632760818 0.13895451691593<br>P 0.31862712385404 0.08788717757076 1.75309576591740<br>O -2.78077468380904 -0.64505639012745 -2.89376478996172<br>C -1.60772103939274 -1.14857680676166 -2.48650721656842<br>C -1.75266855537941 -2.13680703275815 -1.51534924550456<br>C -3.19871175957638 -2.24153946059745 -1.36414397030342<br>C -4.03886104744783 -3.04318468422457 -0.59570242349588<br>H -3.63143836135977 -3.78578064047809 0.07818855912809<br>C -5.40749154378788 -2.86333748103921 -0.71658955213517<br>H -6.07974459719863 -3.47619385262144 -0.13078765980710<br>C -5.94231874695894 -1.90471312232654 -1.58362757546286<br>H -7.01584785788367 -1.79088391697478 -1.65296231476513<br>C -5.12370947492768 -1.09864056614145 -2.36192477800087<br>H -5.52836528058889 -0.35888104839698 -3.03954529277202<br>C -3.76609369559271 -1.30567571301720 -2.22272042205341<br>C -0.41103893499939 -0.59975827621115 -3.10849596704666<br>C 0.79879647208675 -1.29315269146754 -3.00708448845211<br>H 0.83578468637999 -2.25365278477881 -2.50810349638218<br>C 1.95879346157492 -0.76528804385059 -3.54213097465877<br>H 2.89603611762930 -1.30041588930906 -3.46764552096263<br>C 1.89843528926666 0.46113049787451 -4.18047631809819<br>C 0.71651633176885 1.16434722199852 -4.31162255803455<br>H 0.69965368979721 2.11952977646662 -4.81905384074027<br>C -0.44101526841257 0.62584144657289 -3.77569304574914<br>H -1.36384105386108 1.18204122434493 -3.86712804211881<br>F 3.02125675871994 0.98383283914276 -4.68410311863799<br>C 1.05090168791287 1.59083831978590 1.04149817377022<br>C 1.64799698525055 1.50850964653761 -0.21683616464782<br>H 1.65625796494412 0.57286724076205 -0.76456080712517<br>C 2.23814175389218 2.62797575243248 -0.78060330461033<br>H 2.69927514716425 2.55538308201540 -1.75687747808926<br>C 2.22885210063633 3.83756594936237 -0.09711445666708<br>H 2.68411404222455 4.71317999471875 -0.54090803667633<br>C 1.63077450138757 3.92408974341090 1.15168109282110<br>H 1.61919538067755 4.86534255494723 1.68504154720584<br>C 1.04282087114791 2.80390958868394 1.72379010958217<br>H 0.57864541826400 2.88452613115343 2.69826582102714<br>C 1.69893483119302 -0.83643457406625 2.47984671668548<br>C 2.91101585030169 -0.21946075069213 2.77852377275138<br>H 3.06564872594092 0.82802774807995 2.55380340320988<br>C 3.93344074481341 -0.95138242569285 3.36551354072584<br>H 4.87473043548174 -0.46897195725484 3.59310793267703<br>C 3.75001641024046 -2.29572035521664 3.65696319978694<br>H 4.55025221388610 -2.86458992398045 4.11186211456094<br>C 2.54271189248294 -2.91370913119644 3.35776194599266<br>H 2.39907230033318 -3.96341751425344 3.57624690703527<br>C 1.51994992473568 -2.18877976333310 2.76679860999221<br>H 0.58509619666722 -2.68174144720626 2.52711527089925<br>C -0.70268515825912 0.63971453432919 3.14595662017220<br>C -1.99150648453753 1.10357970551425 2.88548676974825<br>H -2.38004234057052 1.10423512585937 1.87370227651348<br>C -2.78558677958184 1.56623338867266 3.92257550869999<br>H -3.78476443898111 1.92505847682836 3.71450908369774<br>C -2.30209174947999 1.55960913381523 5.22484404383461<br>H -2.92554244968612 1.91469641473524 6.03486708392474<br>C -1.02284517638707 1.09143451288599 5.48796330926145<br>H -0.64519189176418 1.08080257497969 6.50175788153881<br>C -0.22093890836140 0.63269748552737 4.45205839670276<br>H 0.77494094479296 0.26904430684341 4.66986919609539<br>C -1.44464191601486 -5.43823296005427 -2.86487217715784<br>C -1.55451721223098 -6.51253252004104 -3.73100407226515<br>C -0.49178330780658 -7.39105570659050 -3.88527265772901<br>C 0.68129692506237 -7.19263816708830 -3.16920836723748<br>C 0.79094453260756 -6.11491372264282 -2.30910596987738<br>C -0.26709769743968 -5.19618005377661 -2.11879627331965<br>H -2.29203527659635 -4.77141203893223 -2.75324025554236<br>H -2.47607411114618 -6.65819532384208 -4.28073090010069<br>Br -0.64309827574517 -8.86595955565010 -5.06758255929383<br>H 1.51742653248796 -7.87193100144986 -3.27949477201417<br>H 1.71220897394888 -5.96947995312628 -1.75721586754353<br>O -0.14440633180465 -4.19363845239476 -1.30435000215394<br>H -1.00350800429534 -3.12956956868012 -1.42695643711130	**TS*anti* (1$_F$ and 2$_{Br}$)**<br>$E_{PBEh-3c/CPCM(Chloroform)}$=<br>-4761.19946225<br>$ZPE_{PBEh-3c/CPCM(Chloroform)}$=<br>0.56195545<br>$FEC_{(298.15)PBEh-3c/CPCM(Chloroform)}$=<br>0.49369689<br>$E_{PW6B95-D3(BJ)/def2-TZVPP/CPCM(Chloroform)}$=<br>-4770.91301608289

30	Au -0.47270935476468 -2.82717845385557 0.61738946906776<br>P 0.10306464049757 -1.02082915451798 1.82537247762096<br>O -1.67911251518889 0.46463980579491 -2.11535155368729<br>C -1.32628952885810 -0.73147800947065 -2.66186455289425<br>C -2.16683451574585 -1.71781195037498 -2.25973200058017<br>C -3.12809030010993 -1.09955715414298 -1.39797218291889<br>C -4.25183427354423 -1.52950739295197 -0.68966387735804<br>H -4.55422516755867 -2.56857426682989 -0.70577574732408<br>C -4.97306900560506 -0.58965401558900 0.02655326120732<br>H -5.85127872372491 -0.89816684346248 0.57864205662392<br>C -4.58674407513528 0.75648426383583 0.05652391966902<br>H -5.17016451959386 1.46319351094285 0.63157559157481<br>C -3.46881710043944 1.20271340375627 -0.63178477846217<br>H -3.16403261374911 2.24056480053896 -0.61156730290622<br>C -2.76984274427627 0.25056360583687 -1.35086727983851<br>C -0.18553191497348 -0.73538854935222 -3.56141882123493<br>C 0.36099476219706 -1.94816693521984 -3.98885129669574<br>H -0.04715931266345 -2.88664577224772 -3.63693355474235<br>C 1.43846452306230 -1.97854157269966 -4.85553853319236<br>H 1.85818939032272 -2.92117366762466 -5.18163506857514<br>C 1.97079957940045 -0.78066000739953 -5.29522170457824<br>C 1.45766855655057 0.43718982072075 -4.89411847875637<br>H 1.89088948536532 1.36050073043583 -5.25543263998507<br>C 0.37783916225534 0.45456862918541 -4.02621969112302<br>H -0.02432046511926 1.40929622399095 -3.71732882583175<br>F 3.01589738168845 -0.80199523252385 -6.13327086972351<br>C 0.46044746524590 0.42862766944492 0.79283191256259<br>C 1.35149662257641 0.27406718435921 -0.26861643289743<br>H 1.77968256648656 -0.69721568775534 -0.48895890654291<br>C 1.69970474278968 1.36500198544182 -1.04685308267677<br>H 2.39797420381438 1.24002934180533 -1.86329691647563<br>C 1.14304478518056 2.61097089449779 -0.78589703034858<br>H 1.40990071526564 3.46140707074567 -1.39971306399989<br>C 0.24206201357870 2.76346733315110 0.25736685489154<br>H -0.19687231642848 3.73147721324219 0.46025126959947<br>C -0.09644270460448 1.67628372133219 1.05243630569763<br>H -0.79304452187970 1.80962564254553 1.86968624895989<br>C 1.60632107552777 -1.27414107919054 2.81376379203854<br>C 2.54179367569023 -0.25754017322020 2.98962331801567<br>H 2.40835772098875 0.70204512958955 2.50699310802549<br>C 3.65730403038210 -0.47146103350870 3.78700640518422<br>H 4.38242874014833 0.32098434615759 3.91689221214199<br>C 3.84191012468120 -1.69551107663017 4.41397397399921<br>H 4.71371668800624 -1.86054493865095 5.03343171122625<br>C 2.91028112500867 -2.71096858391259 4.24170723083341<br>H 3.05268049435678 -3.66827566442253 4.72521074057723<br>C 1.79746964365325 -2.50390047390744 3.44166782873104<br>H 1.08002544873591 -3.30488878353415 3.30784753828341<br>C -1.16448502697360 -0.48909384254262 3.00914606329533<br>C -2.50013293918203 -0.52819156470635 2.61457709876290<br>H -2.77295300549185 -0.89409984805997 1.63200162823657<br>C -3.49259917277622 -0.09485433480469 3.47957453830657<br>H -4.52709952274757 -0.12692922152118 3.16424385588718<br>C -3.15873600083980 0.37000752051272 4.74466817152146<br>H -3.93495960354498 0.70063210852747 5.42237217770065<br>C -1.82925613510574 0.40609251544109 5.14199007620674<br>H -1.56562101057767 0.76505007458892 6.12804339446439<br>C -0.83112750015858 -0.01940693112461 4.27708950088113<br>H 0.20150912360234 0.01339340011967 4.59931473430340<br>C -1.12946044939924 -5.40950347735513 -2.58702059395767<br>C -0.52142789903797 -5.82892583731564 -3.75877778461179<br>C 0.85390722800391 -5.71395244567912 -3.90105577228837<br>C 1.61823620897500 -5.19010980070243 -2.86871226948018<br>C 1.00532773022912 -4.76920156295035 -1.70048112374905<br>C -0.38792637400985 -4.85856478758414 -1.52562168193497<br>H -2.20424682622007 -5.50097655103322 -2.48484957274677<br>H -1.12947923450113 -6.23734319775442 -4.55575921874900<br>Br 1.68884377987525 -6.26474622524430 -5.50851544208515<br>H 2.69249011755625 -5.09925775642596 -2.96571665773774<br>H 1.62351836511972 -4.36318102294567 -0.90719109662128<br>O -1.01184549693455 -4.47438990949201 -0.43428760574330<br>H -2.13241629074050 -2.75223477611341 -2.56103214025892	**PC*anti* (1_F and 2_{Br})**<br>$E_{PBEh-3c/CPCM(Chloroform)}$=<br>-4761.25362696<br>$ZPE_{PBEh-3c/CPCM(Chloroform)}$=<br>0.56728954<br>$FEC_{(298.15)PBEh-3c/CPCM(Chloroform)}$=<br>0.50034745<br>$E_{PW6B95-D3(BJ)/def2-TZVPP/CPCM(Chloroform)}$=<br>-4770.96622540434
31	O -1.77222583068957 0.47986651603052 -2.23997963296152<br>C -1.35163927531216 -0.73223026494403 -2.69354280932897<br>C -2.12511749886470 -1.73136921412919 -2.19863293556959	**2-(4-fluorophenyl)benzofuran**<br>$E_{PBEh-3c/CPCM(Chloroform)}$=

	C -3.11026781444123 -1.10550151843410 -1.36972846779868<br>C -4.17938965645164 -1.54531020352229 -0.58725611519837<br>H -4.41831372897787 -2.59818727691441 -0.51279660311546<br>C -4.92624547987945 -0.59886033732346 0.09125223185864<br>H -5.75985869913602 -0.91573596110852 0.70401271949540<br>C -4.62632608303912 0.76647747815333 0.00229363997564<br>H -5.23170551003007 1.47911291276124 0.54640562927011<br>C -3.56932515635001 1.22508809517535 -0.76868404723396<br>H -3.33258866470967 2.27821154204123 -0.84146223147144<br>C -2.83648434145974 0.26236221137202 -1.43794209981941<br>C -0.20215004102432 -0.73571233792263 -3.58308204041122<br>C 0.26668771316227 -1.93981658397678 -4.11524397594631<br>H -0.22517131562445 -2.87209208273785 -3.87093728406225<br>C 1.35973333698422 -1.96944160751208 -4.96141783968839<br>H 1.71799548465165 -2.90469319644479 -5.37055082539370<br>C 1.98783542962545 -0.77856565564049 -5.27757694490117<br>C 1.55312892139736 0.43045479802346 -4.77265234150330<br>H 2.06122143641758 1.34892603554139 -5.03492989699254<br>C 0.45696769381184 0.44703481155775 -3.92515263231635<br>H 0.12240228240393 1.39704645784741 -3.53226975029966<br>F 3.04669300564209 -0.80070810745828 -6.09692656233236<br>H -2.01487643710636 -2.78619785943479 -2.39148525625513	-712.44973686<br>$ZPE_{PBEh-3c/CPCM(Chloroform)}$=<br>0.19643177<br>$FEC_{(298.15)PBEh-3c/CPCM(Chloroform)}$=<br>0.15946596<br>$E_{PW6B95-D3(BJ)/def2-TZVPP/CPCM(Chloroform)}$=<br>-715.176425599389
32	Au -0.80746137073287 -0.42735266132969 -0.15341745666575<br>P 0.34276389695794 0.29273331803388 1.74020220970431<br>O -2.43652734404654 -1.75477760905198 -3.87476217198232<br>C -1.41005820481868 -1.48369990255883 -3.00325805881983<br>C -1.85309918754701 -1.08348921560270 -1.77664728711007<br>C -3.29367628443300 -1.12892569957081 -1.89818687323798<br>C -4.35427069379923 -0.85811671016209 -1.03126637172242<br>H -4.16584075375419 -0.53421372003772 -0.01446251519026<br>C -5.64877907257300 -1.01075111803951 -1.49722684920884<br>H -6.48325627275724 -0.80580549633285 -0.83921122328791<br>C -5.90190696117645 -1.42650455275878 -2.81039906852379<br>H -6.92499705833570 -1.53625005182351 -3.14528894184172<br>C -4.86688475777951 -1.70123073915986 -3.69162290078595<br>H -5.05666347604183 -2.02319232146013 -4.70720609834033<br>C -3.58271075282671 -1.54117556091304 -3.20166719988508<br>C -0.07390818483901 -1.65257510748197 -3.56521031314482<br>C 1.00764708049277 -2.01272743438059 -2.75755527792885<br>H 0.85801622573402 -2.21767512905395 -1.70656448752629<br>C 2.28274863057267 -2.14351754881440 -3.28158889123428<br>H 3.11341288458488 -2.42458274763486 -2.64766876964888<br>C 2.47166780456085 -1.92993844793404 -4.63330851932522<br>C 1.42508747673059 -1.59512260903364 -5.46979115744286<br>H 1.59628214070411 -1.43610881794841 -6.52625748232429<br>C 0.15593365765341 -1.45986400265545 -4.93053194051066<br>H -0.65801816925409 -1.18456127469412 -5.58737121926436<br>F 3.70305076607486 -2.05904614699964 -5.14807499017355<br>C 0.64310630669576 2.08263257500572 1.81602489175092<br>C 1.00605055669829 2.74129581834430 0.64194268121370<br>H 1.07692338552378 2.19502575187414 -0.29155832945132<br>C 1.27775323091004 4.10038747556893 0.66166526102139<br>H 1.55997210328289 4.60493458961374 -0.25286941281234<br>C 1.17836491635596 4.81198622015477 1.85042811141901<br>H 1.38379469616773 5.87438219973501 1.86381271199623<br>C 0.80947702409697 4.16151286665681 3.01933949654471<br>H 0.72757099148450 4.71376677890978 3.94619385229901<br>C 0.54388797004410 2.79912997294185 3.00547955466056<br>H 0.25856770777323 2.30181950561676 3.92364903156022<br>C 1.98001092789950 -0.47599627934751 1.91645078930017<br>C 3.09209700742958 0.23536961501521 2.35774125465236<br>H 3.01386420281728 1.28956709263219 2.59055341262175<br>C 4.31471057137747 -0.40692581393906 2.49971588935403<br>H 5.17656463071629 0.15197129097520 2.83974240629551<br>C 4.43102051073382 -1.75861912961007 2.20795096633378<br>H 5.38531305723689 -2.25641372993918 2.32020201385027<br>C 3.32285419228270 -2.47103298523501 1.76761071639863<br>H 3.40704433138880 -3.52470983970280 1.53617638612985<br>C 2.10275518728435 -1.83200329301339 1.61558814325648<br>H 1.24680484706727 -2.39541597301747 1.26389421273122<br>C -0.53924599952337 -0.11436698639763 3.27673199176183<br>C -1.92450303592069 0.04537453996104 3.30561656788496<br>H -2.45243388236253 0.40597868566600 2.43029379854518<br>C -2.63739548780443 -0.26173791521532 4.45370202215191<br>H -3.71219033502293 -0.13774951412562 4.46809180443860	**RC_{syn} (1_F and 2_{Br})**<br>$E_{PBEh-3c/CPCM(Chloroform)}$=<br>-4761.24500116<br>$ZPE_{PBEh-3c/CPCM(Chloroform)}$=<br>0.56730484<br>$FEC_{(298.15)PBEh-3c/CPCM(Chloroform)}$=<br>0.4994909<br>$E_{PW6B95-D3(BJ)/def2-TZVPP/CPCM(Chloroform)}$=<br>-4770.95904757436

	C -1.97322671124160 -0.73579885590738 5.57862538238739<br>H -2.53107962753774 -0.98378589520226 6.47213213737390<br>C -0.59572644030630 -0.89713578398142 5.55275765069638<br>H -0.07570299207147 -1.27274975786340 6.42375002043780<br>C 0.12212491324920 -0.58812222907714 4.40529261717810<br>H 1.19498049741915 -0.73023261038274 4.39496083033142<br>C -1.48402631294853 -3.48710006213150 1.47689542162070<br>C -1.04610771677381 -3.56761356093538 2.79090516707189<br>C 0.02782573571206 -4.38423479353521 3.10690423587018<br>C 0.66237537081574 -5.12633787311467 2.12054839356250<br>C 0.22985164906283 -5.03658730753593 0.80816872290728<br>C -0.84441081900907 -4.21344734268041 0.47334350208346<br>H -2.33794571708371 -2.86203873825191 1.24282582813037<br>H -1.55149368463563 -2.98962767476220 3.55338011924625<br>Br 0.64356224536318 -4.48756928364522 4.88987682677983<br>H 1.49974002590610 -5.76822143233549 2.36020769335740<br>H 0.72949170480641 -5.60645616895641 0.03508526240624<br>O -1.21936818328401 -4.17173766299625 -0.81591385622418<br>H -1.78832655719938 -3.40923113969505 -0.98855510076990	
33	Au -0.81674119635270 -1.06629412189157 -0.02426224997067<br>P 0.25931304650721 0.05921886930556 1.66380031799137<br>O -2.08395464109215 -0.82706880374868 -3.51661527786097<br>C -1.29142702816429 -1.59970941811078 -2.75542162427189<br>C -1.92209810220187 -2.05075303588204 -1.60052085959239<br>C -3.26343829915444 -1.48932239571507 -1.72081292366404<br>C -4.41494068987626 -1.55511341906836 -0.94004001032165<br>H -4.42643765605119 -2.12277495897884 -0.01812991664208<br>C -5.54210126929596 -0.87559026716318 -1.37318107059090<br>H -6.44860443052149 -0.91448720644088 -0.78358462291668<br>C -5.53497726729035 -0.13787278882703 -2.56177469716541<br>H -6.43258098099871 0.38091281329906 -2.87077353307568<br>C -4.39978405498936 -0.06043744101925 -3.35612413405915<br>H -4.38657820815556 0.50430158180439 -4.27870800490495<br>C -3.29511776779153 -0.75184610035831 -2.89992103896841<br>C 0.04437696676339 -1.85722206684926 -3.27059557587838<br>C 0.78762510200891 -2.92413838544988 -2.75748279799397<br>H 0.35460030428037 -3.57620877567453 -2.01051831598681<br>C 2.06576324350002 -3.17970646817898 -3.21678154384075<br>H 2.63948340730151 -4.00834710668480 -2.82368528830582<br>C 2.59508367470208 -2.36115950222367 -4.19850568181221<br>C 1.88384668434488 -1.30572814926837 -4.73642751212192<br>H 2.32250224538525 -0.68450720115190 -5.50580224258378<br>C 0.60385520601911 -1.05843321683868 -4.26996902411528<br>H 0.05098212684026 -0.22733568333135 -4.68595211610513<br>F 3.83243315710415 -2.60235714308665 -4.64423756042797<br>C 0.30013547107933 1.86191850443089 1.45661472168731<br>C 0.56679007981705 2.37940867789401 0.18976138964050<br>H 0.71340585483449 1.71370862755356 -0.65306938372576<br>C 0.64345609453386 3.75010734115699 0.00110043357566<br>H 0.85101631453275 4.14568144563500 -0.98427360807818<br>C 0.44477846029874 4.61182810463514 1.07253852886252<br>H 0.49769188792959 5.68221799997839 0.92275331126194<br>C 0.17338644894422 4.10018431470460 2.33328281427378<br>H 0.01583576268791 4.76873730572990 3.16925755590576<br>C 0.10277611115623 2.72764166788340 2.52876962090917<br>H -0.10776811540888 2.33924995603225 3.51695805831067<br>C 1.98739287291267 -0.44793837717816 1.87274453575280<br>C 2.96629160267028 0.44833052764195 2.29573923912703<br>H 2.72140449308216 1.48745794004300 2.47517467699233<br>C 4.26853088347185 0.01009886107395 2.48738460211511<br>H 5.02682136571263 0.70994834590902 2.81272952525731<br>C 4.59693002400371 -1.32027756125894 2.26338715078393<br>H 5.61355692595064 -1.65880677319911 2.41487674932824<br>C 3.62277002190835 -2.21462549924288 1.84072193028530<br>H 3.87354385118005 -3.25194321130730 1.66213622438136<br>C 2.32204090450629 -1.78009264927070 1.63832688536589<br>H 1.57227247071482 -2.48593018124781 1.30197184370404<br>C -0.54503491572067 -0.23635335565372 3.26355339533465<br>C -1.93431500202875 -0.12871556039261 3.33252253792695<br>H -2.50805012192262 0.13935568936511 2.45256499379302<br>C -2.59115770440626 -0.37296729631215 4.52688958359056<br>H -3.66883642662375 -0.28935137209224 4.57439751041412<br>C -1.86728201576177 -0.73597342128269 5.65711996350216<br>H -2.38213706602345 -0.93742294244701 6.58736808896759<br>C -0.48702299161420 -0.84716146159786 5.58963792459598	**TS$_{syn}$ (1$_{F}$ and 2$_{Br}$)**<br>E$_{PBEh\text{-}3c/CPCM(Chloroform)}$=<br>-4761.20870837<br>ZPE$_{PBEh\text{-}3c/CPCM(Chloroform)}$=<br>0.56206150<br>FEC$_{(298.15)PBEh\text{-}3c/CPCM(Chloroform)}$=<br>0.49592777<br>E$_{PW6B95\text{-}D3(BJ)/def2\text{-}TZVPP/CPCM(Chloroform)}$=<br>-4770.92446032632

	H 0.07917489672052 -1.13753088478504 6.46449008647694<br>C 0.17676787887471 -0.59649061722012 4.39568799867524<br>H 1.25305076926837 -0.69848484583496 4.35434732400577<br>C -1.55720595359644 -3.72531392831078 1.56634787538798<br>C -0.90702350490881 -3.68678496870418 2.79304397131429<br>C 0.30003427418271 -4.34707934028599 2.95091608633885<br>C 0.86218101560853 -5.04565117960818 1.88729007282705<br>C 0.21933714233756 -5.06923538440755 0.66399705291067<br>C -1.02509158348756 -4.42869672726667 0.46031021380441<br>H -2.52956235655743 -3.25378737402115 1.47466661169120<br>H -1.35508215726995 -3.14373916365296 3.61585099375498<br>Br 1.20061952307221 -4.29972981994139 4.61664228884777<br>H 1.80648074146360 -5.56270456361467 2.00422843746101<br>H 0.66432658112729 -5.61643261964886 -0.15896964613093<br>O -1.62657697521009 -4.46759004015200 -0.69174652070012<br>H -1.76884540686378 -3.23899679819614 -1.14566034532145	
34	Au -0.99876004134884 -2.22049000005055 1.15282957560595<br>P 0.33735077494724 -0.51351346008639 1.92840780861705<br>O -1.31546313358775 0.04502371120704 -2.95933760761879<br>C -0.91520787033778 -1.21635013814446 -2.62652551801685<br>C -1.98228807509066 -2.03373529389523 -2.44320995306357<br>C -3.14613326426850 -1.22976031812792 -2.66515296816322<br>C -4.52464816719388 -1.44498617032494 -2.62781324237124<br>H -4.92810467908198 -2.42025591778050 -2.38780780223958<br>C -5.36168224967198 -0.37859468385603 -2.90582917026899<br>H -6.43400187886509 -0.52150959420970 -2.88280744463932<br>C -4.84961099111448 0.88673452857711 -3.21875439224966<br>H -5.53287384635486 1.69813646879301 -3.43141301558183<br>C -3.48358829002246 1.12258870340572 -3.26186125835917<br>H -3.08179818142203 2.09763402457530 -3.50422839400965<br>C -2.66660848592604 0.04369497043615 -2.98028802704498<br>C 0.51495625404959 -1.44629368007224 -2.51640507538470<br>C 0.98731204849324 -2.71184466045111 -2.15420234833045<br>H 0.29442140158482 -3.51825541247468 -1.94901907352018<br>C 2.34333832076708 -2.95947018844917 -2.05195096268945<br>H 2.70505724656040 -3.94034502106743 -1.77334229461966<br>C 3.23078386580341 -1.93146602600716 -2.31181642831067<br>C 2.80069653159043 -0.66714251953444 -2.65815502465908<br>H 3.51523186861958 0.12262833810831 -2.84731631532206<br>C 1.43910896212814 -0.42905116714116 -2.76135361663381<br>H 1.10724945915654 0.56225232603571 -3.03783991426459<br>F 4.54606966276275 -2.17102342524958 -2.22317235571788<br>C -0.31834538764633 1.13366089914934 1.54355923876894<br>C -0.76030257956385 1.37396520502014 0.24409949702554<br>H -0.72439750150395 0.58484911248208 -0.49509068227573<br>C -1.24414520454009 2.62320720139741 -0.10789059372764<br>H -1.58458057769460 2.80125254512046 -1.11940823708847<br>C -1.29997924622967 3.63698906155341 0.84035892609495<br>H -1.68472847021911 4.61129988084662 0.56892361664303<br>C -0.86812781180917 3.39883080743462 2.13740528667251<br>H -0.91448527580552 4.18576615988948 2.87849032839453<br>C -0.37524942301097 2.15033750662897 2.49224119258756<br>H -0.04148554236137 1.97705046575574 3.50706153354964<br>C 2.05224858254363 -0.51564992600389 1.33918970844571<br>C 2.70936210326349 0.65853536417417 0.98434766131183<br>H 2.18737615725699 1.60658712870367 0.99403402205066<br>C 4.04782061565142 0.61806533858656 0.61785444003553<br>H 4.55417864668340 1.53318363973598 0.34049736586215<br>C 4.73274314875297 -0.58767323005698 0.61032133880937<br>H 5.77676635276230 -0.61546542418678 0.32769585808809<br>C 4.07662375706176 -1.76139582268849 0.95797715225105<br>H 4.60520635965164 -2.70511730071670 0.94292226551762<br>C 2.73870747725701 -1.72898332899260 1.31366720432933<br>H 2.23073821172735 -2.65050602088055 1.57404677937094<br>C 0.46989256617975 -0.58027480524331 3.74120763992440<br>C -0.63174041808710 -0.99928396703222 4.48640218394141<br>H -1.54734949520183 -1.30460970469811 3.99413335611094<br>C -0.56364023351786 -1.03614897116453 5.87041555226368<br>H -1.42155726661254 -1.36898723151019 6.43929650358464<br>C 0.60428125668552 -0.65871100284066 6.51967582822252<br>H 0.65903758177701 -0.69426221319214 7.59981578739714<br>C 1.70402222440838 -0.24374251934622 5.78194195557499<br>H 2.61728855423966 0.04749689788727 6.28374266133680<br>C 1.64018512867262 -0.20387034617921 4.39637459617659<br>H 2.50713393594949 0.11722839019750 3.83345466394740	**PC*syn* (1_F and 2_{Br})**<br>$E_{PBEh-3c/CPCM(Chloroform)}$=<br>-4761.23017552<br>$ZPE_{PBEh-3c/CPCM(Chloroform)}$=<br>0.56680509<br>$FEC_{(298.15)PBEh-3c/CPCM(Chloroform)}$=<br>0.49928648<br>$E_{PW6B95-D3(BJ)/def2-TZVPP/CPCM(Chloroform)}$=<br>-4770.94700322147

	C -2.41586623711399 -3.85713144314048 0.71172727416041<br>C -2.90762269278756 -3.90771258391525 2.06299412359864<br>C -2.31542774653219 -4.71619026306934 2.98608596044662<br>C -1.22897237701309 -5.54420700658430 2.60923232623112<br>C -0.79552999946693 -5.59232878890152 1.31923354797027<br>C -1.46329622459763 -4.86741622054060 0.24864628145519<br>H -3.11044398599881 -3.46984193968096 -0.03334388666457<br>H -3.75125168495466 -3.28046762776093 2.32634683801525<br>Br -2.88434919086264 -4.70264094082092 4.79020929086481<br>H -0.73802883755835 -6.15671354565080 3.35572496627284<br>H 0.00482692158403 -6.27072720834405 1.04872249026617<br>O -1.22787416005024 -5.09035507825882 -0.94622842320699<br>H -1.94851426473336 -3.07998657624947 -2.18005582719967	
35	Au -4.32130407979919 2.85305105717881 -1.03756486693885<br>P -3.48359548866303 2.92422303361366 1.13785873588138<br>O -5.14436555563736 2.91526522737470 -5.22930790103149<br>C -4.31861601907617 2.88054042284018 -4.13001893808720<br>C -5.00669234185141 2.80307963805292 -2.95559672188219<br>C -6.39041852688769 2.77466267986001 -3.36912946521502<br>C -7.60885916736161 2.70234072240478 -2.69071740402112<br>H -7.63430400884315 2.64840975359791 -1.60896798449193<br>C -8.78060868118261 2.69953753403376 -3.42687271140514<br>H -9.73398867872850 2.64268561602402 -2.91782869421521<br>C -8.75718861879150 2.76955367483357 -4.82586891912699<br>H -9.69042385582913 2.76570213885236 -5.37364275358147<br>C -7.56181114409924 2.84439923756569 -5.52401430148349<br>H -7.53793006856495 2.89966801895450 -6.60450909829847<br>C -6.40444952894568 2.84718437499282 -4.76443408694584<br>C -2.89721813188518 2.97740820558332 -4.43799384193341<br>C -1.93620422456703 2.40917500774822 -3.60119085176509<br>H -2.24110580647366 1.83525487727225 -2.73750349777610<br>C -0.58440736552889 2.53671339828938 -3.87160480331620<br>H 0.12693484971555 2.08367731765636 -3.19469752360154<br>C -0.16316631712945 3.22157215582393 -5.00279826391629<br>C -1.11017130201826 3.76972080716505 -5.86259837437621<br>H -0.80510477240581 4.30376713860270 -6.75374783755583<br>C -2.45838173930551 3.64778794271682 -5.58563500546212<br>H -3.17224115910800 4.09646280963449 -6.26261528789011<br>C -1.66495860158515 2.96985180322411 1.15577962881263<br>C -0.97784625162581 2.09099895876195 0.31800087931183<br>H -1.52310697130389 1.40591140876364 -0.32085780120642<br>C 0.40745628595823 2.08530974719573 0.29852224149579<br>H 0.93272479108392 1.40005776311610 -0.35374707686094<br>C 1.11764977688007 2.96312744630762 1.10836908391226<br>H 2.19955817919414 2.96422718959111 1.08709563008712<br>C 0.43834369896061 3.84088942969568 1.94009038439515<br>H 0.98770848108640 4.52707900495390 2.57100378855417<br>C -0.95024579497105 3.84537768158913 1.96715429381511<br>H -1.46754168206806 4.53603342730924 2.62032958034203<br>C -3.93161964002644 1.48795772609982 2.15675753763821<br>C -3.03007214019327 0.89824153810885 3.03869240122609<br>H -2.01802987467051 1.27334028995822 3.12182546830753<br>C -3.42589771564525 -0.17989557276792 3.81813705237620<br>H -2.71983154442004 -0.63590531848341 4.49952199800692<br>C -4.72050961764288 -0.67057743638127 3.72395288713613<br>H -5.02596406836533 -1.51224643729801 4.33166752082945<br>C -5.62259833983783 -0.08482105388430 2.84513758532118<br>H -6.63133160892374 -0.46773353249202 2.76507499420756<br>C -5.22935825887784 0.98761954086069 2.05985774820514<br>H -5.93667379063213 1.43325040924604 1.37009368127840<br>C -4.00751675803549 4.37296385548409 2.10178215539947<br>C -4.10102550984184 5.60263097885669 1.45097828472307<br>H -3.89003626164409 5.67785860108267 0.39058581921547<br>C -4.46503421493534 6.73855304403500 2.15722427368247<br>H -4.53431707527230 7.68934858513498 1.64563683869633<br>C -4.74845541932207 6.65252585628837 3.51414962760243<br>H -5.03956120370305 7.53829192962234 4.06343257135702<br>C -4.66424369150911 5.42916902722615 4.16370221376777<br>H -4.88801187300003 5.35781355806698 5.21991535027168<br>C -4.29272274225458 4.29068210762242 3.46204429451996<br>H -4.23004011865688 3.34263465614612 3.98054172142131<br>C 1.28609553083322 3.37299852984092 -5.33418134053414<br>F 1.63290017934303 4.66153044812145 -5.46710240649807<br>F 1.59497476929269 2.77806347266041 -6.49605025913860<br>F 2.08645303029926 2.84724801166420 -4.40230574024177	**1**$_{\mathbf{CF3}}$<br>$E_{PBEh\text{-}3c/CPCM(Chloroform)}$=<br>-2119.36539725<br>$ZPE_{PBEh\text{-}3c/CPCM(Chloroform)}$=<br>0.48216336<br>$FEC_{(298.15)PBEh\text{-}3c/CPCM(Chloroform)}$=<br>0.41988642<br>$E_{PW6B95\text{-}D3(BJ)/def2\text{-}TZVPP/CPCM(Chloroform)}$=<br>-2126.4333611884

36	Au -1.46917676976020 3.56015424758035 -1.39465013613592<br>P -0.29201700536556 3.93805620681262 0.57953404561842<br>O -2.62522293420344 3.15656150573756 -5.47306435983371<br>C -1.76855353559825 3.05388963152408 -4.40722284791183<br>C -2.36966666322232 3.32574385514640 -3.21224707665243<br>C -3.73719782871052 3.62022022671591 -3.57764023292338<br>C -4.87984374310639 3.98628221324219 -2.86299653940671<br>H -4.84242540602229 4.10504025024400 -1.78647150754491<br>C -6.05780190759547 4.19802643233840 -3.55785049420369<br>H -6.95363533588173 4.48096569655078 -3.02070583898104<br>C -6.11329172413504 4.05513111921962 -4.95006694097101<br>H -7.04932725002871 4.22792420112703 -5.46462676965444<br>C -4.99206238626660 3.70029737656452 -5.68439999208536<br>H -5.02877305561048 3.59315475196004 -6.76054758160855<br>C -3.82636973814443 3.49659401153893 -4.96686441248507<br>C -0.38107405567444 2.75762415902065 -4.74776200674488<br>C 0.41807786224878 1.99393717618937 -3.89219516733871<br>H -0.01033971187524 1.53795375248627 -3.01030718421520<br>C 1.75272338558682 1.77883544069494 -4.17477916975737<br>H 2.34388554261562 1.18227164861926 -3.49120523578120<br>C 2.31844020835819 2.31142601768934 -5.32941663129149<br>C 1.52720808269984 3.03825340824286 -6.20727247623993<br>H 1.93952794182978 3.45430748403687 -7.11601519162824<br>C 0.18832830062288 3.25366875195083 -5.92092481358165<br>H -0.40460977594045 3.83952574999382 -6.61010635732107<br>C 1.40339247104685 4.48632371025203 0.21071273889000<br>C 2.08414706151245 3.86669421889911 -0.83710369660883<br>H 1.60425127398377 3.08942311971580 -1.41951405005725<br>C 3.38096845205736 4.24315387033290 -1.14834756615888<br>H 3.89495982250349 3.76238135246377 -1.97052688888584<br>C 4.00652411167675 5.24480988267288 -0.41715909699387<br>H 5.01656214703896 5.54471535389462 -0.66387499898534<br>C 3.33290690999739 5.86568928183660 0.62526224676674<br>H 3.81665721201810 6.64821676613087 1.19483507595176<br>C 2.03410852936164 5.48990051251565 0.94066708707375<br>H 1.51861132213536 5.98522031368356 1.75348332456878<br>C -0.10247433525815 2.50230247900457 1.67604445540243<br>C 1.09538421939577 2.23915614578515 2.33606144757736<br>H 1.96076100927464 2.86928731786514 2.17621983159596<br>C 1.18676804724859 1.16249429631773 3.20661220516243<br>H 2.12140963900982 0.96111968539057 3.71326182256508<br>C 0.08303055468867 0.35142201528123 3.42893038892205<br>H 0.15423905276834 -0.48507946122342 4.11177087218909<br>C -1.11204987780473 0.60972921531043 2.77080003250077<br>H -1.97340516546784 -0.02161515549805 2.94015046250378<br>C -1.20468692889263 1.67508382507409 1.88824535983664<br>H -2.13891412501692 1.85860376005974 1.36936041815826<br>C -0.99618874247988 5.24484347806354 1.62890800384874<br>C -1.48766131349645 6.39378399612428 1.00948265602370<br>H -1.46526269168762 6.48492308400250 -0.07034982366592<br>C -2.00640876979508 7.42839862284447 1.77164969674110<br>H -2.38393317002980 8.31742010601124 1.28408278130474<br>C -2.04900375907571 7.31878434262059 3.15592135786456<br>H -2.46039068762002 8.12415549577969 3.75018391164437<br>C -1.56902588240097 6.17396749296765 3.77518695456995<br>H -1.60371524310281 6.08328825756144 4.85283297923219<br>C -1.04133129709494 5.13824346680669 3.01591537457172<br>H -0.66879479822916 4.25121437601030 3.51163732997650<br>C -4.03004674718900 0.81589416685663 -0.80208856841997<br>C -4.68547944900481 0.59364756985631 0.39946566758381<br>C -4.34084722890132 -0.50048193057528 1.17699691048978<br>C -3.34737157762576 -1.37483959163451 0.75898985666665<br>C -2.68786076247970 -1.14549402956971 -0.43619677737784<br>C -3.02237223014741 -0.04770604442515 -1.22610400139423<br>H -4.31712365904645 1.66352636062580 -1.41266472025603<br>H -5.46305431120971 1.27781223707020 0.71216838055329<br>Br -5.22457357488915 -0.80260302973386 2.82028792171032<br>H -3.07380302318569 -2.23251042114413 1.35909108970728<br>H -1.90736100480154 -1.82238409877885 -0.75930130749185<br>O -2.35098082548373 0.12434579079848 -2.37697933607904<br>H -2.59320142787345 0.96387224272396 -2.79349631696201<br>C 3.77336816001565 2.08396680363618 -5.58522389203665<br>F 4.53308018753628 2.62647317147997 -4.61923582061269<br>F 4.18402995972941 2.60701190340450 -6.74260732575403<br>F 4.07622724500813 0.77875552530080 -5.61282924104309	**RC*anti* (1_{CF3} and 2_{Br})**<br>$E_{PBEh-3c/CPCM(Chloroform)}$=<br>-4998.59773313<br>$ZPE_{PBEh-3c/CPCM(Chloroform)}$=<br>0.58075169<br>$FEC_{(298.15)PBEh-3c/CPCM(Chloroform)}$=<br>0.50985036<br>$E_{PW6B95-D3(BJ)/def2-TZVPP/CPCM(Chloroform)}$=<br>-5009.13006050915

37	Au	-1.43460518044671	3.45482832632471	-0.91083827186615	**TS$_{anti}$ (1$_{CF3}$ and 2$_{Br}$)**<br>E$_{PBEh\text{-}3c/CPCM(Chloroform)}$=<br>-4998.55395223<br>ZPE$_{PBEh\text{-}3c/CPCM(Chloroform)}$=<br>0.57521822<br>FEC$_{(298.15)PBEh\text{-}3c/CPCM(Chloroform)}$=<br>0.5040117<br>E$_{PW6B95\text{-}D3(BJ)/def2\text{-}TZVPP/CPCM(Chloroform)}$=<br>-5009.08716673926
	P	-0.00733302203416	4.27992610955071	0.67720720124185	
	O	-2.46217584893237	3.91551568907565	-4.35254206424183	
	C	-1.88246267776220	2.99776986183780	-3.56464278887741	
	C	-2.68556767932430	2.61757287045575	-2.49589391074627	
	C	-3.90017468230814	3.39352191566486	-2.70263437773755	
	C	-5.11883512584883	3.48471146565655	-2.03373608573237	
	H	-5.30972000412526	2.89230055661458	-1.14820912630675	
	C	-6.07625436273101	4.35096760214264	-2.53426139505007	
	H	-7.03157787020862	4.43741950276027	-2.03380705618560	
	C	-5.83637864301074	5.11900773772914	-3.67956317056310	
	H	-6.60735586994592	5.78553665628310	-4.04229726975058	
	C	-4.63240657396516	5.04082820661392	-4.36365858689586	
	H	-4.44143294551375	5.62677178728418	-5.25263765153889	
	C	-3.69982427482213	4.16512618371447	-3.84335151630515	
	C	-0.54782021475132	2.55386558053142	-3.95213961040877	
	C	-0.04177738852837	1.36226813492216	-3.43289538707815	
	H	-0.64988744168258	0.75000321148666	-2.77877839795877	
	C	1.23860757502627	0.94733243586623	-3.75401768934856	
	H	1.60530920323864	0.01897462858230	-3.33914963718694	
	C	2.02647267169090	1.71770070662320	-4.59958152645968	
	C	1.52228646097272	2.89921758112788	-5.13289099730314	
	H	2.12235348815038	3.50888030439803	-5.79611321686633	
	C	0.24264032115577	3.31414368090155	-4.81542772512146	
	H	-0.12652443880760	4.24157585084834	-5.23088577179340	
	C	1.54452888208968	4.83453706944706	-0.08655286467385	
	C	2.09432974791092	4.06949936526198	-1.11552300717741	
	H	1.59446371596847	3.17390484977784	-1.46724831399859	
	C	3.29073500163291	4.45073240271744	-1.70118410506447	
	H	3.70910330615069	3.85357534811962	-2.50008389011269	
	C	3.94153169246063	5.59956496304446	-1.26935842275984	
	H	4.87248748707712	5.89979198340148	-1.73205593240281	
	C	3.39520701460474	6.36497047698745	-0.24931703029152	
	H	3.89819503549770	7.26176651597037	0.08731408044395	
	C	2.19884893348386	5.98549078919203	0.34387683923435	
	H	1.78143330368223	6.59192822463319	1.13738680385148	
	C	0.46504140350251	3.04908159009637	1.92207859068556	
	C	1.76090099279568	2.99049396516639	2.42888566232762	
	H	2.52552219465019	3.66504346150735	2.06556497932498	
	C	2.08041282970662	2.05736445851193	3.40482945318149	
	H	3.08909973665902	2.01324540706815	3.79356018607559	
	C	1.11154572798964	1.18339140144748	3.87751343252153	
	H	1.36450119274604	0.45437199075777	4.63610581609109	
	C	-0.18079531174593	1.23845842475340	3.37201939557622	
	H	-0.93666541436855	0.55364995456913	3.73249572841980	
	C	-0.50452365461138	2.16552564442859	2.39388139597703	
	H	-1.51307157636900	2.19359235013587	1.99832083618463	
	C	-0.64914976692709	5.70596589096982	1.59526086490099	
	C	-1.33880455455761	6.69341019150971	0.89247549919604	
	H	-1.50869764632408	6.59060073161541	-0.17306957867284	
	C	-1.81240223380489	7.81421117147713	1.55507960518342	
	H	-2.34626554439177	8.57746010886065	1.00478221207608	
	C	-1.60968046686642	7.95097800782577	2.92272970304299	
	H	-1.98604960636729	8.82349038685573	3.44038516827578	
	C	-0.93078993453373	6.96638026768013	3.62562657883564	
	H	-0.77515070677845	7.06804252881833	4.69141647258993	
	C	-0.44831190455803	5.84445655677017	2.96560967651863	
	H	0.07931842612609	5.08291712703273	3.52504962543128	
	C	-4.59839224360828	-0.44253761346889	-2.45648896351057	
	C	-5.58329623439890	-1.39911629293383	-2.63312861258078	
	C	-5.56271966105283	-2.56402096875675	-1.87977943901444	
	C	-4.55314607920210	-2.76937060371409	-0.94889187349603	
	C	-3.57061259030660	-1.81159114839170	-0.77320327536498	
	C	-3.55332919723222	-0.60999629458909	-1.51800327425388	
	H	-4.63438899335971	0.45737035104604	-3.05963666401774	
	H	-6.36393139095627	-1.22693135173229	-3.36359807119820	
	Br	-6.91133691651634	-3.87486066066725	-2.12129592773419	
	H	-4.52484371729373	-3.67437932017114	-0.35490748712628	
	H	-2.78830260590817	-1.98299619219355	-0.04315862261956	
	O	-2.62276069824658	0.27533866674704	-1.33318871645820	
	H	-2.68329237662200	1.47939586950513	-1.97203229240462	
	C	3.41662664169304	1.30105517080647	-4.96761957962540	
	F	4.31298080509878	2.24866657922840	-4.65065886258094	
	F	3.53545328463447	1.09311628599702	-6.28489145658159	
	F	3.79886807726157	0.18022059888115	-4.35438103714366	

38	Au -1.81183883746604 2.29770554820100 -0.33963532621702<br>P -0.89679672420389 3.93535104864850 0.90461232500195<br>O -0.80227086034596 4.23672014748889 -3.36868131176174<br>C -1.53881414116524 3.16216689444679 -3.76261116205008<br>C -2.86516207285393 3.44874522439765 -3.74408709134419<br>C -2.97669733219949 4.80679800635854 -3.30745894586019<br>C -4.02739782005673 5.70019963054363 -3.08446274550115<br>H -5.05515657848361 5.40116456768666 -3.24407082201890<br>C -3.71781041023534 6.97968474154418 -2.65914830426008<br>H -4.51434085719220 7.69069038500906 -2.48271810690953<br>C -2.39061591332440 7.37664153552959 -2.44694054674247<br>H -2.18644232528651 8.38338343611023 -2.10751044897062<br>C -1.33336758534468 6.50646168898293 -2.65907284069410<br>H -0.30641943768383 6.80422511032895 -2.49426806826607<br>C -1.66428203095524 5.23482044435153 -3.08941274290846<br>C -0.80342504821734 1.96104932024634 -4.11599160121828<br>C -1.48165823636514 0.84012899241536 -4.59209190412549<br>H -2.55849973607097 0.85529708684017 -4.69017719375247<br>C -0.79738178241693 -0.30817862836174 -4.94404819705110<br>H -1.35710270665530 -1.16051573418912 -5.30120283565847<br>C 0.58422016288193 -0.35543517219885 -4.82625707771392<br>C 1.27241169282215 0.75258818950337 -4.34442475843839<br>H 2.35007134730388 0.73392747342072 -4.24178778234834<br>C 0.58736118070224 1.90029290724580 -3.99301366754146<br>H 1.14651990789397 2.75115254962261 -3.62913349059519<br>C 0.37880706123307 4.93032747556905 0.08321135343301<br>C 1.24368663823432 4.30826034194616 -0.81375142605647<br>H 1.12900891413221 3.25582185564369 -1.04413382803826<br>C 2.25474276111126 5.03538923414675 -1.42253544805617<br>H 2.92074044453011 4.54681662115881 -2.12155639091312<br>C 2.40545433572150 6.38691523790075 -1.14144656729698<br>H 3.19153670162695 6.95495058319393 -1.62159021545981<br>C 1.54490218847440 7.01021746041982 -0.24793104050380<br>H 1.65789658268113 8.06372221188458 -0.02872915627469<br>C 0.53230327963813 6.28572697138764 0.36400741173234<br>H -0.13717529409802 6.78426527143776 1.05310356531638<br>C -0.09961219609081 3.27335376372254 2.39862028655666<br>C 1.14118911935705 3.72856817124653 2.83419731330828<br>H 1.67900705732369 4.48249645223289 2.27448508533766<br>C 1.70176841270038 3.21258533080113 3.99505538683795<br>H 2.66856722975282 3.56829315441475 4.32582560286505<br>C 1.02685252457137 2.24630077839678 4.72594704218046<br>H 1.46659302356236 1.84488939883018 5.62952347102726<br>C -0.21215300454319 1.78946402122889 4.29397269730517<br>H -0.74050067097833 1.03260171547772 4.85830700863777<br>C -0.77214104248869 2.29597375536626 3.13241217859380<br>H -1.73599854028172 1.92729340387637 2.80089750601231<br>C -2.12459458138609 5.12965716707716 1.50667769009275<br>C -3.08017958789173 5.60250230878104 0.60901104832654<br>H -3.10115676843802 5.24249125377935 -0.41292704421085<br>C -4.01199667572305 6.54241411858740 1.01919534094495<br>H -4.74896648973675 6.90567927048739 0.31535313819204<br>C -4.00145741441108 7.00854209033052 2.32752754739068<br>H -4.73431737425671 7.73728394637738 2.64839528162362<br>C -3.05328302102068 6.53690373307583 3.22427742956038<br>H -3.04309177912509 6.89657133541710 4.24467481533649<br>C -2.11327803166474 5.60035115054171 2.81692891268268<br>H -1.37829713567593 5.24068894491735 3.52537621884838<br>C -0.95578818693971 -0.76634162647836 -1.43997678971601<br>C -0.50583402536463 -2.01389340035287 -1.83813846651973<br>C -1.36697674774348 -2.88833151955731 -2.48228900233202<br>C -2.68005391396913 -2.51265843794087 -2.72971740428489<br>C -3.12417643361376 -1.26449261751365 -2.33303875739869<br>C -2.27639914831427 -0.35027776547589 -1.67872667298095<br>H -0.25687769413037 -0.10059496921007 -0.94514072024292<br>H 0.51950736063566 -2.29425790862758 -1.63623320479066<br>Br -0.75767570349147 -4.60067334802838 -3.01428729368224<br>H -3.36453686161208 -3.18623738105303 -3.22958036513052<br>H -4.15035392955211 -0.97865357357318 -2.53052206034192<br>O -2.76067183645088 0.82042501023707 -1.33857948166109<br>H -3.66997842278493 2.77833948960351 -3.99781604965299<br>C 1.36222343179534 -1.57394496216062 -5.20660707948975<br>F 2.01945597481297 -2.09288411966945 -4.15894316168212<br>F 2.29436019083168 -1.29275060986926 -6.13094387273776<br>F 0.59490790376752 -2.54229681256959 -5.70956693650873	**PC_{anti} (1_{CF3} and 2_{Br})**<br>$E_{PBEh\text{-}3c/CPCM(Chloroform)}$=<br>-4998.60905567<br>$ZPE_{PBEh\text{-}3c/CPCM(Chloroform)}$=<br>0.58079156<br>$FEC_{(298.15)PBEh\text{-}3c/CPCM(Chloroform)}$=<br>0.51086104<br>$E_{PW6B95\text{-}D3(BJ)/def2\text{-}TZVPP/CPCM(Chloroform)}$=<br>-5009.14226846788

39	O -1.69980853132998 3.44882146798077 -5.79866936615045<br>C -1.75160185136649 2.09168317540582 -5.71588401320855<br>C -3.02402583559274 1.66421950801882 -5.51642748362766<br>C -3.84001622734019 2.83853573402392 -5.46979284057897<br>C -5.20102380503202 3.09890495368041 -5.29772031421380<br>H -5.90838866476003 2.29231696442624 -5.15607881358039<br>C -5.62053100518902 4.41628933984660 -5.31353291204596<br>H -6.67001380486515 4.64434415971283 -5.18243812469640<br>C -4.71241089820023 5.46788749757059 -5.49639866425627<br>H -5.07621969808355 6.48671694362830 -5.50284877859310<br>C -3.35790503237080 5.23256405545234 -5.66926117107308<br>H -2.65268709161770 6.04054676274100 -5.81094560647803<br>C -2.95994077114743 3.90794448502335 -5.65047718180473<br>C -0.49398424981404 1.37730658280657 -5.84881282143085<br>C -0.47227502703781 -0.01920246747658 -5.78466058728339<br>H -1.38932461452287 -0.57388973912893 -5.63779645699131<br>C 0.71337135671075 -0.71324909352558 -5.91003687143173<br>H 0.69638172121606 -1.79419585269903 -5.85661608851882<br>C 1.90906853563890 -0.02623903851066 -6.10511587505812<br>C 1.90031405484275 1.35910621735020 -6.16784776301015<br>H 2.81475288548051 1.91632340567165 -6.31687588415882<br>C 0.70912727636856 2.05574089017451 -6.04199801623456<br>H 0.73110154049327 3.13504394066888 -6.09685639543530<br>H -3.35147040178101 0.64235908817058 -5.41293762283553<br>C 3.18394211659125 -0.80130887174105 -6.20868164997678<br>F 4.20358395844153 -0.05561147694798 -6.64251594117562<br>F 3.06770897490992 -1.83863859601438 -7.04826909217335<br>F 3.54852518035759 -1.31089124730923 -5.02272544697812	**2-(4-(trifluoromethyl)phenyl)benzofuran**<br>$E_{PBEh-3c/CPCM(Chloroform)}$=<br>-949.80450012<br>$ZPE_{PBEh-3c/CPCM(Chloroform)}$=<br>0.20950125<br>$FEC_{(298.15)PBEh-3c/CPCM(Chloroform)}$=<br>0.16835497<br>$E_{PW6B95-D3(BJ)/def2-TZVPP/CPCM(Chloroform)}$=<br>-953.351422663956
40	Au -2.40756841812750 -3.80928478802418 -1.80728182123815<br>P -2.36915339352890 -2.73795008522243 0.26207046955714<br>O -1.87050284387460 -5.47581228819159 -5.70656131627757<br>C -1.44744558602353 -4.82359547909570 -4.57320885185874<br>C -2.41708028047986 -4.75187515682893 -3.61527182213706<br>C -3.54095008515803 -5.43831308134975 -4.21045896531644<br>C -4.83763786957937 -5.73543756916009 -3.78489605069699<br>H -5.18048963369149 -5.42570692230676 -2.80486543867331<br>C -5.67364304938440 -6.43158235483082 -4.63985811791562<br>H -6.68172048836609 -6.67114631945934 -4.32740832313732<br>C -5.23927234500037 -6.83422045711352 -5.90956310184549<br>H -5.91704173654292 -7.37705396201495 -6.55517962433716<br>C -3.95812760182285 -6.55110071913770 -6.35712398970090<br>H -3.61785517669914 -6.85945707467594 -7.33697611202803<br>C -3.14209226207912 -5.85283646170359 -5.48379609665597<br>C -0.07999321578306 -4.32222049915968 -4.64066189908323<br>C 0.67709025531289 -4.11947172856392 -3.48219010068902<br>H 0.27336317384146 -4.37540214852682 -2.51336858613678<br>C 1.96144291838789 -3.61842575442828 -3.55069284901101<br>H 2.51567743580332 -3.47421679918984 -2.63156561079828<br>C 2.53430812080302 -3.32202360503418 -4.78436016150193<br>C 1.80488069539483 -3.53853412792885 -5.94404183124686<br>H 2.22495349791819 -3.32073641842855 -6.91617596931126<br>C 0.51322410641479 -4.03516067084161 -5.87197234179934<br>H -0.03617343478086 -4.18252315295283 -6.79143607967200<br>C -3.20933069752782 -1.12702251650286 0.29011544752559<br>C -3.01295200560546 -0.26425930590093 -0.78790785269406<br>H -2.39201134768845 -0.56639496926095 -1.62348232702635<br>C -3.61081872634276 0.98590921644196 -0.79694088343165<br>H -3.45252785789589 1.65086298831833 -1.63560830265628<br>C -4.41776045378230 1.37845007845161 0.26363748008878<br>H -4.89003692987314 2.35201513010182 0.25267618884269<br>C -4.62227926417382 0.51959321104935 1.33380889729296<br>H -5.25283581158555 0.82073806727032 2.15992270577034<br>C -4.01834079512206 -0.73045372134786 1.35090523898299<br>H -4.18383658482277 -1.39077845089644 2.19240013319408<br>C -0.67691105574065 -2.40481158585269 0.83528646680966<br>C -0.33573811039303 -1.22771410835780 1.49571551614006<br>H -1.07852441240637 -0.45957483704301 1.66892750630756<br>C 0.96639656260251 -1.03111783474648 1.93482799592106<br>H 1.22667863161578 -0.11272460744196 2.44430196526527<br>C 1.92964665655747 -2.00719986236078 1.72177721474959<br>H 2.94399022469481 -1.85057944120405 2.06474392683217<br>C 1.59194600719917 -3.18332462733614 1.06465052204487<br>H 2.33851071302166 -3.94790080905325 0.89428775180290<br>C 0.29521457261484 -3.38031518865020 0.61723596543359<br>H 0.04510136256322 -4.29840157919780 0.09900387228494	**RC*syn* (1_{CF3} and 2_{Br})**<br><br>$E_{PBEh-3c/CPCM(Chloroform)}$=<br>-4998.60050156<br>$ZPE_{PBEh-3c/CPCM(Chloroform)}$=<br>0.58063835<br>$FEC_{(298.15)PBEh-3c/CPCM(Chloroform)}$=<br>0.50982374<br>$E_{PW6B95-D3(BJ)/def2-TZVPP/CPCM(Chloroform)}$=<br>-5009.1356119638

	C	-3.13982823146946	-3.70967851506410	1.58972881612972	
	C	-4.30022686962043	-4.42596164504777	1.29821414050028	
	H	-4.72381628945363	-4.39910770366829	0.30090704176983	
	C	-4.91592797060369	-5.18445961821024	2.28139072149527	
	H	-5.81449038309525	-5.74006617505953	2.04767027621830	
	C	-4.37359841539448	-5.23809278362327	3.55971292979165	
	H	-4.84925111236257	-5.83771444242100	4.32473680957805	
	C	-3.21781304849109	-4.52865410055962	3.85230214025757	
	H	-2.78783225350962	-4.57433390682079	4.84389971034748	
	C	-2.60032816581446	-3.76507250668395	2.87111388628919	
	H	-1.69248330379216	-3.22625544250776	3.10941713433412	
	C	-1.53283971123007	-6.84440907222731	-0.13163133759693	
	C	-1.35991721277185	-6.69197074228203	1.23632013778423	
	C	-0.08273595027944	-6.71795269632979	1.77289806867068	
	C	1.02186316524504	-6.90377253001236	0.95331756679256	
	C	0.84724543195807	-7.05029898047506	-0.41260094658710	
	C	-0.43143480193527	-7.02127231715566	-0.96718079386895	
	H	-2.53635460207428	-6.83712452608657	-0.54110601796013	
	H	-2.22811957520133	-6.55483318310913	1.86702969924521	
	Br	0.16516503809698	-6.48219711776199	3.63063302803176	
	H	2.02204843144585	-6.92559335827159	1.36532253771028	
	H	1.70748164368828	-7.18725989200950	-1.05551166969206	
	O	-0.53862600461118	-7.18076802125317	-2.29679074843502	
	H	-1.41148392541489	-6.90952420675844	-2.60826450597421	
	C	3.92739146380734	-2.78245798888966	-4.82016000923114	
	F	4.36230176036418	-2.55413696383304	-6.06215985113459	
	F	4.80034189989344	-3.62458185215888	-4.24746685866768	
	F	4.02706012464032	-1.62437956595094	-4.15064852908613	
41	Au	-2.00430881182367	-4.39419016162255	-1.64823660625258	**TS$_{syn}$ (1$_{CF3}$ and 2$_{Br}$)**<br>$E_{PBEh\text{-}3c/CPCM(Chloroform)}$=<br>-4998.56253356<br>$ZPE_{PBEh\text{-}3c/CPCM(Chloroform)}$=<br>0.57532833<br>$FEC_{(298.15)PBEh\text{-}3c/CPCM(Chloroform)}$=<br>0.50579269<br>$E_{PW6B95\text{-}D3(BJ)/def2\text{-}TZVPP/CPCM(Chloroform)}$=<br>-5009.0986145395
	P	-2.21123063868362	-2.96804608910180	0.13963773168505	
	O	-2.20186279451695	-4.55937492919896	-5.32433937395309	
	C	-1.35840880590735	-4.86595965370644	-4.32395576602450	
	C	-1.92404264551162	-5.70711272895387	-3.37429196795651	
	C	-3.26354503465820	-5.93790943234126	-3.90023535063928	
	C	-4.35547459940383	-6.68990434721050	-3.47197492532199	
	H	-4.30345577852195	-7.26983532637253	-2.55922366031129	
	C	-5.50552643258952	-6.67383280523260	-4.24323404116924	
	H	-6.36587863411889	-7.25145450032441	-3.93192318544021	
	C	-5.57995889054509	-5.92486433375334	-5.42329182179853	
	H	-6.49406908001253	-5.93532437237974	-6.00172838679052	
	C	-4.50470184118117	-5.17095918408030	-5.86991788920827	
	H	-4.55341177345369	-4.59226229757811	-6.78255663779875	
	C	-3.37126651699872	-5.20980594550022	-5.08140643628475	
	C	-0.01672540808846	-4.30464864353911	-4.41326156811370	
	C	1.01810900614152	-4.87540192297168	-3.66678642632518	
	H	0.83142210279343	-5.75453703535799	-3.06452400172403	
	C	2.28929162920567	-4.34189609402570	-3.71637935515270	
	H	3.07490790783316	-4.80287551414685	-3.13138439538983	
	C	2.55127721859162	-3.23252381349325	-4.51692788169659	
	C	1.53399989357497	-2.67055296364416	-5.27361381575033	
	H	1.71658264042979	-1.81246057574918	-5.90507808813512	
	C	0.25645609946187	-3.20567979609997	-5.22505020014871	
	H	-0.52483457976765	-2.74664504477894	-5.81503212999352	
	C	-3.12712895016083	-1.44490047226741	-0.22898476010089	
	C	-2.86895915750568	-0.78953466229153	-1.43215190504829	
	H	-2.16042697657334	-1.20429878354721	-2.13989319957550	
	C	-3.51929165251680	0.39712275754544	-1.73052875873231	
	H	-3.31421203606798	0.90154649471661	-2.66541658608581	
	C	-4.43781518684660	0.93101288162407	-0.83531159669607	
	H	-4.95028563555208	1.85413876024964	-1.07236482523450	
	C	-4.70177884577410	0.27793251593847	0.35988100444362	
	H	-5.41829262618202	0.68984049491990	1.05814518203520	
	C	-4.04749133383188	-0.90744479810127	0.66653062081425	
	H	-4.25943858763524	-1.40733180970783	1.60291801240198	
	C	-0.61650922021181	-2.42881429496246	0.81283737855658	
	C	-0.46108214315555	-1.17479484472566	1.39979626097668	
	H	-1.28712490541909	-0.47577736631314	1.43226639171045	
	C	0.76187367514655	-0.81339596469288	1.94544618140325	
	H	0.87922639498396	0.16253532588939	2.39755917964267	
	C	1.83019231773369	-1.69979670014610	1.91126299481306	
	H	2.78293144626622	-1.41457571727030	2.33785922963324	
	C	1.67762971400093	-2.94916936478854	1.32579871407775	
	H	2.50746405444894	-3.64276354035479	1.29384859119226	
	C	0.45990349307517	-3.31253795346408	0.77158764075249	
	H	0.35615041028987	-4.28790053335706	0.31207053005824	

	C -3.09719857345793 -3.75664140062784 1.51265236214508<br>C -4.27511554433513 -4.44719786718009 1.22595826166501<br>H -4.64993908329867 -4.49532478759970 0.20983475538011<br>C -4.97054664955329 -5.08450648946361 2.23963831158951<br>H -5.88290254338605 -5.61943504465399 2.01140871676465<br>C -4.48976093986609 -5.04594070683815 3.54370276643052<br>H -5.02787587839733 -5.55320193165896 4.33371698645447<br>C -3.31697200086019 -4.36429299922624 3.82967517581681<br>H -2.93485898637771 -4.33911198132751 4.84141916303981<br>C -2.61972574174314 -3.71740250822478 2.81750485357177<br>H -1.69983399923266 -3.19896210676295 3.05303966469312<br>C -1.55503102492827 -7.10038227244085 -0.07862764673137<br>C -1.37395316189018 -6.77727224863365 1.26044876268009<br>C -0.09210833198646 -6.65767709894699 1.76995795702692<br>C 1.01201740936544 -6.85845570337585 0.94757360405244<br>C 0.82796269224702 -7.16647655010354 -0.38695124332403<br>C -0.46102568399751 -7.32164502448239 -0.94750570523858<br>H -2.56323948890728 -7.25146893809331 -0.44888899955298<br>H -2.23968577956398 -6.62811854564047 1.89352162917286<br>Br 0.17065378583550 -6.21676717214580 3.59293144746997<br>H 2.01676651711846 -6.76467944395731 1.34039368990321<br>H 1.69383547874950 -7.32290717536325 -1.01985265351157<br>O -0.62251234534406 -7.62359932617442 -2.20239513199258<br>H -1.28186180760430 -6.65073105752469 -2.78858453398534<br>C 3.94085284716107 -2.67630085770350 -4.53738067387698<br>F 4.06321979846984 -1.62373968201010 -5.34909914743685<br>F 4.83114168982066 -3.59204965992943 -4.94139210576045<br>F 4.32784499320147 -2.28028149063987 -3.31711915178971	
42	Au -1.95521082799933 -5.73782998887142 -0.38586924412551<br>P -2.00042306322088 -3.59280317230815 0.44093231404318<br>O -2.31132481492664 -3.29782972635351 -4.72677130043586<br>C -1.35089666453614 -4.08693662549419 -4.16842800882592<br>C -1.79653537142596 -5.35802310807241 -4.00003301095901<br>C -3.14457876412677 -5.37376992091434 -4.47952420379397<br>C -4.15179474782777 -6.33553683471175 -4.57880240793213<br>H -3.98361927308681 -7.35137723602319 -4.24495508417087<br>C -5.36842973054091 -5.95479080454810 -5.11490814750658<br>H -6.16512791776001 -6.68164541729610 -5.20198762950826<br>C -5.59249768935349 -4.64274486249050 -5.55293739732535<br>H -6.55528502433195 -4.38077405965524 -5.97109728142413<br>C -4.60705819134960 -3.67193347614379 -5.46424904201501<br>H -4.77389521516669 -2.65822144558617 -5.80398905640627<br>C -3.39976750365232 -4.07229136775400 -4.92005493319376<br>C -0.07262755598357 -3.46667556181649 -3.87123543748526<br>C 0.92041105041125 -4.21078743749132 -3.23090683859716<br>H 0.73245312251933 -5.24313750911359 -2.96394498515301<br>C 2.15311217485468 -3.65067594469191 -2.95444703685346<br>H 2.90027552862885 -4.25348682855548 -2.45733594920036<br>C 2.41520289144710 -2.33411474141012 -3.31397359044383<br>C 1.43239858165569 -1.58455136619676 -3.94860458715210<br>H 1.61725759048837 -0.55762387225274 -4.23600234693102<br>C 0.19664581778250 -2.14208369094220 -4.22114389130493<br>H -0.55134175859527 -1.53883005925935 -4.71652288347128<br>C -3.19947340503386 -2.47853715396927 -0.33855710767858<br>C -3.76780438595852 -2.81418635695979 -1.56384611275448<br>H -3.51201741250564 -3.74821617393814 -2.04891582359656<br>C -4.68262433807978 -1.95948402092972 -2.16314337846482<br>H -5.12782683232762 -2.23289313164547 -3.11039888076370<br>C -5.03124132936010 -0.76891365388429 -1.54349877660686<br>H -5.74715844104315 -0.10542332027164 -2.01069750986757<br>C -4.46820378038261 -0.43102383787524 -0.31884028418004<br>H -4.74242052233399 0.49459788308883 0.16957748755987<br>C -3.55829083953129 -1.28325482632990 0.28509121336198<br>H -3.13397845437109 -1.01426148340608 1.24447933592752<br>C -0.37398385772042 -2.79882202722551 0.30778586628574<br>C -0.18325752503247 -1.58578003257082 -0.34379048475957<br>H -1.01212583370663 -1.07796077699933 -0.81887283399177<br>C 1.08266516059730 -1.01614604399696 -0.38761550319853<br>H 1.22517389403642 -0.07344779875145 -0.89826815305747<br>C 2.15524176596669 -1.64993943936479 0.21958432307077<br>H 3.13712876029117 -1.19705302012990 0.19581017655668<br>C 1.97016329387832 -2.87158048371770 0.85546450421328<br>H 2.80721434179696 -3.37607800245083 1.31953897578938<br>C 0.71315912040840 -3.45038799367330 0.89233763455002<br>H 0.57906531375833 -4.40646612729109 1.38593271447214	**PC*syn* (1_{CF3} and 2_{Br})**<br>$E_{PBEh\text{-}3c/CPCM(Chloroform)}$=<br>-4998.58593124<br>$ZPE_{PBEh\text{-}3c/CPCM(Chloroform)}$=<br>0.58023182<br>$FEC_{(298.15)PBEh\text{-}3c/CPCM(Chloroform)}$=<br>0.50978105<br>$E_{PW6B95\text{-}D3(BJ)/def2\text{-}TZVPP/CPCM(Chloroform)}$=<br>-5009.12307500738

	C -2.40220027890491 -3.54457327646263 2.21430528220492<br>C -3.39479288865147 -4.39755920423011 2.69449270937314<br>H -3.88772727651433 -5.09576247226808 2.02823553563934<br>C -3.75627492899224 -4.36164119225583 4.03208246444015<br>H -4.52528913527599 -5.02900592629756 4.39769842286851<br>C -3.12587661041577 -3.47927015816625 4.89970173012886<br>H -3.40390562141249 -3.45680111177872 5.94524332570694<br>C -2.13497663225390 -2.63117130785663 4.42630839903975<br>H -1.63947543713099 -1.94423770183449 5.09954760982522<br>C -1.77183707597628 -2.66102785524589 3.08712953219972<br>H -0.99469181707344 -1.99638763291223 2.73191714667953<br>C -1.84994320483027 -7.81498402025723 -1.10103759953139<br>C -2.49713413175501 -8.40699326701295 0.03931393674403<br>C -1.76810718970242 -8.82240405712401 1.11116359611665<br>C -0.35487265908858 -8.70703242829201 1.09559014045657<br>C 0.30115057656820 -8.23220850694362 0.00187894892977<br>C -0.39164290674730 -7.88621017684757 -1.22987846544056<br>H -2.41683862704280 -7.83590621994397 -2.03265917888685<br>H -3.57616034272983 -8.50505594931085 0.02320880724659<br>Br -2.61855617133760 -9.51784282322990 2.65098604736227<br>H 0.21606248985620 -9.00966130265203 1.96502342296614<br>H 1.38404512040647 -8.19730732072787 0.00035178405694<br>O 0.21387741299363 -7.65655635704541 -2.28518731494374<br>H -1.22876969030670 -6.19121345214902 -3.61263408389886<br>C 3.75842266540477 -1.72282355695790 -3.07610386652720<br>F 3.66528044537916 -0.46255736776443 -2.62711019947691<br>F 4.48228084957664 -1.66837224778615 -4.20439670117991<br>F 4.48369339200914 -2.40374830127579 -2.18402862328413	
43	C -0.29917599465823 -4.33720591839114 -1.52851572938678<br>C -0.31461959128432 -5.47471678177168 -0.73564440069641<br>C 0.84828641682197 -5.89193071109285 -0.11134617102860<br>C 2.02692733388967 -5.17889468227205 -0.27314638962459<br>C 2.04164642307418 -4.04382143586547 -1.06504811532934<br>C 0.87819798324317 -3.61417559791033 -1.70032378271956<br>H -1.21512945466991 -4.01913549221646 -2.01346136903608<br>H -1.23509869615745 -6.02869334222211 -0.61053919300591<br>H 2.93552429231827 -5.50476904317992 0.21488316195456<br>H 2.96105841022761 -3.48699266706397 -1.19304949015927<br>O 0.95322882118144 -2.50488917667186 -2.45978751393001<br>H 0.09973335693876 -2.30798999208958 -2.86070378475230<br>Cl 0.83253552307483 -7.31564778125256 0.87978739771430	**4-chlorophenol (2$_{Cl}$)**<br>$E_{PBEh\text{-}3c/CPCM(Chloroform)}$=<br>-765.909582805647<br>$ZPE_{PBEh\text{-}3c/CPCM(Chloroform)}$=<br>0.09823172<br>$FEC_{(298.15)PBEh\text{-}3c/CPCM(Chloroform)}$=<br>0.06719189<br>$E_{PW6B95\text{-}D3(BJ)/def2\text{-}TZVPP/CPCM(Chloroform)}$=<br>-767.963440729346<br>$E_{B3LYP\text{-}D3(BJ)/def2\text{-}TZVPP/CPCM(Chloroform)}$=<br>-767.007133015640
44	Au 0.38123175996666 -4.14543827698326 -2.29675566568261<br>P 1.11581428449495 -3.85523478210021 -0.10684848734563<br>O -0.75407825402471 -3.80955935752608 -6.40011962335662<br>C -0.29368725178989 -3.32654878736415 -5.19811121674274<br>C -0.16427701659846 -4.30708298437564 -4.25584622300301<br>C -0.57427663474579 -5.50869101319956 -4.94669123361995<br>C -0.68277830727612 -6.85309982962102 -4.58517114658417<br>H -0.42710469129445 -7.17601720453148 -3.58282725366924<br>C -1.12164125957352 -7.76368964588940 -5.53181083385202<br>H -1.20976456246218 -8.80982253911632 -5.26855333330790<br>C -1.45766429587595 -7.35574341439344 -6.82825755974323<br>H -1.79807155853554 -8.09055698024111 -7.54595283230057<br>C -1.36440051531258 -6.02551786472327 -7.21108127404423<br>H -1.62448820296954 -5.70358924081155 -8.21106070490991<br>C -0.92258461952685 -5.13641066016870 -6.24749320833208<br>C -0.08160344414913 -1.88267058383923 -5.16915746753250<br>C 0.86360514707838 -1.29812693841586 -4.32925628689255<br>H 1.50387639546844 -1.91653815252690 -3.71533948756046<br>C 1.04104849305982 0.07605582735003 -4.27034873138999<br>H 1.79151636615132 0.47006787169533 -3.59832245198729<br>C 0.27279921809614 0.91071104592063 -5.07791898944339<br>C -0.66280163532657 0.33976400708954 -5.94293133475607<br>H -1.26105022232941 0.98187646315679 -6.57706744916754<br>C -0.83337585942064 -1.02896347234950 -5.98671591990162<br>H -1.57772841647566 -1.43425087926436 -6.65919719093612<br>C 2.00965332431587 -2.27807867553169 0.05927462659919<br>C 2.94412031525746 -1.94506006245641 -0.92232117182913<br>H 3.12841083690544 -2.61393177394640 -1.75520067561809	**RC$_{anti}$ (1$_{OMe}$ and 2$_{Cl}$)**<br>$E_{PBEh\text{-}3c/CPCM(Chloroform)}$=<br>-2663.166923159743<br>$ZPE_{PBEh\text{-}3c/CPCM(Chloroform)}$=<br>0.60998277<br>$FEC_{(298.15)PBEh\text{-}3c/CPCM(Chloroform)}$=<br>0.54164830<br>$E_{PW6B95\text{-}D3(BJ)/def2\text{-}TZVPP/CPCM(Chloroform)}$=<br>-2671.560669489744<br>$E_{B3LYP\text{-}D3(BJ)/def2\text{-}TZVPP/CPCM(Chloroform)}$=<br>-2667.564646471089

	C 3.64585106574857 -0.75301384358726 -0.84080629372901<br>H 4.37004960461212 -0.50408434036111 -1.60531658082097<br>C 3.41394989655214 0.12067667863634 0.21471744292757<br>H 3.95699374849379 1.05484476360181 0.27394151950553<br>C 2.48325042493306 -0.20488228028080 1.19000799229020<br>H 2.29818337372281 0.47282890930987 2.01295987383736<br>C 1.78261298870443 -1.40191470501987 1.11586499133675<br>H 1.05867352976149 -1.64253343628632 1.88354691590650<br>C 2.28272160216038 -5.12785118286879 0.45483908175946<br>C 3.42833920092255 -4.81717211367837 1.18160949460034<br>H 3.66741220697685 -3.78809155239129 1.41734199841311<br>C 4.27564689734187 -5.83168895305101 1.60644060165426<br>H 5.16753048263923 -5.58427727317357 2.16710493150640<br>C 3.98219587049208 -7.15507947671181 1.31164969844943<br>H 4.64808783489318 -7.94344579897715 1.63672387727110<br>C 2.83865674888617 -7.46775299366045 0.58868309507958<br>H 2.61438639713634 -8.49743098704908 0.34568135039415<br>C 1.99443042794840 -6.45887082157741 0.15596143229604<br>H 1.11258334804090 -6.71021623285162 -0.42184056921585<br>C -0.18210314474708 -3.80439335044270 1.16345623027720<br>C -1.40271916716898 -3.21189800446074 0.84346697877048<br>H -1.57596211252482 -2.82106251807703 -0.15241963611189<br>C -2.40374794854310 -3.11940865385157 1.79825208104158<br>H -3.34820687776057 -2.65755694586033 1.54273346918684<br>C -2.19635420706218 -3.62670949340240 3.07420190924792<br>H -2.98047835326625 -3.56024042196784 3.81702832503278<br>C -0.98559241346727 -4.22493424807238 3.39436711590578<br>H -0.82251490034631 -4.62535409431438 4.38625533974317<br>C 0.02127196685531 -4.31369592944556 2.44348609505374<br>H 0.96112749717350 -4.78313661311894 2.70453877849775<br>C 2.95512802228159 -6.50330648408692 -3.83777358838124<br>C 3.38826069996577 -7.66864972058530 -3.22400027409329<br>C 4.49233100923498 -7.63599750156095 -2.39014716540747<br>C 5.16219456238636 -6.44566771541991 -2.15561074750767<br>C 4.72765717099850 -5.28155271017520 -2.76634485466187<br>C 3.62555827773349 -5.30292311373198 -3.61802348465938<br>H 2.09599387421046 -6.54026025663557 -4.49720540366026<br>H 2.86176979717029 -8.59614528165090 -3.40506768932543<br>H 6.01772331818711 -6.41925375491301 -1.49465919143436<br>H 5.24807262693100 -4.34987377749571 -2.58360432659900<br>O 3.25999756775540 -4.14564341021957 -4.20189666338466<br>H 2.40047915074189 -4.23936969331861 -4.63535227152477<br>Cl 5.04042475021788 -9.09569441428387 -1.62809893241103<br>O 0.36688165750359 2.25203831903093 -5.09661776421163<br>C 1.29637251997121 2.87394631418237 -4.23618174574353<br>H 1.08859324603828 2.65983286193761 -3.18413727755159<br>H 2.32459519802603 2.57535468622744 -4.45858347877567<br>H 1.20138515786467 3.94466066478205 -4.39985347658295	
45	Au 0.67075428968098 -4.30565576966881 -1.76393931471630<br>P 0.85164314708798 -3.36992856493673 0.31987066619311<br>O -1.04940240655019 -3.90650443741889 -4.97586438731046<br>C 0.17538325675250 -3.83257614381691 -4.43586698188272<br>C 0.56064266688552 -5.01955014626457 -3.81067965102759<br>C -0.57379445529840 -5.90354272019935 -4.05341197053214<br>C -0.85194572025184 -7.23272953219738 -3.74673070470979<br>H -0.13557044159496 -7.83734819774127 -3.20549858083465<br>C -2.06865684921718 -7.75959722608977 -4.15126395340119<br>H -2.30473642872790 -8.79109486828743 -3.92518166213486<br>C -3.00103866421774 -6.98576593674335 -4.84917473751914<br>H -3.94126005971867 -7.42833752882818 -5.14982792940660<br>C -2.74207582330894 -5.66007678246239 -5.16962014374682<br>H -3.45415140173468 -5.05511349574963 -5.71478680767615<br>C -1.52050546082641 -5.16734303350384 -4.75801552387079<br>C 0.87863783784685 -2.57138857601734 -4.58335528950033<br>C 2.25711667794797 -2.51698730120171 -4.37640032555378<br>H 2.80887667873949 -3.42372250382849 -4.16011606232884<br>C 2.94413355498754 -1.31831772209776 -4.44549651822909<br>H 4.01277005506650 -1.32382454528924 -4.28155566308628<br>C 2.25419747513255 -0.13708367408798 -4.72420352701934<br>C 0.87604368653327 -0.18630152223059 -4.95298034582029<br>H 0.34164688212063 0.72841992367103 -5.17503807243256<br>C 0.20090342612521 -1.38580045504254 -4.88813508612432<br>H -0.86776031389610 -1.39016777497095 -5.05609462474414<br>C 0.75164167500067 -1.55855243674906 0.20844248113547<br>C 1.38150509555634 -0.91757728218887 -0.85870797060035	**TS*anti* (1$_{OMe}$ and 2$_{Cl}$)**<br>$E_{PBEh-3c/CPCM(Chloroform)}$=<br>-2663.123750113634<br>$ZPE_{PBEh-3c/CPCM(Chloroform)}$=<br>0.60450135<br>$FEC_{(298.15)PBEh-3c/CPCM(Chloroform)}$=<br>0.53589482<br>$E_{PW6B95-D3(BJ)/def2-TZVPP/CPCM(Chloroform)}$=<br>-2671.517424124228<br>$E_{B3LYP-D3(BJ)/def2-TZVPP/CPCM(Chloroform)}$=<br>-2667.524975976029

	H 1.90401427334530 -1.48888052226185 -1.61753422332203<br>C 1.34107579362092 0.46375721439230 -0.96155840936005<br>H 1.82766165518796 0.95311485227152 -1.79514406735169<br>C 0.66750432952918 1.21312547822760 -0.00533371134193<br>H 0.63009231867375 2.29121975643217 -0.09063677967189<br>C 0.03649521285957 0.57861275958037 1.05498868636925<br>H -0.49192095662464 1.15900148734411 1.79965277062998<br>C 0.07701864920707 -0.80464534619893 1.16484977023652<br>H -0.42163588421105 -1.28787209877300 1.99524244772498<br>C 2.42785910659911 -3.72926449446350 1.14203542648442<br>C 3.09969290348624 -2.76159296987972 1.88453283932646<br>H 2.70463315228522 -1.75707091967512 1.96523547866537<br>C 4.28851057473225 -3.08233295593991 2.52451262044641<br>H 4.80890678862317 -2.32638395312268 3.09750336832941<br>C 4.80811048177803 -4.36552149193914 2.42820417582049<br>H 5.73679773457664 -4.61192898467987 2.92597873416664<br>C 4.14074032651418 -5.33196861030029 1.68688976806968<br>H 4.54673242016563 -6.33121657766076 1.60339129076353<br>C 2.95605630697921 -5.01546536565479 1.04089027964404<br>H 2.44813791107227 -5.77280134953019 0.45526011934421<br>C -0.44510353402625 -3.86417311851353 1.48812041669592<br>C -1.75849069001878 -3.94354131866968 1.02619302426554<br>H -1.98680217343519 -3.73984707340255 -0.01358702883348<br>C -2.78100711418153 -4.28526967790923 1.89640050319526<br>H -3.79814907349569 -4.34452375919576 1.53260386828982<br>C -2.49785113940425 -4.55991939286224 3.22860900414242<br>H -3.29626797969955 -4.83354942477910 3.90579447162006<br>C -1.19113406857983 -4.48967962209048 3.68891779280315<br>H -0.96676890035900 -4.70701929779636 4.72475597942983<br>C -0.16376956557119 -4.14066890890602 2.82282874469441<br>H 0.85156144424678 -4.08872234705432 3.19395194101086<br>C 2.72682196090629 -7.03945862209147 -5.82952628638518<br>C 3.27256837853647 -7.74489383164407 -6.88841631318742<br>C 4.63626702939209 -7.99048267676806 -6.93074430726757<br>C 5.45424431525299 -7.52953729473744 -5.90957254602506<br>C 4.90941228215300 -6.82085731217926 -4.85361664900292<br>C 3.52412326972839 -6.54883357908995 -4.76856181074014<br>H 1.65690514033376 -6.86631535833094 -5.81591005221032<br>H 2.62924604981673 -8.10475076752031 -7.68154146776793<br>H 6.51989085075006 -7.71962707894301 -5.93704098744140<br>H 5.55882336126292 -6.46490658794167 -4.06242108968060<br>O 3.03410700815141 -5.88445034667162 -3.76682030102113<br>H 1.73679166150961 -5.44363335509627 -3.81838676355200<br>Cl 5.32228008576760 -8.88163585036664 -8.26321365130127<br>O 2.82254571356479 1.07379032599627 -4.78706235153668<br>C 4.21452623980159 1.18644293695852 -4.56447526995112<br>H 4.49471287048698 0.85062445024262 -3.56274406643891<br>H 4.79195734750866 0.62321882730671 -5.30173644655655<br>H 4.45655749508004 2.24160878982975 -4.66133673634132	
46	Au 2.14375326189318 -5.29391115802554 -1.95779064032854<br>P 1.30144038068013 -4.34788325433935 -0.09798531663429<br>O -1.26683874480178 -2.42103460914357 -3.38814801226179<br>C -0.37768664042072 -3.14362063762487 -4.12247904817378<br>C -0.79165625310982 -4.43121840273526 -4.25531037881505<br>C -2.03605407675310 -4.52858136990254 -3.55513813303710<br>C -2.96728572613535 -5.54013414318815 -3.31195385260083<br>H -2.82006853854544 -6.53771362174973 -3.70533070713976<br>C -4.08275187277607 -5.23488623576877 -2.55128046627861<br>H -4.81822144545876 -6.00316924686533 -2.35071723328115<br>C -4.28087937787815 -3.94995588161353 -2.03125391122954<br>H -5.16147538791945 -3.74774857459648 -1.43618688268027<br>C -3.37162198057422 -2.92924848795304 -2.26591968467990<br>H -3.51814867367259 -1.93346725426871 -1.86911533480384<br>C -2.26856484061924 -3.25286703602826 -3.03348684475732<br>C 0.79609926900197 -2.44622550849490 -4.61222544005727<br>C 1.85356814634362 -3.16792840972912 -5.16070027947174<br>H 1.79664590918075 -4.24588134432988 -5.22585897858264<br>C 3.00049751493509 -2.54375965814672 -5.61934281607767<br>H 3.79683245115501 -3.15685925557976 -6.01893016717098<br>C 3.10904113533560 -1.15604907209404 -5.54443446395827<br>C 2.04915650852392 -0.41863275402633 -5.01054079609919<br>H 2.12679859539230 0.66008728957032 -4.95781697502255<br>C 0.91387587685547 -1.05403449366096 -4.55007106671969<br>H 0.11361996678741 -0.45382333877628 -4.13921633538680<br>C 0.67900263203051 -2.66192244755938 -0.34914467620238	**PC*anti* (1$_{OMe}$ and 2$_{Cl}$)**<br>$E_{PBEh\text{-}3c/CPCM(Chloroform)}$=<br>-2663.166923159743<br>$ZPE_{PBEh\text{-}3c/CPCM(Chloroform)}$=<br>0.60998277<br>$FEC_{(298.15)PBEh\text{-}3c/CPCM(Chloroform)}$=<br>0.54164830<br>$E_{PW6B95\text{-}D3(BJ)/def2\text{-}TZVPP/CPCM(Chloroform)}$=<br>-2671.560669489744

	C 1.47017168539257 -1.76581618011979 -1.06634479596328<br>H 2.40143264866913 -2.09166761409717 -1.51519827384462<br>C 1.06897340701466 -0.44788435826381 -1.20986577310353<br>H 1.69021789801817 0.24308713083259 -1.76356439998825<br>C -0.13228319585885 -0.02212041252352 -0.65772021330435<br>H -0.44788505946189 1.00614443558837 -0.77715841751885<br>C -0.93051946219686 -0.91654069533537 0.04042830989565<br>H -1.87009541151429 -0.58989008666327 0.46628414278462<br>C -0.52564637619049 -2.23444654856448 0.20118991062123<br>H -1.15324413896578 -2.92083975438016 0.75430366442384<br>C 2.54524979619114 -4.19492291606329 1.21861738794266<br>C 2.59607715638315 -3.07553686842809 2.04504578580201<br>H 1.91026757944268 -2.25137262004001 1.89775465061127<br>C 3.53374733208986 -3.00864297800703 3.06618142979123<br>H 3.57045686549535 -2.13421025062019 3.70231423396798<br>C 4.41935015524691 -4.05706962790465 3.26967171133649<br>H 5.15067654494167 -4.00144147626314 4.06532745206294<br>C 4.37028939899910 -5.17598762326503 2.44829632323931<br>H 5.06147546343737 -5.99417837198988 2.60104076660849<br>C 3.43999258009628 -5.24451142015129 1.42313740968963<br>H 3.41482252913838 -6.11929363494231 0.78405889816158<br>C -0.07051548420366 -5.27316300743033 0.64800336756145<br>C -1.08244642982104 -5.74866901039079 -0.18450274923995<br>H -1.03517446016813 -5.58740483480273 -1.25487532094846<br>C -2.16397085558022 -6.42666644123996 0.35463292637205<br>H -2.94662761403493 -6.78725732631255 -0.29895328218122<br>C -2.23731724329813 -6.64289495628507 1.72475244142919<br>H -3.07915559220323 -7.17837285393791 2.14385806109054<br>C -1.22921312582715 -6.17517523797266 2.55522295771556<br>H -1.28162391101261 -6.34340964080550 3.62275640905857<br>C -0.14761528194568 -5.48822518717348 2.02128214338038<br>H 0.63036528005911 -5.12677480708272 2.68087104288995<br>C 4.04900044907332 -6.10401918633791 -5.64854557393740<br>C 5.10711522566673 -5.66124519228165 -6.42369899675886<br>C 6.09638996845057 -4.87382747873432 -5.85441864991862<br>C 6.03297204821173 -4.54459171359563 -4.51015294542899<br>C 4.97116374176272 -4.98608571977763 -3.73829376794869<br>C 3.93888604860099 -5.76776681617862 -4.28645653979444<br>H 3.27608505018380 -6.71191049867807 -6.10309850658242<br>H 5.15313481199562 -5.92507926333906 -7.47238495705041<br>H 6.80683406234905 -3.93657995153265 -4.05948396189166<br>H 4.94373720443823 -4.71009159074053 -2.68975708514369<br>O 2.89837565215706 -6.19773656615127 -3.60661269437792<br>H -0.28582214473700 -5.21397185021136 -4.79722345743109<br>Cl 7.40743988342015 -4.28005775984028 -6.83299690697085<br>O 4.17940595065205 -0.45674871214440 -5.95492682156335<br>C 5.29565978629888 -1.16557424342226 -6.45455014243678<br>H 5.71754788948744 -1.84039195210894 -5.70569560023917<br>H 5.04898270579854 -1.74579633235331 -7.34752873535613<br>H 6.04234639655365 -0.42028951858781 -6.71730145595319	
47	Au 3.07323775027812 -4.29453371797018 -1.64555034455228<br>P 4.40999565547994 -3.51044115955147 0.09364309831683<br>O 0.89947420391984 -6.05544749207576 -4.88034776404110<br>C 1.84897062488019 -6.06363183881061 -3.88704980980617<br>C 1.83340437913667 -4.92151894375275 -3.13902877493046<br>C 0.75925627982435 -4.14316536690328 -3.71617165429495<br>C 0.19957418406157 -2.89474663414478 -3.43698555799442<br>H 0.58194332004559 -2.28881141903524 -2.62383464265952<br>C -0.85187860563925 -2.44713494654689 -4.21906451958976<br>H -1.29825374378670 -1.48232376965494 -4.01575991753352<br>C -1.35137972564045 -3.22108317711251 -5.27342846900738<br>H -2.17377368195640 -2.84389909747696 -5.86693864824879<br>C -0.81245138466426 -4.46404485633052 -5.57236532626220<br>H -1.19467365219916 -5.06723874604981 -6.38558841104106<br>C 0.23584719420688 -4.88872959112043 -4.77579528132379<br>C 2.67643052119480 -7.26335557679304 -3.84794070258197<br>C 3.25084228414315 -7.71271214639919 -2.65333704068304<br>H 3.05211189360955 -7.19317769406917 -1.72561592513011<br>C 4.05276594796346 -8.83388926939312 -2.62009432662464<br>H 4.48291790822515 -9.16597129886469 -1.68356738052079<br>C 4.29971311650942 -9.56271283900773 -3.78577220364114<br>C 3.71950810905610 -9.14272278164167 -4.97964205625782<br>H 3.88025237771107 -9.67856925836428 -5.90514800568331<br>C 2.91889211225229 -8.00925989871844 -5.00033031346498<br>H 2.49259832516890 -7.70316415052441 -5.94653336919195	**RC_{syn} (1_{OMe} and 2_{Cl})**<br>$E_{PBEh\text{-}3c/CPCM(Chloroform)}$=<br>-2663.167694751678<br>$ZPE_{PBEh\text{-}3c/CPCM(Chloroform)}$=<br>0.60975782<br>$FEC_{(298.15)PBEh\text{-}3c/CPCM(Chloroform)}$=<br>0.54121233<br>$E_{PW6B95\text{-}D3(BJ)/def2\text{-}TZVPP/CPCM(Chloroform)}$=<br>-2671.561146739696<br>$E_{B3LYP\text{-}D3(BJ)/def2\text{-}TZVPP/CPCM(Chloroform)}$=<br>-2667.566329125127

	C 5.91436020879410 -2.63469387919088 -0.42583630358997<br>C 6.61470470815758 -3.11714469929993 -1.53050968703548<br>H 6.24635524684676 -3.97680117903119 -2.07828493941416<br>C 7.78669454073346 -2.49780897773920 -1.93654640289368<br>H 8.32522705168999 -2.87777062460270 -2.79456448369045<br>C 8.26074180151574 -1.38806954855533 -1.24908937253999<br>H 9.17180995729981 -0.90087624073219 -1.57104454407699<br>C 7.56250864369874 -0.90039945349980 -0.15326459817437<br>H 7.92752606800523 -0.03386408521584 0.38213210396417<br>C 6.39261486259693 -1.52168528105581 0.26079409246574<br>H 5.85653022912606 -1.13351939436504 1.11749988287248<br>C 4.97803646978860 -4.81833511366209 1.21959421323012<br>C 6.26669929433302 -4.84206596047659 1.74486893407018<br>H 6.98521410451842 -4.07893998254723 1.47494777131769<br>C 6.63968060682408 -5.85246654782822 2.62090950965864<br>H 7.64393245045259 -5.86812753223446 3.02340584274679<br>C 5.72956558310710 -6.83677652205657 2.97944957449127<br>H 6.02351769648737 -7.62259752392198 3.66295004662182<br>C 4.44259908613223 -6.81503550008575 2.45725672690855<br>H 3.72844236780587 -7.58060144595067 2.73073695675427<br>C 4.06893362800689 -5.81389243369711 1.57526662503181<br>H 3.06445578565133 -5.80947850703298 1.16919795046506<br>C 3.51157111666033 -2.33238157831427 1.14784772027448<br>C 2.73971550926412 -1.35136452993009 0.52495796580341<br>H 2.68978894608112 -1.29898464652007 -0.55671942202047<br>C 2.02755039145687 -0.43849717453379 1.28596373455497<br>H 1.43027693159372 0.31930535152768 0.79626332039136<br>C 2.07427991056666 -0.50339032026523 2.67369417707325<br>H 1.51081669616119 0.20416436213862 3.26776460015369<br>C 2.83703656501427 -1.48030845684353 3.29567653331428<br>H 2.86878896130221 -1.54041987273717 4.37514241368054<br>C 3.55581831859065 -2.39414054740241 2.53641461121853<br>H 4.13927348032162 -3.15713900579323 3.03485232118608<br>C 0.37597504433125 -4.67863789943634 0.55801954852250<br>C 0.57058678480625 -4.15544466058832 1.82761730922865<br>C 0.72082120441380 -5.01068531070410 2.90614271986048<br>C 0.66872838301897 -6.38480841254618 2.72744933695761<br>C 0.48633270839064 -6.90664703248746 1.45776737534339<br>C 0.34549857415394 -6.05851830787790 0.36025057448248<br>H 0.23492227745736 -4.00180384649526 -0.27660125121248<br>H 0.60281282247519 -3.08298247793395 1.96623619988415<br>H 0.78165570294090 -7.05197708743758 3.57145432978320<br>H 0.45809106562028 -7.97911117511226 1.31284796629728<br>O 0.17797231922425 -6.62146654831329 -0.84849485325210<br>H 0.33237049472232 -5.97722087893609 -1.55363630987959<br>O 5.09138779160011 -10.64234662512155 -3.65912224868902<br>C 5.36244782522478 -11.41865460931016 -4.80578754504937<br>H 6.01044930803604 -12.23033907104625 -4.48403483611221<br>H 4.45252570185033 -11.84602018888175 -5.23607218048847<br>H 5.87928820232133 -10.84268214823939 -5.57840651634596<br>Cl 0.97780866366164 -4.36369607033634 4.49579064210463	
48	Au 2.56548030295261 -4.50013386402508 -1.35844505266580<br>P 4.16699070890143 -3.61462778434037 0.03116497930081<br>O 1.72916105055597 -5.49549759394160 -4.88010492126631<br>C 1.77343895833554 -6.01073439902538 -3.63971009526168<br>C 1.06328545256061 -5.24785205804648 -2.71357623844146<br>C 0.51449562403585 -4.16955548039398 -3.53064917356840<br>C -0.30408617994646 -3.07424450070767 -3.26762019907508<br>H -0.67360815820107 -2.88769271109834 -2.26710042636604<br>C -0.63097947575140 -2.22923736166486 -4.31671602670908<br>H -1.26723953177655 -1.37315826585693 -4.13414503170427<br>C -0.15621406001659 -2.46021420602840 -5.61154481297386<br>H -0.43028138236441 -1.78129491007794 -6.40797246096349<br>C 0.65844612729248 -3.54754406766116 -5.89681248290433<br>H 1.02751472280845 -3.73665973132587 -6.89603115695650<br>C 0.96157137639709 -4.37195035644519 -4.83205533818689<br>C 2.50002058308686 -7.25596633634607 -3.48586120158154<br>C 2.35895418095090 -8.00809652640529 -2.31154751109486<br>H 1.68054794837031 -7.67903150862380 -1.53567341890375<br>C 3.05170874589784 -9.18313584637063 -2.13775717368587<br>H 2.93002061424353 -9.75775067430667 -1.22850647305870<br>C 3.91185012438480 -9.65226000253329 -3.13765604455281<br>C 4.05165784517951 -8.92010078708576 -4.31547698632838<br>H 4.70287226778809 -9.25171804294357 -5.11221641873805<br>C 3.34868965106090 -7.73730523079158 -4.48102370194910	**TS_{syn} (1_{OMe} and 2_{Cl})**<br>$E_{PBEh-3c/CPCM(Chloroform)}$=<br>-2663.132566258964<br>$ZPE_{PBEh-3c/CPCM(Chloroform)}$=<br>0.60462959<br>$FEC_{(298.15)PBEh-3c/CPCM(Chloroform)}$=<br>0.53765009<br>$E_{PW6B95-D3(BJ)/def2-TZVPP/CPCM(Chloroform)}$=<br>-2671.528437183254<br>$E_{B3LYP-D3(BJ)/def2-TZVPP/CPCM(Chloroform)}$=<br>-2667.537359174768

	H 3.48264767033287 -7.18679057411684 -5.40249536744005<br>C 5.47876158192510 -2.69226037589003 -0.81962931097685<br>C 5.98821738326459 -3.20614545296700 -2.01120887081873<br>H 5.57979084859445 -4.11779572077795 -2.43193170520562<br>C 7.01946470527500 -2.55136924841933 -2.66582303387172<br>H 7.41079242327839 -2.95517185684189 -3.59012786854087<br>C 7.54120640932496 -1.37591750228317 -2.14059135941322<br>H 8.34169774656778 -0.86165569732824 -2.65610733676313<br>C 7.03230753117006 -0.85844179161962 -0.95801044828626<br>H 7.43492671824288 0.05819461435766 -0.54770846525650<br>C 6.00403941274746 -1.51430264869522 -0.29521279718916<br>H 5.61559018596552 -1.10296005676060 0.62765377561063<br>C 5.02013541029492 -4.86508021116990 1.02987953879455<br>C 6.35238039166169 -4.71506807794326 1.40818054204521<br>H 6.92638464840564 -3.85931514756039 1.07640721187127<br>C 6.95388779290341 -5.66978883140403 2.21517341143677<br>H 7.98987431288941 -5.55130783944408 2.50394187559466<br>C 6.22970080453878 -6.77147215322391 2.65100729518081<br>H 6.70189878224086 -7.51347271614941 3.28154480759677<br>C 4.90225919479576 -6.92322135666791 2.27412527347963<br>H 4.33373316051107 -7.78121583023040 2.60794252078850<br>C 4.29934873787718 -5.97735470006517 1.45948731249695<br>H 3.26498994532230 -6.11080397973092 1.16694779889405<br>C 3.43759585908423 -2.44883134836089 1.21570328664055<br>C 2.58120403942472 -1.46305017204473 0.72427665663819<br>H 2.37964428001521 -1.39206347379966 -0.33864758187485<br>C 1.97517948161124 -0.57297836658025 1.59470127277197<br>H 1.31152696633996 0.18923914735804 1.20837461813501<br>C 2.21087876266181 -0.66777086074513 2.96189604871236<br>H 1.72821191765524 0.02107807050853 3.64275152185067<br>C 3.05637438857306 -1.65061896759753 3.45243327073469<br>H 3.23437212102640 -1.73422700847433 4.51617224000526<br>C 3.67287832115487 -2.54063662718966 2.58235326340931<br>H 4.31994129168834 -3.31072876715761 2.98030436369985<br>C 0.17101599691876 -4.72565777449600 0.72825891889393<br>C 0.69173629670845 -4.42732847923259 1.98042816945541<br>C 1.09246323128844 -5.45229775408676 2.81890263337902<br>C 0.97027344265579 -6.77650914123334 2.41552822478944<br>C 0.46361427264500 -7.07096701498085 1.16357777567147<br>C 0.02911795175111 -6.05871462159740 0.27610199088675<br>H -0.18890498337186 -3.91555355863384 0.10357776768818<br>H 0.77909678267550 -3.39534966440703 2.29580238889047<br>H 1.28492059287557 -7.57676487730577 3.07361824071821<br>H 0.37360559780607 -8.10644562822878 0.85620697296517<br>O -0.44625940768113 -6.34431760038778 -0.90019968459916<br>H 0.32710728760437 -5.78968226223523 -1.81791772564794<br>O 4.54773946360846 -10.80170788599598 -2.87915871960180<br>C 5.42396891462741 -11.33074001813155 -3.85355833929163<br>H 5.82259726706558 -12.25359316376757 -3.44014647141889<br>H 4.90463533673381 -11.55882764332061 -4.78786140385527<br>H 6.25432941995292 -10.65249206155049 -4.06630563913566<br>Cl 1.77004115902416 -5.08954833234727 4.38140362609712	
49	Au 2.11650209920511 -4.66467109731827 -1.03708489540525<br>P 3.89244767372450 -3.69277466899019 0.05280392111990<br>O 2.54207014273535 -5.11902944236579 -4.29513585673177<br>C 1.94369360052206 -5.91140821293493 -3.38363068131626<br>C 0.82027322000610 -5.29506132896316 -2.83759148937834<br>C 0.72102938776598 -4.02624161326075 -3.53738742754266<br>C -0.16095719039524 -2.94959976033208 -3.49558133706837<br>H -1.02226221309686 -2.96294676707774 -2.84056589838593<br>C 0.10096879154941 -1.86099065573762 -4.31114002082276<br>H -0.57051013847381 -1.01273643512484 -4.29942986675488<br>C 1.21974880460970 -1.83184885540354 -5.15012039794159<br>H 1.39631769206477 -0.96301065784450 -5.76985821860501<br>C 2.10853317499008 -2.89602200397058 -5.20696582222968<br>H 2.97511898621009 -2.88203661753458 -5.85425251030756<br>C 1.81745993600799 -3.97346936622270 -4.39504557662415<br>C 2.49515236458613 -7.23735937419922 -3.19016305657303<br>C 1.79292051757771 -8.18111930688099 -2.42858720298736<br>H 0.82749129698188 -7.93278697199223 -2.00262799375817<br>C 2.31032278806631 -9.43881052026468 -2.22040009321691<br>H 1.75947833147083 -10.16197937671389 -1.63266866000545<br>C 3.54739310411605 -9.79688313201705 -2.76751441824977<br>C 4.25038688648980 -8.86682919740905 -3.53249842636601<br>H 5.20766717948301 -9.10700753322173 -3.97370805689827	**PC_{syn} (1_{OMe} and 2_{Cl})**<br>$E_{PBEh\text{-}3c/CPCM(Chloroform)}$=<br>-2663.138869823377<br>$ZPE_{PBEh\text{-}3c/CPCM(Chloroform)}$=<br>0.60891722<br>$FEC_{(298.15)PBEh\text{-}3c/CPCM(Chloroform)}$=<br>0.54039898<br>$E_{PW6B95\text{-}D3(BJ)/def2\text{-}TZVPP/CPCM(Chloroform)}$=<br>-2671.537812436120

	C 3.72328000745727 -7.60241294244110 -3.73795977978672<br>H 4.29291253340685 -6.89938574737304 -4.33083195387212<br>C 4.92630397248009 -2.70423908432139 -1.07176180009484<br>C 5.18127147848700 -3.19434406390283 -2.35171106777918<br>H 4.74796914803590 -4.13364006815159 -2.67421064561695<br>C 5.98767685750305 -2.48034682621738 -3.22371532293473<br>H 6.17869155675700 -2.86719175038231 -4.21597470359924<br>C 6.53948199977875 -1.26871821687868 -2.82746252891104<br>H 7.16258567152009 -0.70772110161289 -3.51156299995760<br>C 6.28612818104485 -0.77569884986277 -1.55563972334378<br>H 6.71215893428603 0.16857878045528 -1.24329415968967<br>C 5.48287990848475 -1.49010380834343 -0.67724394584310<br>H 5.29159441679507 -1.09337655427798 0.31159385215913<br>C 5.02797552447043 -4.86320872116923 0.84511351444332<br>C 6.36529468193207 -4.53350795300038 1.06167281718197<br>H 6.75809782639071 -3.58034784791574 0.73078395047617<br>C 7.20499976244213 -5.43142840734824 1.70233864902743<br>H 8.24235966485149 -5.17166083292405 1.86679989735961<br>C 6.71654035112406 -6.65982561322277 2.12926946226182<br>H 7.37478023083691 -7.35928339313741 2.62783968520114<br>C 5.38717738425830 -6.99205940022483 1.91126822082315<br>H 5.00304136739360 -7.94955920365017 2.23713324324230<br>C 4.54406599391207 -6.09874146997946 1.26681631817155<br>H 3.50976626804458 -6.37127245121967 1.09587858869606<br>C 3.36186344680023 -2.53132668438947 1.34096766447256<br>C 2.41793165784445 -1.56317504354356 0.99443748148168<br>H 2.02980613275375 -1.51419244513163 -0.01700226362848<br>C 1.96806358793228 -0.66119763596479 1.94296609561183<br>H 1.23769894524963 0.08839738114024 1.66850913856689<br>C 2.44760336394722 -0.72669214580120 3.24687424284082<br>H 2.08963299660939 -0.02667188722164 3.99039407831452<br>C 3.37731502667330 -1.69352337210451 3.59492054554368<br>H 3.74601465093523 -1.75315558606884 4.61031173990980<br>C 3.83745513381086 -2.59659171714002 2.64451032206939<br>H 4.55714298682712 -3.35130099047250 2.93142465919981<br>C -0.07022191029655 -5.05667176048080 0.53585224696585<br>C 0.59325276800816 -4.72773995961232 1.72295412290397<br>C 1.18232079236185 -5.71569204818649 2.48355534803055<br>C 1.11608656634424 -7.04698463601150 2.06784702355499<br>C 0.44651126312207 -7.38662606243879 0.91509639225917<br>C -0.24958415786412 -6.42021171269270 0.11349749829977<br>H -0.63536903984626 -4.28149639558691 0.02542505897004<br>H 0.63872158952935 -3.69275358990541 2.03985493536971<br>H 1.59431625984296 -7.81885606518711 2.65921706646115<br>H 0.38560190326744 -8.43020379945082 0.62739281957269<br>O -0.94520453172303 -6.74413925485996 -0.89051693290523<br>H 0.00539849374258 -5.81851546647257 -2.32126487366681<br>O 3.97175129673980 -11.04010592077362 -2.50829601534008<br>C 5.21356861262487 -11.46127403324676 -3.03505691106098<br>H 5.35582788107548 -12.48700130403756 -2.70471519335690<br>H 5.22143466616367 -11.43912014660135 -4.12787903131559<br>H 6.04199529979174 -10.85330327760934 -2.66241576431865<br>Cl 2.02919536636242 -5.31745165784630 3.95422237252735	
50	Au 1.83975365077572 -4.40977308716215 0.20349582611883<br>P 3.88432026583125 -3.45073920868523 0.65847969570783<br>C 4.68906516974607 -2.74888338517052 -0.80933509689896<br>C 4.63013475491981 -3.46365185103956 -2.00525874712685<br>H 4.08930236254717 -4.40153329965778 -2.05812698471275<br>C 5.26417943862757 -2.97651276092388 -3.13713859437982<br>H 5.21486018039733 -3.53569421423074 -4.06192814296757<br>C 5.95138776849529 -1.77043003915957 -3.08494387731715<br>H 6.44021173467768 -1.38779130081617 -3.97128342753999<br>C 6.00572515812025 -1.05353074220548 -1.89832020881288<br>H 6.53711444965199 -0.11204834301013 -1.85540460571421<br>C 5.37800182707898 -1.54009706757309 -0.75994855831577<br>H 5.42773173734699 -0.97190866950262 0.15994572508603<br>C 5.07420006516738 -4.63457130720987 1.34988486771765<br>C 6.41882968336912 -4.61491232520470 0.98941395748084<br>H 6.78341909697819 -3.90094324598614 0.26221669731099<br>C 7.30295353720426 -5.51895272989331 1.56165600013259<br>H 8.34622586548605 -5.50150543853687 1.27529693243292<br>C 6.85101920027950 -6.44101893286883 2.49479294109064<br>H 7.54229183600303 -7.14602002297496 2.93761250863367<br>C 5.50983915927277 -6.46371792901505 2.85547175757586<br>H 5.15237036823947 -7.18501981451521 3.57811208634408	**4-ClBrOAuPPh$_3$**<br>$E_{PBEh-3c/CPCM(Chloroform)}$=<br>-1935.453884634502<br>$ZPE_{PBEh-3c/CPCM(Chloroform)}$=<br>0.37008869<br>$FEC_{(298.15)PBEh-3c/CPCM(Chloroform)}$=<br>0.31604921<br>$E_{PW6B95-D3(BJ)/def2-TZVPP/CPCM(Chloroform)}$=<br>-1941.030265872598

	C 4.62168555535500 -5.56760844128793 2.28210314652584<br>H 3.57558564336921 -5.59984195568121 2.56386057080352<br>C 3.79039648380918 -2.09122207748330 1.85767752761939<br>C 2.75506067317984 -1.16568390478518 1.73069038643611<br>H 2.01289193982816 -1.27784782016568 0.94860842460570<br>C 2.67014744917025 -0.09365192576444 2.60455081966301<br>H 1.86533590768762 0.62173695362137 2.50000439449322<br>C 3.61100492610373 0.05544380791893 3.61574030561007<br>H 3.54004389427012 0.88930780838403 4.30172486353437<br>C 4.63745386601230 -0.86807771220403 3.74960077312107<br>H 5.36959184185200 -0.75675147756164 4.53835739014856<br>C 4.73097469216893 -1.94003321305624 2.87241650606924<br>H 5.53718934330143 -2.65304582103777 2.98642990823774<br>C -0.10528561501556 -5.31816916259133 -0.21574264473705<br>C -0.51777986798823 -5.54481404620786 1.14763840634517<br>C -0.23537479600491 -6.71810634832776 1.76893702432912<br>C 0.43881817901668 -7.75212257670790 1.06766495826524<br>C 0.76105860695612 -7.61877332343879 -0.24526941280908<br>C 0.37649086415827 -6.44670330795063 -1.02547735325812<br>H -0.66920708976876 -4.55789335116847 -0.75878463428838<br>H -1.04620871273266 -4.75609945627433 1.66937439619873<br>H 0.68850822376011 -8.66667460421549 1.59182430548123<br>H 1.23463571583411 -8.44119597618929 -0.76784654734024<br>O 0.47508198751377 -6.41214792540920 -2.25398835145473<br>Cl -0.65509127105325 -6.97416809707316 3.43757606455413	
51	C -0.31228386387804 -4.33695698532799 -1.54795067364231<br>C -0.33362088576529 -5.48506456565802 -0.76805739304914<br>C 0.82347148909414 -5.88957182441567 -0.13563322547416<br>C 2.00016027560892 -5.17608337397198 -0.26187677556460<br>C 2.01766596701509 -4.03095136536352 -1.04163394513261<br>C 0.86184494432984 -3.60303947786363 -1.69135845836220<br>H -1.22176848384712 -4.01936072577247 -2.04512058559444<br>H -1.24747211079849 -6.05427411728904 -0.65920875640092<br>H 2.89862827286479 -5.50804052193953 0.24177190112627<br>H 2.93387496010391 -3.46459338902686 -1.14853730637199<br>O 0.94113115696883 -2.48160025382976 -2.43827062891064<br>H 0.09254977763523 -2.28844458541914 -2.85052518638176<br>F 0.80691717966820 -7.00207308312235 0.62028161975850	**4-fluorophenol**<br>$E_{PBEh-3c/CPCM(Chloroform)}$=<br>-405.856375856060<br>$ZPE_{PBEh-3c/CPCM(Chloroform)}$=<br>0.09962267<br>$FEC_{(298.15)PBEh-3c/CPCM(Chloroform)}$=<br>0.06947027<br>$E_{PW6B95-D3(BJ)/def2-TZVPP/CPCM(Chloroform)}$=<br>-407.378681140866<br>$E_{B3LYP-D3(BJ)/def2-TZVPP/CPCM(Chloroform)}$=<br>-406.689863975313
52	Au 0.42354351748441 -3.99966097959115 -2.24053332829668<br>P 1.07388748708313 -3.65375645754524 -0.03059867895401<br>O -0.75634874228864 -3.73657646716030 -6.33212261238731<br>C -0.14804932976021 -3.28751434539749 -5.18621692792845<br>C -0.16495564579265 -4.21536764341702 -4.18354583868855<br>C -0.84549165923258 -5.34784360138415 -4.77220571648414<br>C -1.21248241536562 -6.61398623134754 -4.31155196712058<br>H -0.98212157397680 -6.91940658892716 -3.29752685632501<br>C -1.87875365984185 -7.46871905558141 -5.17365233049546<br>H -2.16882307804988 -8.45412639181474 -4.83276894368095<br>C -2.18894714389408 -7.08050806715069 -6.48235428263747<br>H -2.71025384749339 -7.77055401439456 -7.13266168875763<br>C -1.84363179758176 -5.82530198149980 -6.96176437925673<br>H -2.08446983855370 -5.51773891084514 -7.97100906790130<br>C -1.17906187254330 -4.99066151803232 -6.08122085247465<br>C 0.33954657524183 -1.91375429130421 -5.24972721229639<br>C 1.46327596857018 -1.50861838817607 -4.53451223330068<br>H 2.03826222256667 -2.23278351092605 -3.97335373438303<br>C 1.89921075661698 -0.19197386845705 -4.54333265343943<br>H 2.78082048441805 0.06407371034275 -3.97149113244158<br>C 1.21475193711500 0.76066558828078 -5.29358127884087<br>C 0.10404730254391 0.36347251851391 -6.04116665762771<br>H -0.42822916633014 1.09762231815046 -6.63279095767225<br>C -0.32168865540701 -0.94893726051209 -6.01960177631663<br>H -1.19608578642788 -1.21873331454068 -6.59713991316267<br>C 1.39862544051375 -1.89346505363975 0.29713789607325<br>C 2.12563121071974 -1.17126704930936 -0.64915468664737<br>H 2.47747614335436 -1.65405105290253 -1.55310821256343<br>C 2.40391617892611 0.16993938427965 -0.44042408792018<br>H 2.96573601595218 0.72306119417529 -1.18195011503692<br>C 1.95318617326983 0.80245508533806 0.71138849775445<br>H 2.16407783338093 1.85174203332281 0.87114106070909	**RC*anti* (1OMe and 2F)**<br>$E_{PBEh-3c/CPCM(Chloroform)}$=<br>-2303.111374067888<br>$ZPE_{PBEh-3c/CPCM(Chloroform)}$=<br>0.61165072<br>$FEC_{(298.15)PBEh-3c/CPCM(Chloroform)}$=<br>0.54373349<br>$E_{PW6B95-D3(BJ)/def2-TZVPP/CPCM(Chloroform)}$=<br>-2310.972509771272<br>$E_{B3LYP-D3(BJ)/def2-TZVPP/CPCM(Chloroform)}$=<br>-2307.244469289205

	C 1.22751438039899 0.08864924026026 1.65398648174181<br>H 0.87281587586324 0.57798066358643 2.55148674204503<br>C 0.95013240277590 -1.25664292230913 1.45041324586022<br>H 0.38157928202267 -1.80094974032719 2.19331548788885<br>C 2.59294442677582 -4.51736433349628 0.46665492170189<br>C 3.54852996099544 -3.91430262282553 1.28043031721806<br>H 3.42203440331841 -2.89137827617022 1.61106231823716<br>C 4.67365008255894 -4.62529007629842 1.67377960647681<br>H 5.41449488039934 -4.14995874075929 2.30310293222309<br>C 4.84526154973716 -5.94007694069734 1.26457375941294<br>H 5.72199685413235 -6.49362806185741 1.57435967332148<br>C 3.89484606373646 -6.54424322521017 0.45207800014306<br>H 4.02882431673391 -7.56700775837551 0.12780020731039<br>C 2.77608917159054 -5.83376343788884 0.04534379237434<br>H 2.04780952629550 -6.30959699654149 -0.60092123188962<br>C -0.17204936954090 -4.14345297970687 1.19926628591377<br>C -1.51319456438338 -3.88083270085249 0.92158707071199<br>H -1.79711404767117 -3.41911741475408 -0.01700890386715<br>C -2.49311836387006 -4.20858328150641 1.84547993754627<br>H -3.53138454446213 -4.00059710238890 1.62338346533107<br>C -2.14242488338493 -4.81044772752461 3.04718176384467<br>H -2.90860386666214 -5.07264079340356 3.76500122092108<br>C -0.80991609885156 -5.08094829620212 3.32372707714223<br>H -0.53330498366974 -5.55332385939506 4.25706444071262<br>C 0.17535455027098 -4.74736555072401 2.40457870234337<br>H 1.21116639167799 -4.96305471013049 2.63262337068598<br>C 2.52014096360948 -6.96331405240034 -3.70006736245774<br>C 2.91836061725029 -8.12957307301554 -3.06163788167657<br>C 4.23490715768481 -8.26684725837479 -2.67453869527762<br>C 5.16316520743004 -7.27045126310841 -2.90585956674274<br>C 4.76023613130567 -6.10502239678742 -3.53771256370192<br>C 3.43611902765330 -5.94200673731846 -3.93933258680860<br>H 1.49037764280593 -6.85819798092695 -4.01976573877946<br>H 2.20441537518473 -8.92164025491583 -2.87648107604028<br>H 6.19104158467103 -7.39551057043970 -2.59129693316830<br>H 5.47658357957649 -5.31422414232642 -3.71979306234372<br>O 3.10220514227390 -4.79071089220664 -4.55595960026867<br>H 2.14230347360864 -4.72928124400975 -4.66784190542333<br>F 4.62275302459165 -9.39258766409564 -2.04365780660361<br>O 1.55008131748980 2.06100643446510 -5.36233355633980<br>C 2.65266492850823 2.51299033724788 -4.60690384314533<br>H 2.51011486790228 2.34748545797972 -3.53492547831629<br>H 3.58580310554425 2.03346195688018 -4.91513376249874<br>H 2.73424589466854 3.58214756534258 -4.78695493064495	
53	Au 0.66844079775611 -4.31610096126188 -1.76313254851514<br>P 0.84200540842641 -3.37767564970609 0.31988889518042<br>O -1.04174058095058 -3.91096386148621 -4.95134486365087<br>C 0.19025248712844 -3.84827107717078 -4.42684646679725<br>C 0.57324361832741 -5.04151481548576 -3.81071008878592<br>C -0.57040463459512 -5.91607294636752 -4.04381022343889<br>C -0.85656502616323 -7.24363086010857 -3.73707860284313<br>H -0.13945449036810 -7.85577307055278 -3.20531902109027<br>C -2.08253394340704 -7.75879453222895 -4.12863841268550<br>H -2.32478931955508 -8.78886721219112 -3.90260964192630<br>C -3.01668157709440 -6.97472691470406 -4.81251388340390<br>H -3.96426793638636 -7.40822001200384 -5.10307576709282<br>C -2.75053133169347 -5.65000666792777 -5.13116405140890<br>H -3.46434739764431 -5.03689958103059 -5.66483442949143<br>C -1.51979576850998 -5.16910026060436 -4.73332994911904<br>C 0.90175264696366 -2.59221922646266 -4.57854318755199<br>C 2.28081833118170 -2.54641363397150 -4.37229320352160<br>H 2.82624582067183 -3.45648734930419 -4.15262176741853<br>C 2.97506098020598 -1.35216194549085 -4.44502812983877<br>H 4.04368546138023 -1.36390308131226 -4.28126736985580<br>C 2.29255716914461 -0.16727212925315 -4.72660828397512<br>C 0.91395808071226 -0.20819085149623 -4.95441294283248<br>H 0.38533331929261 0.70933942917133 -5.17868443425277<br>C 0.23121409981290 -1.40314286214381 -4.88579861390106<br>H -0.83752450619775 -1.40096479220430 -5.05321360703027<br>C 0.75944638320211 -1.56574384781653 0.20455936662403<br>C 1.40036720696821 -0.93227079688101 -0.86047154554989<br>H 1.92130661419528 -1.50947473762002 -1.61590541049795<br>C 1.37304723210097 0.44922636119783 -0.96558005685055<br>H 1.86807608849776 0.93279652534412 -1.79756638790077<br>C 0.70174497748138 1.20620293231283 -0.01378102392695	**TS*anti* (1$_{OMe}$ and 2$_{F}$)**<br>$E_{PBEh-3c/CPCM(Chloroform)}$=<br>-2303.068595699782<br>$ZPE_{PBEh-3c/CPCM(Chloroform)}$=<br>0.60627832<br>$FEC_{(298.15)PBEh-3c/CPCM(Chloroform)}$=<br>0.53859691<br>$E_{PW6B95-D3(BJ)/def2-TZVPP/CPCM(Chloroform)}$=<br>-2310.931062155416<br>$E_{B3LYP-D3(BJ)/def2-TZVPP/CPCM(Chloroform)}$=<br>-2307.206228469660

	H 0.67464195076049 2.28446091624481 -0.10090365417965<br>C 0.05977514463137 0.57916181375810 1.04442649135437<br>H -0.46691683304523 1.16551897296815 1.78562684675674<br>C 0.08704437319399 -0.80421512689101 1.15653113712321<br>H -0.42011355993548 -1.28161377551715 1.98514612282144<br>C 2.40827500477367 -3.75078990130915 1.15448845849879<br>C 3.08427958893688 -2.78811393799987 1.89967027850353<br>H 2.69882635657550 -1.77946469886198 1.97510477699991<br>C 4.26504994065926 -3.11928074968150 2.54916238586618<br>H 4.78880996905996 -2.36724160972296 3.12422341114685<br>C 4.77241245251397 -4.40784123200725 2.45962727054796<br>H 5.69490330169023 -4.66237945433452 2.96477534063258<br>C 4.10088236368030 -5.36929912468389 1.71559161801699<br>H 4.49749430060871 -6.37271896210480 1.63723532774208<br>C 2.92423975499890 -5.04246109686669 1.06014549548906<br>H 2.41314292092001 -5.79601061299490 0.47240778615245<br>C -0.46862945874319 -3.85791192697371 1.47839316517423<br>C -1.77921953933946 -3.92367914295551 1.00646119901122<br>H -1.99749429302522 -3.71777188780699 -0.03503977739007<br>C -2.81179593257581 -4.25469229507337 1.86887033259859<br>H -3.82669792353764 -4.30343426951511 1.49734214324442<br>C -2.54164494683885 -4.53211289608386 3.20321554839857<br>H -3.34798423143826 -4.79739755072466 3.87430927777677<br>C -1.23782612207688 -4.47531723615810 3.67346897650027<br>H -1.02362620098244 -4.69480664065600 4.71100315074177<br>C -0.20032106229320 -4.13708205475704 2.81521148927028<br>H 0.81266074165114 -4.09559501776309 3.19400108961542<br>C 2.73574120309057 -7.26222392195498 -5.68825320781213<br>C 3.26979963727005 -7.94460628307680 -6.77159415239863<br>C 4.63272519452138 -7.91504775881129 -6.98915202436119<br>C 5.47095944206215 -7.21382403683041 -6.14411979795299<br>C 4.93302189933762 -6.52690518702015 -5.06686524257518<br>C 3.54636808360391 -6.52674962933315 -4.79291158316521<br>H 1.66509176739788 -7.30224615738649 -5.52279595615197<br>H 2.62311721820055 -8.49982002059221 -7.44029777706142<br>H 6.53897924862878 -7.19868124708682 -6.32530877547824<br>H 5.59262359619217 -5.97648173453641 -4.40625756295926<br>O 3.06059874939371 -5.87997241880415 -3.77316907261406<br>H 1.73928670657499 -5.46070267613676 -3.81469180061558<br>F 5.15619455666777 -8.58421571409584 -8.04468394320259<br>O 2.86859753793522 1.03978862515756 -4.79293518320162<br>C 4.26138015228954 1.14409735717893 -4.57138945507198<br>H 4.53995364664149 0.80932354387803 -3.56886073260317<br>H 4.83478548690718 0.57505589911886 -5.30731057870968<br>H 4.51016734557771 2.19740566956514 -4.67137367012947	
54	Au 2.08841781394225 -5.21853805112772 -1.98654827326261<br>P 1.29708369904921 -4.27771239635573 -0.10179138444321<br>O -1.24028802671910 -2.47443010079198 -3.35289426028266<br>C -0.37546573350752 -3.18704873439654 -4.12571351865225<br>C -0.80075390348874 -4.46932857041764 -4.27275744359176<br>C -2.02575116002831 -4.57417032247053 -3.54043672227079<br>C -2.95419591444377 -5.58644867501670 -3.28952388057591<br>H -2.82227773623098 -6.57719648557187 -3.70489775057024<br>C -4.04694923534972 -5.29065971870900 -2.49297179515281<br>H -4.77996421620738 -6.05962766927373 -2.28610689289461<br>C -4.22487018487570 -4.01459519768976 -1.94457854950041<br>H -5.08786808467842 -3.81964566727144 -1.32193202617597<br>C -3.31738221719634 -2.99366961154369 -2.18492313264256<br>H -3.44783708799301 -2.00495321754941 -1.76555921283255<br>C -2.23704337492422 -3.30773150058362 -2.98797987468678<br>C 0.79186280657379 -2.48705835982654 -4.62702582646140<br>C 1.81392192864879 -3.19774759874829 -5.25212953185287<br>H 1.73390964281611 -4.26929188704287 -5.37232405912518<br>C 2.95671812628953 -2.57113762613036 -5.71753661718065<br>H 3.72632215904096 -3.17765777036537 -6.17565825329219<br>C 3.09703443126984 -1.19201999719792 -5.57101790391773<br>C 2.07206349775354 -0.46479645189590 -4.96101788877913<br>H 2.17395623444872 0.60776374367911 -4.85282504218749<br>C 0.94026538417751 -1.10265802206340 -4.49578632292681<br>H 0.16731728596328 -0.51043733599043 -4.02576891287016<br>C 0.66428423888403 -2.59126892155240 -0.32416581528397<br>C 1.44745778812740 -1.68185125940191 -1.03333702511240<br>H 2.37996519744619 -1.99673390802663 -1.48745933827737<br>C 1.03648479158012 -0.36540884842608 -1.16224247664002<br>H 1.65180610223073 0.33624419018673 -1.70933341927407	**PC$_{anti}$ (1$_{OMe}$ and 2$_{F}$)**<br>$E_{PBEh\text{-}3c/CPCM(Chloroform)}$=<br>-2303.122037491857<br>$ZPE_{PBEh\text{-}3c/CPCM(Chloroform)}$=<br>0.61135072<br>$FEC_{(298.15)PBEh\text{-}3c/CPCM(Chloroform)}$=<br>0.54409713<br>$E_{PW6B95\text{-}D3(BJ)/def2\text{-}TZVPP/CPCM(Chloroform)}$=<br>-2310.983851635816

	C -0.16730871691079 0.04551298975016 -0.60413087897762<br>H -0.49086994082058 1.07253771980561 -0.71242210520024<br>C -0.95722750480225 -0.86203244820637 0.08630261288680<br>H -1.89833402368411 -0.54680042726986 0.51738860701950<br>C -0.54185430937514 -2.17842561871660 0.23331024749289<br>H -1.16314737250075 -2.87504249912820 0.78064492966031<br>C 2.57637121385355 -4.12689933539809 1.18126214826448<br>C 2.64401825220628 -3.01302163889365 2.01407338532063<br>H 1.94961433783764 -2.19193535976445 1.89202330403200<br>C 3.60942311880530 -2.94744028892126 3.00908219762262<br>H 3.65875686029816 -2.07720334181379 3.65011008910753<br>C 4.50638840981964 -3.99196141697251 3.18047869230960<br>H 5.25941815007109 -3.93746022780324 3.95571111269739<br>C 4.44053401334507 -5.10551506201002 2.35306994567981<br>H 5.14029873770079 -5.92071580023016 2.48071405076963<br>C 3.48221370265454 -5.17262521034289 1.35389905712398<br>H 3.44417292098204 -6.04320473088202 0.70976688540463<br>C -0.05127395227498 -5.20953188372320 0.67925522858054<br>C -1.07319009735728 -5.70714273746628 -0.12773167500039<br>H -1.04823353465032 -5.56030228482137 -1.20097967829321<br>C -2.13651564684425 -6.39052677394867 0.44052941554924<br>H -2.92656307626344 -6.76900134242591 -0.19376334049972<br>C -2.18195100195732 -6.58961893195541 1.81426215889369<br>H -3.00948654067204 -7.12920836040985 2.25600124031629<br>C -1.16383239342132 -6.09980946803906 2.61961969668149<br>H -1.19418383649394 -6.25500101702812 3.68998951962578<br>C -0.10043559400492 -5.40808105830229 2.05652732387232<br>H 0.68599647104228 -5.03028898493910 2.69666177726266<br>C 3.97621228687487 -6.14507926429630 -5.67297579404570<br>C 5.06869750459104 -5.78121383551483 -6.44533642073806<br>C 6.07825357916240 -5.03034810510836 -5.87559027722769<br>C 6.01671589145603 -4.64543437912949 -4.55176439887498<br>C 4.92003786186729 -5.01117424648536 -3.78474756998534<br>C 3.86387647062241 -5.76486015761116 -4.32366733251111<br>H 3.18165166793894 -6.73031556235184 -6.11985839107462<br>H 5.12845872792876 -6.08085612499153 -7.48424959453297<br>H 6.81678910212619 -4.06063251321621 -4.11514832354432<br>H 4.88767416087102 -4.69855282765006 -2.74673328931324<br>O 2.79480819495542 -6.13538816707620 -3.64538573717068<br>H -0.31309683081781 -5.24548456451989 -4.84012707675998<br>F 7.14096677928275 -4.66496439184303 -6.62609844195888<br>O 4.16743421603402 -0.49080527384472 -5.97892009903847<br>C 5.23907103106478 -1.18594705513183 -6.58276471728299<br>H 5.67769986321066 -1.92629554663563 -5.90852583378834<br>H 4.93369579825369 -1.68918804885753 -7.50403377976020<br>H 5.99293680889471 -0.44079802380522 -6.82466034749629	
55	Au 3.06823739067543 -4.25499832150602 -1.65673006655803<br>P 4.40950822373774 -3.49584478912555 0.08942930745238<br>O 0.91579641142536 -6.06214560811079 -4.87868039522549<br>C 1.85850991276418 -6.05307366645775 -3.87897290767522<br>C 1.83569640837553 -4.89915689948788 -3.14901099968131<br>C 0.76444179581203 -4.13195767881945 -3.74576117153786<br>C 0.20132334546337 -2.88010887179422 -3.49045635068179<br>H 0.57740877169431 -2.26086966769136 -2.68449360045890<br>C -0.84521173450878 -2.44644446639096 -4.28687890285914<br>H -1.29419161865071 -1.47911143873609 -4.10213096626464<br>C -1.33636599494210 -3.23784743689173 -5.33214732077531<br>H -2.15518583804050 -2.87146822456247 -5.93728413729682<br>C -0.79371877546260 -4.48466635115637 -5.60748093139159<br>H -1.16952825067377 -5.10122300252761 -6.41359075740119<br>C 0.24965530610995 -4.89498031620241 -4.79705068678061<br>C 2.68721344064967 -7.25114264982897 -3.81529637752521<br>C 3.25831991060973 -7.67892030321788 -2.61109771755409<br>H 3.05827754705040 -7.14281983754606 -1.69314450677892<br>C 4.06025854961709 -8.79917778123804 -2.55532724804487<br>H 4.48771788309292 -9.11375917254648 -1.61152266951837<br>C 4.31046761000811 -9.54914331042981 -3.70679365386870<br>C 3.73411630818555 -9.15048323919027 -4.90975401383499<br>H 3.89766960708848 -9.70274428698066 -5.82503777276134<br>C 2.93356718555577 -8.01760078910700 -4.95327186917799<br>H 2.51034465088242 -7.72875018691117 -5.90622409137593<br>C 5.90764726496089 -2.59800261227587 -0.40882803364979<br>C 6.59373823708711 -3.02782284785789 -1.54357306836694<br>H 6.21752344292281 -3.86014311054050 -2.12691587348406<br>C 7.76162249560743 -2.39073539349227 -1.93403622049555	**RC*syn* (1_{OMe} and 2_F)**<br>$E_{PBEh\text{-}3c/CPCM(Chloroform)}$=<br>-2303.113456582791<br>$ZPE_{PBEh\text{-}3c/CPCM(Chloroform)}$=<br>0.61125220<br>$FEC_{(298.15)PBEh\text{-}3c/CPCM(Chloroform)}$=<br>0.54344946<br>$E_{PW6B95\text{-}D3(BJ)/def2\text{-}TZVPP/CPCM(Chloroform)}$=<br>-2310.975627103217<br>$E_{B3LYP\text{-}D3(BJ)/def2\text{-}TZVPP/CPCM(Chloroform)}$=<br>-2307.247902532031

	H 8.28910408718420 -2.72983366114008 -2.81570931401104<br>C 8.24560277056730 -1.31551761606740 -1.20040406301050<br>H 9.15337712690415 -0.81429669618874 -1.50991317582444<br>C 7.56153823572854 -0.87998441602909 -0.07397310782777<br>H 7.93433219780528 -0.04025789936923 0.49756073920237<br>C 6.39597578725928 -1.51924953264881 0.32420047365489<br>H 5.87074505260852 -1.17177289490567 1.20477238429812<br>C 4.98644043760371 -4.84284792768369 1.16379871306430<br>C 6.28693665237561 -4.90217298731117 1.65523579080919<br>H 7.00897793907934 -4.13900781332889 1.39510296914669<br>C 6.66764975083262 -5.94918895758590 2.48401646462772<br>H 7.68133868050680 -5.99250181926954 2.86000209932860<br>C 5.75364640994838 -6.93457073318917 2.82892598755439<br>H 6.05399038277198 -7.74922292082756 3.47486621164358<br>C 4.45413082739396 -6.87679634055219 2.34109596385536<br>H 3.73629953158141 -7.64253108758996 2.60445465436397<br>C 4.07250007472766 -5.83897892552366 1.50635455198756<br>H 3.05791631819957 -5.80711423237215 1.12684229873632<br>C 3.51563078123686 -2.35769116062480 1.18989710451907<br>C 2.75194808032087 -1.34693595107488 0.60655689424412<br>H 2.70567510257933 -1.24958379988276 -0.47220556009128<br>C 2.04290911803733 -0.46223424815111 1.40324502638397<br>H 1.45283003708061 0.31999280907322 0.94418975265380<br>C 2.08294537286016 -0.58703709463244 2.78700167845063<br>H 1.52107811695022 0.09799184789013 3.40835253847232<br>C 2.83662712217474 -1.59496093222984 3.36977114664276<br>H 2.86687878105929 -1.69828790793772 4.44622824235242<br>C 3.55515994814938 -2.47806273030882 2.57484026279446<br>H 4.13584711752077 -3.26268128988940 3.04207924442112<br>C 0.33864497901899 -4.64450065046480 0.50916628494842<br>C 0.51064647902856 -4.08484641356703 1.76739511159860<br>C 0.66128319153418 -4.91602600292999 2.85810606777740<br>C 0.64042169904409 -6.29095418268784 2.72540256198575<br>C 0.48129563837866 -6.84663085599763 1.46476366837369<br>C 0.33452561942860 -6.02904820070688 0.34570419123479<br>H 0.19379081330194 -3.99265324958559 -0.34440289873825<br>H 0.52432591290646 -3.00957631047328 1.88972683207491<br>H 0.75669249079436 -6.92715241284658 3.59320272973234<br>H 0.47505156721528 -7.92254417595996 1.34520868982136<br>O 0.18297850753082 -6.62385249076623 -0.85402340305452<br>H 0.36179559268960 -5.99887140755269 -1.57052225704035<br>O 5.10147117161610 -10.62650158946254 -3.55823793764168<br>C 5.37300843423536 -11.42540937690959 -4.68919687069453<br>H 6.01778150563878 -12.23269255253347 -4.35033263000926<br>H 4.46284439865207 -11.85815572524948 -5.11356088166358<br>H 5.89354353350176 -10.86588337687368 -5.47138682322216<br>F 0.83067602285367 -4.37695872112998 4.07993398983908	
56	Au 2.51017065887250 -4.49355037661779 -1.34453936656049<br>P 4.14087571437007 -3.63477499239463 0.02701610011115<br>O 1.78046636965426 -5.48366688117615 -4.85286801791088<br>C 1.77238920105050 -6.00356681054925 -3.61305364245933<br>C 1.01801858160632 -5.24792236706373 -2.71658470345836<br>C 0.49804502943631 -4.17078369815220 -3.55327583179924<br>C -0.33867615522752 -3.08178956689661 -3.32298752797851<br>H -0.75355669935921 -2.90156631132375 -2.33927843700572<br>C -0.62431303998130 -2.23504444097602 -4.38258502138227<br>H -1.27393563251432 -1.38395063818445 -4.22555562459252<br>C -0.09061518244535 -2.45779669999804 -5.65575722586392<br>H -0.33359270848688 -1.77762256504779 -6.46117057678477<br>C 0.74328271321759 -3.53853686840220 -5.90838050598829<br>H 1.15770131158716 -3.72123641003678 -6.89088057955133<br>C 1.00423572979860 -4.36545193234745 -4.83436846427047<br>C 2.49797454483533 -7.24563076128947 -3.42917320667118<br>C 2.28345148302458 -8.01495496287360 -2.27744022326345<br>H 1.54584531302762 -7.70376222648894 -1.54929292774594<br>C 2.97608212743219 -9.18508934058143 -2.07129143515759<br>H 2.79739084852953 -9.77406499435738 -1.18079236400761<br>C 3.90906941606539 -9.63106797087352 -3.01468034145741<br>C 4.12145529450824 -8.88182804842168 -4.17075657392873<br>H 4.82994293397338 -9.19587917261467 -4.92459128322095<br>C 3.41812685618704 -7.70427959012928 -4.36969986182048<br>H 3.60941279494409 -7.13912306304448 -5.27207986427168<br>C 5.42388652747821 -2.68604455662977 -0.83986508207149<br>C 5.89730310376684 -3.16708638923366 -2.05973057364790<br>H 5.47772628908776 -4.06832787417838 -2.49177334377012	**TS$_{syn}$ (1$_{OMe}$ and 2$_{F}$)**<br>E$_{PBEh\text{-}3c/CPCM(Chloroform)}$=<br>-2303.076607424158<br>ZPE$_{PBEh\text{-}3c/CPCM(Chloroform)}$=<br>0.60609987<br>FEC$_{(298.15)PBEh\text{-}3c/CPCM(Chloroform)}$=<br>0.53939043<br>E$_{PW6B95\text{-}D3(BJ)/def2\text{-}TZVPP/CPCM(Chloroform)}$=<br>-2310.941086962705<br>E$_{B3LYP\text{-}D3(BJ)/def2\text{-}TZVPP/CPCM(Chloroform)}$=<br>-2307.217318835723

	C 6.90715516758593 -2.49320546071705 -2.72810510001496<br>H 7.27030657559544 -2.87177711001088 -3.67433026254629<br>C 7.44345419951148 -1.33098298210256 -2.18833576692921<br>H 8.22708055620615 -0.80166921902941 -2.71441886293336<br>C 6.97048884821259 -0.84606643900607 -0.97742745337374<br>H 7.38444631713770 0.06014344564901 -0.55540809806059<br>C 5.96391863513278 -1.52134918224698 -0.30091626940287<br>H 5.60398177031094 -1.13511532053366 0.64406610353250<br>C 5.03122085433035 -4.91387854794759 0.95568234937231<br>C 6.36998994008116 -4.75878380196766 1.30871169861586<br>H 6.92085471939554 -3.87729447727614 1.00636019958223<br>C 7.00893335045646 -5.74169706220323 2.05019784619056<br>H 8.04964213797297 -5.61815899177133 2.31915768575232<br>C 6.31649402461185 -6.87884096539138 2.44452267527014<br>H 6.81831642948487 -7.64435911512214 3.02181539573168<br>C 4.98274908426749 -7.03603234684410 2.09328493379808<br>H 4.43912296079428 -7.92164543611535 2.39479626430215<br>C 4.34114044030520 -6.05973478745357 1.34639391712108<br>H 3.30135099899361 -6.19754813349839 1.07478055202799<br>C 3.45189282003706 -2.50173807297428 1.26538693141829<br>C 2.61226330311860 -1.47994629376073 0.82185528876937<br>H 2.40116559644242 -1.36451358150744 -0.23529708142129<br>C 2.03536827613684 -0.61105096964646 1.73266422915013<br>H 1.38513865913053 0.18011562199839 1.38349644024634<br>C 2.28256445786513 -0.76430493708798 3.09239710037031<br>H 1.82278234343228 -0.09177070481616 3.80466011293989<br>C 3.11149800399671 -1.78349234496744 3.53530046790173<br>H 3.30081705845330 -1.90985916457013 4.59292294801308<br>C 3.69931835774000 -2.65192990196005 2.62458627186812<br>H 4.33940263396777 -3.44678561215663 2.98379511994647<br>C 0.09819779124145 -4.66047874928065 0.69106322521544<br>C 0.61565603662644 -4.32106151476889 1.93641648187163<br>C 1.04085768513055 -5.32103625489039 2.78347691310447<br>C 0.96345751572780 -6.65339800820050 2.41658245560011<br>C 0.45740895808562 -6.98674119062270 1.17141740964006<br>C -0.01566638282100 -6.00614030423192 0.26884185168190<br>H -0.28680249093109 -3.87439293930613 0.05080236427737<br>H 0.67986575657758 -3.28313780223430 2.23942929869344<br>H 1.30511618797534 -7.42511249870301 3.09592905026456<br>H 0.39420436652538 -8.03021160334159 0.88519090390836<br>O -0.50076642853834 -6.32742671036542 -0.89687260759902<br>H 0.27767258630629 -5.78162539277418 -1.83246649336474<br>O 4.53906434227750 -10.77729419782203 -2.72724510036156<br>C 5.48638734708610 -11.28378504042023 -3.64526397210715<br>H 5.86589974484325 -12.20807876332671 -3.21727893712691<br>H 5.03567472759000 -11.50442307740775 -4.61628837933433<br>H 6.32190184817509 -10.59424953900478 -3.79127589716707<br>F 1.54808780997542 -4.99930026537788 3.99536742109471	
57	Au 1.41357797616211 -4.35889279651135 0.16003265948192<br>P 3.65407965465197 -3.91982896515837 0.46777601914984<br>O 2.90429889890944 -4.97621357981182 -4.67791554640789<br>C 2.36101028409074 -5.76491517971183 -3.70644113408618<br>C 1.09263835752157 -5.37568201055924 -3.41533240490911<br>C 0.81648853209593 -4.25030964981150 -4.25659823128618<br>C -0.26718716489550 -3.39100753972305 -4.44440405129571<br>H -1.17766689054492 -3.51336993934748 -3.87177438590631<br>C -0.14748901266496 -2.37860269298809 -5.38089495892751<br>H -0.97495330847379 -1.70021671456278 -5.54233303996250<br>C 1.02434669855529 -2.21345039406359 -6.12905647254686<br>H 1.08306620491071 -1.41331864419969 -6.85489554706995<br>C 2.11315731934422 -3.05643521410065 -5.95961356782829<br>H 3.02100479328024 -2.93615305306428 -6.53625249150234<br>C 1.97366034716710 -4.05743485487098 -5.01619607726339<br>C 3.19866009406409 -6.82244117756172 -3.17355491769771<br>C 2.73418805562316 -7.61870692207416 -2.11832794823207<br>H 1.75073735220288 -7.44869272534600 -1.69683790852318<br>C 3.50976280889339 -8.63097594333925 -1.60034978540063<br>H 3.13917295179858 -9.23400258766622 -0.78147672515027<br>C 4.78276349229587 -8.88440493816444 -2.11874687317243<br>C 5.25694437475995 -8.10136897239290 -3.16662159872899<br>H 6.23616200951721 -8.26458758369171 -3.59522743667576<br>C 4.46842818681703 -7.08249015376503 -3.68237537336134<br>H 4.86450546001951 -6.49207819801385 -4.49767386753233<br>C 4.41501386511263 -2.89736349691843 -0.82290995190085<br>C 3.81805817998316 -2.80942116374203 -2.07749583347866	**PC*syn* (1_{OMe} and 2_F)**<br>$E_{PBEh\text{-}3c/CPCM(Chloroform)}$=<br>-2303.098015209274<br>$ZPE_{PBEh\text{-}3c/CPCM(Chloroform)}$=<br>0.61073510<br>$FEC_{(298.15)PBEh\text{-}3c/CPCM(Chloroform)}$=<br>0.54316315<br>$E_{PW6B95\text{-}D3(BJ)/def2\text{-}TZVPP/CPCM(Chloroform)}$=<br>-2310.963823115174

	H 2.88871574402924 -3.32829655672087 -2.27798951538545<br>C 4.40414539767147 -2.04148377708474 -3.07397923172097<br>H 3.92878099916691 -1.96924100065065 -4.04304575090962<br>C 5.58538074302302 -1.35971972036928 -2.82196323207950<br>H 6.03871960726745 -0.75800393532490 -3.59881390922908<br>C 6.18247412670189 -1.44149096355920 -1.57011669674500<br>H 7.10071319520016 -0.90577966767150 -1.36895842534765<br>C 5.59935144998135 -2.20468423452575 -0.57137488224518<br>H 6.06865953333081 -2.25354785432365 0.40333008900261<br>C 4.64745979255896 -5.43344272414320 0.59090170779935<br>C 5.77215079939490 -5.65523174726053 -0.19542842318278<br>H 6.07955933922189 -4.92907636743395 -0.93620470068765<br>C 6.50968467068491 -6.82083100047040 -0.03402744074880<br>H 7.38281241598844 -6.98837222279081 -0.65076267759604<br>C 6.13168514027755 -7.76291290317179 0.90929474617306<br>H 6.71222891798949 -8.66700657019838 1.03549567114475<br>C 4.99910868102935 -7.55018823708423 1.68575109641440<br>H 4.69242704308981 -8.28813875188790 2.41512029454870<br>C 4.25400403021052 -6.39466116470953 1.52310218980650<br>H 3.36788821771186 -6.23988977544562 2.12861268367560<br>C 3.97455761727213 -3.02180872000649 2.01739974786846<br>C 3.11902570718609 -1.97742097580737 2.36571527316057<br>H 2.26855254835768 -1.73392659798364 1.73950184515038<br>C 3.34899395365317 -1.24373523877180 3.51864407785137<br>H 2.67954937207132 -0.43594331127918 3.78289412544977<br>C 4.42950260238419 -1.55182495520558 4.33541730601373<br>H 4.60427269844122 -0.98293791599289 5.23918121545550<br>C 5.28095440458578 -2.59255420433806 3.99414041382191<br>H 6.12271082451081 -2.83685123848856 4.62855886705367<br>C 5.05734206445187 -3.32721297943480 2.83765137335662<br>H 5.72920596569468 -4.13693895398686 2.58372500008686<br>C -0.73435509310570 -4.73737410090254 -0.06605840254050<br>C -1.19933498219419 -3.92737373643808 1.03753352548300<br>C -1.35237397724738 -4.47953776145590 2.26219487525390<br>C -1.09125120387423 -5.84984268799083 2.48493504347244<br>C -0.73654892609569 -6.66629155352437 1.45624829184931<br>C -0.69183044419580 -6.19490393014749 0.07783703197324<br>H -0.99106050332604 -4.36993076375708 -1.06073778790533<br>H -1.41347793146057 -2.87810869510302 0.87225129831276<br>H -1.18626900351129 -6.24734683500623 3.48883352876036<br>H -0.58589661876885 -7.72424071550799 1.63369712000551<br>O -0.61113085125118 -6.97877073670835 -0.87841836570831<br>H 0.42470500628112 -5.84306722578421 -2.70672016498581<br>O 5.46698458315639 -9.89032799094789 -1.54892185360081<br>C 6.76944632048312 -10.16835365715237 -2.01297952815453<br>H 7.14106557248478 -10.99763118345098 -1.41588258523350<br>H 6.77606777138896 -10.46420636162702 -3.06572839091059<br>H 7.44074630128770 -9.31444002847814 -1.88452802296243<br>F -1.73972333686511 -3.73112631595704 3.31806984299057	
58	Au 2.15370239828317 -5.16467420302244 -1.94381021317247<br>P 1.35049501423930 -4.26352070058932 -0.04471046584160<br>C 0.71930208455171 -2.57511573645083 -0.26857363215937<br>C 1.44987519305664 -1.69028793688818 -1.06108311773282<br>H 2.35622614543973 -2.01784911817470 -1.55692278809675<br>C 1.01962007016787 -0.38283679841001 -1.22098759940382<br>H 1.59104750505601 0.29845126339863 -1.83719104927267<br>C -0.14589202847888 0.04694455962977 -0.59909417568630<br>H -0.48464128388406 1.06626629394463 -0.73030227381093<br>C -0.87806603118531 -0.83228417199947 0.18530056593151<br>H -1.78788333419812 -0.50169491964700 0.66857745746583<br>C -0.44746558363866 -2.14117152599547 0.35429203754860<br>H -1.02677806541167 -2.81676155888294 0.97017675699801<br>C 2.59269642478716 -4.14143032398855 1.27421999463550<br>C 2.66410842302078 -3.02890321823110 2.10808361596933<br>H 1.99256283479605 -2.19181242841712 1.96804589622271<br>C 3.60518037054398 -2.98559586488958 3.12746958355496<br>H 3.65858889497350 -2.11658284880703 3.76978098228487<br>C 4.47312290078804 -4.05050538245951 3.32088744224570<br>H 5.20704764810141 -4.01328726263408 4.11521259795646<br>C 4.40386900747910 -5.16217824790339 2.49099124277450<br>H 5.08193520610915 -5.99269941273236 2.63560218654851<br>C 3.47062422949578 -5.20729643549953 1.46739259439969<br>H 3.43002604983590 -6.07583869184819 0.82060470338825<br>C -0.02865463011948 -5.20148391140360 0.67329972837741<br>C -1.02705727733953 -5.67254702844109 -0.17879249477876	**4-F-PhOAuPPh$_3$**<br>$E_{PBEh\text{-}3c/CPCM(Chloroform)}$=<br>-1575.421287583973<br>$ZPE_{PBEh\text{-}3c/CPCM(Chloroform)}$=<br>0.37223068<br>$FEC_{(298.15)PBEh\text{-}3c/CPCM(Chloroform)}$=<br>0.31873246<br>$E_{PW6B95\text{-}D3(BJ)/def2\text{-}TZVPP/CPCM(Chloroform)}$=<br>-1580.462219091144

	H -0.96022497179447 -5.50494100542459 -1.24747594151984<br>C -2.11404179892468 -6.35962576758765 0.33724813229801<br>H -2.88556667511357 -6.72164132298798 -0.32917369243166<br>C -2.20676605733674 -6.58910823497146 1.70432384332804<br>H -3.05271643013303 -7.13162552240196 2.10557305965038<br>C -1.21201789377355 -6.12754642693860 2.55380699435211<br>H -1.27922898019463 -6.30742382631437 3.61857192783804<br>C -0.12461880026664 -5.43214371207315 2.04251810181135<br>H 0.64375931962200 -5.07586229061820 2.71624699545524<br>C 4.06581343379463 -6.34627357584389 -5.59038078196290<br>C 5.15018812873768 -6.04377871779439 -6.39761852238036<br>C 6.04113221193500 -5.06734306230958 -5.99596431850527<br>C 5.86582634466467 -4.39358438435784 -4.80552128856951<br>C 4.77746411671319 -4.70096046429939 -3.99993485619686<br>C 3.84460923975028 -5.68427781723313 -4.36848716433738<br>H 3.36628608956946 -7.11104024002172 -5.90484811469858<br>H 5.29741481715171 -6.56711324502438 -7.33411731389837<br>H 6.57132855234838 -3.63106244616700 -4.50001379231609<br>H 4.65835286479714 -4.16145461089002 -3.06654371527541<br>O 2.79250712751313 -6.02451958602962 -3.65025045682700<br>F 7.09934770047041 -4.76881243736856 -6.78120602516026	
59	C -0.31377546265889 -4.35148461413732 -1.53878919080530<br>C -0.32678133795492 -5.49831293385314 -0.75698555185743<br>C 0.82256794714607 -5.92789155270436 -0.10974598979316<br>C 1.99282525651481 -5.19221863188247 -0.25315800533565<br>C 2.02023540471247 -4.04562992860161 -1.03089598960721<br>C 0.86284734336893 -3.61966254961999 -1.67998415009862<br>H -1.22093965830406 -4.02939668823148 -2.03859446677384<br>H -1.24820318258691 -6.05754655187939 -0.65710043915430<br>H 2.89860225823893 -5.51301196342104 0.24533137306789<br>H 2.93490526082564 -3.47639574827084 -1.14027788700921<br>O 0.94057691611803 -2.49910040844426 -2.42713499220243<br>H 0.09029098513266 -2.30717514984840 -2.83673529285114<br>H 0.80794694844722 -6.82222754810568 0.49795116842041	**phenol (2_H)**<br>$E_{PBEh-3c/CPCM(Chloroform)}$=<br>-306.804889387712<br>$ZPE_{PBEh-3c/CPCM(Chloroform)}$=<br>0.10804597<br>$FEC_{(298.15)PBEh-3c/CPCM(Chloroform)}$=<br>0.07919207<br>$E_{PW6B95-D3(BJ)/def2-TZVPP/CPCM(Chloroform)}$=<br>-307.993240471476<br>$E_{B3LYP-D3(BJ)/def2-TZVPP/CPCM(Chloroform)}$=<br>-307.440649106190
60	Au 0.40143543884672 -3.94838599803623 -2.22499943459913<br>P 1.06042036765484 -3.56677711301544 -0.02374545886956<br>O -0.74185919381744 -3.70841113333276 -6.32397473527400<br>C -0.10897045459165 -3.27944408225999 -5.18403984236006<br>C -0.19509364489028 -4.18191247743299 -4.16244451442753<br>C -0.94871816715246 -5.27713616093648 -4.73136742063515<br>C -1.40449930736107 -6.50462395115468 -4.24674929817705<br>H -1.20014205338859 -6.80393098298075 -3.22534373737361<br>C -2.12452337437776 -7.32925594343198 -5.09482259935195<br>H -2.48337505588001 -8.28497676865625 -4.73539553648720<br>C -2.40152968582805 -6.94817225340257 -6.41304953513692<br>H -2.96659353630523 -7.61409038177877 -7.05186515757084<br>C -1.96812736030504 -5.73017502764751 -6.91644345665403<br>H -2.18280922915327 -5.42795220994331 -7.93317664306682<br>C -1.25128473600114 -4.92510056643529 -6.04952404641399<br>C 0.47435670226033 -1.94470616988231 -5.26800867152820<br>C 1.63392890850452 -1.61568571567252 -4.57115505679307<br>H 2.16512370416283 -2.37589665548503 -4.01409000134368<br>C 2.15976391065529 -0.33209254391306 -4.59420896662141<br>H 3.06595055868397 -0.13484575669781 -4.03765654926771<br>C 1.53238800694030 0.66151193290986 -5.34133014168724<br>C 0.38725208069160 0.33824811553709 -6.07245887738434<br>H -0.10036490371120 1.10428360141785 -6.66214876455418<br>C -0.12812484047516 -0.94131097669014 -6.03652257038131<br>H -1.02694394811635 -1.15387108918808 -6.60045992793605<br>C 1.17201058808785 -1.78709654138350 0.33876206799590<br>C 1.73014516810312 -0.95159205921746 -0.62855508741097<br>H 2.06990604483363 -1.35767207735461 -1.57384973157532<br>C 1.85022352955404 0.40832827324145 -0.39108472844221<br>H 2.27965787125640 1.04880486876311 -1.15081445327861<br>C 1.40888741293522 0.94560790852432 0.81137675650066<br>H 1.49604654032713 2.00860149839781 0.99435040502511<br>C 0.84955906472895 0.11881476025508 1.77519208975880<br>H 0.50179220230741 0.53400560834496 2.71191885963587<br>C 0.73053699360241 -1.24475882745972 1.54236960808748	**RC_{anti} (1_{OMe} and 2_H)**<br>$E_{PBEh-3c/CPCM(Chloroform)}$=<br>-2204.059416605667<br>$ZPE_{PBEh-3c/CPCM(Chloroform)}$=<br>0.61980862<br>$FEC_{(298.15)PBEh-3c/CPCM(Chloroform)}$=<br>0.55253542<br>$E_{PW6B95-D3(BJ)/def2-TZVPP/CPCM(Chloroform)}$=<br>-2211.585822319420<br>$E_{B3LYP-D3(BJ)/def2-TZVPP/CPCM(Chloroform)}$=<br>-2207.994224528026

	H 0.29029477127931 -1.87835824522084 2.30162733139865<br>C 2.68834199675154 -4.24220379015547 0.41614830855680<br>C 3.55068825565250 -3.57131565059457 1.28055888312777<br>H 3.27869718837471 -2.60605827748716 1.68817201174316<br>C 4.76962420595244 -4.13817098342123 1.62358871117930<br>H 5.43615036513228 -3.61066403454860 2.29315373487524<br>C 5.13173013247502 -5.37596465159529 1.10981923054452<br>H 6.08357615550305 -5.81560442258544 1.37797478189282<br>C 4.27620933602288 -6.04617840586827 0.24601249840598<br>H 4.55535793407932 -7.00575766831554 -0.16748715527424<br>C 3.06027647587926 -5.47982001984531 -0.10616389578018<br>H 2.40841411454847 -6.00699401202107 -0.79298220148983<br>C -0.08704818908813 -4.23155613391171 1.22025619551605<br>C -1.45550086996647 -4.06692704551484 1.00583082898624<br>H -1.81297307718384 -3.57278944182580 0.10971334300036<br>C -2.36794465525897 -4.53332772036710 1.93865659131008<br>H -3.42769743864321 -4.39960888739422 1.76650835254435<br>C -1.92205764608046 -5.17835182130281 3.08558106679288<br>H -2.63538958161068 -5.54830700737747 3.81037086171422<br>C -0.56233868420558 -5.35289282317473 3.29754297796996<br>H -0.21113027430814 -5.85853363968159 4.18735334981844<br>C 0.35570012622719 -4.87974081566438 2.36937672447130<br>H 1.41373683471335 -5.02148795490067 2.54748335716740<br>C 2.33362785983045 -7.13177692542753 -3.54821210514712<br>C 2.71091577908469 -8.29915386589838 -2.89914977911330<br>C 4.04686355821609 -8.56384957294419 -2.63188486888340<br>C 5.00808440812070 -7.63971152930563 -3.02421355662075<br>C 4.64515915988990 -6.46615793400573 -3.66520594528935<br>C 3.30179682063140 -6.20547416027100 -3.93124204291609<br>H 1.28745692632237 -6.94824568214374 -3.76399787988926<br>H 1.94731090652287 -9.00896759313357 -2.60729007852682<br>H 6.05516693125696 -7.83054718505518 -2.82577250174456<br>H 5.39756883323753 -5.74685607832907 -3.96370356779942<br>O 3.00064986589065 -5.05331606601801 -4.55972289173816<br>H 2.04182117020238 -4.93758326824221 -4.63011499773171<br>H 4.33613695411885 -9.47760406854718 -2.13020372576143<br>O 1.95550448155633 1.93533316305803 -5.42217903126791<br>C 3.09298985949691 2.31548652655291 -4.67929409272440<br>H 2.94850927093004 2.16711381323038 -3.60489401024508<br>H 3.98854798867626 1.77101620760334 -4.99105049491285<br>H 3.24616145873610 3.37533266321744 -4.86751097988731	
61	Au 0.65974136901644 -4.30533584336163 -1.78707056594437<br>P 0.85146760309408 -3.37898192502211 0.29929178894292<br>O -1.08446369533869 -3.88167954584399 -4.94428911041722<br>C 0.15445165462981 -3.81958813058538 -4.43526480578744<br>C 0.55145444207717 -5.01837887768514 -3.84022401832520<br>C -0.58974107145233 -5.89642503324987 -4.07081437268216<br>C -0.86241274555316 -7.23056175607733 -3.78148611910905<br>H -0.13312135577243 -7.84614871414521 -3.27082014654835<br>C -2.09042252159421 -7.74760530282539 -4.16375982741408<br>H -2.32276784730542 -8.78275924352453 -3.95092631559169<br>C -3.03896435773956 -6.95927510053927 -4.82263843711084<br>H -3.98769932406224 -7.39457677747862 -5.10673075263571<br>C -2.78556685281394 -5.62835944845072 -5.12536183345791<br>H -3.51028718790903 -5.01227004320066 -5.64058452046814<br>C -1.55248698080247 -5.14540652441469 -4.73710395381065<br>C 0.85998075509239 -2.55965636392392 -4.58344485914718<br>C 2.24214811590894 -2.51353970398650 -4.39994907471302<br>H 2.79296031096222 -3.42474249025027 -4.19927852348670<br>C 2.93309097276858 -1.31722334269081 -4.47100928460575<br>H 4.00432057710854 -1.32879972738740 -4.32527291353582<br>C 2.24383779000488 -0.13072877885759 -4.72813992474845<br>C 0.86164775249324 -0.17175216632587 -4.93287034178449<br>H 0.32771601859381 0.74720146269815 -5.13794794958627<br>C 0.18217564519626 -1.36869831783831 -4.86588428552063<br>H -0.88926284035721 -1.36695341198589 -5.01514800517617<br>C 0.75286321528848 -1.56707960962451 0.19495425781059<br>C 1.38267768451016 -0.92265529587858 -0.87017899443895<br>H 1.90432095583127 -1.49154234573676 -1.63146463568170<br>C 1.34345990742649 0.45909139852845 -0.96761404649895<br>H 1.83010312627894 0.95119364630352 -1.79952610602645<br>C 0.67109669807025 1.20540396782724 -0.00812969297929<br>H 0.63466049341507 2.28385456962023 -0.08928691458806<br>C 0.04011769281316 0.56743181011144 1.05009753954196<br>H -0.48732834512550 1.14542982174923 1.79730793938577	**TS$_{anti}$ (1$_{OMe}$ and 2$_{H}$)**<br>E$_{PBEh\text{-}3c/CPCM(Chloroform)}$=<br>-2204.015979213154<br>ZPE$_{PBEh\text{-}3c/CPCM(Chloroform)}$=<br>0.61445690<br>FEC$_{(298.15)PBEh\text{-}3c/CPCM(Chloroform)}$=<br>0.54742055<br>E$_{PW6B95\text{-}D3(BJ)/def2\text{-}TZVPP/CPCM(Chloroform)}$=<br>-2211.544601103587<br>E$_{B3LYP\text{-}D3(BJ)/def2\text{-}TZVPP/CPCM(Chloroform)}$=<br>-2207.955816455511

	C 0.07950999120990 -0.81628909330766 1.15466448771472<br>H -0.41898012886747 -1.30222831346750 1.98356431203572<br>C 2.43161736383342 -3.74264136225107 1.11166472135401<br>C 3.10320514772384 -2.78019370598759 1.86128821607865<br>H 2.70555106624835 -1.77762770359225 1.95299263057053<br>C 4.29509883118957 -3.10362223803207 2.49401437961878<br>H 4.81529519459514 -2.35177254474652 3.07255144395682<br>C 4.81817518374813 -4.38426968275841 2.38320141692797<br>H 5.74941280716673 -4.63272896797413 2.87515969516576<br>C 4.15117425527556 -5.34534418463872 1.63469822622432<br>H 4.55999378789966 -6.34238792851656 1.53958468873188<br>C 2.96319184213579 -5.02617546202177 0.99603699752476<br>H 2.45580886155430 -5.77924270696035 0.40450864014678<br>C -0.44029381008737 -3.87655032470791 1.47154629710135<br>C -1.75604174568921 -3.95176324534849 1.01567622512404<br>H -1.98907357475930 -3.74299960443481 -0.02205266293387<br>C -2.77482426411698 -4.29587413785273 1.88930067210413<br>H -3.79382192926505 -4.35186697873237 1.53020954344990<br>C -2.48557781704781 -4.57712596205033 3.21882460739983<br>H -3.28110580496769 -4.85260847334165 3.89865577471113<br>C -1.17650419048795 -4.51116155507784 3.67300916707984<br>H -0.94738458480920 -4.73375033844593 4.70668971707358<br>C -0.15282514929038 -4.15978181398076 2.80351322479447<br>H 0.86440783888581 -4.11132839694717 3.16984648257154<br>C 2.77776753031570 -7.03079329665341 -5.84609722679164<br>C 3.35101416976375 -7.75529715004832 -6.87879203852535<br>C 4.71294164497253 -8.03610476532993 -6.89475606374352<br>C 5.49239622833997 -7.57037966535675 -5.84037926181532<br>C 4.93446943201796 -6.84191074552096 -4.80317788117291<br>C 3.55155101673918 -6.54469885505344 -4.76487524362489<br>H 1.71114870076076 -6.83433329681532 -5.86277048391683<br>H 2.71994140470238 -8.10809939910875 -7.68675692864062<br>H 6.55686194164619 -7.77622000808098 -5.82694706599499<br>H 5.56138092887873 -6.48796495350550 -3.99217598925385<br>O 3.04021125262526 -5.86123056621961 -3.78482803734269<br>H 1.71847021765400 -5.43150442853257 -3.85171815134550<br>H 5.15389519315637 -8.60418561101800 -7.70325907249217<br>O 2.81660909680262 1.07820577296179 -4.79122643095648<br>C 4.21291053340156 1.18254737140860 -4.59318835925376<br>H 4.50970894983969 0.83775519935474 -3.59933131962079<br>H 4.77411580426212 0.62220145765703 -5.34503535109525<br>H 4.45830887128937 2.23727119108884 -4.68647567380051	
62	Au 1.86152825754027 -5.68301696736136 -1.67541668747471<br>P 1.17455750651334 -4.50784816136798 0.11374532317082<br>O -0.90112967884058 -2.60124508419094 -3.56441950224624<br>C 0.16948277517641 -3.08989952287365 -4.25084700679455<br>C 0.07356963517939 -4.43459855499299 -4.40950973898291<br>C -1.14981513902682 -4.82760170468091 -3.77907775958500<br>C -1.84042438380685 -6.02849154385315 -3.60648514925696<br>H -1.43770534527449 -6.95924349194265 -3.98464057605813<br>C -3.05632190476398 -5.99636608788274 -2.94415503791037<br>H -3.61120508067488 -6.91480206713526 -2.80296798859629<br>C -3.58281585644332 -4.79798993144737 -2.44664328598161<br>H -4.53263802550258 -4.80970600358708 -1.92867159731162<br>C -2.90949990989260 -3.59495149300973 -2.60087634696498<br>H -3.31034970109201 -2.66558839370915 -2.21843061744017<br>C -1.70396519325136 -3.64693118204924 -3.27552696341441<br>C 1.16795939931687 -2.13331925400642 -4.69019923406944<br>C 2.42184405064210 -2.57479020547472 -5.10650423745740<br>H 2.66523949097050 -3.62980730950992 -5.08417011522094<br>C 3.39482074995811 -1.68769319999909 -5.53697503246707<br>H 4.35248073969356 -2.08569433220536 -5.84350853222480<br>C 3.12622648157739 -0.32010307026568 -5.56009672803388<br>C 1.87216647811814 0.13388368094882 -5.14487262790829<br>H 1.65870198345526 1.19521949486370 -5.16334266093601<br>C 0.91127835052369 -0.75871288441278 -4.71643190888452<br>H -0.05010719568118 -0.37530572456954 -4.40198875525521<br>C 0.73175123047186 -2.79912615504959 -0.30893751250575<br>C 1.62694679635762 -2.06255938002222 -1.08380713904703<br>H 2.53667024113043 -2.51944116820884 -1.45628323105780<br>C 1.35885929895128 -0.73763353770796 -1.38371130613419<br>H 2.05882328076361 -0.17223340546263 -1.98360099515060<br>C 0.18698960810791 -0.14590457149295 -0.92983571410987<br>H -0.02633393300970 0.88693547549157 -1.17261951190493<br>C -0.71329184877953 -0.88092523247193 -0.17267741040546	**PC*anti* (1_{OMe} and 2_{H})**<br>$E_{PBEh-3c/CPCM(Chloroform)}$=<br>-2204.069777842991<br>$ZPE_{PBEh-3c/CPCM(Chloroform)}$=<br>0.61967281<br>$FEC_{(298.15)PBEh-3c/CPCM(Chloroform)}$=<br>0.55342064<br>$E_{PW6B95-D3(BJ)/def2-TZVPP/CPCM(Chloroform)}$=<br>-2211.596652126459

	H -1.62952916865728 -0.42442598577381 0.17807781087328<br>C -0.44179412616169 -2.20531029390710 0.14426591688745<br>H -1.14800701932527 -2.76630408693369 0.74225112203045<br>C 2.44826814350053 -4.35658882939034 1.40095598037235<br>C 2.60768901865362 -3.18192208799486 2.13174480153994<br>H 1.99292177563542 -2.31622398141930 1.92146196378529<br>C 3.56236831302537 -3.11368597938751 3.13669873643623<br>H 3.68364231082970 -2.19694812794380 3.69844985419013<br>C 4.35780348168325 -4.21516477368907 3.41867230661232<br>H 5.10302677927422 -4.15860219022624 4.20127414641774<br>C 4.20094475162338 -5.38850237007368 2.69239193990357<br>H 4.82203817647438 -6.24818635603348 2.90616422424494<br>C 3.25243856630793 -5.45946569111766 1.68397339037351<br>H 3.14298028194323 -6.37695374329074 1.11785554925882<br>C -0.27563339214497 -5.20027918302882 0.95626271115487<br>C -1.32108184449693 -5.69311858642368 0.17796221423641<br>H -1.25247530755267 -5.68743322061164 -0.90332158736432<br>C -2.46211677136957 -6.19375523687905 0.78457400289307<br>H -3.26974057652755 -6.57207009657237 0.17207854938813<br>C -2.56199622166449 -6.21495444256820 2.16950004146020<br>H -3.45021535583855 -6.61306687022099 2.64248887387632<br>C -1.52003957719536 -5.72963996198364 2.94731288767527<br>H -1.59313670777750 -5.74727802575554 4.02668295935334<br>C -0.37850641287128 -5.21999225602280 2.34477022177572<br>H 0.42647377079038 -4.84406553551336 2.96267619316841<br>C 3.08503626421260 -6.54063365370977 -5.53675311598457<br>C 3.92397378979544 -6.03404031690390 -6.51753520506195<br>C 5.01061947545379 -5.23381682081456 -6.18538751654114<br>C 5.24531834553765 -4.95738268518611 -4.84372460842761<br>C 4.41166070166888 -5.45577138455319 -3.85448228004571<br>C 3.30161325437373 -6.25818592957803 -4.17598175916802<br>H 2.24057365482583 -7.16154681753397 -5.81326409426801<br>H 3.72102432020199 -6.26522551109588 -7.55633529614669<br>H 6.08963344071831 -4.34073810264283 -4.55924524238278<br>H 4.62121407544976 -5.22356550161401 -2.81535805550517<br>O 2.48111131621730 -6.76106334392059 -3.27249889034235<br>H 0.76664859927079 -5.06549471098718 -4.94226261338855<br>H 5.66193708121571 -4.83860367888668 -6.95363579035688<br>O 4.00012547705284 0.61978443577775 -5.95790878109112<br>C 5.28027406013857 0.21133266308056 -6.39105484875988<br>H 5.83692708844272 -0.29865235614639 -5.60011829897765<br>H 5.22713636277063 -0.44611513528572 -7.26303572931565<br>H 5.81610082147637 1.11600240221080 -6.66771623953594	
63	Au 3.07349187119365 -4.26578496235210 -1.64989017325557<br>P 4.40898442552297 -3.49748318567636 0.09657536189200<br>O 0.91731207411633 -6.06287560947854 -4.87596533998304<br>C 1.86774158515502 -6.05431015836337 -3.88325264333255<br>C 1.84092749348204 -4.90743136744392 -3.14310332074680<br>C 0.75826848142105 -4.14438784654935 -3.72435081234451<br>C 0.18611239213740 -2.89990660296385 -3.45332068508608<br>H 0.56281860324097 -2.28473199946256 -2.64452782651469<br>C -0.87035107951113 -2.46825056109103 -4.23760129992645<br>H -1.32636437073065 -1.50665573620828 -4.04050240810967<br>C -1.36273883167361 -3.25441984538697 -5.28622582548622<br>H -2.18946076809972 -2.88986131710870 -5.88169024111630<br>C -0.81143609008087 -4.49389669027443 -5.57711658582271<br>H -1.18808957144602 -5.10650238612339 -6.38584143323982<br>C 0.24177556268256 -4.90243575569260 -4.77851927230756<br>C 2.70751897617015 -7.24534820115063 -3.83674403200738<br>C 3.28083859948415 -7.68576015197591 -2.63828187419207<br>H 3.07264646026110 -7.16665085026262 -1.71240862215058<br>C 4.09406716489120 -8.79868749689072 -2.59862549600218<br>H 4.52283393246414 -9.12387763899839 -1.65903106416379<br>C 4.35350747577620 -9.52814528429056 -3.76112328079319<br>C 3.77469570810480 -9.11705611308114 -4.95871512430629<br>H 3.94502459125466 -9.65354225598809 -5.88212417948642<br>C 2.96283328125411 -7.99173428396277 -4.98598694585045<br>H 2.53789264922522 -7.69229662333569 -5.93492556387564<br>C 5.91916000938276 -2.62130146432633 -0.40446841907291<br>C 6.60322425861993 -3.06768734415286 -1.53403746740533<br>H 6.21869206871597 -3.89972239888941 -2.11236514369522<br>C 7.78001334443907 -2.44785060553466 -1.92581094586196<br>H 8.30576735847553 -2.80005975884318 -2.80336474390644<br>C 8.27494200338131 -1.37316437917196 -1.19886265104420<br>H 9.18962742932631 -0.88530991770516 -1.50939865264009	**RC$_{syn}$ (1$_{OMe}$ and 2$_{H}$)**<br>$E_{PBEh-3c/CPCM(Chloroform)}$=<br>-2204.061184719712<br>$ZPE_{PBEh-3c/CPCM(Chloroform)}$=<br>0.61959935<br>$FEC_{(298.15)PBEh-3c/CPCM(Chloroform)}$=<br>0.55251666<br>$E_{PW6B95-D3(BJ)/def2-TZVPP/CPCM(Chloroform)}$=<br>-2211.589112643128<br>$E_{B3LYP-D3(BJ)/def2-TZVPP/CPCM(Chloroform)}$=<br>-2207.997532043526

	C 7.59285295148344 -0.92086788626876 -0.07778764747413<br>H 7.97402326962591 -0.08125509510663 0.48837409231818<br>C 6.41857528403382 -1.54298491835819 0.32172849254860<br>H 5.89509671383719 -1.18214070004233 1.19794699383386<br>C 4.96677763614911 -4.83657865373045 1.19136549509198<br>C 6.27826646871466 -4.93457263161554 1.64556470558695<br>H 7.01960047787766 -4.20379201131177 1.34932360317296<br>C 6.64530238392181 -5.97920044755585 2.48379394504108<br>H 7.66761335894330 -6.05280393201408 2.83065483726224<br>C 5.70684765687457 -6.92343640740961 2.87449905582196<br>H 5.99645481547005 -7.73659814253228 3.52722665196396<br>C 4.39598549810940 -6.82613829826092 2.42428679913842<br>H 3.65853225817592 -7.55921868586289 2.72382952500100<br>C 4.02700082147945 -5.79098928794542 1.58072300676117<br>H 3.00237434490903 -5.72776435345027 1.23313292428406<br>C 3.51996702444096 -2.33748581519412 1.17834065289290<br>C 2.73117618435443 -1.35443359630402 0.58180727655342<br>H 2.65976498336226 -1.29106111670649 -0.49804077074837<br>C 2.02748549300533 -0.45448543194071 1.36631829257783<br>H 1.41650420558122 0.30485832037147 0.89634124597972<br>C 2.09920359434863 -0.53543513274887 2.75170538253891<br>H 1.54243362105454 0.16173238756154 3.36413627403469<br>C 2.88166885442632 -1.51333715221509 3.34845240875278<br>H 2.93902370026094 -1.58069217624525 4.42690547237562<br>C 3.59279403823226 -2.41269192348749 2.56541657362686<br>H 4.19487821750117 -3.17460908851727 3.04320167213931<br>C 0.33730097018544 -4.65343797430294 0.53703262255466<br>C 0.48248466709899 -4.10439069906845 1.80341301277154<br>C 0.60210903558079 -4.91889337017490 2.92051870321909<br>C 0.57087780152954 -6.29836322489700 2.75707883819880<br>C 0.44137062166397 -6.86087026752662 1.49628443109679<br>C 0.32876791242505 -6.03880030903339 0.37556129510265<br>H 0.21906741406505 -4.00351394532279 -0.32277751723115<br>H 0.50321411296479 -3.02736553286306 1.91212356099195<br>H 0.65898859303691 -6.94827077491958 3.61853884020963<br>H 0.43054177051425 -7.93654550735170 1.37100737417551<br>O 0.20940861409010 -6.63204674571197 -0.82750140076130<br>H 0.36154970354162 -5.99517968045594 -1.53940447939610<br>O 5.15536350693405 -10.59962954110104 -3.62803184049276<br>C 5.43551652766718 -11.37877362877669 -4.77049879646658<br>H 6.08806737815313 -12.18462114741962 -4.44325678482148<br>H 4.52998874078155 -11.81416589342892 -5.20202965512367<br>H 5.95093360585420 -10.80252100616419 -5.54389044195127<br>H 0.71761672209266 -4.48465381309034 3.90447840041738	
64	Au 2.51433636039749 -4.48904684021478 -1.33954837010021<br>P 4.14373646244575 -3.62374584205215 0.02821502713537<br>O 1.77563742990182 -5.47490338832751 -4.84846053645893<br>C 1.77821307240185 -5.99850437407621 -3.61007218713449<br>C 1.02277308818370 -5.25140853388872 -2.70749681285771<br>C 0.49015942713283 -4.17583121705356 -3.53821975308230<br>C -0.35385465294510 -3.09407505428877 -3.30039793010027<br>H -0.76504519621910 -2.92004391438614 -2.31405499927684<br>C -0.65164372568968 -2.24648134621870 -4.35598839932549<br>H -1.30709941511551 -1.40099040959902 -4.19300052975116<br>C -0.12294857695264 -2.46129854838427 -5.63259573716946<br>H -0.37543699047322 -1.78068738685431 -6.43471348839404<br>C 0.71794407808930 -3.53482986831671 -5.89275950114496<br>H 1.12856839937210 -3.71146861064427 -6.87796036684549<br>C 0.99093751998233 -4.36281771688999 -4.82257083542488<br>C 2.51402722153319 -7.23589046341804 -3.43465937589721<br>C 2.31927879466130 -8.00635245666598 -2.28011304078223<br>H 1.59054392614893 -7.69922887324481 -1.54134866861914<br>C 3.02121422271079 -9.17253646462525 -2.08342239618100<br>H 2.85796409990837 -9.76218323924453 -1.19042255111604<br>C 3.94402434776902 -9.61329512850998 -3.03920617837116<br>C 4.13707042627147 -8.86275571835002 -4.19774903953443<br>H 4.83729244668601 -9.17274349895731 -4.96093345107045<br>C 3.42454083228319 -7.68913975279049 -4.38710199308880<br>H 3.60060512620777 -7.12306506476787 -5.29198143718993<br>C 5.44026832745726 -2.69475790337700 -0.83946522272247<br>C 5.91348886397380 -3.18804918786690 -2.05449071407375<br>H 5.48613641238966 -4.08741665158616 -2.48279576370050<br>C 6.93306956998128 -2.52874574977271 -2.72271236301335<br>H 7.29613894400671 -2.91677175934982 -3.66513287525593<br>C 7.47912847040322 -1.36884810909410 -2.18776817134412	**TS$_{syn}$ (1$_{\mathrm{OMe}}$ and 2$_{\mathrm{H}}$)**<br>$E_{\mathrm{PBEh\text{-}3c/CPCM(Chloroform)}}$=<br>-2204.023461722398<br>$ZPE_{\mathrm{PBEh\text{-}3c/CPCM(Chloroform)}}$=<br>0.61438612<br>$FEC_{\mathrm{(298.15)PBEh\text{-}3c/CPCM(Chloroform)}}$=<br>0.54838107<br>$E_{\mathrm{PW6B95\text{-}D3(BJ)/def2\text{-}TZVPP/CPCM(Chloroform)}}$=<br>-2211.553867608308<br>$E_{\mathrm{B3LYP\text{-}D3(BJ)/def2\text{-}TZVPP/CPCM(Chloroform)}}$=<br>-2207.966101402908

	H 8.27028926486591 -0.85082034934464 -2.71381742657882<br>C 7.00621264309205 -0.87160646232065 -0.98181192121204<br>H 7.42761589050380 0.03301505052974 -0.56376399793688<br>C 5.99006444055166 -1.53242991110564 -0.30539090736874<br>H 5.63010438558680 -1.13672787801194 0.63565896508530<br>C 5.01513424344195 -4.90416650896455 0.97283533456284<br>C 6.36983364411819 -4.79538152229287 1.27801618790756<br>H 6.94609058621370 -3.94680568090134 0.93211383281116<br>C 6.99195172026282 -5.78328144349860 2.02770606274227<br>H 8.04534096058007 -5.69648754091524 2.25912208318714<br>C 6.26647453407200 -6.87822061430420 2.47762396399914<br>H 6.75520425530271 -7.64795656550997 3.06057442237091<br>C 4.91590068527005 -6.98801886592549 2.17502315495453<br>H 4.34586173118749 -7.84033275961506 2.52072882887945<br>C 4.29043912331872 -6.00758698686319 1.42021515068628<br>H 3.23631076433550 -6.10757604771976 1.18973703662046<br>C 3.45925035888209 -2.47431353209794 1.25434982648069<br>C 2.59218004598880 -1.47782598325554 0.80769211432844<br>H 2.35229619520774 -1.39262119823506 -0.24613421835793<br>C 2.02319779620796 -0.59563750653333 1.71117633956390<br>H 1.35036089228414 0.17501417063152 1.35901468162463<br>C 2.30663365118626 -0.71016652109946 3.06713005503235<br>H 1.85343501506481 -0.02757120989694 3.77403696862974<br>C 3.16626004245970 -1.70262070536821 3.51386198800882<br>H 3.38690728747123 -1.79636936168790 4.56896463917895<br>C 3.74543144670307 -2.58396349717246 2.61027361743541<br>H 4.40952599492286 -3.35805778081861 2.97183723851623<br>C 0.11091532374642 -4.69111464652400 0.69894536743919<br>C 0.60953126842653 -4.36416553485773 1.95443730850434<br>C 1.01133944015175 -5.35203943828401 2.84169990036592<br>C 0.90804661348052 -6.68515511523817 2.44881790067915<br>C 0.42815318040055 -7.02425530662979 1.19613088199332<br>C -0.01197102149284 -6.03837632390031 0.28093082635595<br>H -0.25574007370877 -3.90455680618704 0.04738577135297<br>H 0.67959837535491 -3.32115118679803 2.24078860860779<br>H 1.21812929940020 -7.47098989503914 3.12829831154886<br>H 0.35414917846470 -8.06702388974294 0.90748383390969<br>O -0.47959097492846 -6.35848281607361 -0.89106198823095<br>H 0.29008480085858 -5.79369184382024 -1.82123762102072<br>O 4.58415629961124 -10.75611733644356 -2.76041610809430<br>C 5.52181216156754 -11.25711914304154 -3.69118611668201<br>H 5.91219140105637 -12.17939423137317 -3.26863512915608<br>H 5.05935309062322 -11.48005955072333 -4.65615526907417<br>H 6.35146475368151 -10.56294213059941 -3.84828324744326<br>H 1.39623931831992 -5.09249977661027 3.81919552868394	
65	Au 1.49012497035590 -4.33321896613711 0.13745439013147<br>P 3.73468756093519 -3.99150573767045 0.51778101206616<br>O 2.69769713752803 -4.92088722931309 -4.74522449751578<br>C 2.20055890097828 -5.74435850268478 -3.77618097664160<br>C 0.91578544189605 -5.42392974472542 -3.47715104718761<br>C 0.57584163592355 -4.30951246125366 -4.30935947595014<br>C -0.55329345200841 -3.50876059012712 -4.48698253980682<br>H -1.45427299844500 -3.68536788657166 -3.91354374189608<br>C -0.49072721081801 -2.48292810307846 -5.41444531705100<br>H -1.35419125949339 -1.84913659896256 -5.56847102597079<br>C 0.66936942207451 -2.24680840591440 -6.16182923536358<br>H 0.68351744847826 -1.43679523631887 -6.87890452561141<br>C 1.80298874547245 -3.03048631734581 -6.00152482490863<br>H 2.70261730516367 -2.85337259617692 -6.57620835991106<br>C 1.71848362349258 -4.04764471847377 -5.06974391471530<br>C 3.09781255472675 -6.75056480642051 -3.24025512344330<br>C 2.66290741761836 -7.59566683100757 -2.21063507833244<br>H 1.65922160909369 -7.49878385851859 -1.81444512170053<br>C 3.49267444022619 -8.56190075520067 -1.68867032577307<br>H 3.14210767368420 -9.20687050073910 -0.89341170033698<br>C 4.79246963602719 -8.71993140248734 -2.17878814398897<br>C 5.24149654748467 -7.87929305154293 -3.19313160953884<br>H 6.24163787552399 -7.96420457454622 -3.59528700094641<br>C 4.39790566693478 -6.90739517533334 -3.71317554226554<br>H 4.77508579550034 -6.27193257839339 -4.50322349759436<br>C 4.57133452461285 -3.01587359566232 -0.76277722729999<br>C 4.18898435445618 -3.19164222066805 -2.09049314532744<br>H 3.37901844565078 -3.86657190796846 -2.33408898516443<br>C 4.83964361410032 -2.50017463486032 -3.10049590871147<br>H 4.53852635602833 -2.64356742913905 -4.12950283797195	**PC*syn* (1$_{OMe}$ and 2$_{H}$)**<br>$E_{PBEh\text{-}3c/CPCM(Chloroform)}$=<br>-2204.047811513738<br>$ZPE_{PBEh\text{-}3c/CPCM(Chloroform)}$=<br>0.61922575<br>$FEC_{(298.15)PBEh\text{-}3c/CPCM(Chloroform)}$=<br>0.55248740<br>$E_{PW6B95\text{-}D3(BJ)/def2\text{-}TZVPP/CPCM(Chloroform)}$=<br>-2211.578518137013

	C	5.86790285191247	-1.62126180635860	-2.78753806650385	
	H	6.37154355340026	-1.07582986520136	-3.57488284786561	
	C	6.24596927803133	-1.43649359871240	-1.46453822727678	
	H	7.04384756298808	-0.74843894759196	-1.21827864226164	
	C	5.60233939258543	-2.13244254404322	-0.45151364878541	
	H	5.90628693573431	-1.98067152313278	0.57625927169564	
	C	4.67468234237229	-5.53630226999960	0.66377304942968	
	C	5.91548003615347	-5.70490307432024	0.05778229658484	
	H	6.33809184163858	-4.92273002327947	-0.55921751088544	
	C	6.62165423210489	-6.88547251534238	0.24387765616410	
	H	7.58634687751861	-7.01078308554875	-0.23013525852150	
	C	6.09577783970143	-7.89713879294435	1.03257880016589	
	H	6.64993334351241	-8.81494226611535	1.17714097772763	
	C	4.85272685037239	-7.73489903764498	1.63065713756916	
	H	4.43341599454900	-8.52601493189233	2.23798858724934	
	C	4.14007154005947	-6.56216336204948	1.44294713722675	
	H	3.16656939537897	-6.44821416285147	1.90634452124489	
	C	4.04444895385194	-3.09941325623182	2.07107988692407	
	C	3.23737611290898	-2.00536288791561	2.38071553880014	
	H	2.42759816103534	-1.71777490648295	1.72012943612494	
	C	3.46510389681252	-1.27793318047463	3.53793524046468	
	H	2.83486806427619	-0.43013104430752	3.77153450698344	
	C	4.49313618914300	-1.64301985481204	4.39804797820201	
	H	4.66591419134980	-1.07866481055886	5.30504356539419	
	C	5.29432097507485	-2.73455263288560	4.09612982658825	
	H	6.09423331828229	-3.02347482824268	4.76498082423231	
	C	5.07410044816347	-3.46244590178958	2.93462471018582	
	H	5.70680689983655	-4.31137688836758	2.71011635410821	
	C	-0.66617801452699	-4.63416264083909	-0.15361801443436	
	C	-1.07718660296162	-3.72457437136929	0.88458022450764	
	C	-1.26715999957496	-4.14180036836363	2.16995153484509	
	C	-1.08315299652753	-5.51605707059637	2.46828113010852	
	C	-0.77722071369577	-6.43569256631289	1.50918960670718	
	C	-0.69983434599800	-6.07851242367785	0.10215861024930	
	H	-0.87350285432987	-4.32542698329914	-1.17920647462228	
	H	-1.22946222402642	-2.68372822062291	0.62126611782133	
	H	-1.20332653664303	-5.84784204076791	3.49345145289585	
	H	-0.69530645400664	-7.48539704047836	1.76683788184513	
	O	-0.65471122502870	-6.93307330521712	-0.79564269501922	
	H	0.28363720876588	-5.92401886714059	-2.75829061048394	
	O	5.52308669149214	-9.69939363106071	-1.62133753153534	
	C	6.84895956019875	-9.88721098998470	-2.06302723042892	
	H	7.25554358580693	-10.71162666939369	-1.48214564908865	
	H	6.89471479880856	-10.14967240359058	-3.12368270967757	
	H	7.46631530365457	-9.00042718113485	-1.89417817720367	
	H	-1.54694599309466	-3.44542129174147	2.94822858790625	
66	Au	1.58741560060644	-5.07817296259164	-2.02111043161542	**4-PhOAuPPh$_3$**
	P	1.03162203921587	-4.19320516987911	-0.02826450514556	$E_{PBEh-3c/CPCM(Chloroform)}$=
	C	0.46086155345652	-2.47203062195965	-0.13149237919786	-1476.369175128465
	C	1.14775589071532	-1.59289777987588	-0.96819906261237	$ZPE_{PBEh-3c/CPCM(Chloroform)}$=
	H	1.98381897851274	-1.94203792867341	-1.56293098672702	0.38060380
	C	0.76425878127628	-0.26356652412878	-1.04467385241332	$FEC_{(298.15)PBEh-3c/CPCM(Chloroform)}$=
	H	1.30177099821821	0.41330160297410	-1.69532784252345	0.32832475
	C	-0.31212580943629	0.19417146834696	-0.29509216780140	$E_{PW6B95-D3(BJ)/def2-TZVPP/CPCM(Chloroform)}$=
	H	-0.61529941976517	1.23086084072024	-0.36113083937951	-1481.076295189504
	C	-1.00160728591312	-0.67927857830163	0.53319463070206	
	H	-1.84267518723553	-0.32707551540889	1.11557494041062	
	C	-0.61660421520488	-2.01034187656219	0.61902456094380	
	H	-1.16221333722599	-2.68118347666253	1.26983967808146	
	C	2.42403081554797	-4.17060400550264	1.13784082209352	
	C	2.67170026233867	-3.07661333791446	1.96199157308525	
	H	2.04669120499307	-2.19465791307061	1.90887922515819	
	C	3.73020478002823	-3.11006414821467	2.85956698058846	
	H	3.92018769452033	-2.25517521706564	3.49493375709569	
	C	4.54029939027649	-4.23319613648510	2.94094128256849	
	H	5.36581459340380	-4.25593953069186	3.64022084146831	
	C	4.29510858451288	-5.32699240698561	2.12039442050138	
	H	4.92706457203711	-6.20323865151586	2.17756577624424	
	C	3.24380639669575	-5.29598562435759	1.21819766887183	
	H	3.06517423736605	-6.15188066950705	0.57773903189202	
	C	-0.29650859229184	-5.09235759483013	0.82281515829313	
	C	-1.38460227543548	-5.54119714626353	0.07542447815452	
	H	-1.41651098031121	-5.38321638810507	-0.99621634904366	
	C	-2.43448803351694	-6.19544281537717	0.70012261659430	
	H	-3.27571962850638	-6.54128822215211	0.11433093333221	

	C -2.40094426123212 -6.41327007376983 2.07160192766525<br>H -3.21812072059601 -6.93026954886996 2.55731358050229<br>C -1.31696032071803 -5.97316969477169 2.81733717732360<br>H -1.28591367335474 -6.14431948453331 3.88521190955329<br>C -0.26604030553857 -5.31153761068115 2.19724548969771<br>H 0.57349738768552 -4.97246259381579 2.79021462829236<br>C 3.23831178686196 -6.58793779033339 -5.67434053350950<br>C 4.44421635464449 -6.71666280995364 -6.34236883604470<br>C 5.63090647140213 -6.26998610207208 -5.77131217565400<br>C 5.57944283681179 -5.69023324068295 -4.51079214503398<br>C 4.37757068438617 -5.55552800434542 -3.83083814496247<br>C 3.17009287117834 -6.00016039402898 -4.39682115432807<br>H 2.32308223269901 -6.94077604808740 -6.13502849472735<br>H 4.45611821442185 -7.17322733227897 -7.32492779321050<br>H 6.48951654777861 -5.33465920649073 -4.04249337496017<br>H 4.37851361755810 -5.09772782772515 -2.84660292547382<br>O 1.99252795741890 -5.90302629753356 -3.81366532634958<br>H 6.57148975571362 -6.37200437398431 -6.29601499640020	
67	C -0.29076175193252 -4.36356289087008 -1.50772862114314<br>C -0.29953094127993 -5.49783323333636 -0.71547472506905<br>C 0.86165408636923 -5.93207715158215 -0.07962285211513<br>C 2.03338348461384 -5.20238905733461 -0.25512673378371<br>C 2.03970021549723 -4.06369679721317 -1.05188995583190<br>C 0.88247890763841 -3.63315532866637 -1.68547931403744<br>H -1.21002480923453 -4.04998994314903 -1.99030897674724<br>H -1.21813928817327 -6.05654841286954 -0.58651599982425<br>H 2.95921131968186 -5.50013999359958 0.21826865742555<br>H 2.95983170963537 -3.50726106301654 -1.17945792445189<br>O 0.94858906319550 -2.51802215790880 -2.45044142109855<br>H 0.09087050973569 -2.33324120335794 -2.84633778018085<br>O 0.75585231747532 -7.05094538909418 0.67322026707733<br>C 1.90883577997259 -7.52212605591712 1.33035527673214<br>H 1.61452294692099 -8.41809474159909 1.87227007771615<br>H 2.70572278673570 -7.78547076574848 0.62823733426067<br>H 2.30237530414851 -6.79472078773690 2.04683469207129	**4-methoxyphenol (2$_{OMe}$)**<br>$E_{PBEh-3c/CPCM(Chloroform)}$=<br>-421.085545605927<br>$ZPE_{PBEh-3c/CPCM(Chloroform)}$=<br>0.14159031<br>$FEC_{(298.15)PBEh-3c/CPCM(Chloroform)}$=<br>0.10948152<br>$E_{PW6B95-D3(BJ)/def2-TZVPP/CPCM(Chloroform)}$=<br>-422.697506141063<br>$E_{B3LYP-D3(BJ)/def2-TZVPP/CPCM(Chloroform)}$=<br>-346.748057296124
68	Au 0.53702515595870 -4.02763026034694 -2.57531749820274<br>P 1.22316595203678 -3.86266180874457 -0.35528453616329<br>O -0.82377366854650 -3.29886557335678 -6.54710445326646<br>C -0.14248602437812 -3.00066517948242 -5.39364865393934<br>C -0.13672045390792 -4.03605021122815 -4.50249930601818<br>C -0.88355724950428 -5.07551069306701 -5.17603174510612<br>C -1.26778118067619 -6.37537825559420 -4.84116200384959<br>H -0.99490754343446 -6.80077280315021 -3.88243556751742<br>C -2.00626616346515 -7.10763655024849 -5.75559035847067<br>H -2.31058677441404 -8.11745456068406 -5.51262432770946<br>C -2.37187248605596 -6.56413892561905 -6.99263963759009<br>H -2.94942603617302 -7.16101233946649 -7.68618715012368<br>C -2.01088834729805 -5.27207382292481 -7.34628511796932<br>H -2.29433383729183 -4.84469427504882 -8.29924864262389<br>C -1.27346966006062 -4.56204084364373 -6.41598952539611<br>C 0.38843184191409 -1.64359744222856 -5.32398557893195<br>C 1.56785009153759 -1.36144343217018 -4.63977063981204<br>H 2.15266888896352 -2.16549532177432 -4.21305576542995<br>C 2.04462935010103 -0.06493523621177 -4.51247709765166<br>H 2.96858913314872 0.09492869729510 -3.97339697958144<br>C 1.34629946938207 0.99150161250356 -5.09152527289781<br>C 0.18048198866808 0.71990705446096 -5.81080504052698<br>H -0.36225364045804 1.53595696182746 -6.27100705577120<br>C -0.28576895253457 -0.57381083804087 -5.92506252081630<br>H -1.20207171691937 -0.74762951254356 -6.47386458383593<br>C 1.24261415281889 -2.12188963615077 0.17697680589635<br>C 1.78552064755428 -1.17616575727829 -0.69273303581747<br>H 2.17093202785004 -1.47867096917343 -1.65912855645269<br>C 1.83288447600340 0.16010343734911 -0.32959040708205<br>H 2.25083683503203 0.88706693977561 -1.01430605205760<br>C 1.33339109212871 0.56377509297902 0.90218285924366<br>H 1.36370384939787 1.60840296335056 1.18308321920194<br>C 0.78930427981287 -0.37301076076518 1.76914241480710<br>H 0.39680576801186 -0.06208736840500 2.72835986421770<br>C 0.74309372857104 -1.71362184217319 1.41023825768525<br>H 0.31445551488041 -2.43396584776781 2.09509530245723<br>C 2.88445085822679 -4.48904212300407 0.02965341281965<br>C 3.70096136831845 -3.85678785776372 0.96508335231233	**RC$_{anti}$ (1$_{OMe}$ and 2$_{OMe}$)**<br>$E_{PBEh-3c/CPCM(Chloroform)}$=<br>-2318.340824060378<br>$ZPE_{PBEh-3c/CPCM(Chloroform)}$=<br>0.65338192<br>$FEC_{(298.15)PBEh-3c/CPCM(Chloroform)}$=<br>0.58397144<br>$E_{PW6B95-D3(BJ)/def2-TZVPP/CPCM(Chloroform)}$=<br>-2326.291457873531<br>$E_{B3LYP-D3(BJ)/def2-TZVPP/CPCM(Chloroform)}$=<br>-2322.512466085187

	H 3.37654049025921 -2.94508016407263 1.45019002408653<br>C 4.93966349755906 -4.39472113586041 1.28224837378182<br>H 5.57027284287358 -3.89708635867634 2.00718645355578<br>C 5.36510172244543 -5.56789806823196 0.67449128109991<br>H 6.33014478980045 -5.98859206864173 0.92519979301394<br>C 4.55639848417514 -6.19832248676977 -0.26134261550623<br>H 4.88907733792188 -7.10892671883449 -0.74030173128502<br>C 3.32267927166715 -5.65711955721524 -0.59217009160641<br>H 2.70691424601011 -6.14900959462827 -1.33661465068707<br>C 0.11798813870598 -4.70430494407646 0.81895391985149<br>C -1.25819951027030 -4.60754716507987 0.61298188208528<br>H -1.64660263003798 -4.05940470589306 -0.23758005618420<br>C -2.13953500985761 -5.21121628962423 1.49578380494918<br>H -3.20551572091029 -5.12927506479464 1.32973627694204<br>C -1.65395166010393 -5.92567650871950 2.58375348576108<br>H -2.34225796780499 -6.40269794188707 3.26911413007369<br>C -0.28585342517293 -6.03173947010746 2.78719615476185<br>H 0.09681036433273 -6.59036664875067 3.63116253666568<br>C 0.60049484056891 -5.42168300605355 1.90963241487473<br>H 1.66521054051065 -5.51214848089383 2.08096561605000<br>C 2.50463629462427 -6.81480011619283 -4.27155025400182<br>C 2.94955833062747 -7.96710559723106 -3.62892931947871<br>C 4.29907548982183 -8.13166594338545 -3.34371541618853<br>C 5.19306783444749 -7.12745951205205 -3.71656903505425<br>C 4.74754595453087 -5.98065929031404 -4.34430625959036<br>C 3.39278612893865 -5.80965390180617 -4.62714208171247<br>H 1.44982866251083 -6.71591283287319 -4.49859205806396<br>H 2.21873408710912 -8.72031273070871 -3.36668669780062<br>H 6.24719839586000 -7.24762228950284 -3.49906783413303<br>H 5.45375234436766 -5.20652621944935 -4.61750220897548<br>O 3.01308725913212 -4.66987856832287 -5.24646960571788<br>H 2.04918315815653 -4.58140546615577 -5.22766930837931<br>O 4.82919497385552 -9.20343945741919 -2.70751806769999<br>C 3.95624245740384 -10.23148069497134 -2.30242982720909<br>H 3.20355334553017 -9.87676835978228 -1.59153646119661<br>H 3.44263479056923 -10.69492701086575 -3.15022228084088<br>H 4.56759583826432 -10.98630014468869 -1.81286372288533<br>O 1.71751416299234 2.28181732038906 -5.01678400709249<br>C 2.87363053928981 2.60842449052612 -4.27745288087968<br>H 2.78548100897205 2.31219583192159 -3.22782635404177<br>H 3.77316414692325 2.14960972538827 -4.69674279078374<br>H 2.97838250280555 3.68951415723334 -4.32694972769002	
69	Au 0.64693610639198 -4.30794044928017 -1.78794370560207<br>P 0.84669932124268 -3.38268320352054 0.29804862779663<br>O -1.09968260018165 -3.87220107017920 -4.93356647378043<br>C 0.14250922747187 -3.81891754709750 -4.43169157799102<br>C 0.53529677276086 -5.02192527775997 -3.84204435274354<br>C -0.61214062235132 -5.89265527111506 -4.06906295861061<br>C -0.89118509223830 -7.22598866971004 -3.78215458798662<br>H -0.16285939531751 -7.84739246797913 -3.27719154893871<br>C -2.12441909942012 -7.73446723620842 -4.15909563559335<br>H -2.36186175861458 -8.76882745085236 -3.94802655870602<br>C -3.07187788980217 -6.93841709032679 -4.81018154172334<br>H -4.02481517687690 -7.36711283914772 -5.09021199339740<br>C -2.81221237109277 -5.60809125688480 -5.11022115280848<br>H -3.53614064903269 -4.98606828387972 -5.61938417548038<br>C -1.57406483303092 -5.13375629764391 -4.72759559073229<br>C 0.85548166895788 -2.56342185073477 -4.58113474855104<br>C 2.23840167997428 -2.52634155577160 -4.40095597783118<br>H 2.78362009964921 -3.44125950491327 -4.20149019688450<br>C 2.93672725570941 -1.33444236996918 -4.47325750746186<br>H 4.00821802185507 -1.35287205275304 -4.33016404424930<br>C 2.25436896941779 -0.14344322256027 -4.72819796276035<br>C 0.87143054532357 -0.17551969950765 -4.92952111984445<br>H 0.34292110989453 0.74692368025413 -5.13295039387269<br>C 0.18449423970171 -1.36810737054974 -4.86134590301545<br>H -0.88726018391273 -1.35947005908624 -5.00805802421716<br>C 0.75169662075759 -1.57060683474276 0.19416878256322<br>C 1.38163805775266 -0.92734802096920 -0.87161627995054<br>H 1.90114290947580 -1.49718541801424 -1.63367625660988<br>C 1.34543701687598 0.45450234563396 -0.96859910152560<br>H 1.83229866596340 0.94573089757959 -1.80089779547641<br>C 0.67590916191090 1.20205197765492 -0.00807901951002<br>H 0.64183985983675 2.28060591825888 -0.08887920965605<br>C 0.04476898907383 0.56522670407748 1.05073422126362	**TS*anti* (1$_{\mathbf{OMe}}$ and 2$_{\mathbf{OMe}}$)**<br>$E_{\text{PBEh-3c/CPCM(Chloroform)}}$=<br>-2318.292996901402<br>$ZPE_{\text{PBEh-3c/CPCM(Chloroform)}}$=<br>0.64761920<br>$FEC_{\text{(298.15)PBEh-3c/CPCM(Chloroform)}}$=<br>0.57760887<br>$E_{\text{PW6B95-D3(BJ)/def2-TZVPP/CPCM(Chloroform)}}$=<br>-2326.244161710127<br>$E_{\text{B3LYP-D3(BJ)/def2-TZVPP/CPCM(Chloroform)}}$=<br>-2322.468647859359

	H -0.48045612617099 1.14421102350964 1.79874397380173<br>C 0.08121175628936 -0.81861033120494 1.15492222083425<br>H -0.41727838540470 -1.30365210251141 1.98434552355646<br>C 2.42872155649792 -3.74966726917246 1.10524761673584<br>C 3.10402627346532 -2.78883660307678 1.85363759069273<br>H 2.70825361126320 -1.78570015863812 1.94723827557523<br>C 4.29723673664301 -3.11463697521766 2.48263992988832<br>H 4.82031201901600 -2.36405523914441 3.06022190371347<br>C 4.81796043402550 -4.39602498173262 2.36931683166048<br>H 5.75024595061914 -4.64632460154546 2.85834812678233<br>C 4.14725577295122 -5.35546812489935 1.62204006376481<br>H 4.55423094908037 -6.35307784620774 1.52497307628418<br>C 2.95789916069867 -5.03395525586303 0.98711811202984<br>H 2.44758803474644 -5.78577567647499 0.39652954167633<br>C -0.44238898914981 -3.87812056024135 1.47407733213734<br>C -1.75962571957213 -3.95113365385540 1.02216845247518<br>H -1.99545273665588 -3.74181340431308 -0.01481808669361<br>C -2.77630955116178 -4.29374875946071 1.89882020273385<br>H -3.79648016149040 -4.34803099396918 1.54281153428066<br>C -2.48348959794266 -4.57568740462128 3.22741583211364<br>H -3.27739755645908 -4.85000453781281 3.90960681541427<br>C -1.17294712542252 -4.51188587523996 3.67765741384921<br>H -0.94106017388462 -4.73500060080438 4.71060666027304<br>C -0.15134171596783 -4.16201805723981 2.80512853563060<br>H 0.86706923627525 -4.11527279250840 3.16839536638810<br>C 2.75418098360815 -7.04363541232368 -5.85134001933199<br>C 3.31977487787579 -7.75994632796442 -6.89473913239103<br>C 4.68424633444016 -8.01815174524627 -6.92381871787409<br>C 5.47387053424799 -7.55234677028087 -5.87956214143423<br>C 4.91579328195866 -6.83140819487910 -4.83613470451911<br>C 3.53137610866892 -6.55121633438268 -4.77693426379594<br>H 1.68558819232878 -6.85869660262067 -5.86015019328614<br>H 2.69023982683116 -8.11572690826343 -7.70317655572156<br>H 6.54132205264389 -7.74447391821855 -5.88918233371350<br>H 5.55191190554177 -6.46995696925633 -4.03596591001641<br>O 3.02218312399145 -5.87570590950789 -3.78835345743472<br>H 1.69727268511919 -5.43881567355224 -3.85435058576116<br>O 5.24576829767205 -8.71051140748865 -7.97040058789498<br>C 5.26032950848871 -10.10999214643084 -7.78564823719431<br>H 5.84082187303695 -10.40079341838215 -6.90362018930097<br>H 4.25061406868924 -10.51997255525239 -7.67721741761806<br>H 5.72205473011647 -10.55579418831135 -8.66654945081909<br>O 2.83458691169262 1.06181277508760 -4.79210903422453<br>C 4.23210978837931 1.15727086023498 -4.59805097692472<br>H 4.52955928977363 0.81036022897974 -3.60513307507051<br>H 4.78756599922819 0.59358404055287 -5.35164754839107<br>H 4.48388210125170 2.21044868945059 -4.69179265299275	
70	Au 2.07119157836880 -5.71608480211848 -1.93222141554718<br>P 1.55489296129623 -4.40209129721451 -0.18113431467349<br>O -1.99888058474971 -2.95744959991527 -3.48901084157621<br>C -1.00645005649570 -3.73178687541613 -4.00934714786963<br>C -1.41025305987711 -5.02477091786878 -4.12141653014794<br>C -2.75736019519063 -5.07044389429214 -3.63826432993814<br>C -3.72357998633425 -6.06378680251399 -3.47033245319523<br>H -3.51955836275429 -7.09012600859301 -3.74653290297766<br>C -4.95064813812038 -5.70231067185927 -2.94035254822749<br>H -5.71478499119357 -6.45602783852589 -2.80246336643957<br>C -5.22614212537946 -4.37816478223220 -2.57735712316417<br>H -6.19583669875319 -4.13083763423373 -2.16626366469906<br>C -4.27928038624484 -3.37629894399587 -2.73241594572023<br>H -4.48340425306831 -2.35193135801789 -2.45040606979862<br>C -3.06181478095887 -3.75923465079470 -3.26259216404442<br>C 0.24406451632702 -3.06936020368715 -4.32741135085411<br>C 1.30806159410066 -3.80075733229198 -4.85131395090400<br>H 1.19991118802146 -4.86003716572182 -5.04288852119117<br>C 2.52233531381832 -3.20559409785107 -5.14849398249232<br>H 3.31575856140611 -3.82434506941251 -5.54614274087192<br>C 2.69286498089304 -1.84010073239070 -4.92886906784975<br>C 1.62868621276687 -1.09261594966491 -4.42226275593305<br>H 1.75894992955104 -0.03205145269512 -4.25149525356904<br>C 0.42705969041373 -1.69775081958137 -4.12124485464892<br>H -0.37021519333397 -1.09000810969580 -3.71565711036531<br>C 1.74315507573773 -2.63772915183608 -0.56942705938699<br>C 2.92978266999367 -2.22174533923449 -1.17362152750374<br>H 3.69264965008015 -2.94480530150023 -1.43901404579868	**PC$_{anti}$ (1$_{OMe}$ and 2$_{OMe}$)**<br>E$_{PBEh-3c/CPCM(Chloroform)}$=<br>-2318.350370805303<br>ZPE$_{PBEh-3c/CPCM(Chloroform)}$=<br>0.65332233<br>FEC$_{(298.15)PBEh-3c/CPCM(Chloroform)}$=<br>0.58444929<br>E$_{PW6B95-D3(BJ)/def2-TZVPP/CPCM(Chloroform)}$=<br>-2326.301787287034

	C 3.14144173950739 -0.87940586390675 -1.43742765240672<br>H 4.06333938478190 -0.56363511198412 -1.90737899993326<br>C 2.16635830773196 0.05526170092465 -1.11298662902555<br>H 2.33093758688096 1.10384723581866 -1.32411042684826<br>C 0.97716846572185 -0.35718988454459 -0.53192228973048<br>H 0.21094790976788 0.36671569392791 -0.28757339129055<br>C 0.76295486328223 -1.70181894056847 -0.25739096409279<br>H -0.16731694169854 -2.01031533081598 0.20131946471123<br>C 2.63788978511638 -4.68306477656431 1.25280086388893<br>C 3.13967026639344 -3.63094314278446 2.01398417033548<br>H 2.91735726427826 -2.60532970537741 1.74925314197226<br>C 3.93392768543322 -3.89277228192508 3.12216469187813<br>H 4.32303634326760 -3.07026085843104 3.70763998476325<br>C 4.22726128920787 -5.20145303610143 3.47664585504273<br>H 4.84841692205349 -5.40282935514917 4.33956438195518<br>C 3.72685200079843 -6.25367592896360 2.72015904354031<br>H 3.95613844575862 -7.27582385828667 2.99069283534391<br>C 2.93845290907024 -5.99694625683958 1.60980599769830<br>H 2.55962972083593 -6.82453554094990 1.02143131082924<br>C -0.13000040965853 -4.60041755395166 0.46314502582121<br>C -1.15423550435528 -4.95143440941542 -0.41298135058069<br>H -0.93900356165260 -5.14285882420708 -1.45698127132666<br>C -2.45723658516991 -5.06751674571428 0.04810668190155<br>H -3.24679856364675 -5.34229511345214 -0.63908812000396<br>C -2.74188311131651 -4.83797869006590 1.38694540821802<br>H -3.75777341079163 -4.93341123029065 1.74755072751878<br>C -1.72303585470419 -4.49331533528405 2.26536356670568<br>H -1.94157343541170 -4.31899904012147 3.31062407234436<br>C -0.41934427843164 -4.37405217264636 1.80774060851079<br>H 0.36625779162572 -4.10844926184091 2.50354240807293<br>C 3.21988609667379 -6.78276211491855 -5.74600294721464<br>C 4.14392432931713 -6.42489136556556 -6.71096781494975<br>C 5.35669945661760 -5.83580632015188 -6.35593173392402<br>C 5.62220565060108 -5.62862260519384 -5.00729368750149<br>C 4.68753552294769 -5.98817780766784 -4.04103381655596<br>C 3.45753853364562 -6.56328467291351 -4.37845025373817<br>H 2.28222755633942 -7.23321749608972 -6.05032471293732<br>H 3.92460305728945 -6.59808009796114 -7.75797445356315<br>H 6.55302557489014 -5.18468674642586 -4.67839247009819<br>H 4.92846740436047 -5.81011511113270 -2.99819252115672<br>O 2.53748262243250 -6.91556249625744 -3.48944757163799<br>H -0.82534214804617 -5.84873209922823 -4.49768723703260<br>O 6.19619370169320 -5.50691420856709 -7.37200616498112<br>C 7.41862870046536 -4.89592849062471 -7.04417072340147<br>H 7.27991563755513 -3.94167171905226 -6.52460203879427<br>H 8.05079677587164 -5.53720176539822 -6.42173155546602<br>H 7.93680601615449 -4.70571187884169 -7.98183239397538<br>O 3.83350752260836 -1.17015269193703 -5.16773598097615<br>C 4.94743396090382 -1.89136534431022 -5.65166804526674<br>H 5.25960882359078 -2.67576285031773 -4.95683008540923<br>H 4.75002486498456 -2.34914909260832 -6.62494516782811<br>H 5.75698263103195 -1.17354135928293 -5.76044011844206	
71	Au 2.97362068743947 -4.19678177051232 -1.87578772716558<br>P 4.35228952198670 -3.49078313365642 -0.13598573682471<br>O 0.84973516352441 -6.04892269792458 -5.08416061099424<br>C 1.76256260742165 -6.02489032150322 -4.05694215373799<br>C 1.75451200862708 -4.84020985312947 -3.37802870637025<br>C 0.72728071772799 -4.06715262487270 -4.03966509095312<br>C 0.19837706406258 -2.78804948220038 -3.85701814520569<br>H 0.57021450037907 -2.14611570813280 -3.06717100491273<br>C -0.80842650366409 -2.35702785563783 -4.70437146153825<br>H -1.23024580471538 -1.36851896053205 -4.57626379563222<br>C -1.29375917018678 -3.17792125761266 -5.72951654633716<br>H -2.08123390062383 -2.81286862697428 -6.37570524839776<br>C -0.78468561338503 -4.45232987432269 -5.93312667789854<br>H -1.15592579401472 -5.09185646276921 -6.72331311273332<br>C 0.21965434662890 -4.85923913290362 -5.07306205381830<br>C 2.55307572481990 -7.24276750284505 -3.91848258174006<br>C 3.07276530983075 -7.63947345485372 -2.68064750900543<br>H 2.85748122281918 -7.06593140753884 -1.78941112735925<br>C 3.84246346673609 -8.77669290282927 -2.55597708788815<br>H 4.23097393670705 -9.06439732311436 -1.58697683156509<br>C 4.10902960840801 -9.57709794137898 -3.66903259790020<br>C 3.58223628750810 -9.21061061119447 -4.90440596165474<br>H 3.75967012834389 -9.80213343974072 -5.79215760032625	**RC$_{syn}$ (1$_{OMe}$ and 2$_{OMe}$)**<br>E$_{PBEh-3c/CPCM(Chloroform)}$=<br>-2318.342940725940<br>ZPE$_{PBEh-3c/CPCM(Chloroform)}$=<br>0.65306537<br>FEC$_{(298.15)PBEh-3c/CPCM(Chloroform)}$=<br>0.58376534<br>E$_{PW6B95-D3(BJ)/def2-TZVPP/CPCM(Chloroform)}$=<br>-2326.295560998022<br>E$_{B3LYP-D3(BJ)/def2-TZVPP/CPCM(Chloroform)}$=<br>-2322.516197132167

	C 2.81511971922200 -8.05935925271656 -5.01743746226551<br>H 2.43143814737979 -7.79681687313161 -5.99443024768130<br>C 5.81244789909571 -2.53976509370464 -0.65188453419053<br>C 6.48915270835192 -2.94109157450491 -1.80300328105706<br>H 6.12940161790171 -3.78347593415164 -2.38240838953967<br>C 7.62650509027062 -2.26385896438814 -2.21425787217468<br>H 8.14690962370582 -2.58183428993577 -3.10795251448315<br>C 8.08917646390583 -1.17541498653589 -1.48585530315582<br>H 8.97305047191532 -0.64271935535938 -1.81153789809537<br>C 7.41392720022981 -0.76784354240246 -0.34418000281199<br>H 7.76947878335349 0.08196339853677 0.22346028218131<br>C 6.27899211168419 -1.44813982435974 0.07535140240896<br>H 5.76119985711616 -1.12176790446092 0.96814860887519<br>C 5.00632895681230 -4.88519193411198 0.82960586804603<br>C 6.31716312368751 -4.91785391611060 1.29696937630240<br>H 6.99903133008777 -4.10596347688903 1.07887988636232<br>C 6.75990519460120 -5.99942566677735 2.04633838152748<br>H 7.78109082904082 -6.02108667890050 2.40348723330377<br>C 5.89755445883565 -7.04759707635375 2.33574041534988<br>H 6.24617316527454 -7.88998828728771 2.91886556514163<br>C 4.58861917778508 -7.01703307393812 1.87182063844711<br>H 3.91080068773622 -7.83152822673301 2.09131763748653<br>C 4.14445305862227 -5.94324715672947 1.11628704011035<br>H 3.12292148138306 -5.93464038062105 0.75459502272049<br>C 3.49649739170344 -2.43599555309516 1.07107475757390<br>C 2.62453541396327 -1.45974410231132 0.59077095516737<br>H 2.46837100373299 -1.34265067692056 -0.47547050321647<br>C 1.94503616045739 -0.63714935379620 1.47517529335396<br>H 1.26839702893068 0.11723574049946 1.09594344895812<br>C 2.12556380346563 -0.78919236242206 2.84463413204119<br>H 1.58728565223233 -0.15307801206486 3.53505341455437<br>C 2.98951841320535 -1.76196327643521 3.32566924154005<br>H 3.12747874979577 -1.88793139203588 4.39128263603745<br>C 3.67587805985658 -2.58417843517403 2.44250178041729<br>H 4.34045365785120 -3.34513028069158 2.83051474651219<br>C 0.37181752757503 -4.81553283412396 0.45298293967387<br>C 0.68786116157454 -4.44453589348357 1.74780971956857<br>C 0.94469780410121 -5.40770555500562 2.72174600127898<br>C 0.87151916513087 -6.75204987562757 2.37173910592953<br>C 0.56621565854658 -7.12186511781581 1.06627884196622<br>C 0.31548838578644 -6.16300789043192 0.09437259241677<br>H 0.15824619373340 -4.04628933464628 -0.28057788679941<br>H 0.73936110152970 -3.39473161766308 2.00895472572760<br>H 1.05972881264502 -7.53443566059894 3.09466330826971<br>H 0.52488559680334 -8.17180505517561 0.80409087177640<br>O 0.00660256452229 -6.57981541227052 -1.15445265831530<br>H 0.18756400617179 -5.88277620967898 -1.79907379448347<br>O 4.86560128423582 -10.66819262210589 -3.45384328327112<br>C 5.15020117314915 -11.51728248920468 -4.54418445402889<br>H 5.76053403357556 -12.32821118821870 -4.15404068685936<br>H 4.24215410251936 -11.94122682074880 -4.98181930692101<br>H 5.71121852064704 -11.00291341368594 -5.32940211492319<br>O 1.25386753436765 -4.95167735545293 3.95785632353099<br>C 1.61194462624343 -5.89285197789904 4.94132340315235<br>H 0.79275839549747 -6.57871019907206 5.17787091563723<br>H 2.48593496694228 -6.48266909582900 4.64719618790402<br>H 1.85942059291536 -5.32887833565318 5.83801485226947sq	
72	Au 2.40195745143224 -4.54380880061278 -1.49889610908407<br>P 4.05456196032170 -3.69242553583620 -0.14959070213449<br>O 1.73765636025492 -5.47066570378892 -4.99824657316337<br>C 1.69329860550058 -6.01881546254037 -3.77061155100502<br>C 0.90600289321206 -5.28765198271191 -2.88229598305476<br>C 0.40466735282977 -4.19599244134617 -3.71073784116331<br>C -0.44709228221503 -3.11848055479697 -3.48194966146645<br>H -0.89387681004214 -2.96467701705609 -2.50775240193904<br>C -0.70514339828889 -2.24949676091667 -4.53049250851715<br>H -1.36581415104581 -1.40664215080508 -4.37512053413701<br>C -0.12900383661032 -2.43913322117949 -5.79055927228906<br>H -0.35108721255325 -1.74203259477664 -6.58750629807775<br>C 0.72052944221579 -3.50811838816955 -6.04127244975386<br>H 1.16732172049017 -3.66487414892061 -7.01401968275193<br>C 0.95321694813281 -4.35843028421531 -4.97901051028117<br>C 2.42031374308439 -7.26010090792603 -3.58761555155121<br>C 2.16910318471820 -8.05285086981849 -2.45947291658783<br>H 1.40032305468731 -7.76167342415751 -1.75552780909923	**TS*syn* (1OMe and 2OMe)**<br>$E_{PBEh\text{-}3c/CPCM(Chloroform)}$=<br>-2318.304451725696<br>$ZPE_{PBEh\text{-}3c/CPCM(Chloroform)}$=<br>0.64797354<br>$FEC_{(298.15)PBEh\text{-}3c/CPCM(Chloroform)}$=<br>0.57962400<br>$E_{PW6B95\text{-}D3(BJ)/def2\text{-}TZVPP/CPCM(Chloroform)}$=<br>-2326.259268243329<br>$E_{B3LYP\text{-}D3(BJ)/def2\text{-}TZVPP/CPCM(Chloroform)}$=<br>-2322.484304019584

	C 2.86620437363046 -9.21949369592305 -2.24773832817911	
	H 2.66079563066354 -9.82545757102330 -1.37446998656760	
	C 3.83871588138009 -9.63889954266531 -3.16279602400164	
	C 4.08651095449136 -8.86690862235475 -4.29668945863709	
	H 4.82606174521293 -9.16045661890737 -5.02865458988019	
	C 3.37963225718674 -7.69226695096884 -4.50092255260661	
	H 3.59958962613276 -7.10800626037112 -5.38445786860539	
	C 5.32722590022192 -2.75416960897253 -1.04501835189185	
	C 5.79521444213230 -3.26064023438952 -2.25686987150956	
	H 5.37923932255827 -4.17512162001024 -2.66402671936977	
	C 6.79472301157272 -2.59573963735647 -2.94902427790277	
	H 7.15358503268583 -2.99438064641036 -3.88863924819260	
	C 7.32619866493622 -1.41657190167278 -2.44175766183667	
	H 8.10165733200032 -0.89418860291270 -2.98654387876662	
	C 6.85853475940860 -0.90636168829064 -1.23944411176816	
	H 7.26836536989070 0.01299193040779 -0.84252681856478	
	C 5.86227180667275 -1.57285659340413 -0.53890688179242	
	H 5.50657852485619 -1.16636121028850 0.39909604988453	
	C 4.96174054937558 -4.97750439875083 0.75490150450016	
	C 6.29912351153480 -4.80983983592001 1.10922620521322	
	H 6.83445778990439 -3.91157998475039 0.82875188151981	
	C 6.95646781513892 -5.80101863710949 1.82268877892018	
	H 7.99550299426929 -5.66699866868615 2.09313820023130	
	C 6.28495311819796 -6.96146866862303 2.18496788355227	
	H 6.80192664699286 -7.73511087946754 2.73758731681215	
	C 4.95344065753602 -7.13138073816152 1.83188445773610	
	H 4.42653802763906 -8.03631640714688 2.10494527346078	
	C 4.29197688098365 -6.14445515879671 1.11614652570616	
	H 3.25325676929199 -6.29190996407580 0.84574314885986	
	C 3.40252180784961 -2.55111976784166 1.10002732826126	
	C 2.53741710715285 -1.54226103211892 0.67595653723520	
	H 2.27819744678889 -1.44800813053695 -0.37255300677162	
	C 1.99718074610796 -0.65924334099084 1.59537118902984	
	H 1.32655759526551 0.12137948184959 1.26137611996056	
	C 2.30769736056871 -0.78460243536739 2.94490307549801	
	H 1.87737193225125 -0.10046978038568 3.66451232827569	
	C 3.16176303321892 -1.79087628412445 3.36868930156683	
	H 3.39892077631183 -1.89655514445979 4.41894038083597	
	C 3.71161493721892 -2.67444962766351 2.44903070802168	
	H 4.36984250219343 -3.46072722554003 2.79381263986472	
	C 0.04730414080922 -4.76511156469116 0.56212906787398	
	C 0.61195504717069 -4.47529124618211 1.79564353525100	
	C 1.08377533573335 -5.49143601327964 2.61968759465146	
	C 0.97766532398416 -6.80948760102314 2.18008189565186	
	C 0.42242136517631 -7.09634137733168 0.94009007529384	
	C -0.08799514372904 -6.09396126965837 0.09058971004712	
	H -0.36108494753012 -3.95376727557887 -0.03173614982559	
	H 0.68774334556699 -3.44564178708099 2.12592596167830	
	H 1.33333029454102 -7.63285454102033 2.78693212167182	
	H 0.34875029242228 -8.13137328764246 0.62488184457062	
	O -0.62229974128146 -6.37092342360782 -1.06685157189571	
	H 0.16856572795637 -5.82350836376468 -2.01593215988724	
	O 4.47003353801970 -10.78404431519738 -2.87198776619775	
	C 5.45688517163483 -11.26353952837211 -3.76217470037085	
	H 5.83040915617696 -12.19150737519306 -3.33681211272219	
	H 5.04588312849518 -11.47114016064362 -4.75352066710237	
	H 6.28961020070025 -10.56274484440945 -3.86412272559304	
	O 1.62726643378659 -5.11548834135386 3.80920490098892	
	C 2.13332106206690 -6.12150337083497 4.64818769237360	
	H 1.36017433665846 -6.82911861386535 4.96536195362475	
	H 2.94397254802567 -6.68695359664905 4.17620747301869	
	H 2.52906884206337 -5.62494192786042 5.53214463285395	
73	Au 1.69809070602619 -5.03153955563923 0.42237196880715	**PC*syn* (1$_{OMe}$ and 2$_{OMe}$)**
	P 3.76036942382856 -4.01870112348578 0.44019838356128	$E_{PBEh\text{-}3c/CPCM(Chloroform)}$=
	O 2.54240934562629 -4.15912840650514 -4.11674334920813	-2318.326546474504
	C 2.07876732702771 -5.36096431824626 -3.66911147097917	
	C 0.98340995935169 -5.20128669967882 -2.88260742709381	$ZPE_{PBEh\text{-}3c/CPCM(Chloroform)}$=
	C 0.73135618793582 -3.79184233496686 -2.83202348249532	0.65246075
	C -0.22483145838367 -2.97190609542934 -2.22979215053716	
	H -1.01110814085433 -3.39340880652437 -1.61662208501134	$FEC_{(298.15)PBEh\text{-}3c/CPCM(Chloroform)}$=
	C -0.14540335829291 -1.60509646543450 -2.44013435154069	0.58317491
	H -0.87852142995514 -0.95194198856639 -1.98508431121623	
	C 0.86715584149297 -1.04815350888265 -3.23058989601608	$E_{PW6B95\text{-}D3(BJ)/def2\text{-}TZVPP/CPCM(Chloroform)}$=
	H 0.90062463091578 0.02381956141955 -3.37392332997830	
	C 1.83200428913429 -1.84345154411278 -3.83223122044037	

	H 2.61819850268595 -1.41929954790043 -4.44270412878349<br>C 1.72992325214356 -3.20493351047918 -3.61533994499463<br>C 2.79352942211652 -6.54513420138026 -4.10576691088365<br>C 2.60040192174096 -7.76314806073259 -3.44413503252906<br>H 1.91392961948948 -7.82772011991136 -2.60813321381478<br>C 3.28240719560575 -8.89579770752985 -3.83247608019660<br>H 3.12640216771570 -9.83024222738172 -3.30831209981544<br>C 4.18275889001243 -8.84987534576247 -4.90140483995605<br>C 4.38005563044340 -7.64454236059577 -5.57032407166593<br>H 5.06435468851946 -7.56652122742562 -6.40400222639998<br>C 3.69071868814709 -6.50934113847042 -5.16873184272023<br>H 3.86850950497628 -5.58638452080933 -5.70420359277361<br>C 4.76441913266094 -4.37272970720176 -1.02763610098450<br>C 4.87830174694129 -5.70212096164872 -1.43308901449256<br>H 4.34780994546049 -6.48449493653463 -0.90246422193686<br>C 5.66588663460990 -6.03025788697149 -2.52288402848001<br>H 5.74254471855314 -7.06244712707291 -2.83801761658273<br>C 6.33124143489374 -5.03271389964040 -3.22390799561248<br>H 6.93618928660683 -5.28942519673380 -4.08372878255910<br>C 6.20811178661644 -3.70798055165338 -2.83220634195553<br>H 6.71962793801996 -2.92836682977624 -3.38123832314411<br>C 5.42873809673369 -3.37410950130035 -1.73217264026586<br>H 5.34410761148237 -2.33782289042449 -1.43201616796546<br>C 4.76924187918353 -4.57016078470421 1.84805102525547<br>C 6.15143248686222 -4.70519802569820 1.74064837969069<br>H 6.65049613281664 -4.50827419538566 0.80034473698644<br>C 6.89855456807951 -5.09858250955721 2.84187693563412<br>H 7.97145756235855 -5.20463230001242 2.75076985313557<br>C 6.27222428979635 -5.35704583037855 4.05308284040862<br>H 6.85665547626680 -5.66729099427795 4.90935567036456<br>C 4.89441212490784 -5.22357844527351 4.16385441513955<br>H 4.40085162254919 -5.42944162383768 5.10452197323710<br>C 4.14331894413314 -4.83515536616752 3.06524978378000<br>H 3.06775713849344 -4.74353191098530 3.16264799948677<br>C 3.69073434391386 -2.20993689056642 0.56956211993362<br>C 2.73723351280121 -1.52997600664697 -0.18593615208425<br>H 2.04007937045297 -2.07633058812889 -0.80992728212141<br>C 2.67469526738559 -0.14618430500667 -0.14262717207181<br>H 1.93305946009387 0.37310964930346 -0.73493345691595<br>C 3.55381185113707 0.56527932674586 0.66333703641902<br>H 3.49922298473325 1.64538342690653 0.70259378189357<br>C 4.49926490254783 -0.10966887566385 1.42283404145006<br>H 5.18330805081996 0.44118967438234 2.05475744455268<br>C 4.57196362312241 -1.49479427656062 1.37628304189490<br>H 5.31400960417970 -2.00914481056831 1.97294988855706<br>C -0.27912510200022 -5.99390323590364 0.60649472885150<br>C -0.44373229459464 -5.65216077199494 1.99564916349662<br>C 0.01839384975684 -6.46681202349284 2.99276412204435<br>C 0.64231843385015 -7.68644221642314 2.62726785259443<br>C 0.74396147478942 -8.08422542914107 1.32361481578391<br>C 0.14990254602533 -7.33827771009281 0.23067518874602<br>H -0.93816066806363 -5.48570471639482 -0.09781417829825<br>H -0.93627267101924 -4.71959597837220 2.24854661096765<br>H 1.05133618382758 -8.33934252906718 3.38866038235255<br>H 1.18586015349329 -9.04731362725417 1.09596262568456<br>O 0.03066102671309 -7.80937766819056 -0.91416141159705<br>H 0.40781298171429 -5.99169853779588 -2.42318136828210<br>O 4.80247165324618 -10.00213766018445 -5.20806851144050<br>C 5.72433322609437 -10.01041580439117 -6.27645418368033<br>H 6.10332986864351 -11.02678544450511 -6.35024576561909<br>H 5.25351231767036 -9.74169024819032 -7.22603644331605<br>H 6.56513805413104 -9.33443709082722 -6.09730834493213<br>O -0.13309478997634 -6.04840755579087 4.28048010167704<br>C 0.42249017826470 -6.83159919128325 5.30697684287369<br>H -0.02082624874465 -7.83113138771157 5.36028368964219<br>H 1.50811701481044 -6.93993679083768 5.20868607905762<br>H 0.21268150716600 -6.31514545643898 6.24155194636631	**-2326.281361975545**
74	Au 1.92026328152884 -5.17072447783857 -2.19968458412633<br>P 1.49101389220302 -4.12433151433784 -0.25443287892848<br>C 1.52447407381179 -2.31367583464508 -0.39078878901659<br>C 2.54137558584754 -1.72204626373165 -1.13914462327084<br>H 3.27172927387728 -2.33546229178213 -1.65370529755572<br>C 2.62281151169198 -0.34173213545256 -1.23209892009018<br>H 3.41451710522174 0.11099592038771 -1.81408957447632<br>C 1.68568928991216 0.45546921371171 -0.58701959348959	**4-OMe-PhOAuPPh$_3$**<br>$E_{PBEh-3c/CPCM(Chloroform)}$=<br>-1590.648646954402<br>$ZPE_{PBEh-3c/CPCM(Chloroform)}$=<br>0.41402880

	H 1.74746852141144 1.53306013969008 -0.66418716076866<br>C 0.66834240890589 -0.13030349079709 0.15220664999306<br>H -0.06600057098235 0.48742805048690 0.65196392832148<br>C 0.58650349658133 -1.51243282056956 0.25404182832965<br>H -0.21203313533653 -1.95744341996843 0.83335229815484<br>C 2.68155902778159 -4.52369157526380 1.05871007950916<br>C 3.12728241312934 -3.55920822189047 1.95883133342388<br>H 2.79702123602393 -2.53203449545989 1.87328342747457<br>C 4.00559045233426 -3.91094947533878 2.97418997412519<br>H 4.35081120862760 -3.15626852039784 3.66838019138698<br>C 4.43917346031624 -5.22320987644947 3.09781457454810<br>H 5.12540884719936 -5.49436153179738 3.88936410514738<br>C 3.99730995373800 -6.18746718336216 2.20122940851451<br>H 4.33722920014039 -7.21065961619927 2.29064302709084<br>C 3.12459811191485 -5.83982563346886 1.18207519781541<br>H 2.79120098228886 -6.59810594300533 0.48335290906821<br>C -0.13952063318505 -4.52655330756596 0.43785264117056<br>C -1.23613540255107 -4.56883418635878 -0.42269678075605<br>H -1.10695820498096 -4.39181859954390 -1.48405797392008<br>C -2.50109019377923 -4.83775211853817 0.07523853874576<br>H -3.34738787905570 -4.86862045292883 -0.59793423253139<br>C -2.67802487404002 -5.07538390490462 1.43247405881616<br>H -3.66513049500667 -5.29240121255540 1.81918701917396<br>C -1.58851744509882 -5.04025497185599 2.29034641666856<br>H -1.72187993203264 -5.22814970730070 3.34746313475978<br>C -0.32051590703730 -4.76420332349612 1.79733324188486<br>H 0.52069844783622 -4.73946321851471 2.47767039645223<br>C 3.29266730024931 -6.86699110253158 -5.88504267957924<br>C 4.40816113055184 -6.93862191701696 -6.69645431511215<br>C 5.60181143534060 -6.31777448386373 -6.32939748750825<br>C 5.64222913713219 -5.62714819494090 -5.12588287668915<br>C 4.51491105602427 -5.55778079921071 -4.31017304028954<br>C 3.30761305073765 -6.16962634594001 -4.66234328793146<br>H 2.37856841816215 -7.35955388482797 -6.19502928904545<br>H 4.35925573206011 -7.48405879569269 -7.63160775184141<br>H 6.54378361326367 -5.12997684927053 -4.79183529904210<br>H 4.59440259791428 -5.00981005619910 -3.37679093302675<br>O 2.20050509222905 -6.14224777631643 -3.93790515745688<br>O 6.64436031897947 -6.44046091697328 -7.19323543338299<br>C 7.86032027655264 -5.82842534475965 -6.84573949552234<br>H 7.76451467489095 -4.74252636788063 -6.74285358698949<br>H 8.27516780844030 -6.22650358484111 -5.91386644364949<br>H 8.56029684523413 -6.03807198669164 -7.65214097157819	FEC$_{(298.15)\text{PBEh-3c/CPCM(Chloroform)}}$=<br>0.35862114<br>E$_{\text{PW6B95-D3(BJ)/def2-TZVPP/CPCM(Chloroform)}}$=<br>-1595.779234924484
75	C 0.71913820810607 -3.40684720083652 -0.44737894010676<br>C 0.75916230651965 -4.42972628639284 0.48970211274124<br>C 0.84578634683140 -5.77501569333235 0.10522112328148<br>C 0.91700176567577 -6.03025232710135 -1.27394884215750<br>C 0.87754247167865 -5.00736412497469 -2.20660557417644<br>C 0.77482795619294 -3.68060155722230 -1.80705582012603<br>H 0.64967422347057 -2.38191295667044 -0.09814193707367<br>H 0.72063085756544 -4.15310112405164 1.53368326893733<br>H 1.00381578342850 -7.04173831351916 -1.64472549304357<br>H 0.93315364330105 -5.24740059105035 -3.26141539449650<br>O 0.74260045303473 -2.71943804079449 -2.76404680343347<br>H 0.67026948929497 -1.85150287500693 -2.35494525736102<br>N 0.84930171032011 -6.80111029889734 1.03526181508808<br>C 1.26594459489041 -8.11402020339130 0.60601740603923<br>H 1.22076941291769 -8.79822424583683 1.45014029488963<br>H 0.59892921028675 -8.51292484137209 -0.16028362978407<br>H 2.28749908713908 -8.13819850657422 0.20602523092917<br>C 1.05200938051424 -6.46588384503048 2.42312969856100<br>H 0.25845058410407 -5.81441102479269 2.79370015038137<br>H 1.01920519921162 -7.37434414035298 3.02010192995018<br>H 2.01093502451622 -5.96849206079869 2.61736359596030	**4-(dimethylamino)phenol (2$_{\text{NMe2}}$)**<br>E$_{\text{PBEh-3c/CPCM(Chloroform)}}$=<br>-440.482536480674<br>ZPE$_{\text{PBEh-3c/CPCM(Chloroform)}}$=<br>0.18312876<br>FEC$_{(298.15)\text{PBEh-3c/CPCM(Chloroform)}}$=<br>0.14910910<br>E$_{\text{PW6B95-D3(BJ)/def2-TZVPP/CPCM(Chloroform)}}$=<br>-442.179581914908<br>E$_{\text{B3LYP-D3(BJ)/def2-TZVPP/CPCM(Chloroform)}}$=<br>-441.384643030792
76	Au 0.12816833220487 -4.43673356129696 -1.93894013816625<br>P 1.08039621779634 -3.69641804723427 0.05403067455399<br>O -1.04245245351071 -5.13580011306101 -5.97497610569525<br>C -0.33832995542318 -4.51762805881478 -4.96629059295672<br>C -0.63083767690143 -5.02009032324980 -3.73525719504655<br>C -1.58758948310579 -6.06675167416928 -4.00550435280857<br>C -2.27761294491850 -6.98857688963454 -3.21464986649163<br>H -2.14374831060786 -6.99898488469084 -2.13930397520528<br>C -3.13785509864527 -7.88344216395383 -3.83000617748866<br>H -3.67847243939545 -8.60494296227994 -3.23109326367755<br>C -3.32431619521597 -7.87120209548882 -5.21726199839028	**RC$_{anti}$ (1$_{\text{OMe}}$ and 2$_{\text{NMe2}}$)**<br>E$_{\text{PBEh-3c/CPCM(Chloroform)}}$=<br>-2337.738978587532<br>ZPE$_{\text{PBEh-3c/CPCM(Chloroform)}}$=<br>0.69474820<br>FEC$_{(298.15)\text{PBEh-3c/CPCM(Chloroform)}}$=<br>0.62336698

	H -4.00345526408247 -8.58215589548956 -5.66929125335934<br>C -2.65353029816208 -6.96430366401852 -6.02571696752088<br>H -2.79367172806153 -6.95081551050970 -7.09886294287687<br>C -1.79703761415450 -6.08387714756612 -5.38910897814338<br>C 0.58118428959834 -3.47523485419855 -5.40610553588186<br>C 1.81749753872358 -3.31370379481617 -4.78778142987364<br>H 2.12707427483548 -4.00824270124163 -4.01813189683873<br>C 2.69607975210886 -2.30972623191081 -5.16630308428280<br>H 3.64720003372942 -2.23603127987713 -4.65606691715749<br>C 2.36034274763690 -1.45109007777140 -6.20937289677322<br>C 1.13806950691164 -1.62213645858867 -6.86288347075784<br>H 0.87474971847625 -0.96142927046016 -7.67935483726039<br>C 0.26720019357452 -2.61799015342705 -6.46650370526124<br>H -0.67906348512670 -2.71595461465494 -6.98302122430992<br>C 1.74595904122152 -2.01075124250804 -0.11962572650896<br>C 2.49316104484342 -1.71942154608706 -1.26117243793633<br>H 2.65959658944697 -2.47976940570896 -2.01504327192269<br>C 3.02619536943606 -0.45371802018997 -1.44196676682077<br>H 3.59983369979275 -0.23607736859969 -2.33388960544592<br>C 2.81259166805371 0.53355260791105 -0.48787250926337<br>H 3.22277534516071 1.52447281783796 -0.63241214631473<br>C 2.06752259443222 0.24904833640894 0.64706663624420<br>H 1.89685089546098 1.01566756335896 1.39136912200148<br>C 1.53464959638806 -1.02000370027577 0.83389775620156<br>H 0.95388995298117 -1.22795147859864 1.72328668745759<br>C 2.49216273324634 -4.69030762378153 0.62237918619430<br>C 3.57843123397759 -4.11365891779806 1.27731218588792<br>H 3.61366357788799 -3.04436172896653 1.44379028147682<br>C 4.62682631100584 -4.90852351089366 1.71748147142972<br>H 5.46842903179690 -4.45384652995436 2.22325062955738<br>C 4.59696875073125 -6.28066928212719 1.50792879442077<br>H 5.41743829022855 -6.89840132329816 1.84899377735308<br>C 3.51792090074235 -6.85878181949552 0.85310817311891<br>H 3.49391070063438 -7.92622524289016 0.67819105316630<br>C 2.46982356412013 -6.06703491839637 0.40773882569011<br>H 1.64240475768495 -6.52621199890455 -0.11940472809983<br>C -0.05819101317740 -3.61538683269576 1.46851364816689<br>C -1.35619976325589 -3.15887608089152 1.24051969379211<br>H -1.67080462634540 -2.88541891227618 0.24001420204461<br>C -2.25223100913173 -3.05160676632061 2.29248938612733<br>H -3.25668360727958 -2.69444962340509 2.10768604962594<br>C -1.86334346517085 -3.41145966122853 3.57650573250413<br>H -2.56547635873416 -3.33468453312855 4.39636326299692<br>C -0.57607102941570 -3.87547491820682 3.80589685314021<br>H -0.27091666214412 -4.16130699887719 4.80388660990716<br>C 0.32707496314314 -3.97637726085808 2.75642417361042<br>H 1.32802989294098 -4.34003427419623 2.94926764588247<br>C 1.97085068190385 -6.95487165065258 -5.11609563761184<br>C 2.85785955406362 -6.31519631841682 -5.96495507422525<br>C 4.07669180473468 -5.79910806385938 -5.50183383980746<br>C 4.35089083559253 -5.96487740888904 -4.13664306799680<br>C 3.45757080053483 -6.60259621969326 -3.28721329063561<br>C 2.25468604149194 -7.10214069910979 -3.76491898380703<br>H 1.03888422750410 -7.33333614312880 -5.52229950490009<br>H 2.56897879454171 -6.20861843408292 -7.00064223865567<br>H 5.26604339883228 -5.58453467584766 -3.70506602626395<br>H 3.70226343010142 -6.70147594823538 -2.23633547582992<br>O 1.40256579317097 -7.70180612595375 -2.89309371734469<br>H 0.52806704766102 -7.77299209301582 -3.29268352220838<br>O 3.14570085206938 -0.45259541635146 -6.65565277685555<br>C 4.38695781963723 -0.24141185616402 -6.02120238552091<br>H 4.26945554490371 0.00325556415589 -4.96107912024067<br>H 5.04723684466183 -1.10962448591549 -6.10705146219808<br>H 4.85331583982856 0.60234799005487 -6.52422091221749<br>N 4.96253947011587 -5.15835619131319 -6.35524603985809<br>C 6.04737869698440 -4.40978200075292 -5.77212941394319<br>H 6.70533482944047 -5.05462915005113 -5.18694893339067<br>H 6.65259934250660 -3.97716452490368 -6.56577667582252<br>H 5.71418457606964 -3.59247629164679 -5.11815476360856<br>C 4.46217210255756 -4.69714908052442 -7.62892495081241<br>H 5.26068080697542 -4.18828822028946 -8.16422079130477<br>H 4.14229101874730 -5.53063039308247 -8.25669792439638<br>H 3.61839387347247 -4.00172485776344 -7.53650598535435	$E_{PW6B95\text{-}D3(BJ)/def2\text{-}TZVPP/CPCM(Chloroform)}$=<br>-2345.775916171854<br><br>$E_{B3LYP\text{-}D3(BJ)/def2\text{-}TZVPP/CPCM(Chloroform)}$=<br>-2341.941276427811
77	Au 0.58896607080184 -4.44106874879952 -1.66165223802857<br>P 0.76548198477221 -3.38816804910328 0.36523720938159	**TS*anti* (1_{OMe} and 2_{NMe2})**

	O -1.00340699380831 -4.11380740744588 -4.83809897344551<br>C 0.23446289464682 -4.11467800445864 -4.32383653590523<br>C 0.53832340838017 -5.30984977840096 -3.66600834591008<br>C -0.65877220708139 -6.11676451977473 -3.86870313031622<br>C -1.02743433925795 -7.41305397792481 -3.52051453805244<br>H -0.34726346809992 -8.05406009993668 -2.97472101167573<br>C -2.28736646415936 -7.85813399437368 -3.88932806256053<br>H -2.59450174333668 -8.86308268019261 -3.63133881957273<br>C -3.17370330454006 -7.03518091819977 -4.59094923955795<br>H -4.14985634079769 -7.41418805326729 -4.86250280894109<br>C -2.82419574340198 -5.74101403236372 -4.95183220999727<br>H -3.50039169819541 -5.09911326556364 -5.50037727884928<br>C -1.56140042104282 -5.33022390515271 -4.57676710589984<br>C 1.03303887463076 -2.92085143716218 -4.53307738969260<br>C 2.40705156954922 -2.95593611394112 -4.29194841650477<br>H 2.88044497343938 -3.88344592169652 -3.99111678313067<br>C 3.18374938416045 -1.81912477137941 -4.42304743624057<br>H 4.24477866975324 -1.89298254299894 -4.22894304104691<br>C 2.59188922610020 -0.61194749829854 -4.79986278973210<br>C 1.21915628282983 -0.57374001306356 -5.06133085193099<br>H 0.76125012141607 0.36072003570874 -5.35915051434987<br>C 0.45330003684858 -1.71214736471807 -4.93361821237594<br>H -0.60842508173861 -1.64848755397168 -5.13065193250602<br>C 0.75709048141539 -1.58449074250619 0.14368613938771<br>C 1.44348338560862 -1.03953826329275 -0.94187687144653<br>H 1.95478596721331 -1.67923061573629 -1.65232197483787<br>C 1.47521225524300 0.33362004122892 -1.12536518694395<br>H 2.00644976759098 0.74808383632131 -1.97221641399772<br>C 0.81738084595234 1.17022010804486 -0.23259140825660<br>H 0.83649014568352 2.24192179651695 -0.38106758285575<br>C 0.12996846902245 0.63121172207681 0.84540071741655<br>H -0.38626934282266 1.27977355198084 1.54070051876098<br>C 0.09851858020031 -0.74342995635120 1.03648812362044<br>H -0.44294461911582 -1.15198276267355 1.88007195439310<br>C 2.30559755210779 -3.77224612482602 1.24189149783042<br>C 3.00837640256024 -2.79471022383300 1.94170910267231<br>H 2.66090180230266 -1.76965747119318 1.95486219204720<br>C 4.16756705074044 -3.13243553869777 2.62568489446909<br>H 4.71234069917704 -2.36914787046801 3.16530090924306<br>C 4.62691374259953 -4.44192197651575 2.61530714693903<br>H 5.53294600382797 -4.70161266108986 3.14692067318190<br>C 3.92888492663635 -5.41811627009135 1.91628192485270<br>H 4.28850964891187 -6.43823599775499 1.89913055151204<br>C 2.77344845985808 -5.08538107605478 1.22696753406647<br>H 2.24181714809157 -5.85137723113628 0.67465046213066<br>C -0.57884147537610 -3.75112651857637 1.52746663135779<br>C -1.88420819500371 -3.79422460061789 1.03876653243905<br>H -2.07960048369461 -3.63977652077472 -0.01608681865742<br>C -2.94068843268411 -4.03689203011602 1.90154124548335<br>H -3.95139901687384 -4.06844570273754 1.51697809656290<br>C -2.70009175579263 -4.24824336120862 3.25353024072595<br>H -3.52535043147418 -4.44464589473755 3.92532092021870<br>C -1.40177145150216 -4.21409500332476 3.74088927417350<br>H -1.21078688592383 -4.38253031171272 4.79239894674969<br>C -0.34012122747091 -3.96425813454242 2.88194024522462<br>H 0.66842990120834 -3.93968361604708 3.27384354710939<br>C 2.80966497130273 -7.38621530852031 -5.62451674032145<br>C 3.37221959166056 -7.79734704307552 -6.82430472150943<br>C 4.70877692975512 -7.53015762276005 -7.14648390062623<br>C 5.43751910051552 -6.80339783965573 -6.19572789949275<br>C 4.87160860442501 -6.38562438335701 -5.00011698388516<br>C 3.53381035508766 -6.66325743708599 -4.65392005406539<br>H 1.77147218734289 -7.63475643728639 -5.42834534795535<br>H 2.73887464386591 -8.34265991851201 -7.51171456303223<br>H 6.47231045141507 -6.54336922989814 -6.37650886590107<br>H 5.48289112523720 -5.82368125419178 -4.30172746041144<br>O 3.00599888382189 -6.25720384709860 -3.53179250791419<br>H 1.65444504395123 -5.77168993850360 -3.61447999152416<br>O 3.25171915782151 0.54599616474722 -4.92917363671428<br>C 4.64352287772762 0.56983469193972 -4.67999435213556<br>H 4.87753549658687 0.28513972789539 -3.65098504417826<br>H 5.19087345784260 -0.08524176291683 -5.36214356220061<br>H 4.96709616899242 1.59462771230672 -4.84274402253842<br>N 5.28981871522721 -7.98647187438802 -8.34071628120298<br>C 6.52366198515193 -7.36136846766394 -8.74852788387883	$E_{PBEh-3c/CPCM(Chloroform)}$=<br>-2337.689757852278<br>$ZPE_{PBEh-3c/CPCM(Chloroform)}$=<br>0.69066427<br>$FEC_{(298.15)PBEh-3c/CPCM(Chloroform)}$=<br>0.61942334<br>$E_{PW6B95-D3(BJ)/def2-TZVPP/CPCM(Chloroform)}$=<br>-2345.728295669423<br>$E_{B3LYP-D3(BJ)/def2-TZVPP/CPCM(Chloroform)}$=<br>-2341.897149033470

	H 7.31546386986362 -7.53775993131000 -8.01908002664178<br>H 6.85657409464221 -7.80278243819744 -9.68639532827269<br>H 6.44351808488169 -6.27483134953681 -8.89573837308626<br>C 4.39071114284091 -8.32077149427633 -9.41848199747495<br>H 4.97010116371899 -8.63805724054984 -10.28386815264837<br>H 3.74408367798901 -9.15724204737428 -9.14999617591825<br>H 3.74475860324652 -7.48782488580344 -9.73001583620092	
78	Au 1.71095761901365 -6.02550079134760 -1.49020182744516<br>P 1.22634850232254 -4.51363683381801 0.10230318921018<br>O -0.91603537883487 -2.92636372585707 -3.74901298107099<br>C 0.08399768796856 -3.62245014910925 -4.35799515475156<br>C -0.13601045031190 -4.95962013658664 -4.28151437963226<br>C -1.36815554507965 -5.12409923789336 -3.57213775006151<br>C -2.15620091974641 -6.20859426985662 -3.18264309825602<br>H -1.85177005557962 -7.22343762817403 -3.40386952926019<br>C -3.33863720191502 -5.95153200506159 -2.50906567296233<br>H -3.96707902996088 -6.77689219409543 -2.20053141795576<br>C -3.73701981102912 -4.64255536876955 -2.21124602338128<br>H -4.66336552091470 -4.47767530669847 -1.67709851140060<br>C -2.96687186158930 -3.55102332005154 -2.58549760736729<br>H -3.26923010296366 -2.53706758482722 -2.35930771177838<br>C -1.79743574039939 -3.82861300609920 -3.26848056568865<br>C 1.15114000551849 -2.85412471381371 -4.97079102366256<br>C 2.36018944471275 -3.47132505954975 -5.28113991621106<br>H 2.51585452394468 -4.51731560590509 -5.04769203503093<br>C 3.39915844941735 -2.76975790280930 -5.86964440114833<br>H 4.32062336154111 -3.29624149374867 -6.07677074750090<br>C 3.24365302733988 -1.41744598699542 -6.16813032730832<br>C 2.03568578840335 -0.78743422337124 -5.85948489183919<br>H 1.90972433230988 0.26291821178998 -6.09016675273549<br>C 1.00900045093608 -1.49497563911444 -5.26797302463000<br>H 0.08594480613816 -0.97861624933196 -5.04053089705783<br>C 0.93921464405773 -2.85492488831920 -0.57731485531146<br>C 1.85774275679397 -2.35556446481035 -1.49981032460831<br>H 2.69058852648623 -2.96494736253417 -1.83130310848479<br>C 1.71214992504333 -1.07282004741290 -2.00055241528859<br>H 2.42909908892974 -0.69223922995610 -2.71525618831818<br>C 0.63880636100541 -0.28808203042335 -1.59912628614398<br>H 0.52070011851180 0.71098826914717 -1.99805485704958<br>C -0.28579202535938 -0.78776516717548 -0.69372011134299<br>H -1.12610896897633 -0.18079645210739 -0.38333129079523<br>C -0.13596589125177 -2.06780727345714 -0.17697375877697<br>H -0.85951810598248 -2.44407537541794 0.53434499939203<br>C 2.56864315095519 -4.30107611469207 1.30902133648895<br>C 2.90005018990832 -3.04878300397905 1.81951086153340<br>H 2.38001221169882 -2.16207316802040 1.48067281072215<br>C 3.90596280582350 -2.92990383946550 2.76842559312694<br>H 4.16150256604226 -1.95340660858100 3.15812756119749<br>C 4.58031651873337 -4.05738767926559 3.21484565951679<br>H 5.36533539749376 -3.96174851132698 3.95351956820992<br>C 4.25105758552031 -5.30836365733040 2.70913626096646<br>H 4.77729850408278 -6.18958374523347 3.05126559159009<br>C 3.25207620764662 -5.43094836757397 1.75608366781824<br>H 3.00847242457814 -6.41023910325336 1.36104743094267<br>C -0.24457445015359 -4.90130447446272 1.09174540003445<br>C -1.37541827082349 -5.38352493667762 0.43570674500402<br>H -1.36219530799873 -5.54586996687501 -0.63548688932126<br>C -2.52978064258877 -5.65728931823537 1.15195673345542<br>H -3.40357853938632 -6.02974516025332 0.63397046335762<br>C -2.55930471440341 -5.46068806139578 2.52644971464198<br>H -3.45880773037604 -5.68185529682854 3.08603402351937<br>C -1.43326616505376 -4.98429300583297 3.18298812019121<br>H -1.45171590117350 -4.83185716203327 4.25403451911284<br>C -0.27707620135443 -4.70132484127834 2.46928828010676<br>H 0.59372378077821 -4.32946949466387 2.99326260075650<br>C 2.56997013355681 -7.48776303689106 -5.22824069101489<br>C 3.34826892049807 -7.18745659896781 -6.33177433251428<br>C 4.53314436281810 -6.44958821422672 -6.22098414937414<br>C 4.90448254797598 -6.05466995504674 -4.93438982595070<br>C 4.13219618316043 -6.37029582107388 -3.82204306860933<br>C 2.93275528574097 -7.08198160428645 -3.93329021239478<br>H 1.65810268177722 -8.05849011526277 -5.36363886549845<br>H 3.02263113492307 -7.54740095962122 -7.29989779836968<br>H 5.80751036240400 -5.48099483899753 -4.77244142338026<br>H 4.47135421371966 -6.04057956364262 -2.84521963946695	**PC$_{anti}$ (1$_{OMe}$ and 2$_{NMe2}$)**<br>$E_{PBEh\text{-}3c/CPCM(Chloroform)}$=<br>-2337.747595316005<br>$ZPE_{PBEh\text{-}3c/CPCM(Chloroform)}$=<br>0.69525039<br>$FEC_{(298.15)PBEh\text{-}3c/CPCM(Chloroform)}$=<br>0.62567405<br>$E_{PW6B95\text{-}D3(BJ)/def2\text{-}TZVPP/CPCM(Chloroform)}$=<br>-2345.782756759301

	O 2.16129669444219 -7.39976548463058 -2.90085509369905<br>H 0.48206072056490 -5.73276047352500 -4.70919121909378<br>O 4.18944956198763 -0.65446698012508 -6.74208638024103<br>C 5.41237267914910 -1.25942974098195 -7.10500089412277<br>H 5.94902281173882 -1.65359224264867 -6.23745690303882<br>H 5.26995617961568 -2.07002532976668 -7.82561565265941<br>H 6.01710765619235 -0.48267663728355 -7.56658040086001<br>N 5.30878226945450 -6.17127725175028 -7.36253249266030<br>C 6.58466557188249 -5.54022668958220 -7.13953272510569<br>H 7.17862908138375 -6.10954391447815 -6.42438160288496<br>H 7.13815298236360 -5.51473071835937 -8.07824249482690<br>H 6.51266625318466 -4.50464378991677 -6.77127953216046<br>C 4.60457762070459 -5.59611524872781 -8.49110399662474<br>H 5.25157644547671 -5.61287893874115 -9.36861323708296<br>H 3.71383072704375 -6.16849207358238 -8.73885806678185<br>H 4.29603602074832 -4.55500768061526 -8.31814890051697	
79	Au -1.66445213354986 -8.92517375401446 1.37485055360038<br>P -1.10315260762321 -6.75036353621330 0.75650602982521<br>O -2.04297399639430 -13.06995080751908 2.32490069963732<br>C -1.34377325810859 -11.93601638023747 1.98628706052730<br>C -2.13644953379177 -10.82570043165930 1.94216521758062<br>C -3.44589633670625 -11.31873970248000 2.30605178240682<br>C -4.70184906784578 -10.72944546790306 2.46270256637213<br>H -4.83492957426563 -9.66622236824870 2.30121060117908<br>C -5.77159654751894 -11.52966852841950 2.82706678489734<br>H -6.75200237043379 -11.08891790165182 2.95377610315463<br>C -5.60999835302771 -12.90479155306146 3.03527713694159<br>H -6.46509666657780 -13.50400693195634 3.31935017388900<br>C -4.37330362959460 -13.51527324670480 2.88473152953081<br>H -4.24210331292767 -14.57759761872210 3.04423802332954<br>C -3.32093284335252 -12.69418731160554 2.52083237502288<br>C 0.07558767663307 -12.14980599210078 1.72801629901881<br>C 1.01617802402707 -11.15092907536413 1.96922199095307<br>H 0.70830200993445 -10.20764072319916 2.39949970391842<br>C 2.36354804596030 -11.33542330267654 1.69650405295559<br>H 3.04737386323553 -10.52379956483231 1.90509712975841<br>C 2.80855185492722 -12.55193780422053 1.18515303153348<br>C 1.88131662559270 -13.57127063150969 0.96003578200647<br>H 2.22079156077533 -14.52097959148600 0.56607314725322<br>C 0.54256826691805 -13.37216210137252 1.22913440088772<br>H -0.14962808966803 -14.17975935218981 1.03060308664774<br>O 4.09047931244732 -12.83268122041335 0.88977439865334<br>C -1.35542737521525 -6.36267625039107 -1.00079415095415<br>C -1.05276645151051 -7.34329902613275 -1.94454708752895<br>H -0.70359365524193 -8.31863208076804 -1.62570867890014<br>C -1.19799149235371 -7.07704010985563 -3.29720005289100<br>H -0.96046298669326 -7.84283113711829 -4.02366079483481<br>C -1.65643320133121 -5.83459582409089 -3.71583770457370<br>H -1.77657297123705 -5.62976135176885 -4.77161232708823<br>C -1.96612902181874 -4.85836591357048 -2.77944583465349<br>H -2.32691122820091 -3.89058608995164 -3.10204193815160<br>C -1.81484817073202 -5.11848907034860 -1.42444826924213<br>H -2.05845577935283 -4.34891747640249 -0.70320895017439<br>C 0.64523970851750 -6.37337532957733 1.08240576771608<br>C 1.42802366306709 -5.64177999268337 0.19366106349036<br>H 1.01223590003982 -5.28356730468603 -0.73929650459206<br>C 2.75444179698519 -5.36882731484459 0.49957261170226<br>H 3.35886153132754 -4.80275410775788 -0.19689264479941<br>C 3.30247598498022 -5.81953077098330 1.69237142458346<br>H 4.33696289794046 -5.60569672296069 1.92766750645389<br>C 2.52316094577554 -6.54840934849662 2.58168064451052<br>H 2.94270925254668 -6.90416508300062 3.51361898835846<br>C 1.20075907479020 -6.83040326707154 2.27698386208957<br>H 0.60494101386383 -7.40334066683252 2.97767502834663<br>C -2.04160405402540 -5.48572934909048 1.66394355278399<br>C -3.40851651538494 -5.68920895797521 1.85137956054010<br>H -3.88712702749098 -6.58039860749165 1.46181701774663<br>C -4.16281189879977 -4.75581424978864 2.54422566064642<br>H -5.22246570595653 -4.92091283173948 2.68796491701211<br>C -3.55622233199486 -3.61678048273366 3.06046797801222<br>H -4.14396052010716 -2.89192597291903 3.60855901221374<br>C -2.19643202838380 -3.41359328255284 2.87839807976227<br>H -1.71860855139940 -2.53281585963901 3.28634796196575<br>C -1.43835439377951 -4.34473708292663 2.18206588720394<br>H -0.37627944461511 -4.17953218684149 2.05744324716101	**RC$_{syn}$ (1$_{OMe}$ and 2$_{NMe2}$)**<br>$E_{PBEh-3c/CPCM(Chloroform)}$=<br>-2337.740776931885<br>$ZPE_{PBEh-3c/CPCM(Chloroform)}$=<br>0.69478044<br>$FEC_{(298.15)PBEh-3c/CPCM(Chloroform)}$=<br>0.62408461<br>$E_{PW6B95-D3(BJ)/def2-TZVPP/CPCM(Chloroform)}$=<br>-2345.778297567133<br>$E_{B3LYP-D3(BJ)/def2-TZVPP/CPCM(Chloroform)}$=<br>-2341.944519132767

	C -1.67689398695693 -7.96039700749344 4.77792110156405<br>C -1.48920597443784 -6.60085898007721 4.95334116968917<br>C -0.29611712204753 -6.08507733850980 5.47396507445687<br>C 0.69760536670228 -7.00697103581102 5.81367837894372<br>C 0.52410307293667 -8.37085467871630 5.61194092512005<br>C -0.66453826925137 -8.86569553288118 5.09431949018886<br>H -2.62406996214447 -8.31593632443387 4.38751553856009<br>H -2.28756704615985 -5.93359634748780 4.65589267248842<br>N -0.11577450352388 -4.70104641101090 5.59648834531452<br>H 1.63669359699649 -6.67606218421132 6.23560823310542<br>H 1.32194975410839 -9.05661345499134 5.87002041720328<br>O -0.79396168313997 -10.20284490295478 4.93567739833384<br>H -1.49582970574868 -10.39960686246560 4.30161888737684<br>C 1.19891474103378 -4.24119591458328 5.96788109792656<br>C -1.17511928417783 -3.96108641396524 6.25089649946815<br>H 1.48531318097191 -4.48933520923551 7.00015361926119<br>H 1.95380616521462 -4.65559614697860 5.29878700837092<br>H 1.23516228584247 -3.15640528637837 5.86953771450351<br>H -1.02102678616498 -2.89336935379744 6.09309213811268<br>H -2.14818673229734 -4.20703857406119 5.83306710077022<br>H -1.21422847924812 -4.14137567443996 7.33379190275625<br>C 5.06442275177785 -11.83351888985060 1.09876422658577<br>H 5.13392050240332 -11.54187164603234 2.15040119722311<br>H 6.01630033096105 -12.26001127594776 0.79162501154919<br>H 4.87065554138847 -10.93982198418227 0.49903293045519	
80	Au -1.54157405813574 -8.87813493716745 2.07155683417687<br>P -1.16656862276368 -6.86141932101140 1.03755213644076<br>O -2.01162304364247 -12.44226634718821 1.28728248532276<br>C -1.22603500745794 -11.65953053104399 2.04915286132278<br>C -1.95391884049376 -10.83753643590401 2.90788526996167<br>C -3.33546640678634 -11.21764467850342 2.63180818015519<br>C -4.56989802707140 -10.82038357845124 3.13933177304795<br>H -4.63152999615054 -10.07124845112476 3.91867388777145<br>C -5.71427543171063 -11.40561316561678 2.62058931843271<br>H -6.68368484267949 -11.11294729044018 3.00222231956363<br>C -5.64363026333474 -12.37092020861757 1.61112063224228<br>H -6.55605790838418 -12.80800659818695 1.22794087818805<br>C -4.42374721425415 -12.78352478997982 1.09261638429686<br>H -4.35922241331432 -13.53239354129942 0.31448526105586<br>C -3.30271347656438 -12.18640748159100 1.63337535940491<br>C 0.20561219459268 -11.81986223696989 1.88237586385482<br>C 1.07436680082137 -11.33151933111698 2.85825042296134<br>H 0.67487002682562 -10.88116148217189 3.75790472599620<br>C 2.44624032156677 -11.43926577486662 2.71658264274106<br>H 3.07939597225598 -11.05003100981942 3.50192690866271<br>C 2.98149643101950 -12.04960185491606 1.58164295794673<br>C 2.11801298096779 -12.55733195183154 0.60633515022825<br>H 2.53251009580338 -13.03582305025278 -0.27173734449365<br>C 0.75258421376015 -12.44619621573681 0.75645218591676<br>H 0.11073851036061 -12.84007497369588 -0.02027828742250<br>O 4.29263275384488 -12.19581025316553 1.34731262141766<br>C -1.67073741445832 -6.82610141803763 -0.70810636352031<br>C -1.40164893704065 -7.94138386285571 -1.50025175663663<br>H -0.93390313140509 -8.81851025449009 -1.06813371792645<br>C -1.73323871748307 -7.93553371300223 -2.84582706366524<br>H -1.52147543293644 -8.80447474020461 -3.45465734512840<br>C -2.34387799208827 -6.82112675930549 -3.40707341645951<br>H -2.60891680789659 -6.82023766498692 -4.45626459393340<br>C -2.61884701965555 -5.71173794827378 -2.62064033760307<br>H -3.09660483529982 -4.84289266485905 -3.05375067860532<br>C -2.28211816866242 -5.71043499236255 -1.27385247707487<br>H -2.49956486107334 -4.83837769763953 -0.67063011574577<br>C 0.58432217245023 -6.38372957595347 1.03464966745830<br>C 1.12753518237524 -5.61955107331213 0.00367651510456<br>H 0.51435429668908 -5.30763179142724 -0.83218912300858<br>C 2.46597532413720 -5.25769545727240 0.03988326136999<br>H 2.88318083608575 -4.66670950699550 -0.76470591482834<br>C 3.26739785915277 -5.65597460829824 1.10175064677974<br>H 4.31260488299994 -5.37632239900342 1.12491899638075<br>C 2.72877511447223 -6.41776425854892 2.12937464564189<br>H 3.34933329553717 -6.73518205689340 2.95726368791403<br>C 1.39137724137181 -6.78401930993463 2.09744197464536<br>H 0.98467292414234 -7.37845021042772 2.90694754612294<br>C -2.07007505231439 -5.49924680088216 1.82332957198633<br>C -3.43386107314587 -5.67233831479599 2.06151929482548	**TS$_{syn}$ (1$_{OMe}$ and 2$_{NMe2}$)**<br>E$_{PBEh\text{-}3c/CPCM(Chloroform)}$=<br>-2337.701902646888<br>ZPE$_{PBEh\text{-}3c/CPCM(Chloroform)}$=<br>0.68981007<br>FEC$_{(298.15)PBEh\text{-}3c/CPCM(Chloroform)}$=<br>0.62050108<br>E$_{PW6B95\text{-}D3(BJ)/def2\text{-}TZVPP/CPCM(Chloroform)}$=<br>-2345.741795693980<br>E$_{B3LYP\text{-}D3(BJ)/def2\text{-}TZVPP/CPCM(Chloroform)}$=<br>-2341.912413009313

	H	-3.92722455551772	-6.59384838329612	1.77359726110234	
	C	-4.16423095475247	-4.66901156432401	2.67510918687436	
	H	-5.22151562398903	-4.80843608593679	2.85788745519945	
	C	-3.53627213726664	-3.49045318197906	3.06345842519437	
	H	-4.10568827524201	-2.70928456986434	3.55002874861010	
	C	-2.18025730785857	-3.31964937306702	2.83257697455862	
	H	-1.68644980895587	-2.40773564754753	3.14096755353235	
	C	-1.44421788992710	-4.32181213506413	2.21382435087712	
	H	-0.38445575554746	-4.18050280114018	2.05027977332663	
	C	-1.79856793112488	-7.96754041177999	5.03816568310373	
	C	-1.44834336563061	-6.62541980011958	5.02871197593709	
	C	-0.14563814335904	-6.20270352026798	5.30518704865378	
	C	0.79331807275159	-7.20239663850800	5.58421399267901	
	C	0.45463606169222	-8.54764315568891	5.56467787727679	
	C	-0.86143533062646	-8.98909986999642	5.32323094003056	
	H	-2.83716479676779	-8.23881182489730	4.87680450105469	
	H	-2.21421475200948	-5.89857690520711	4.78693813736632	
	N	0.18793844494915	-4.83459756046801	5.25792256474087	
	H	1.81849817869767	-6.94275350583042	5.81550471999173	
	H	1.21850680400731	-9.28564149256326	5.78531332430630	
	O	-1.18677943701618	-10.25296148009040	5.33100954101807	
	H	-1.58029786315229	-10.56940346121243	4.08120908034541	
	C	1.58026884848752	-4.49966429508742	5.41644585099593	
	C	-0.67884673273275	-3.94232822352638	5.99904271149484	
	H	1.98056266746283	-4.70645470982496	6.42037023123418	
	H	2.19155316285199	-5.04017984740439	4.69335411940436	
	H	1.71358294127641	-3.43492899365546	5.22388742535256	
	H	-0.43915382124732	-2.90926020491623	5.74438749449722	
	H	-1.72308336243248	-4.09863934603376	5.73988494410597	
	H	-0.57875939251902	-4.05452855573227	7.08799432690709	
	C	5.21348915368037	-11.69330195562995	2.29407846328253	
	H	5.10629781143602	-12.18066065703476	3.26655039493904	
	H	6.20478401877062	-11.90753175312778	1.90285185933989	
	H	5.11286373558273	-10.61294749674287	2.42694642637946	
81	Au	-1.04522040394806	-7.66700290001786	3.32584825378019	**PC$_{syn}$ (1$_{OMe}$ and 2$_{NMe2}$)**
	P	-0.57845650609371	-6.64269498705834	1.31459633408213	$E_{PBEh\text{-}3c/CPCM(Chloroform)}$=
	O	-2.02422507245290	-11.79848208662900	0.67842268236320	-2337.724703416256
	C	-1.15287529882073	-11.47502635258898	1.67853329579962	$ZPE_{PBEh\text{-}3c/CPCM(Chloroform)}$=
	C	-1.79794435123404	-11.35836109340316	2.86708369910450	0.69435011
	C	-3.18114444320838	-11.61421737509538	2.59937366626900	$FEC_{(298.15)PBEh\text{-}3c/CPCM(Chloroform)}$=
	C	-4.35279607757001	-11.64011549103213	3.35726949181516	0.62362694
	H	-4.33016161881039	-11.43830114455521	4.42044724868162	$E_{PW6B95\text{-}D3(BJ)/def2\text{-}TZVPP/CPCM(Chloroform)}$=
	C	-5.54422829617467	-11.92866847217999	2.71413296700189	-2345.764316069811
	H	-6.46495115415634	-11.95367190757827	3.28215850281056	$E_{B3LYP\text{-}D3(BJ)/def2\text{-}TZVPP/CPCM(Chloroform)}$=
	C	-5.58471872291454	-12.19054017534632	1.33933609670258	-2341.941276427811
	H	-6.53316412693401	-12.41268632883175	0.86855823297570	
	C	-4.43240292667005	-12.17012491734722	0.56693313752624	
	H	-4.45676280708625	-12.37111601090751	-0.49603711810547	
	C	-3.25507822339316	-11.87900272243075	1.22947813114386	
	C	0.23601771852855	-11.28370892614190	1.30238127986345	
	C	1.17532347561825	-10.90853764124242	2.26287329747965	
	H	0.87691077300039	-10.76983030137186	3.29509726833382	
	C	2.50445458197163	-10.70752047911507	1.93136180287087	
	H	3.19251892917941	-10.41852732375694	2.71369773561748	
	C	2.92779203553870	-10.87336144006918	0.61335592790365	
	C	1.99464427804779	-11.24020126724984	-0.35742616466660	
	H	2.31699005973992	-11.36882031106735	-1.38292665355277	
	C	0.67261291477532	-11.44406867584980	-0.01726754765297	
	H	-0.02160239082346	-11.73331803634498	-0.79493897921548	
	O	4.19331676877837	-10.70668915192453	0.19464945833012	
	C	-1.72092016961004	-7.11876233653926	-0.01302349069090	
	C	-1.85629402925660	-8.47676247675516	-0.29783846423086	
	H	-1.26341880600513	-9.20113357999118	0.24515082226517	
	C	-2.72731386351769	-8.89893419248649	-1.28781752439587	
	H	-2.82087328249943	-9.95444402897169	-1.50523555217492	
	C	-3.48009978397023	-7.96656475669850	-1.99174167924311	
	H	-4.16696399202381	-8.29499358287654	-2.76072666819838	
	C	-3.35244394317385	-6.61512403900069	-1.70692655925014	
	H	-3.93778111770035	-5.88714126294294	-2.25292637506053	
	C	-2.47302725438525	-6.18697880692330	-0.72054969774703	
	H	-2.38284934663909	-5.12937446812839	-0.51028777470609	
	C	1.09112024420111	-6.98268570139663	0.68727003668756	
	C	1.33852943086595	-7.29453184779425	-0.64610664110902	
	H	0.52323467979934	-7.37580969361896	-1.35301113927179	
	C	2.64093756225398	-7.50478470387352	-1.07847183222963	

	H 2.82656191253053 -7.75468175291326 -2.11462302207836<br>C 3.69820754568668 -7.39366217610330 -0.18836509440146<br>H 4.71229517062102 -7.55489761903155 -0.52952842936703<br>C 3.45391046108770 -7.07907660479382 1.14241131649917<br>H 4.27542749499344 -6.99272680025499 1.84148816496725<br>C 2.15523370926261 -6.88408082074914 1.58295107983343<br>H 1.97337050411881 -6.65374420293887 2.62652485005138<br>C -0.67801178187225 -4.83110537029263 1.41483858891059<br>C -1.57033437079189 -4.24503045184196 2.31015506982760<br>H -2.17528651500795 -4.86109476349716 2.96464611567211<br>C -1.68862422393986 -2.86467794168067 2.37368991125146<br>H -2.38175215984081 -2.41771293719110 3.07404207858796<br>C -0.91313265280110 -2.06209944945145 1.54837002872960<br>H -1.00106499649589 -0.98491794197171 1.60292597637693<br>C -0.01988635360209 -2.64166561065705 0.65726974615829<br>H 0.58857993474738 -2.01896369598738 0.01492285607203<br>C 0.09874547316118 -4.02181234347664 0.58821561038953<br>H 0.80051148437560 -4.46208089105517 -0.10878831818742<br>C -1.56872295930715 -8.45076606886696 5.30064333774841<br>C -2.00291882131013 -7.18790255731544 5.86782618845153<br>C -1.13891588383643 -6.34670446410239 6.50857673297071<br>C 0.21607108770953 -6.78359581663019 6.64450418877667<br>C 0.64362738629848 -8.00697728290219 6.23904873496183<br>C -0.26822264019744 -8.99588562921682 5.68073122821711<br>H -2.35733714914996 -9.18886849087583 5.14640556204246<br>H -3.04557482961542 -6.92373971478192 5.73887990236818<br>N -1.47249981143929 -5.05876115988058 6.97648037847536<br>H 0.93501751324217 -6.10148310701725 7.08526678893361<br>H 1.67266102294124 -8.30373083093503 6.40635252451745<br>O 0.05965856288614 -10.18378958609109 5.53591436756760<br>H -1.34616104076931 -11.11808560129270 3.81804773077877<br>C -1.28052711904634 -4.85919982432808 8.40087879265748<br>C -2.73464275903039 -4.52863296392251 6.52528553407721<br>H -2.02339979248016 -5.39365628703705 9.00957811446193<br>H -0.29289328304096 -5.19423600321099 8.71325974191600<br>H -1.35398204907629 -3.79646893875655 8.63510643792519<br>H -2.80880728606845 -3.48175847501379 6.82143385608354<br>H -2.79790542113761 -4.57385947241060 5.43715724934983<br>H -3.61173097710158 -5.04971104033095 6.93728936362296<br>C 5.19117125362377 -10.43321659768474 1.15435217436532<br>H 5.27222981505574 -11.23210720645290 1.89671084454466<br>H 6.13085811997038 -10.36258005463156 0.61172829511596<br>H 5.01454154155076 -9.48808897603040 1.67390871197443	
82	Au 1.23417223354296 -4.97138696462534 -2.09418087347223<br>P 0.98929929681843 -4.02235263669763 -0.06929657821998<br>C 0.47738342538067 -2.28060002788693 -0.13527897573965<br>C 1.06660826871585 -1.44997141071832 -1.08783068438196<br>H 1.78910710058773 -1.84804073510825 -1.79072061815245<br>C 0.73010614773917 -0.10682056340092 -1.14258228597066<br>H 1.19157763358863 0.53210352744389 -1.88372844840941<br>C -0.20295082124494 0.41335897315488 -0.25455142955448<br>H -0.47020726952494 1.46086759252177 -0.30271792129225<br>C -0.79610297738717 -0.41167850419904 0.68990597958105<br>H -1.52597212633089 -0.01078776513571 1.38072394977655<br>C -0.45678876529683 -1.75630553296289 0.75350208129054<br>H -0.92595648613606 -2.38910515913217 1.49565190879155<br>C 2.52606899188284 -4.03492467258803 0.89914883941225<br>C 2.91722896054353 -2.94206834254312 1.66754821502411<br>H 2.32025275424167 -2.03950102406848 1.68503224062475<br>C 4.08382857149775 -3.00336765284139 2.41743719234681<br>H 4.38463902751166 -2.14939025169007 3.00977404496847<br>C 4.86000541877664 -4.15318330150714 2.40699265071250<br>H 5.76980627165105 -4.19773388722938 2.99118061934556<br>C 4.47214942287862 -5.24555750056936 1.64151058897786<br>H 5.07692470139510 -6.14249188510028 1.62667730656128<br>C 3.31187895811642 -5.18685709784651 0.88603642098117<br>H 3.02159365576581 -6.04173548934749 0.28646582551073<br>C -0.24555506910259 -4.84664763574824 0.97683214423901<br>C -1.44714656773296 -5.25037340170130 0.39588162643870<br>H -1.62122291044594 -5.10023705875751 -0.66315317097157<br>C -2.42759371043580 -5.84918951515244 1.17074866297402<br>H -3.35754896023765 -6.16006567211803 0.71356317887529<br>C -2.21169845056170 -6.05713625513663 2.52717120007797<br>H -2.97512063233100 -6.53121803006782 3.13001165152063<br>C -1.01492944805958 -5.66228225361108 3.10722188381966	**4-NMe2-PhOAuPPh$_3$**<br>$E_{PBEh-3c/CPCM(Chloroform)}$=<br>-1610.044587725454<br>$ZPE_{PBEh-3c/CPCM(Chloroform)}$=<br>0.45575609<br>$FEC_{(298.15)PBEh-3c/CPCM(Chloroform)}$=<br>0.39912538<br>$E_{PW6B95-D3(BJ)/def2-TZVPP/CPCM(Chloroform)}$=<br>-1615.260303948799

	H	-0.84190483024111	-5.82593167761817	4.16263430172015	
	C	-0.03251303213561	-5.05582538108190	2.33643182941617	
	H	0.89652557567668	-4.75188831066517	2.80104472909748	
	C	2.37382163056712	-6.74887673797585	-5.81653332583834	
	C	3.48701931011260	-7.03261851366598	-6.59071214754643	
	C	4.78053520955415	-6.67688667455036	-6.18297707226526	
	C	4.87673001404582	-5.99767809831233	-4.96278754538132	
	C	3.75869111217518	-5.71518030185046	-4.18878445807567	
	C	2.46860737376269	-6.08291933315508	-4.58654005378866	
	H	1.39592133316106	-7.04680186357419	-6.17711544191116	
	H	3.32501880496821	-7.54461600773743	-7.52945366627232	
	H	5.83680087108236	-5.67206725245763	-4.58624699564224	
	H	3.90918215784579	-5.18555823596109	-3.25296273877095	
	O	1.35921786039203	-5.83944785088235	-3.90083381224537	
	N	5.90726237084014	-6.99860693393109	-6.94170084739547	
	C	7.13574749265431	-6.30810573325231	-6.63583397501128	
	H	7.46717857832585	-6.52298232002043	-5.61870824457953	
	H	7.92040376405281	-6.65921760880395	-7.30312508739717	
	H	7.06625120206907	-5.21665467929478	-6.74008954527165	
	C	5.70306198971171	-7.33632973017068	-8.32939375356530	
	H	6.66453085441691	-7.55308722493751	-8.79077856012270	
	H	5.09450316426000	-8.23586820967884	-8.43304614001164	
	H	5.22093937489569	-6.53868079705113	-8.91063013782705	

6. References

(1) Taschinski, S. Gold(I) Catalysis: Mechanistic Insights, Reactivity of Intermediates and its Applications. *University of Groningen Press* **2020**, DOI: 10.33612/diss.126022756.

(2) Pangborn, A. B., Giardello, M. A., Grubbs, R. H., Rosen, R. K., Timmers, F. J. Safe and Convenient Procedure for Solvent Purification. *Organometallics* **1996**, *15*, 1518-1520.

(3) Wegner, H., Auzias, M., Neuburger, M. 3,3′-Bis(arylbenzofurans) via a Gold-Catalyzed Domino Process. *Synlett* **2010**, *2010*, 2443-2448.

(4) (a) Hashmi, A. S. K., Ramamurthi, T. D., Rominger, F. On the Trapping of Vinylgold Intermediates. *Adv. Synth. Catal.* **2010**, *352*, 971-975; (b) Hashmi, A. S. K. Homogeneous gold catalysis beyond assumptions and proposals-characterized intermediates. *Angew. Chem. Int. Ed.* **2010**, *49*, 5232-5241.

(5) Liu, J., Liu, Y. Gold-Catalyzed Cyclizations of (*o*-Alkynyl)phenoxyacrylates with External Nucleophiles: Regio- and Stereoselective Synthesis of Functionalized Benzo[*b*]oxepines. *Org. Lett.* **2012**, *14*, 4742-4745.

(6) Taschinski, S., Dopp, R., Ackermann, M., Rominger, F., de Vries, F., Menger, M. F. S. J., Rudolph, M., Hashmi, A. S. K., Klein, J. E. M. N. Light-Induced Mechanistic Divergence in Gold(I) Catalysis: Revisiting the Reactivity of Diazonium Salts. *Angew. Chem. Int. Ed.* **2019**, *58*, 16988-16993.

(7) Hansch, C., Leo, A., Taft, R. W. A Survey of Hammett Substituent Constants and Resonance and Field Parameters. *Chem. Rev.* **1991**, *91*, 165-195.

(8) Grimme, S., Brandenburg, J. G., Bannwarth, C., Hansen, A. Consistent structures and interactions by density functional theory with small atomic orbital basis sets. *J. Chem. Phys.* **2015**, *143*, 054107.

(9) Barone, V., Cossi, M. Quantum calculation of molecular energies and energy gradients in solution by a conductor solvent model. *J. Phys. Chem. A* **1998**, *102*, 1995-2001.

(10) Lange, A. W., Herbert, J. M. A smooth, nonsingular, and faithful discretization scheme for polarizable continuum models: The switching/Gaussian approach. *J. Chem. Phys.* **2010**, *133*, 244111

(11) (a) Grimme, S., Antony, J., Ehrlich, S., Krieg, H. A consistent and accurate ab initio parametrization of density functional dispersion correction (DFT-D) for the 94 elements H-Pu. *J. Chem. Phys.* **2010**, *132*, 154104; (b) Grimme, S., Ehrlich, S., Goerigk, L. Effect of the damping function in dispersion corrected density functional theory. *J. Comput. Chem.* **2011**, *32*, 1456-1465; (c) Zhao, Y., Truhlar, D. G. Design of density functionals that are broadly accurate for thermochemistry, thermochemical kinetics, and nonbonded interactions. *J. Phys. Chem. A* **2005**, *109*, 5656-5667.

(12) Weigend, F., Ahlrichs, R. Balanced basis sets of split valence, triple zeta valence and quadruple zeta valence quality for H to Rn: Design and assessment of accuracy. *Phys. Chem. Chem. Phys.* **2005**, *7*, 3297-3305.

(13) Kruse, H., Goerigk, L., Grimme, S. Why the Standard B3LYP/6-31G* Model Chemistry Should Not Be Used in DFT Calculations of Molecular Thermochemistry: Understanding and Correcting the Problem. *J. Org. Chem.* **2012**, *77*, 10824-10834.

(14) Lu, T., Chen, F. Multiwfn: A multifunctional wavefunction analyzer. *J. Comput. Chem.* **2012**, *33*, 580-592.

(15) Humphrey, W., Dalke, A., Schulten, K. VMD: Visual molecular dynamics. *J. Mol. Graph.* **1996**, *14*, 33-38.
